# Topologically Controlled Syntheses of Unimolecular Oligo[*n*]catenanes

**DOI:** 10.1021/acscentsci.2c00697

**Published:** 2022-11-29

**Authors:** Nathan D. Colley, Mark A. Nosiglia, Sheila L. Tran, Gray H. Harlan, Christy Chang, Ruihan Li, Abigail O. Delawder, Yipei Zhang, Jonathan C. Barnes

**Affiliations:** Department of Chemistry, Washington University, St. Louis, Missouri 63130, United States

## Abstract

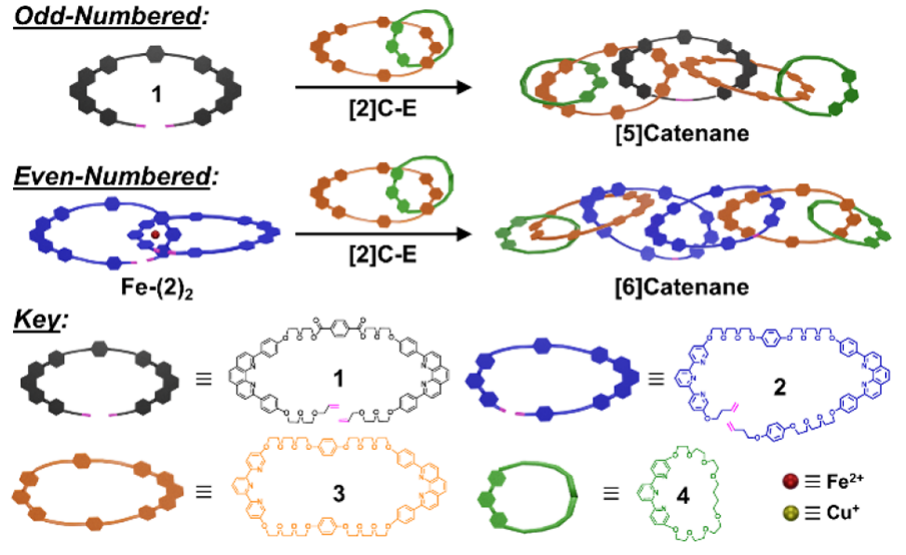

Catenanes are a well-known class of mechanically interlocked molecules that possess chain-like architectures and have been investigated for decades as molecular machines and switches. However, the synthesis of higher-order catenanes with multiple, linearly interlocked molecular rings has been greatly impeded by the generation of unwanted oligomeric byproducts and figure-of-eight topologies that compete with productive ring closings. Here, we report two general strategies for the synthesis of oligo[*n*]catenanes that rely on a molecular “zip-tie” strategy, where the “zip-tie” is a central core macrocycle precursor bearing two phenanthroline (phen) ligands to make odd-numbered oligo[*n*]catenanes, or a preformed asymmetric iron(II) complex consisting of two macrocycle precursors bearing phen and terpyridine ligands to make even-numbered oligo[*n*]catenanes. In either case, preformed macrocycles or [2]catenanes are threaded onto the central “zip-tie” core using metal templation prior to ring-closing metathesis (RCM) reactions that generate several mechanical bonds in one pot. Using these synthetic strategies, a family of well-defined linear oligo[*n*]catenanes were synthesized, where *n* = 2, 3, 4, 5, or 6 interlocked molecular rings, and *n* = 6 represents the highest number of linearly interlocked rings reported to date for any *isolated* unimolecular oligo[*n*]catenane.

## Abstract 2

The synthesis of a series of oligo[*n*]catenanes (*n* = 2, 3, 4, 5, and 6) was achieved using a molecular “zip-tie” strategy involving orthogonal metal templation and ring-closing metathesis chemistry.

## Introduction

In comparison to the most well-studied types of chemical bonds—covalent, ionic, and metallic—the mechanical bond remains relatively underexplored. Mechanical bonding occurs when two or more molecular species are interlocked via a physical entanglement in space, such that they cannot be separated without breaking covalent bonds.^1^ The two main classes of mechanically interlocked molecules (MIMs)^2^ are rotaxanes^3^ and catenanes,^4^ the former consisting of a molecularly shaped dumbbell threaded through one or more macrocycles, and the latter being constructed from two or more linearly, radially, or cyclically interlocked macrocycles. Of the two types of MIMs, catenanes^5^ are generally more challenging to synthesize because the ring-closing reactions required to establish the mechanical bond are notorious for generating non-interlocked byproducts^6^ and so-called figure-of-eight topologies.^7^ Linear catenanes are of particular interest because this topology maximizes the conformational degrees of freedom through rotations, translations, and rocking motions of the molecular rings.^6^ To facilitate catenation, template-directed syntheses operating under thermodynamic control have been pursued. These include metal-coordination,^8^ donor–acceptor,^9^ hydrogen-bonding,^10^ and anion–dipole^11^ interactions, to name some. For example, Stoddart and co-workers capitalized on charge-transfer interactions between π-electron-deficient and -rich macrocycles to successfully synthesize a linear [5]catenane they called “Olympiadane”.^12,13^ More recently, Iwamoto and co-workers used hydrogen bonding to template the synthesis of another linear [5]catenane.^14^ To date, both of these examples have stood as the record for the total number of interlocked macrocycles isolated as a linear, unimolecular oligo[*n*]catenane, even though Stoddart and co-workers did report by mass spectrometry a linear [7]catenane that was never isolated.^15^

Out of all the template-directed strategies, however, metal-coordination is usually the most efficient for assembling molecular precursors. Sauvage and co-workers pioneered^16^ the use of orthogonal metal templation to selectively assemble and ring-close macrocyclic precursors to make functional asymmetric catenanes. The selective orthogonal metalation arises from extended phenanthroline (phen) ligands that favor the formation of tetracoordinate complexes with monovalent metals (e.g., Cu^+^), and terpyridine (terpy) ligands that favor the corresponding hexacoordinate complexes with bivalent metals (e.g., Fe^2+^). Metal-based templation has also been used to construct catenanes of complex topology, such as Leigh and co-workers’ interwoven Star of David [2]catenane^17^ and Nitschke and co-workers’ cyclic [3]catenane^18^—both of which were preassembled using Fe^2+^-based helicates. Outside of well-defined unimolecular catenanes, there has also been interest in using [2]catenanes as building blocks in poly[2]catenanes,^19^ or as cross-linkers in polymer networks.^20^ Moreover, Di Stefano and co-workers demonstrated^21^ a fast route to synthesize a broad mixture of poly[*n*]catenates by performing ring-opening metathesis polymerizations on a copper(I) complex of a phen-based [2]catenane. Rowan and co-workers^22,23^ also adopted a one-pot approach, preassembling polydisperse metallosupramolecular polymers (MSPs) via metal coordination of open and closed macrocycles with zinc ions (i.e., Zn(II)), which were subjected to ring-closing metathesis (RCM) that produced a mixture of mostly linear and some branched and circular poly[*n*]catenanes. Lastly, Yagai and co-workers^24^ investigated the formation of supramolecular poly[*n*]catenanes through the self-assembly of kinetically produced toroids, where the latter catenane structures can disassemble at elevated temperatures.

**Figure 1:**
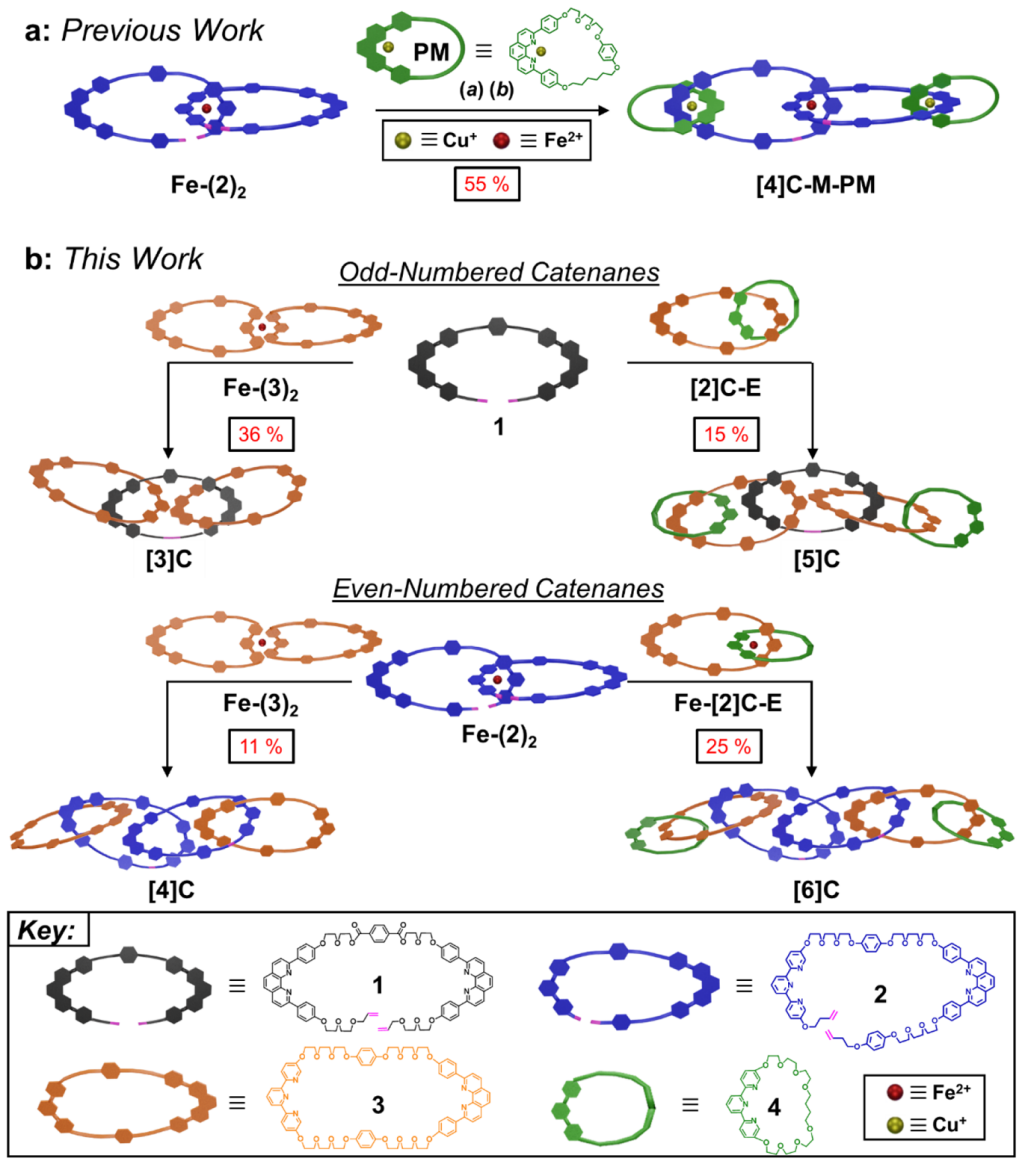
Overview of the “zip-tie” strategies to synthesize linear oligo[*n*]catenanes (*n* is the number of interlocked macrocycles). (a) Previous synthetic approach to prepare a metalated [4]catenane, **[4]C-M-PM**, in 55% yield. (b) This work, where single and double “zip-tie” synthetic strategies allowed for the synthesis of odd- and even-numbered [*n*]catenanes, respectively.

Even with these examples, there remains a fundamental synthetic gap between well-defined oligo[*n*]catenanes and the more broadly dispersed poly[*n*]catenanes. Oligo[*n*]catenanes, for example, can be made on a larger scale through moderately high-yielding reactions; however, they are usually of low molecular weight and possess “dead end” macrocycles that cannot be extended further (Figure 1a, previous work^25^ and others^26,27^). Poly[*n*]catenane syntheses may require fewer synthetic steps (i.e., one-pot reactions) and result in higher molecular weight polymers; yet, their syntheses lack control and generate product mixtures that are often difficult to purify. Here, we describe two molecular “zip-tie” synthetic strategies (Figure 1b) that build off our previous efforts to bridge this fundamental gap. The molecular “zip-tie” utilizes an open macrocycle with two phen ligands that are threaded through preformed macrocycles or [2]catenanes via coordination with copper(I), followed by a RCM step to produce odd-numbered linear oligo[2*n*+1]catenanes. Even-numbered [*n*]catenanes were synthesized as well via a double “zip-tie” approach by implementing sequential, orthogonal metal templation steps of dual-ligand macrocyclic precursors that were complexed with two transition metal ions (iron(II) and copper(I)) and preformed macrocycles or [2]catenanes. In the final step, two simultaneous RCM reactions on the precatenate metal complexes yielded well-defined, oligo[2*n*+2]catenanes. The sequential metal-coordinated assembly of the precursors prevented the formation of unwanted figure-of-eight topologies that often arise during simultaneous ring-closing reactions,^7,28−31^ a critical outcome that was proven by comparing the products generated from the syntheses of [2]- and [4]catenanes. The full utility of the double “zip-tie” strategy was demonstrated by synthesizing a linear [6]catenane, which, to the best of our knowledge, represents the highest number of linearly interlocked macrocycles ever isolated for a unimolecular catenane. This work sets the stage for future efforts to expand the syntheses toward the preparation of poly[*n*]catenanes, either with all mechanical bonds, or through the polymerization of terminal functional groups.^32^

## Results and Discussion

**Figure 2:**
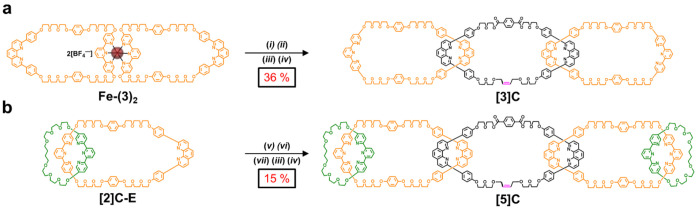
| “Zip-tie” strategies to synthesize odd-numbered linear oligo[*n*]catenanes. (a) (*i*) CuI, MeCN, **1**, 25 °C, 2 days; (*ii*) Grubbs’ second-generation catalyst, CH_2_Cl_2_, 35 °C, 4 days; (*iii*) K_2_CO_3_, DMF, 75 °C, 1 day. (*iv*) KCN, MeCN/H_2_O, 25 °C, 2 h. (b) (*v*) FeCl_2_, CH_2_Cl_2_, DMF, 25 °C, 1 h; (*vi*) CuI, MeCN, **1**, 25 °C, 18 h; (*vii*) Grubbs’ second-generation catalyst, CH_2_Cl_2_, 35 °C, 1 day; (*iii*) K_2_CO_3_, DMF, 75 °C, 1 day. (*iv*) KCN, MeCN/H_2_O, 25 °C, 2 h.

**Figure 3:**
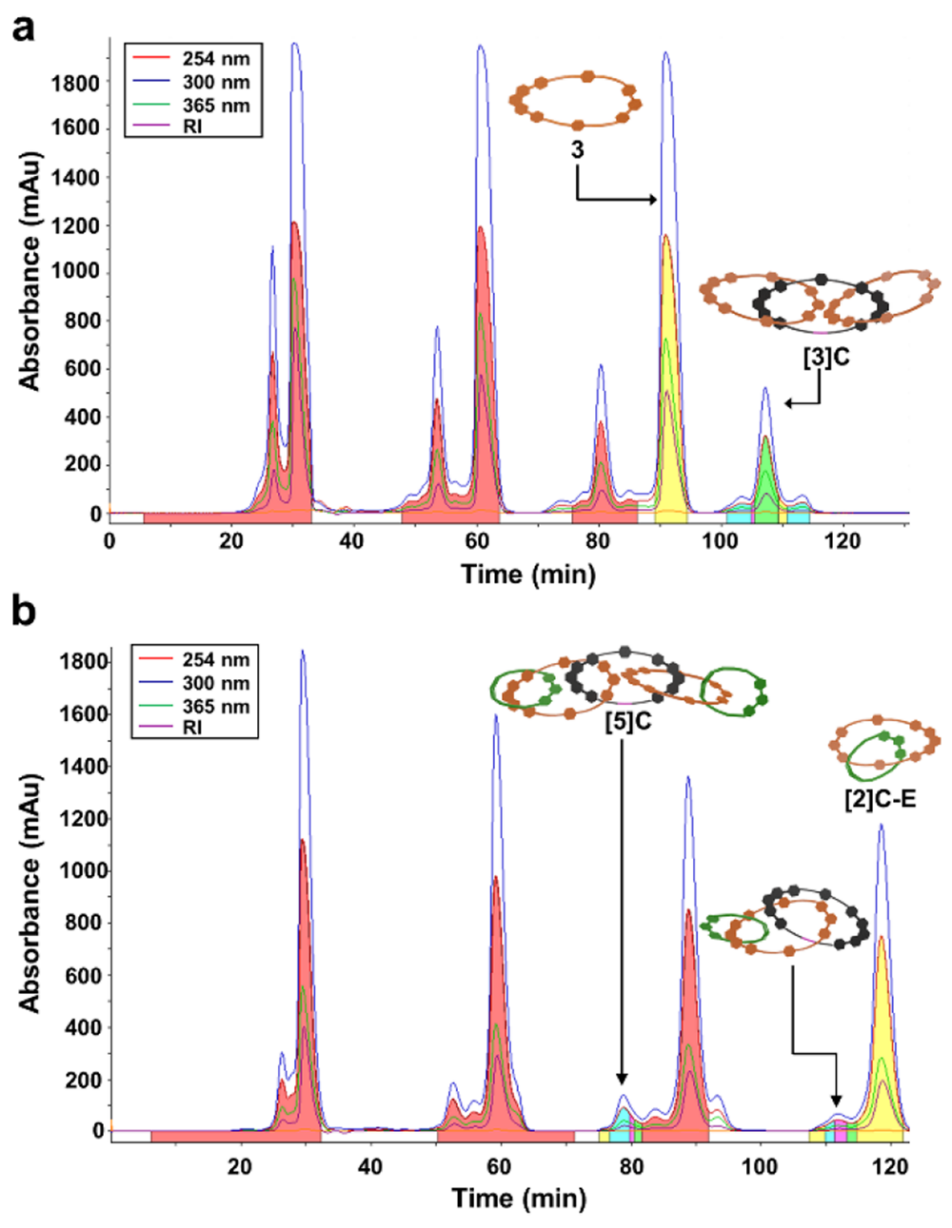
Recycling prep-GPC data for **[3]C** (a) and **[5]C** (b) collected in a DMF mobile phase at 8 mL·min^–1^. Pure **[3]C** was isolated in the green fraction at 108 min. Pure **[5]C** was isolated in the blue fraction at 75 min.

**Figure 4:**
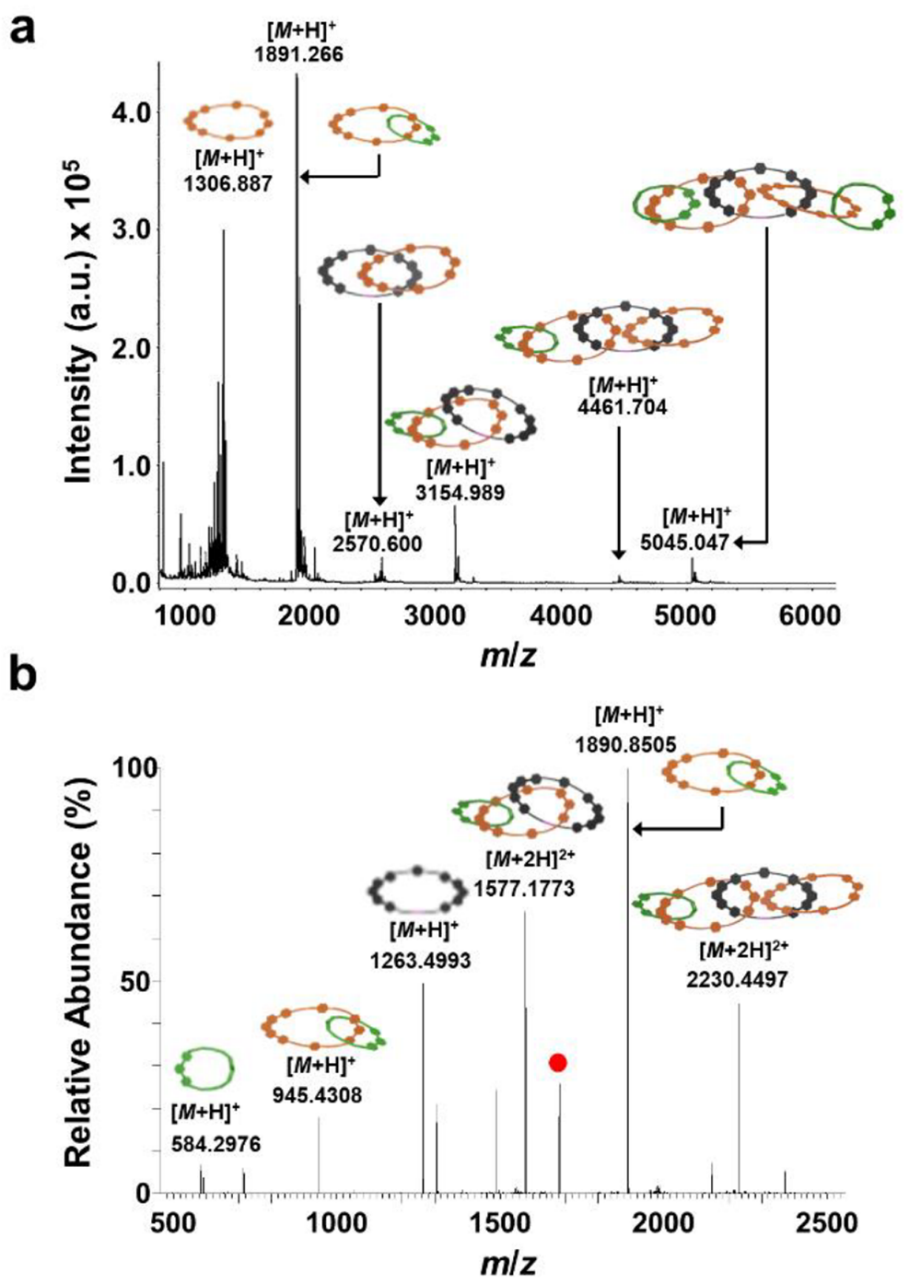
(a) MALDI-TOF-MS of **[5]C** with α-cyano-4-hydroxycinnamic acid matrix. (b) THRMS-ESI (i.e., MS/MS) of **[5]C**; the [*M* + 3H]^3+^ = 1682.06 Da peak (red dot) was isolated and fragmented.

The synthesis of linear [3]catenanes often proceeds by the coupling of two [2]pseudorotaxanes^33−36^ or through the ring-closing of a [3]pseudorotaxane,^37−39^ the latter of which was employed here for the synthesis of the linear [3]catenane **[3]C** (Figure 2a, Scheme S18). However, many [3]catenanes have been constructed using small monofunctionalized terminal macrocycles, often resulting in low molecular weight products with limited opportunity for further catenation. In order to quickly achieve higher molecular weight MIMs while retaining the potential to synthesize higher order catenanes, a “zip-tie” approach was implemented with open phen-based macrocycle **1** (Figure 1b and Schemes S1–S3) and a 73-membered dual-ligand molecular ring **3** (Figure 1b, and Schemes S10–S11) to synthesize **[3]C**. Initial efforts to directly complex macrocycle **1** with **3** were unsuccessful because **3** collapses during monometalation with Cu^+^, producing a heteroleptic Cu^+^-phen-terpy complex.^40^ This unwanted complexation was circumvented by first “blocking” the terpy ligands with Fe^2+^ to give the dimeric macrocycle complex **Fe-(3)**_**2**_ (Figure 2a, Scheme S14). With the terpy ligands occupied, the phen ligands of **Fe-(3)**_**2**_ were readily monometalated with CuI to give **Cu·Fe-(3)_2_**. The pre-[3]catenate complex was formed by the addition of **1** in CH_2_Cl_2_, which then underwent a single RCM reaction with Grubbs' catalyst. In order to simplify the purification, the crude, ring-closed products were demetalated in a two-step process (Figure 2a, Scheme S18) using K_2_CO_3_ in DMF to remove Fe^2+^, followed by KCN in acetonitrile (MeCN)/H_2_O to remove Cu^+^. After an aqueous workup, the demetalated mixture was purified via recycling prep-GPC (Figure 3a, Figure S57) to afford **[3]C**, which was isolated in a 36% yield over the final three steps (i.e., complexation, ring-closing, and demetalation). The lower yield is due to the formation of [2]catenane byproduct and higher molecular weight oligomers which were observed during purification by recycling prep-GPC. Moreover, although this yield is lower than that (55%) from our previously reported synthesis to make the monophen-macrocycle-terminated [4]catenate (Figure 1a, **[4]C-M-PM**), the terminal rings of **[3]C** can be used for further catenation reactions and growth toward higher molecular weight oligo[*n*]catenanes.

**Figure 5:**
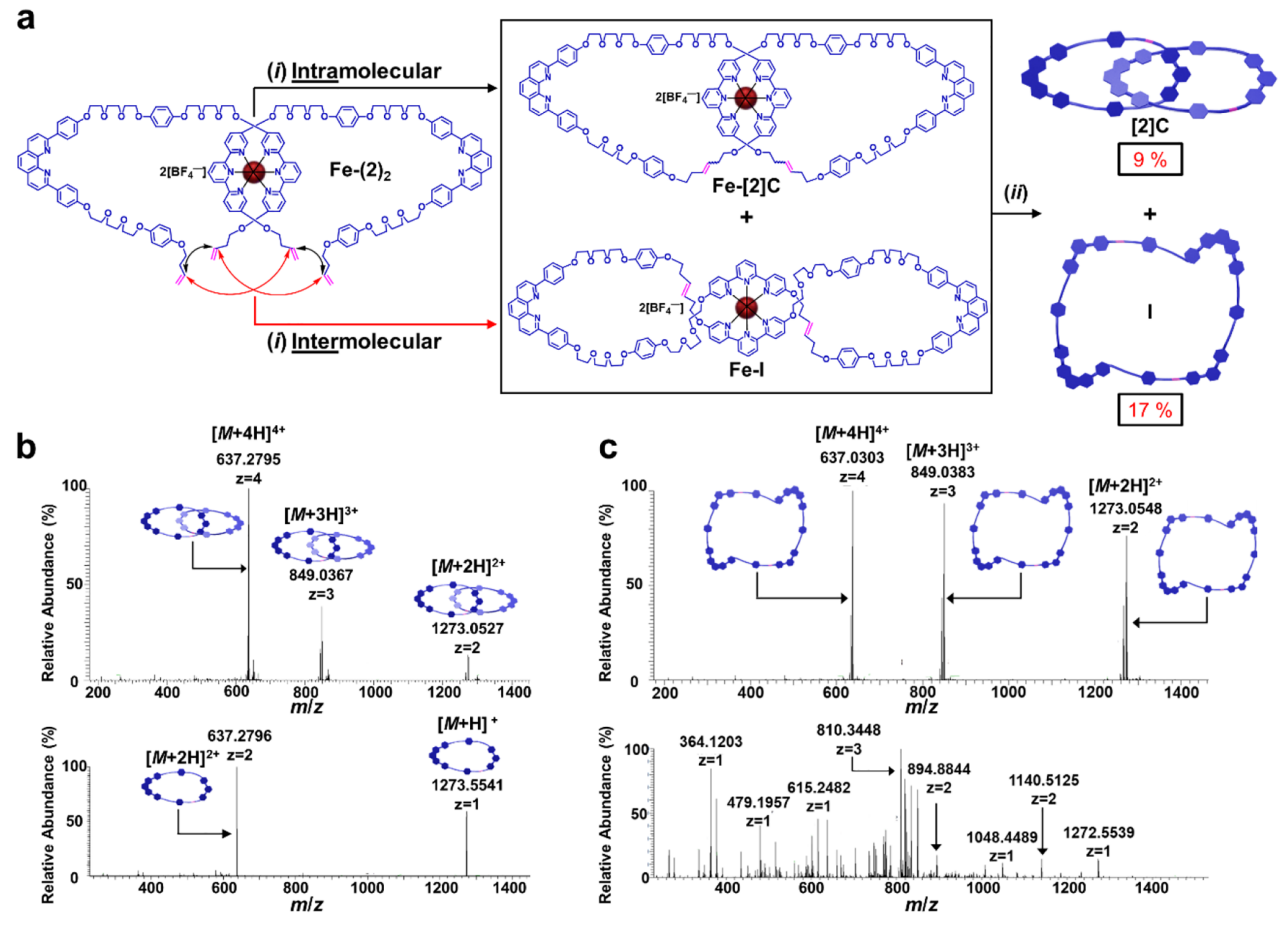
(a) Synthesis of topological products **[2]C** and **I**. (*i*) Grubbs’ second-generation catalyst, CH_2_Cl_2_, 35 °C, 1 day; (*ii*) Cs_2_CO_3_, DMF, 75 °C, 1 day. (b) Tandem high-resolution mass spectrometry–electrospray ionization (THRMS-ESI, i.e., MS/MS) of **[2]C**; the [*M* + 3H]^3+^ peak was isolated, and its fragmentation data are shown in the bottom spectrum. (c) THRMS-ESI of **I**; the [*M* + 3H]^3+^ peak was isolated, and its fragmentation data are shown in the bottom spectrum.

**Figure 6:**
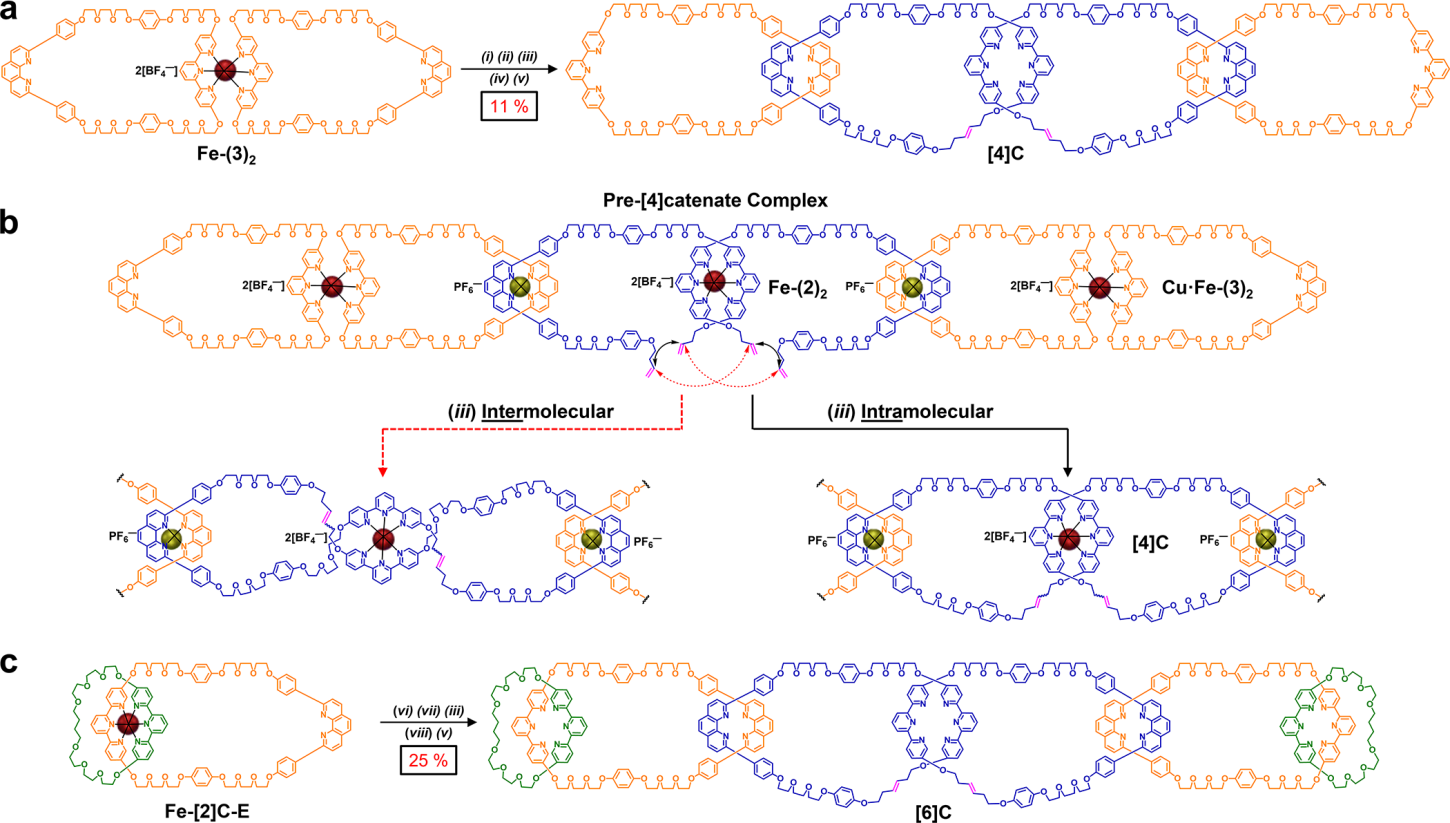
Double “zip-tie” strategies to synthesize even-numbered oligo[*n*]catenanes. (a) (*i*) in MeCN, added to Cu(MeCN)_4_PF_6_ (solid), 25 °C, 30 min; (*ii*) **Fe-(2)**_**2**_, 50% CH_2_Cl_2_/MeCN, 25 °C, 1 day; (*iii*) Grubbs’ second-generation catalyst, CH_2_Cl_2_, 35 °C, 2 day; (*iv*) Cs_2_CO_3_, DMF, 75 °C, 1 day; (*v*) KCN, MeCN/H_2_O, 25 °C, 30 min (b) “Zip-tie” synthesis of **[4]C** under topological control favors intramolecular ring closing and thus the catenane topology. (c) (*vi*) Cu(MeCN)_4_PF_6_ in MeCN, 25 °C, 30 min; (*vii*) **Fe-(2)**_**2**_, MeCN, 25 °C, 2.5 day; (*iii*) Grubbs’ second-generation catalyst, CH_2_Cl_2_, 35 °C, 18 h. (*viii*) K_2_CO_3_, DMF, 75 °C, 1 day; (*v*) KCN, MeCN/H_2_O, 25 °C, 30 min.

After the successful synthesis of **[3]C**, the “zip-tie” strategy was used to evaluate the efficacy of synthesizing a higher order linear [5]catenane **[5]C** (Figure 2b, Scheme S20) by replacing macrocycle **3** with a preformed [2]catenane **[2]C-E** (Schemes S15–S16), the latter of which bears phen and terpy coordination sites. Compound **[2]C-E** was first metalated with FeCl_2_ to occupy the terpy ligands, followed by monometalation of the unobstructed phen with CuI in the same reaction flask. Addition of **1** to this bimetallic intermediate yielded the pre-[5]catenate complex, which was subjected to the same RCM reaction and demetalation conditions as **[3]C**. Purification of the crude material via recycling prep-GPC (Figure 3b, Figure S59) produced **[5]C** in 15% yield (*vide infra*) over the final four steps. The purity of **[3]C** and **[5]C** was confirmed by narrow and unimodal analytical GPC traces (Figure 6c, Figure S51) and by ^1^H NMR (Figures S17–S18, S21–S22). Additionally, MALDI-TOF-MS of **[5]C** (Figure 4a, Figure S73) exhibited a well-defined parent molecular ion [*M* + H]^+^ with *m*/*z* = 5045 Da, as well as macrocyclic and [*n*]catenane fragments that are indicative of the linear [5]catenane topology. A similar fragmentation pattern was observed during MS/MS of **[5]C** (Figure 4b, Figures S67–S68), in which the [*M* + 3H]^3+^ adduct was isolated and fragmented, which produced distinct [3]- and [4]catenane fragments that could only be derived from **[5]C**. All other observable peaks by prep-GPC were isolated and subjected to MS/MS analysis. None showed any ring-closed products. Thus, the tandem MS data confirmed only one ring-closing reaction occurred during the synthesis of **[5]C** instead of the formation of deleterious figure-of-eight intermediates^6,22−25^ which have been reported previously for multiple, concurrent ring-closures (e.g., Figure 5).

Since the “zip-tie” approach with a single central ring combined with preformed macrocycles or [2]catenanes is designed to minimize the number of necessary ring-closing events, and thus reduce the likelihood of generating unwanted byproducts, higher yielding syntheses were anticipated. However, an obvious dichotomy exists between the combined overall yields obtained for catenanes **[5]C** (15%) and **[3]C** (36%), where each yield includes metalation, self-assembly, RCM, and demetalation but excludes the steps required to make the premade macrocycle and [2]catenane end-caps. We speculate the difference in yields for this head-to-head comparison largely stems from the relative stability of the respective precatenate complexes during the RCM step, which in principle should be the same for each mechanical bond-forming reaction due to the commonality of the approach and central macrocycle **1**. This notion is supported by comparing the recycling prep-GPC traces obtained during the purification of **[3]C** versus **[5]C** (Figure 3a,b). For the former, the largest peak outside of the starting macrocycle **3** (which was added in excess) relates to the **[3]C** product, whereas for the latter, there is a substantial peak for the incomplete [3]catenane byproduct that results from ring closing of a partial precatenate complex. This partial instability may also lead to the production of other unwanted kinetic byproducts, such as disperse oligomers, which may further decrease the overall yield for **[5]C**.

Although the single “zip-tie” reaction with **1** was successfully implemented to produce **[3]C** and **[5]C**, it has thus far been limited to the synthesis of lower molecular weight [2*n*+1]catenanes, where *n* is the number of preformed interlocked macrocycles. Inspired by the double ring-closing approach established by Sauvage over three decades ago,^41^ we envisioned a process to achieve higher-order even-numbered linear catenanes by using a preformed [2]catenane building block that can undergo further catenation. We previously reported^25^ the synthesis of the dual-ligand open macrocycle **2** (Figure 1, and Schemes S7–S9) and its iron(II) complex **Fe-(2)**_**2**_ (Figure 5a and Scheme S12). Open macrocycle **2** contains tridentate terpy and bidentate phen ligands, which preferentially bind bivalent and monovalent metals, respectively. Asymmetric short and long olefin-functionalized linkers were also implemented in an attempt to mitigate the intermolecular metathesis reaction that leads to topologically trivial figure-of-eights, while still promoting intramolecular ring-closing to afford the desired [2]catenane product. Despite these efforts, the double-RCM reactions of **Fe-(2)**_**2**_ with the second-generation Grubbs’ catalyst (Figure 5a, Scheme S13) afforded the figure-of-eight product **I** and the [2]catenane product **[2]C** in a nearly 2:1 ratio. Moreover, due to the asymmetric nature of **2**, compound **[2]C** was isolated as a racemic mixture (Figure S1). To simplify the purification process, the mixture of ring-closed products was demetalated with a suspension of Cs_2_CO_3_ in DMF at 75 °C. The metal-free products were separated via recycling prep-GPC (Figure S56). The identity of the isolated fractions was determined by MS/MS experiments (Figure 5b,c, Figures S62–S63), where in each case, the [*M* + 3H]^3+^ adduct was isolated and fragmented until the parent adduct was consumed. The cleavage of any bond in one of the macrocycles that make up **[2]C** yields an intact macrocycle, which is what was observed (Figure 5b, lower spectrum) as the major species in the MS/MS experiment. However, the fragmentation of the larger macrocycle **I** generated a complex mass spectrum with a wide range of fragments (Figure 5c, lower spectrum). These fragmentation patterns are consistent with those observed by Au-Yeung and co-workers^31^ who also differentiated linear oligo[*n*]catenanes from their figure-of-eight counterparts using MS/MS.

The predominate isolation of **I** over **[2]C** led to the hypothesis that the flexible glycol-based linkers in **Fe-(2)**_**2**_ readily allowed for intermolecular ring-closings instead of the desired intramolecular reaction (Figure 5a), ultimately yielding the figure-of-eight product as the major topological intermediate product. We speculated that if the flexible linkers could be “rigidified” (Figure 6),^26,27^ the intermolecular RCM reaction would become less favorable. As an alternative to making chemical modifications of **2** to accomplish this goal, we envisaged complex **Fe-(2)**_**2**_ could be “rigidified” by noncovalently threading on the iron(II)-macrocycle dimer **Fe-(3)**_**2**_ before ring closing to afford a pre-[4]catenate complex (Figure 6a,b), in a manner similar to the synthesis of **[3]C**. Thus, under topological control, the resultant pre-[4]catenate complex (Figure 6b) can spatially arrange the olefin linkers to favor *intra*molecular RCM reactions over unproductive *inter*molecular pathways.

**Figure 7:**
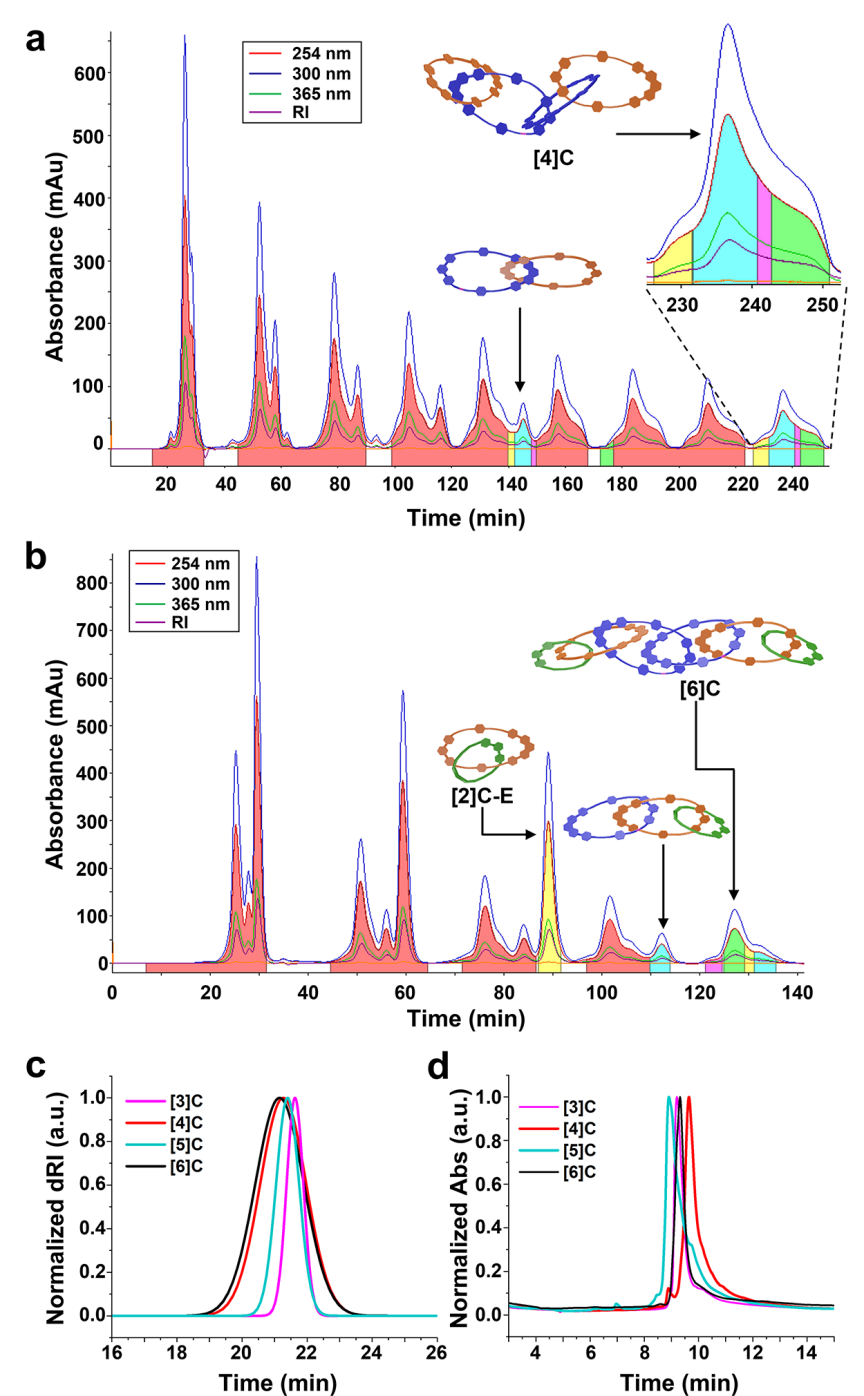
Recycling pre-GPC data for **[4]C** (a) (second injection) and **[6]C** (b) were collected in a DMF mobile phase at 8 mL·min^–1^. Pure **[4]C** was isolated in the blue fraction at 235 min, whereas pure **[6]C** was isolated in the green fraction at 125 min. (c) Overlay of analytical GPC traces (normalized dRI) of **[3]C**, **[4]C**, **[5]C**, and **[6]C** in DMF with 0.025 M LiBr at 60 °C. (d) Overlay of analytical HPLC traces (normalized absorbance at 254 nm) of **[3]C**, **[4]C**, **[5]C**, and **[6]C** with a gradient mobile phase of MeCN/H_2_O (0.1% TFA): 5 to 100% in 10 min and 100% MeCN for 15 min at 40 °C. *Note*: the solubility of the catenane products in the HPLC mobile phase was poor until the end of the gradient, i.e., closer to 95% MeCN. This caused the catenane products to streak on the column. Additionally, see Figures S75–S78 for corresponding LRMS-ESI spectra.

We previously demonstrated (Figure 1a) a similar double “zip-tie” strategy to synthesize a linear [4]catenane after two simultaneous one-pot RCM reactions; however, in that case the smaller, monofunctionalized phen-based macrocycles at each terminus produced lower molecular weight products and prevented further syntheses of higher-order catenanes.^25^ Additionally, the asymmetric nature of the previous end-cap macrocycles led to difficult purification, as well as a mixture of six topologically diastereomeric linear [4]catenanes that gave rise to complicated NMR spectra. In order to alleviate these issues, the double “zip-tie” strategy was adapted using the larger and more symmetric macrocycle **3** as its Fe^2+^ dimer **Fe-(3)**_**2**_ to synthesize the linear [4]catenane **[4]C**, which is composed of four large dual-ligand terpy-phen-based macrocycles (Figure 1, Figure 6a,b, and Scheme S19). With the terpy ligands “rigidified” as octahedral Fe^2+^ complexes, the phen ligands were readily monometalated with Cu(MeCN)_4_PF_6_ to give **Cu·Fe-(3)**_**2**_ as an air-sensitive intermediate, which was stirred under N_2_ atmosphere at 25 °C for 15 min before a solution of **Fe-(2)**_**2**_ was added to form the pre-[4]catenate complex (Figure 6b). Double RCM reactions were carried out next with Grubbs' catalyst, and the conversion to **[4]C** was confirmed by ^1^H NMR. After sequential demetalation steps with Cs_2_CO_3_ and KCN (Figure 6a, Scheme S19), the demetalated mixture was purified via recycling prep-GPC (Figure 7a, Figure S58) to afford **[4]C** in an 11% yield over the final three steps. Once again, the isolated yield to make the larger-ringed **[4]C** was lower presumably because of complex instability during the RCM step. The prep-GPC trace reveals substantial peaks for a [2]catenane byproduct, as well as unidentified higher and lower molecular weight byproducts, which were purified out. All other minor peaks by prep-GPC were isolated and subjected to MS/MS analysis. None showed any ring-closed products. This result is consistent with the aforementioned synthesis to make **[5]C**, except in this instance two RCM steps were required to establish the desired number of mechanical bonds.

**Figure 8:**
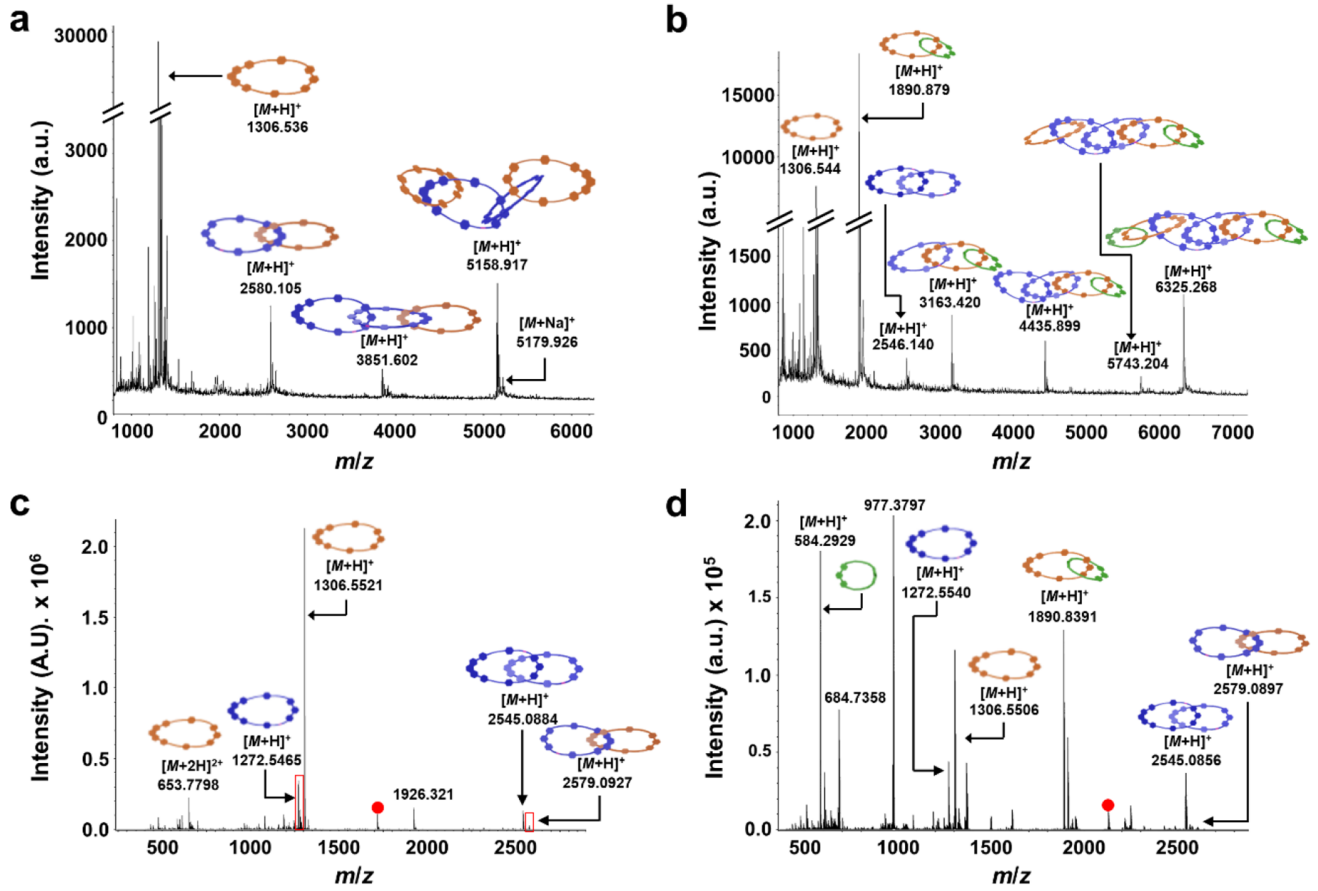
MALDI-TOF-MS of **[4]C** (a) and **[6]C** (b) with α-cyano-4-hydroxycinnamic acid matrix. (c) THRMS-ESI (i.e., MS/MS) of **[4]C**; the [*M* + 3H]^3+^ = 1720.06 Da peak (red dot) was isolated and fragmented. (d) THRMS-ESI of **[6]C**; the [*M* + 3Na]^3+^ = 2130 Da peak (red dot) was isolated and fragmented. Each peak labeled with a red dot represents remaining unfragmented catenane.

The purity of **[4]C** was confirmed by the narrow and unimodal analytical GPC traces (Figure 7c, Figure S51) and HPLC traces (Figure 7d, Figure S61), as well as by ^1^H NMR (Figures S19–S20). The topology of **[4]C** was confirmed using spectrometric methods, whereby MALDI-TOF-MS (Figure 8a, Figure S72) showed a prominent peak for the corresponding parent molecular ion [*M* + H]^+^, as well as the macrocycle and catenane fragments. The asymmetric [2]catenane fragment at *m*/*z* = 2579 Da (Figure 8a) could have only originated from the linear [4]catenane, as opposed to the hypothetical [3]catenane derived from the figure-of-eight topological product. A similar fragmentation pattern was observed during MS/MS of **[4]C** (Figure 8c, Figures S65–S66), in which the [*M* + 3H]^3+^ adduct was isolated and fragmented. In addition to the asymmetric [2]catenane, the ring-closed product of **2** was also observed, both of which can only originate from the pathway to **[4]C**. In contrast with the RCM reaction of complex **Fe-(2)**_**2**_, in which intramolecular and intermolecular pathways occur concurrently to produce a 2:1 ratio of figure-of-eight to catenane products, the double “zip-tie” approach involving two **Cu·Fe-(3)**_**2**_ threaded onto **Fe-(2)**_**2**_ exclusively produced the catenane product **[4]C** as no figure-of-eight impurities were ever isolated.

The complete versatility of the double “zip-tie” approach was demonstrated by substituting the end-cap macrocycles **3** that were used to prepare **[4]C** with preformed [2]catenanes **[2]C-E** to synthesize a linear [6]catenane **[6]C** (Figure 6c, Scheme S21). A slight modification of the synthetic protocol was made between **[5]C** and **[6]C**, namely, the preformed [2]catenane was metalated with Fe^2+^ in a separate step producing [2]catenate **Fe-[2]C-E** (Scheme S17). The open phen-coordination site of **Fe-[2]C-E** was then monometalated with Cu(MeCN)_4_PF_6_, analogous to the synthesis of **[4]C**. A solution of **Fe-(2)**_**2**_ was added to **Cu·Fe-[2]C-E** via syringe, and the dark red solution was allowed to stir under N_2_ atmosphere for 2.5 days to ensure formation of the pre-[6]catenate complex consisting of two **Cu·Fe-[2]C-E** catenates threaded onto complex **Fe-(2)**_**2**_. Next, RCM with Grubbs' catalyst was carried out on the pre-[6]catenate complex in anhydrous CH_2_Cl_2_ under mild heating. The crude ring-closed products were demetalated (Figure 6c, Scheme S21) and purified via recycling prep-GPC (Figure 7b, Figure S60) to afford **[6]C**, which was isolated in a 25% yield over the final three steps. It is important to note that the yield here was higher than that for **[4]C**, which we surmise is due to the [2]catenane end-cap **[2]C-E** being relatively more stable than the iron(II)-macrocycle dimer complex **Fe-(3)**_**2**_ during the course of two RCM reactions. Also, the use of complex **Fe-(2)**_**2**_ as the key “zip-tie” core seems to be more compatible with the end-cap [2]catenane **[2]C-E** versus that when macrocycle **1** was used to synthesize **[5]C** in 15% yield.

The purity of **[6]C** was verified using analytical GPC (Figure 7c, Figure S51) and HPLC (Figure 7d, Figure S61), as well as ^1^H NMR (Figures S23–S24, S26). Due to its higher molecular weight, **[6]C** exhibited a shorter retention time by GPC compared to **[3]C**, **[4]C**, and **[5]C**. MALDI-TOF-MS of **[6]C** (Figure 8b, Figure S74) displayed clear peaks for the parent molecular ion [*M* + H]^+^ and the various [*n*]catenane fragments. The asymmetric [3]catenane fragment at *m*/*z* = 3163 Da is distinctive to the linear [6]catenane topology because a hypothetical [5]catenane derived from the figure-of-eight topological product could not fragment in this manner. Further evidence for the topology of **[6]C** was obtained via MS/MS experiments (Figure 8d, Figures S69–S70), in which the [*M* + 3H]^3+^ adduct was isolated and fragmented. The asymmetric [2]catenane fragment at *m*/*z* = 2579 Da and the ring-closed macrocycle from **2** at *m*/*z* = 1272 Da could only be observed by fragmentation of **[6]C**. The detection of these fragmented species by MALDI-TOF-MS and MS/MS, combined with analytical chromatography methods, provide definitive evidence for the successful synthesis and isolation of the unimolecular, linear [6]catenane. Moreover, all other observable peaks by prep-GPC were isolated and subjected to MS/MS analysis. None showed any ring-closed products. As was observed during the synthesis of **[4]C**, the topological control afforded by the double “zip-tie” method again prevented intermolecular ring-closing and yielded only the expected catenane product **[6]C** and no figure-of-eights (based on characterization of all isolated GPC fractions).

## Conclusion

Two general synthetic blueprints have been developed to make higher-order, linear [2*n*+1]catenanes and [2*n*+2]catenanes under topological control using orthogonal metal templation to generate precatenate complexes, followed by RCM reactions in a single step. In the case of the even-numbered catenane, the so-called molecular “zip-tie” approach eliminated the formation of figure-of-eight topological products during the critical ring-closing steps that lead to mechanical bond formation. These methodologies were applied to synthesize a family of linear oligo[*n*]catenanes, where *n* = 2, 3, 4, 5, or 6, the latter of which representing the highest order unimolecular and linear catenane ever isolated. We offer these synthetic approaches as a way to bridge the gap between the synthesis of lower molecular weight oligo[*n*]catenanes and true linear poly[*n*]catenanes, making their study in materials a realistic proposition. Future endeavors in this research area relate to the synthesis of catenanes with terminal rings that bear polymerizable functional groups,^32^ such that poly[*n*]catenanes can be generated through traditional polymerization methods (e.g., step-growth), in addition to refining the synthetic approach toward true poly[*n*]catenanes consisting exclusively of mechanically bonded molecular rings.

## Methods

The synthesis and spectroscopic characterization of all precursors and catenanes are described in full detail in Schemes S1–S21 and Figures S1–S78.

### Synthesis of Linear [6]catenane [6]C

A solution of end-cap [2]catenate **Fe-[2]C-E** (0.0601 g, 0.0284 mmol, 4.0 equiv) was prepared in 10 mL of N_2_-purged anhydrous MeCN in an oven-dried 50 mL round-bottom (RB) flask. To this was added a solution of Cu(MeCN)_4_PF_6_ (0.0105 g, 0.028 mmol, 4.0 equiv) in 3 mL of N_2_-purged anhydrous MeCN. The solution was stirred at room temperature for 0.5 h under N_2_ atmosphere before the addition of a solution of **Fe-(2)**_**2**_ (0.022 g, 0.0077 mmol, 1.0 equiv) in 7 mL of N_2_-purged anhydrous MeCN via syringe. The dark red solution was stirred under N_2_ atmosphere for 2.5 days. The solvent was removed via rotary evaporator, and the crude was taken up in 100 mL of CH_2_Cl_2_. The organics were washed with 3 × 50 mL of DI H_2_O and dried over Na_2_SO_4_. The dark red solution was filtered, and the filtrate was concentrated via rotary evaporator to afford the pre-[6]catenate complex as a dark red film, which was used without further purification. The crude pre-[6]catenate complex was redissolved in 25 mL of anhydrous CH_2_Cl_2_ in a 100 mL RB flask, and a solution of Grubbs’ second-generation catalyst (0.0013 g, 0.0015 mmol, 0.2 equiv) in 1 mL CH_2_Cl_2_ was added. The flask was fitted with a Vigreux column and was heated to 35 °C while stirring under N_2_ atmosphere. After 18 h, an aliquot was taken and quenched with ethyl vinyl ether (EVE). The reaction was deemed complete by ^1^H NMR, and the remaining reaction mixture was quenched with 1 mL of EVE and 5 mL of MeCN. The solvent was then removed via rotary evaporator to afford the crude [6]catenate mixture as a dark red film. In order to remove noninterlocked structures and simplify the purification process, the Fe^2+^ and Cu^+^ templates were removed. The Fe^2+^ ion was removed from the crude mixture with addition of a weak inorganic base and moderate heating, while the Cu^+^ ion was removed in a second step by the addition of a strongly competing ligand. The crude film was redissolved in 25 mL of DMF in a 100 mL RB flask. Solid K_2_CO_3_ (1.0 g, 7.23 mmol, 1000 equiv) was added, and the suspension was heated to 75 °C for 1 day while stirring open to air. The solvent was removed via rotary evaporator, and 50 mL of MeCN was added to the reaction mixture. A solution of KCN (0.2 g, 3.07 mmol, 400 equiv) in 10 mL of H_2_O was added via syringe. The suspension was stirred open to air at room temperature for 0.5 h. The suspension was diluted with 300 mL of CH_2_Cl_2_. The organics were washed with 3 × 100 mL DI H_2_O, dried over Na_2_SO_4_, and filtered. The solvent was removed via rotary evaporator, and the crude was redissolved in 4 mL of GPC grade DMF. The solution was filtered via syringe filter and was purified via recycling preparative GPC with DMF mobile phase. The mixed fractions were also collected and repurified. The linear [6]catenane **[6]C** was isolated as a yellow/orange film (0.0122 g, 25% based on **Fe-(2)**_**2**_).

### Gel Permeation Chromatography (GPC)

Recycling preparative gel permeation chromatography (prep-GPC) was performed on a Japan Analytical Industry LaboAce instrument with one JAIGEL-2HR column and one JIAGEL-2.5HR column in sequence, running with DMF at 8 mL·min^–1^ as the mobile phase. Analytical GPC analyses were performed on an Agilent 1260 Infinity setup with two Shodex GPC KD-8060 columns in sequence, running with DMF (0.025 M LiBr) at 1 mL·min^–1^ as the mobile phase. The differential refractive index (dRI) of each compound was monitored using a Wyatt Optilab T-rEX detector.

### High-Pressure Liquid Chromatography (HPLC)

Analytical HPLC analyses were performed on an Avant 2000 HPLC with a Shodex Asahipak ODP-50-2D reverse-phase column with a gradient mobile phase of H_2_O with 0.1% trifluoracetic acid (TFA) and MeCN with 0.1% TFA running at 0.2 mL·min^–1^, which was in series with an Advion Expression-L Compact Mass Spectrometer. UV–vis absorbance was recorded at 254 nm.

### Tandem High-Resolution Mass Spectrometry Electrospray Ionization (THRMS-ESI)

THRMS-ESI mass spectra were recorded on a Bruker maXis 4G Q-TOF mass spectrometer.

### MALDI-Time-of-Flight (MALDI-TOF)

MALDI-TOF mass spectra were recorded on a Bruker Solaris 12T FT-MS; samples were prepared using 2,5-dihydroxybenzoic or α-cyano-4-hydroxycinnamic acid matrices.

## Supporting Information Available

The Supporting Information is available free of charge on the ACS Publications Web site as a single PDF. The Supporting Information is available free of charge at https://pubs.acs.org/doi/10.1021/acscentsci.2c00697.

Synthesis and characterization data (i.e., nuclear magnetic resonance (NMR), mass spectrometry (MS), gel permeation chromatography (GPC), and liquid chromatography (LC), etc.) (PDF)
