# Supplementary material for: Topologically Controlled Syntheses of Unimolecular Oligo[n]catenanes
Source: ACS Cent Sci. 2022 Nov 29;8(12):1672–82. doi: 10.1021/acscentsci.2c00697 (PMC9801505; doi:10.1021/acscentsci.2c00697)
Supplement: Supplementary file 1 — oc2c00697_si_001.pdf [file oc2c00697_si_001.pdf]

Supporting Information

Topologically Controlled Syntheses of Unimolecular Oligo[*n*]catenanes

Nathan D. Colley, Mark A. Nosiglia, Sheila L. Tran, Gray H. Harlan, Christy Chang, Ruihan Li, Abigail O. Delawder, Yipei Zhang, Jonathan C. Barnes*

Department of Chemistry, Washington University, St. Louis, MO 63130, USA

*E-mail: jcbarnes@wustl.edu

Table of Contents

Section A. Materials / General Methods / Instrumentation* --------------------------------------- *S3

Section B. Synthetic Protocols* --- *S4

1) ***Synthesis of Open and Closed Macrocyclic Ligands* -------------------------------- *S4***
   a) *Mono-Allyl Diethylene Glycol Mesyl (**A**)* -- S4
   b) *Phen Mono Allyl Diethylene Glycol (**B**)* --- S6
   c) *Open Phen-Phen Macrocycle (**1**)* -- S7
   d) *Mono-Allyl Triethylene Glycol (**C**)* -- S8
   e) *Mono-Allyl Triethylene Glycol Mesyl (**D**)* -- S9
   f) *Di-Allyl Triethylene Glycol Terpy (**E**)*--- S10
   g) *Terpy HQ Phenol (**F**)* --- S11
   h) *Phen HQ Olefin (**G**)* -- S12
   i) *Open Terpy-Phen Macrocycle (**2**)* -- S13
   j) *Terpy HQ Triethylene Glycol Mesyl (**H**)* --------------------------------------- S14
   k) *Symmetric Terpy-Phen Macrocycle (**3**)* --- S16
2) ***Synthesis of Linear Catenanes* --- *S18***
   a) *Fe^{2+}-Open Terpy-Phen Macrocycle Dimer (**Fe-(2)$_2$**)* -------------------------- S18
   b) *[2]Catenane-TPM (**[2]C**) and Figure-Eight TPM (**I**)* ---------------------------- S19
   c) *Fe^{2+}-Symmetric Terpy-Phen Macrocycle (**Fe-(3)$_2$**)* ----------------------------- S22
   d) *[2]Catenane-Endcap Unsaturated (**[2]C-U**)* -------------------------------------- S23
   e) *[2]Catenane-Endcap (**[2]C-E**)* -- S26
   f) *Fe^{2+}-[2]C-Endcap (**Fe-[2]C-E**)* -- S28
   g) *[3]Catenane (**[3]C**)* -- S30
   h) *[4]Catenane (**[4]C**)* -- S32
   i) *[5]Catenane (**[5]C**)* -- S35
   j) *[6]Catenane (**[6]C**)* -- S37

Section C. Spectroscopic Characterization*-- *S40

1) ***^{1}H NMR Spectra*** -- ***S40***
2) ***2D ^{1}H-^{1}H NMR Spectra*** -- ***S63***
3) ***^{13}C NMR Spectra*** --- ***S67***
4) ***UV-Vis Spectra of [2]C, [4]C, and [6]C* --- *S76***
5) ***Gel Permeation Chromatography (GPC)* -- *S77***
6) ***High-Pressure Liquid Chromatography (HPLC)* -------------------------------------- *S83***

Section D. Spectrometric Characterization* --- *S84

1) ***High-res Mass Spectrometry Electrospray Ionization (HRMS-ESI) and Tandem High-res Mass Spectrometry Electrospray Ionization (THRMS-ES)---S84***
2) ***Matrix-Assisted Laser Desorption/Ionization Time-of-Flight (MALDI-TOF) --- S93***
3) ***Low-res Mass Spectrometry Electrospray Ionization (LRMS-ESI) --------------- S97***

Section E. References* -- *S99

Section A. Materials / General Methods / Instrumentation

All reagents were purchased from commercial suppliers and used without further purification unless stated otherwise. Metal sources $Fe(BF_4)_2$•6 H_2O, $Cu(MeCN)_4PF_6$, and CuI were purchased from Sigma-Aldrich. $FeCl_2$ was purchased from Fisher Scientific. Modified literature procedures were employed in the synthesis of compounds **F**, **G**, **2**, and **Fe-(2)₂**.[1] All reactions were performed under N_2 using common Schlenk techniques. Column chromatography was carried with silica gel (Sorbtech, 0.040 – 0.063 mm) or neutral alumina (Sorbtech, Act. 1, 0.050 – 0.2 mm) or basic alumina (Sorbtech, Act. 1, 0.050 – 0.2 mm). All ring-closing reactions were done using Grubbs' 2nd generation catalyst (Chem Scene). Recycling preparative gel permeation chromatography (prep-GPC) was performed on a Japan Analytical Industry LaboACE instrument with one GEL-2HR column and one JAIGEL-2.5HR column in sequence, running with dimethylformamide (DMF) at 8 mL·min^{-1} as the mobile phase. All nuclear magnetic resonance (NMR) spectra were recorded on Varian Inova-500 spectrometer at 25 °C, with working frequencies of 500 (^{1}H) and 125 (^{13}C) MHz. Chemical shifts are reported in ppm relative to the signals corresponding to the residual non-deuterated solvents: $CDCl_3$: δ_H = 7.26 ppm and δ_C = 77.16 ppm. Ultraviolet-Visible (UV-Vis) absorbance spectra were recorded on an Agilent Cary 5000 spectrophotometer with a quartz cuvette (1.0 cm pathlength). Analytical GPC analyses were performed on an Agilent 1260 Infinity setup with two Shodex GPC KD-806M columns in sequence in DMF mobile phase (0.025 M LiBr) running at 60 °C at 1.0 mL·min^{-1}. The differential refractive index (dRI) of each compound was monitored using a Wyatt Optilab T-rEX detector. Analytical HPLC analyses were performed on an Avant 2000 HPLC with a Shodex Asahipak ODP-50-2D reverse-phase column with a gradient mobile phase of H_2O with 0.1% TFA and MeCN with 0.1% TFA running at 40 °C at 0.2 mL·min^{-1}, which was in series with an Advion Expression-L Compact Mass Spectrometer; UV-vis absorbance was recorded at 254 nm. Low-resolution mass spectrometry electrospray ionization (LRMS-ESI) was recorded on an Advion Expression-L Compact Mass Spectrometer. High-

resolution mass spectrometry electrospray ionization (HRMS-ESI) was recorded on a Waters Synapt G2 HDMS or a Bruker maXis 4G UHR-TOF mass spectrometer. Tandem high-resolution mass spectrometry electrospray ionization (THRMS-ESI) was recorded on a Bruker maXis 4G Q-TOF mass spectrometer. Matrix-assisted laser desorption/ionization time-of-flight mass spectrometry (MALDI-TOF-MS) was recorded on a Bruker Solaris 12T FT-MS; samples were prepared using 2,5-dihydroxybenzoic or α-cyano-4-hydroxycinnamic acid matrices.

Section B. Synthetic Protocols

1) Synthesis of Open and Closed Macrocyclic Ligands

*a) Mono-Allyl Diethylene Glycol Mesyl (**A**)*

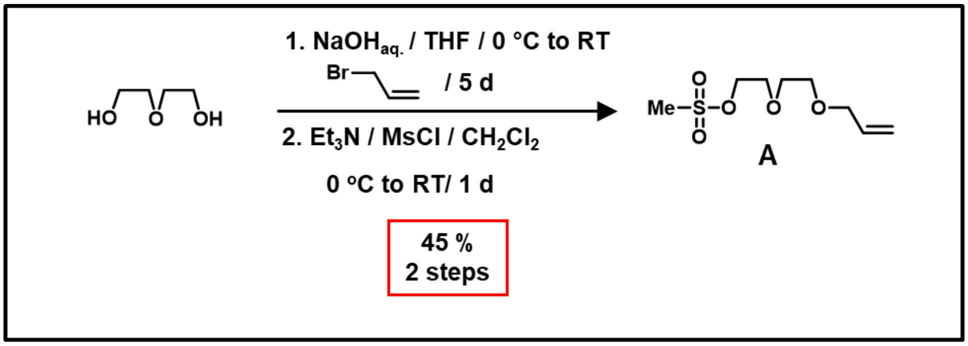

Scheme S1. Synthesis of compound **A**.

<u>Step 1:</u> A solution of diethylene glycol (25.00 g, 22.3 mL, 235.6 mmol, 1.0 eq.) was prepared in 300 mL tetrahydrofuran (THF) in a 1 L round-bottom (RB) flask with a stir bar. While stirring, a solution of NaOH (6.59 g, 164.9 mmol, 0.7 eq.) in 25 mL DI H_2O was added. The solution was cooled using an ice bath for 1 h before adding a solution of allyl bromide (34.2 g, 24.4 mL, 282.7 mmol, 1.2 eq.) in 200 mL THF via slow addition funnel over the course of several hours. The reaction was allowed to warm up to room temperature while stirring open to air for 5 d. The solvent was then removed via rotary evaporator. The crude oil was taken up in 1L brine and 500 mL dichloromethane (CH_2Cl_2). The aqueous layer was further extracted with 4 x 500 mL CH_2Cl_2. The

organics were dried over Na_2SO_4 and filtered. The solvent was removed via rotary evaporator to afford the crude asymmetric product as an orange oil, which was taken forward to mesylation without further purification.

Step 2: The crude orange oil was redissolved in 500 mL CH_2Cl_2 in a 1 L RB flask with a stir bar. The reaction was cooled on an ice bath and neat mesyl chloride (MsCl) (80.9 g, 54.7 mL, 706.8 mmol, 3.0 eq.) was added slowly via syringe. Next, neat triethylamine (Et_3N) (87.1 g, 120 mL, 860.9 mmol, 3.7 eq.) was added slowly via syringe. The reaction was allowed to warm up to room temperature while stirring for 1 d. The reaction mixture was diluted with 700 mL CH_2Cl_2 and was washed with 4 x 350 mL 1 M HCl and 2 x 350 mL aqueous saturated $NaHCO_3$. The organics were dried over Na_2SO_4 and were filtered. The solvent was removed via rotary evaporator. The crude oil was purified via silica chromatography to yield the product as an orange oil (23.9 g, 45% over two steps). ^{1}H NMR (500 MHz, $CDCl_3$): δ_H 5.97 – 5.83 (m, 1H), 5.32 – 5.14 (m, 2H), 4.41 – 4.34 (m, 2H), 4.05 – 3.98 (m, 2H), 3.80 – 3.75 (m, 2H), 3.72 – 3.66 (m, 2H), 3.64 – 3.58 (m, 2H), 3.06 (s, 3H). ^{13}C NMR (125 MHz, $CDCl_3$): δ_C 134.5, 117.2, 72.2, 70.7, 69.32, 69.27, 69.0, 37.7.

*b) Phen Mono Allyl Diethylene Glycol (**B**)*

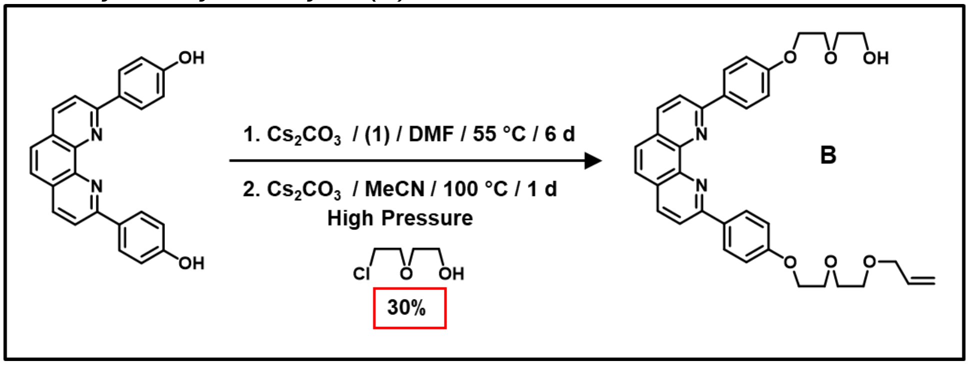

Scheme S2. Synthesis of compound **B**.

<u>Step 1:</u> A suspension of phenanthroline (phen) diol[1] and Cs_2CO_3 was prepared in 200 mL DMF in a 500 mL RB flask. The suspension was heated to 55 °C for 1 h while stirring under N_2 before adding a solution of **A** (0.990 g, 4.41 mmol, 1 eq.) in 25 mL DMF via syringe pump at 3 $mL \cdot h^{-1}$. After heating for a total of 6 d, the solvent was removed via rotary evaporator. The crude was taken up in 300 mL 10% MeOH / CH_2Cl_2. The suspension was filtered via vacuum filtration over a 150 mL fritted funnel with medium porosity. The remaining solid was washed with 200 mL 10% MeOH / CH_2Cl_2. The filtrate was concentrated via rotary evaporator and was again taken up in 200 mL 10% MeOH / CH_2Cl_2. The resultant solid was again collected via vacuum filtration and was kept to later repurify the phen diol starting material. The filtrate was concentrated into a 100 mL high pressure vessel using a rotary evaporator. The sticky orange crude solid was dried on high vacuum within the high-pressure vessel for 1 h before proceeding to the next step.

<u>Step 2:</u> The 100 mL high pressure vessel containing the crude material was charged with 40 mL MeCN and a stir bar. While stirring, solid Cs_2CO_3 (7.2 g, 22.1 mmol, 5 eq.) was added, followed by neat 2-(2-chloro-ethoxy)ethanol (4.0 g, 30.9 mmol, 7 eq.). The vessel was sealed and heated to 100 °C for 18 h. The reaction was cooled to RT and was diluted with 300 mL CH_2Cl_2. The insoluble solids were removed via gravity filtration and the filtrated was washed with 3 x 100 mL brine. The aqueous layer was back-extracted with 2 x 100 mL CH_2Cl_2. The combined organics were dried over Na_2SO_4 and were filtered. The solvent was removed via rotary evaporator and the crude was purified via silica chromatography with a slow gradient (CH_2Cl_2 to 4% MeOH /

CH_2Cl_2) to afford the product as an orange solid (0.77 g, 30%). ^{1}H NMR (500 MHz, $CDCl_3$): δ_H 8.46 – 8.40 (m, 4H), 8.28 (d, J = 8.4 Hz, 2H), 8.10 (d, J = 8.4 Hz, 2H), 7.75 (s, 2H), 7.17 – 7.10 (m, 4H), 5.99 – 5.89 (m, 1H), 5.34 – 5.17 (m, 2H), 4.30 – 4.25 (m, 4H), 4.06 (dt, J = 5.7, 1.3 Hz, 2H), 3.97 – 3.92 (m, 4H), 3.83 – 3.77 (m, 4H), 3.75 – 3.71 (m, 2H), 3.70 – 3.65 (m, 2H). ^{13}C NMR (125 MHz, $CDCl_3$): δ_C 160.20, 160.03, 156.35, 156.26, 146.05, 136.86, 134.81, 132.53, 132.35, 129.07, 129.02, 127.62, 127.60, 125.72, 125.68, 119.39, 117.29, 114.95, 114.92, 72.73, 72.39, 71.01, 69.87, 69.73, 69.57, 67.61, 67.56, 61.87. LR-ESI: calculated for $C_{35}H_{36}N_2O_6$: m/z = 581.3 $[M + H]^+$; Found: 581.3 $[M + H]^+$.

*c) Open Phen-Phen Macrocycle (**1**)*

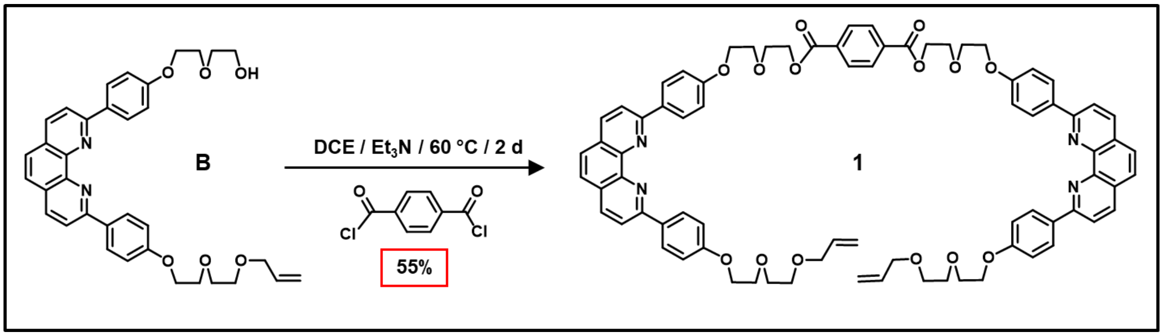

Scheme S3. Synthesis of compound **1**.

Compound **1** was prepared using a similar procedure that was reported by Mayer and co-workers[2] to synthesize an isomer of **1**. A solution of **B** (0.82 g, 1.42 mmol, 3.0 eq.) and Et_3N (1.9 g, 2.6 mL, 19.0 mmol, 40 eq.) was prepared in 30 mL anhydrous 1,2-dichloroethane ($C_2H_4Cl_2$) in a 38 mL high pressure vessel (Kemtech) with a stir bar. Solid terephthaloyl chloride (0.10 g, 0.49 mmol, 1.0 eq.) was add to the solution and the vessel was sealed with a Teflon screw cap. The reaction was heated to 60 °C for 2 d while stirring. The reaction was allowed to cool to room temperature, was diluted with 300 mL CH_2Cl_2 and was washed with 3 x 100 mL brine. The organics were dried over Na_2SO_4 and were filtered. The solvent was removed via rotary evaporator to afford the crude as a sticky orange oil. Silica column chromatography (CH_2Cl_2 to 2% MeOH / CH_2Cl_2) of the crude material afforded the product as an orange solid (0.35 g, 55%). ^{1}H NMR (500 MHz, $CDCl_3$): δ_H

8.46 – 8.37 (m, 8H), 8.25 (dd, J = 8.4, 3.2 Hz, 4H), 8.11 (s, 4H), 8.09 – 8.04 (m, 4H), 7.73 (s, 4H), 7.11 (dd, J = 11.2, 8.8 Hz, 8H), 6.01 – 5.87 (m, 2H), 5.34 – 5.15 (m, 4H), 4.56 – 4.47 (m, 4H), 4.29 – 4.19 (m, J = 10.9, 5.8 Hz, 8H), 4.11 – 4.03 (m, 4H), 3.98 – 3.88 (m, 12H), 3.82 – 3.74 (m, 4H), 3.71 – 3.62 (m, 4H). ^{13}C NMR (125 MHz, $CDCl_3$): δ_C 165.7, 160.1, 160.0, 156.2, 145.9, 136.8, 134.7, 133.9, 132.3, 132.2, 129.6, 128.9, 127.5, 125.6, 119.3, 117.2, 114.9, 114.8, 72.2, 70.9, 69.7, 69.4, 69.2, 67.5, 64.4. MALDI calculated for $C_{78}H_{74}N_4O_{14}$: *m/z* = 1291.5 [*M* + H]$^+$, 1314.5 [*M* + Na]$^+$; Found: 1292.0 [*M* + H]$^+$, 1314.1 [*M* + Na]$^+$ (2,5-dihydroxybenzoic acid matrix).

*d) Mono-Allyl Triethylene Glycol (**C**)*

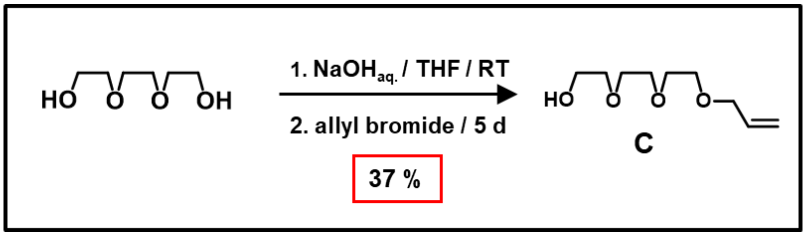

Scheme S4. Synthesis of compound **C**.

A solution of triethylene glycol (25.00 g, 150.2 mmol, 1.0 eq.) was prepared in 300 mL THF in a 1 L round-bottom (RB) flask with a stir bar. While stirring, a solution of NaOH (4.66 g, 116.5 mmol, 0.7 eq.) in 25 mL DI H_2O was added. The solution was cooled using an ice bath before adding a solution of allyl bromide (24.2 g, 17.3 mL, 199.8 mmol, 1.2 eq.) in 200 mL THF via slow addition funnel over the course of several hours. The reaction was allowed to warm up to room temperature while stirring open to air for 5 d. The solvent was then removed via rotary evaporator and the crude yellow sludge was taken up in 500 mL CH_2Cl_2. The organic layer was washed with 2 x 200 mL DI H_2O and 2 x 200 mL brine. The organics were dried over Na_2SO_4 and filtered. The solvent was removed via rotary evaporator to afford the product as a light yellow oil (10.5 g, 37%). ^{1}H NMR (500 MHz, $CDCl_3$): δ_H 5.97 – 5.87 (m, 1H), 5.31 – 5.15 (m, 2H), 4.03 (d, *J* = 5.7 Hz, 2H), 3.75 – 3.71 (m, 2H), 3.71 – 3.65 (m, 6H), 3.64 – 3.59 (m, 4H). ^{13}C NMR (125 MHz, $CDCl_3$): δ_C 134.7, 117.1, 72.5, 72.3, 70.7, 70.6, 70.4, 69.4, 61.7.

*e) Mono-Allyl Triethylene Glycol Mesyl (**D**)*

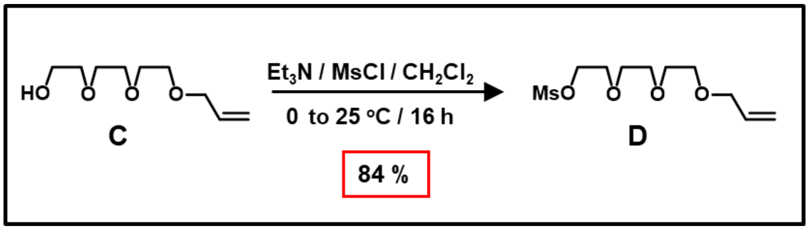

Scheme S5. Synthesis of compound **D**.

A solution of **C** (10.47 g, 55.1 mmol, 1.0 eq.) and Et_3N (29.2 g, 40.0 mL, 288.6 mmol, 5.2 eq.) was prepared in 500 mL CH_2Cl_2 in a 2 L RB flask fitted with a slow addition funnel and containing a stir bar. The solution was cooled using an ice bath before adding a solution of mesyl chloride (MsCl) (19.2 g, 13.0 mL, 167.9 mmol, 3.0 eq.) in 300 mL CH_2Cl_2 via slow addition funnel over the course of 2 h. The reaction was allowed to warm up to room temperature while stirring overnight. After 16 h, the crude was washed with 5 x 200 mL 1M HCl. The organics were dried over Na_2SO_4 and filtered. The solvent was removed via rotary evaporator to afford the crude as an orange oil. Silica column chromatography (CH_2Cl_2 to 3% MeOH / CH_2Cl_2) of the crude material afforded the product as a yellow oil (12.4 g, 84%). ^1H NMR (500 MHz, $CDCl_3$): δ_H 5.87 – 5.78 (m, 1H), 5.23 – 5.07 (m, 2H), 4.32 – 4.27 (m, 2H), 3.93 (dt, *J* = 5.6, 1.4 Hz, 2H), 3.73 – 3.67 (m, 2H), 3.63 – 3.49 (m, 8H), 3.00 (s, 3H). ^{13}C NMR (125 MHz, $CDCl_3$): δ_C 134.6, 116.8, 71.9, 70.39, 70.37, 70.32, 69.3, 69.2, 68.8, 37.5. LR-ESI: calculated for $C_{10}H_{20}O_6S$: *m/z* = 291.1 [*M* + Na]$^+$; Found: 291.2 [*M* + Na]$^+$.

*f) Di-Allyl Triethylene Glycol Terpy (**E**)*

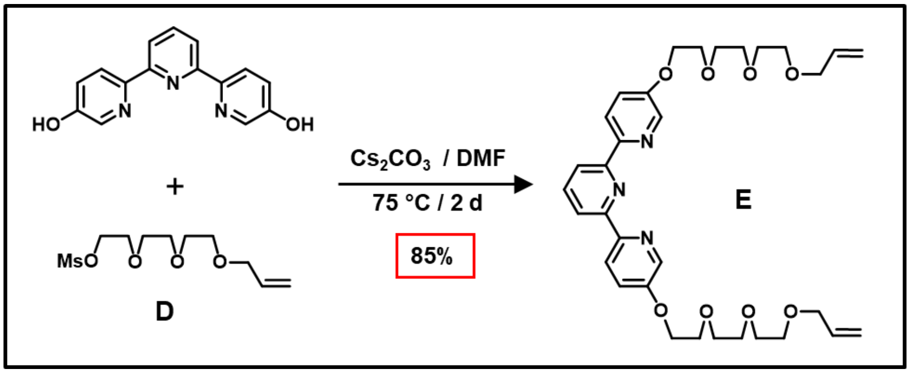

Scheme S6. Synthesis of compound **E**.

A suspension of terpyridine (terpy) diol[1] (1.50 g, 5.65 mmol, 1.0 eq.) and Cs_2CO_3 (9.2 g, 28.3 mmol, 5 eq.) was prepared in 200 mL DMF in a 1L RB flask with a stir bar. While stirring under N_2, the suspension was heated to 75 °C before adding a solution of **D** (4.6 g, 16.95 mmol, 3 eq.) in 300 mL DMF via slow addition funnel over the course of 1 h. The reaction temperature was kept at 75 °C while stirring under N_2 for 2 d. The reaction mixture was allowed to cool to room temperature and was filtered via gravity filtration to remove insoluble inorganic salts. The filtrate was concentrated via rotary evaporator. The crude was resuspended in 300 mL CH_2Cl_2 and was washed with 3 x 100 mL brine. The organics were dried over Na_2SO_4 and filtered. The solvent was removed via rotary evaporator to afford the crude as a yellow oil. Basic alumina column chromatography (50% hexanes / CH_2Cl_2 to 1% MeOH / CH_2Cl_2) of the crude material afforded the product as a bright yellow oil (2.9 g, 85%). ^{1}H NMR (500 MHz, $CDCl_3$): δ_H 8.54 (d, J = 8.8 Hz, 2H), 8.40 (d, J = 2.8 Hz, 2H), 8.30 (d, J = 7.8 Hz, 2H), 7.88 (t, J = 7.8 Hz, 1H), 7.38 (dd, J = 8.8, 2.9 Hz, 2H), 5.96 – 5.87 (m, 2H), 5.30 – 5.14 (m, 5H), 4.29 – 4.23 (m, 4H), 4.02 (dt, J = 5.7, 1.3 Hz, 4H), 3.95 – 3.90 (m, 4H), 3.76 (dd, J = 4.6, 2.0 Hz, 4H), 3.73 – 3.66 (m, 8H), 3.61 (dd, J = 6.0, 3.6 Hz, 4H). ^{13}C NMR (125 MHz, $CDCl_3$): δ_C 155.5, 155.1, 149.5, 137.8, 137.2, 134.9, 122.0, 121.8, 119.7, 117.20, 72.4, 71.1, 70.8, 69.8, 69.6, 68.1. MALDI-TOF: calculated for $C_{33}H_{43}N_3O_8$: m/z = 610.3 $[M + H]^+$; Found: 610.5 $[M + H]^+$ (2,5-dihydroxybenzoic acid matrix).

*g) Terpy HQ Phenol (**F**)*

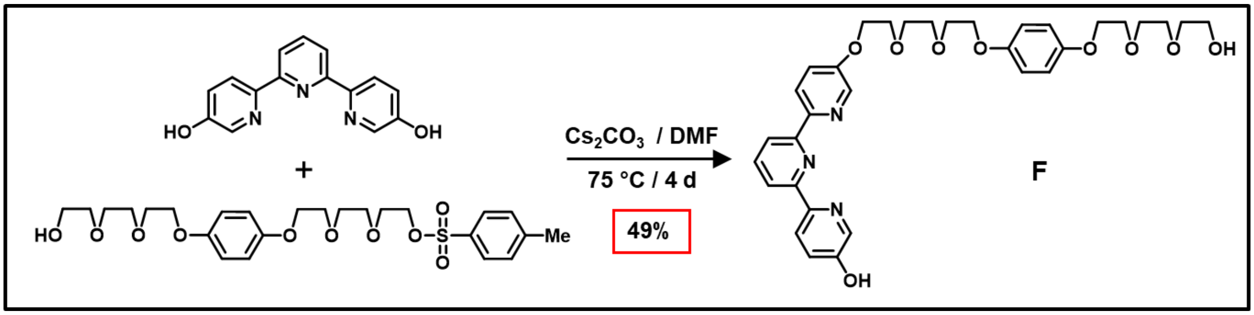

Scheme S7. Synthesis of compound **F**.

We have previously reported[1] the synthesis of compound **F**. Herein, we report an improved synthetic method; terpy diol and mono tosylated bis(triethylene glycol) hydroquinone were prepared according to our previous work.[1] A suspension of terpy diol (1.51 g, 5.68 mmol, 3.0 eq.) and Cs_2CO_3 (9.3 g, 28.4 mmol, 15.0 eq.) was prepared in 250 mL DMF in a 500 mL RB flask. The suspension was heated to 100 °C while stirring under N_2 for 1h. The reaction mixture was then cooled to 75 °C before adding a solution of mono tosylated bis(triethylene glycol) hydroquinone (1.00 g, 1.91 mmol, 1.0 eq.) in 150 mL DMF via slow addition over several hours. The reaction temperature was kept at 75 °C while stirring under N_2 for a total of 4 d. The reaction mixture was then allowed to cool to room temperature and the suspension was filtered via gravity filtration to remove solid inorganic salts. The solvent was then removed via rotary evaporator to afford the crude as a black oily mixture. To the crude was added 300 mL 1M CH_3COOH. The suspension was sonicated and then stirred at room temperature for 1 day before dilution with 300 mL CH_2Cl_2. The aqueous layer was then extracted with 3 x 100 mL CH_2Cl_2. The organics were dried over Na_2SO_4 and were filtered. The solvent was then removed via rotary evaporator to afford the crude product as a brown oil. The crude oil was redissolved in 18 mL GPC grade DMF and was purified via recycling prep-GPC with DMF over five injections to afford the asymmetric product as a brown oil (0.57 g, 49%). ^1H NMR is consistent with our previous work.

*h) Phen HQ Olefin (**G**)*

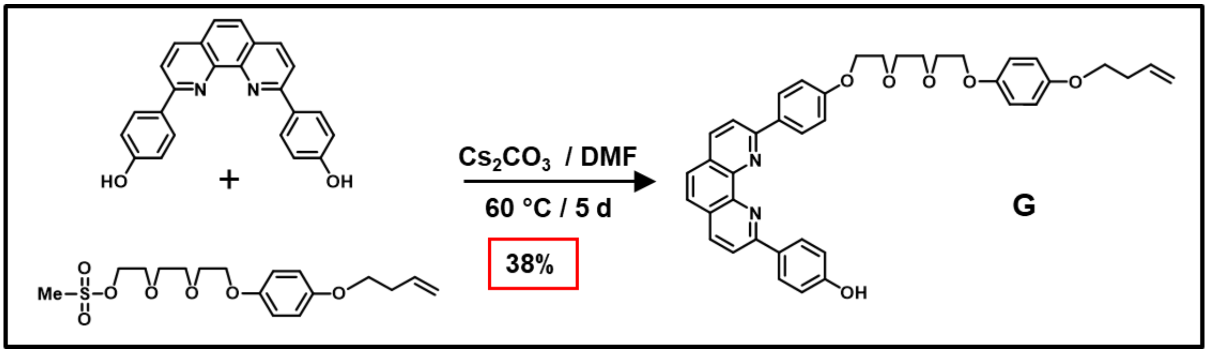

Scheme S8. Synthesis of compound **G**.

We have previously reported[1] the synthesis of compound **G**. Herein, we report an improved synthetic protocol; phen diol and mesylated triethylene glycol hydroquinone olefin were prepared according to our previous work.[1] A suspension of phen diol (3.30 g, 9.05 mmol, 2.5 eq.) and Cs_2CO_3 (1.73 g, 5.30 mmol, 1.5 eq.) was prepared in 250 mL DMF in a 500 mL RB flask and was heated to 60 °C while stirring under N_2. A solution of mono mesylated triethylene glycol hydroquinone olefin (1.33 g, 3.56 mmol, 1.0 eq.) in 50 mL anhydrous DMF was added via syringe pump at 3 mL·h^{-1}. After heating for a total of 5 d, the reaction solvent was removed via rotary evaporator. To the crude was added 400 mL 10% MeOH / CH_2Cl_2. The suspension was stirred at room temperature overnight. The suspension was then filtered via vacuum filtration over a 150 mL fritted funnel with medium porosity. The filtrate was concentrated via rotary evaporator. The crude sludge was taken up in 300 mL CH_2Cl_2, which caused unreacted phen diol starting material to precipitate as an orange solid. The starting material was collected via vacuum filtration. The filtrate was concentrated and was purified via silica column chromatography with a very slow gradient (CH_2Cl_2 to 2 % MeOH / CH_2Cl_2). The product was recovered as a sticky orange solid (0.87 g, 38%). ^{1}H NMR is consistent with our previous work.

*i) Open Terpy-Phen Macrocycle (**2**)*

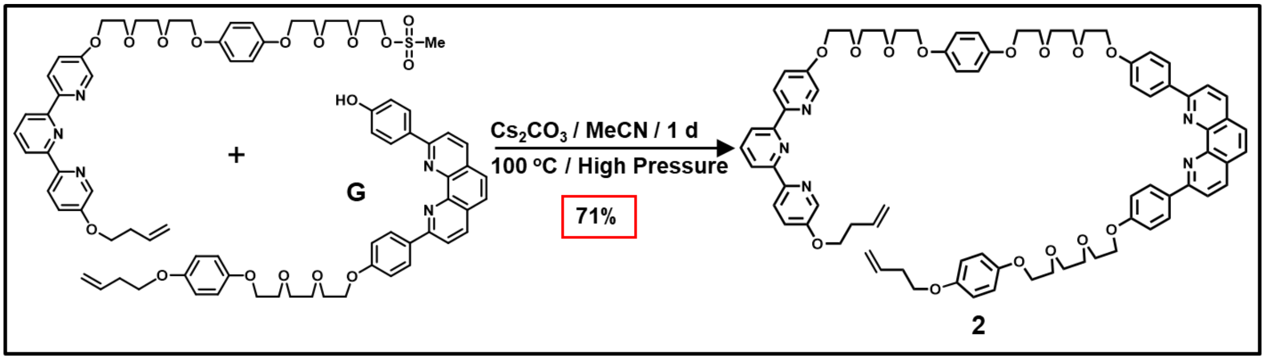

Scheme S9. Synthesis of compound **2**.

We have previously reported[1] the synthesis of **2.** Herein, we report an improved and scaled synthetic protocol; mesylated terpyr olefin was prepared according to our previous work.[1]

Compound **G** (0.55 g, 0.86 mmol, 1.2 eq.) and mesylated terpy olefin (0.54 g, 0.715 mmol, 1.0 eq.) was transferred to a 100 mL high-pressure flask (Kemtech) using a minimal amount of CH_2Cl_2. The solvent was removed via rotary evaporator and the resulting foam was dried on high vacuum for 2 h. Solid Cs_2CO_3 (1.16 g, 3.56 mmol, 5.0 eq.) was added to the vessel, followed by the addition of 40 mL anhydrous MeCN and a stir bar. The vessel was sealed with a Teflon screw cap and was heated to 100 °C for 1 d. After 1 d, the reaction was allowed to cool to room temperature. The crude suspension was diluted with 400 mL CH_2Cl_2 and was washed with 3 x 100 mL brine. The organics were dried over Na_2SO_4 and were filtered. The solvent was removed via rotary evaporator to afford crude product as an orange film. Basic alumina column chromatography (CH_2Cl_2 to 3% MeOH / CH_2Cl_2) of the crude material afforded the product as a sticky orange solid (0.66 g, 71%). ^{1}H NMR is consistent with our previous work.

*j) Terpy HQ Triethylene Glycol Mesyl (**H**)*

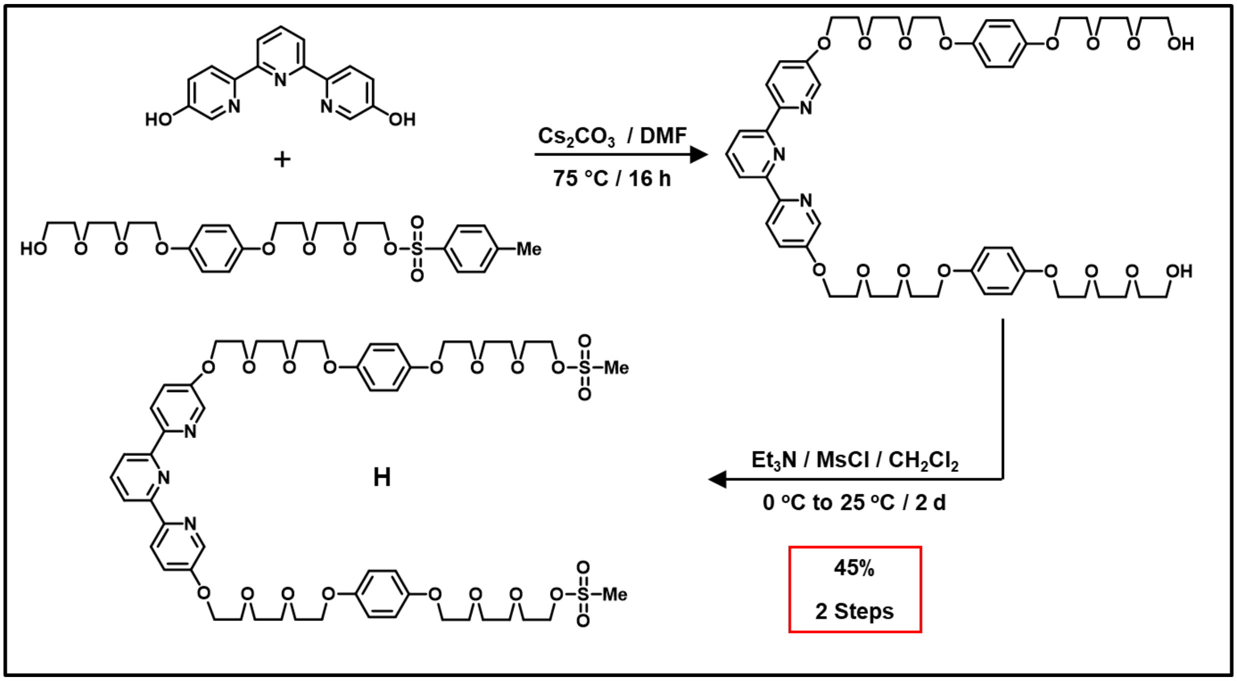

Scheme S10. Synthesis of compound **H**.

Step 1: A suspension of terpy diol[1] (0.50 g, 1.87 mmol, 1.0 eq.) and Cs_2CO_3 (3.71 g, 11.38 mmol, 6.0 eq.) was prepared in 400 mL DMF in a 1 L RB flask fitted with a slow addition funnel. A solution of mono tosylated bis(triethylene glycol) hydroquinone (2.98 g, 5.65 mmol, 3.0 eq.) in 200 mL DMF was added via slow addition over 2 h. The reaction was heated to 75 °C while stirring under N_2. After 16 h, the solvent was removed by rotary evaporator and the crude product was taken up in 300 mL CH_2Cl_2. The organics were washed with 3 x 100 mL brine, dried over Na_2SO_4, and filtered. The filtrate was concentrated via rotary evaporator and was transferred to a 50 mL centrifuge tube. Et_2O was added to precipitate the product out of solution as a white solid and the product was collected via centrifugation. The supernatant was decanted off and the white solid pellet was redissolved in a minimal amount of CH_2Cl_2. The precipitation and centrifugation processes were repeated a second time. The pellet was then dried on high vacuum and was taken forward to mesylation without further purification.

Step 2: Stoichiometry assumes full conversion for previous step. A solution of crude terpy hydroquinone triethylene glycol (1.87 mmol, 1.0 eq.) and Et_3N (2.85 g, 3.9 mL, 28.2 mmol, 15 eq.) was prepared in 150 mL CH_2Cl_2 in a 500 mL RB flask fitted with a stir bar. The solution was cooled using an ice bath under N_2 before adding a solution of mesyl chloride (MsCl) (2.15 g, 1.45 mL, 18.76 mmol, 10 eq.) in 100 mL CH_2Cl_2 via slow addition funnel. The reaction was allowed to warm up to room temperature while stirring under N_2 for 2 d. The solution was then diluted with 300 mL CH_2Cl_2 and was washed with 3 x 100 mL 1 M CH_3COOH and 2 x 100 mL aqueous saturated $NaHCO_3$. The organics were dried over Na_2SO_4 and were filtered. The solvent was removed via rotary evaporator. The crude was redissolved in 25 mL GPC grade DMF and was filtered. It was then purified via recycling prep-GPC with DMF over five injections to afford the product as a white solid (0.97 g, 45% over two steps). ^{1}H NMR (500 MHz, $CDCl_3$): δ_H 8.53 (d, J = 8.8 Hz, 2H), 8.39 (d, J = 2.9 Hz, 2H), 8.29 (d, J = 7.8 Hz, 2H), 7.88 (t, J = 7.8 Hz, 1H), 7.37 (dd, J = 8.8, 2.9 Hz, 2H), 6.88 – 6.77 (m, 8H), 4.38 – 4.34 (m, 4H), 4.27 – 4.23 (m, 4H), 4.10 – 4.05 (m, 4H), 4.05 – 4.00 (m, 4H), 3.94 – 3.90 (m, 4H), 3.87 – 3.82 (m, 4H), 3.81 – 3.73 (m, 16H), 3.72 – 3.64 (m, 8H), 3.03 (s, 6H). ^{13}C NMR (125 MHz, $CDCl_3$): δ_C 155.6, 155.1, 153.3, 153.1, 149.3, 137.9, 137.2, 122.2, 121.9, 119.7, 115.7, 115.6, 71.1, 71.0, 70.8, 70.8, 70.1, 70.0, 69.8, 69.4, 69.2, 68.2, 68.1, 37.8. MALDI-TOF: calculated for $C_{53}H_{71}N_3O_{20}S_2$: m/z = 1134.4 $[M + H]^+$; Found: 1134.7 $[M + H]^+$ (2,5-dihydroxybenzoic acid matrix).

*k) Symmetric Terpy-Phen Macrocycle (**3**)*

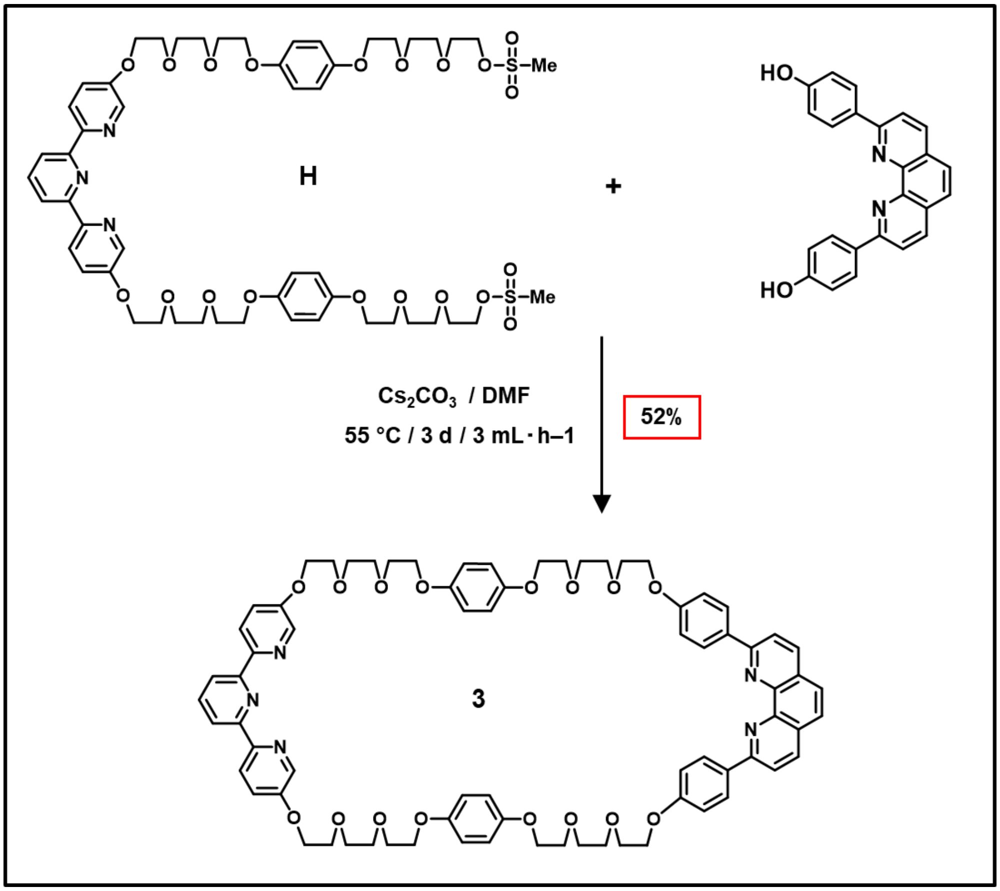

Scheme S11. Synthesis of compound **3**.

A suspension of phen diol[1] (0.312 g, 0.85 mmol, 1.0 eq.) and **H** (0.966 g, 0.85 mmol, 1.0 eq.) was prepared in 50 mL DMF and was added via syringe at 3 $mL{\cdot}h^{-1}$ using a syringe pump to a suspension of Cs_2CO_3 (2.80 g, 8.5 mmol, 10 eq.) in 300 mL DMF in a 500 mL RB flask. The reaction was heated to 55 °C for a total of 3 d. After 3 d, the suspension was filtered via gravity filtration to remove insoluble salts. The filtrate was concentrated via rotary evaporator and the crude was taken up in 300 mL CH_2Cl_2. The organics were washed with 3 x 100 mL DI H_2O and the aqueous layer was back-extracted with 3 x 50 mL CH_2Cl_2. The combined organics were dried over Na_2SO_4 and were filtered. The solvent was removed to afford the crude product as a sticky brown film. This was redissolved in 20 mL GPC grade DMF and was filtered via syringe filter. It was then purified via recycling preparative GPC with DMF over four injections to afford the product

as a sticky orange solid (0.57 g, 52%). ^{1}H NMR (500 MHz, $CDCl_3$): δ_H 8.49 (d, *J* = 8.8 Hz, 2H), 8.40 (d, *J* = 8.8 Hz, 4H), 8.37 (d, *J* = 2.9 Hz, 2H), 8.28 (d, *J* = 7.8 Hz, 2H), 8.21 (d, *J* = 8.4 Hz, 2H), 8.03 (d, *J* = 8.4 Hz, 2H), 7.86 (t, *J* = 7.8 Hz, 1H), 7.69 (s, 2H), 7.32 (dd, *J* = 8.8, 2.9 Hz, 2H), 7.09 (d, *J* = 8.8 Hz, 4H), 6.78 (s, 8H), 4.24 – 4.16 (m, 8H), 4.03 – 3.97 (m, 8H), 3.91 – 3.84 (m, 8H), 3.81 – 3.67 (m, 24H). ^{13}C NMR (125 MHz, $CDCl_3$): δ_C 160.2, 156.3, 155.5, 155.1, 153.2, 153.1, 149.3, 146.1, 137.8, 137.4, 136.8, 132.3, 129.0, 127.6, 125.7, 121.9, 121.8, 119.5, 119.32, 115.66, 115.66, 115.0, 71.04, 71.00, 70.96, 70.93, 69.97, 69.8, 69.7, 68.13, 68.06, 67.6. MALDI-TOF: calculated for $C_{75}H_{79}N_5O_{16}$: *m/z* = 1328.5 [*M* + Na]$^+$; Found: 1329.5 [*M* + Na]$^+$ (2,5-dihydroxybenzoic acid matrix).

2) Synthesis of Linear Catenanes

*a) Fe^{2+}-Open Terpy-Phen Macrocycle Dimer (**Fe-(2)$_2$**)*

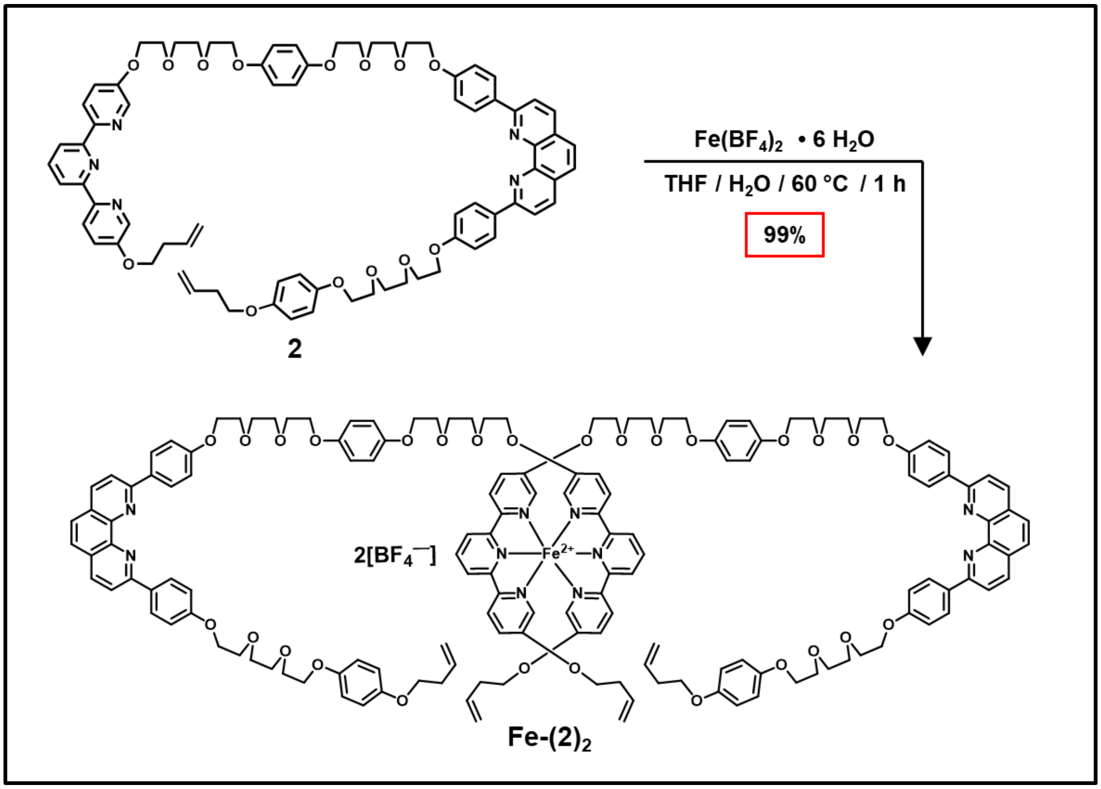

Scheme S12. Synthesis of compound **Fe-(2)$_2$**.

We have previously reported[1] the synthesis of **Fe-(2)$_2$**. Herein, we report an improved and scaled synthetic protocol. A solution of **2** (0.500 g, 0.385 mmol, 1 eq.) was prepared in 150 mL THF in a 250 mL RB flask. The flask was purged with N_2 and a solution of $Fe(BF_4)_2$ • 6 H_2O (0.169 g, 0.499 mmol, 1.3 eq.) in 25 mL DI H_2O was added via syringe. The dark red solution was heated at 60 °C for 1 h while stirring under N_2. The solution was then allowed to cool to room temperature before diluting with 300 mL CH_2Cl_2. The dark red solution was washed with 3 x 100 mL brine. The aqueous layer was back-extracted with 2 x 100 mL CH_2Cl_2. The combined organics were dried over Na_2SO_4 and were filtered. The solvent was removed via rotary evaporator to afford the product as a dark red sticky solid (0.542 g, 99%). ^1H NMR is consistent with our previous work.

*b) [2]Catenane-TPM (**[2]C**) and Figure-Eight TPM (**I**)*

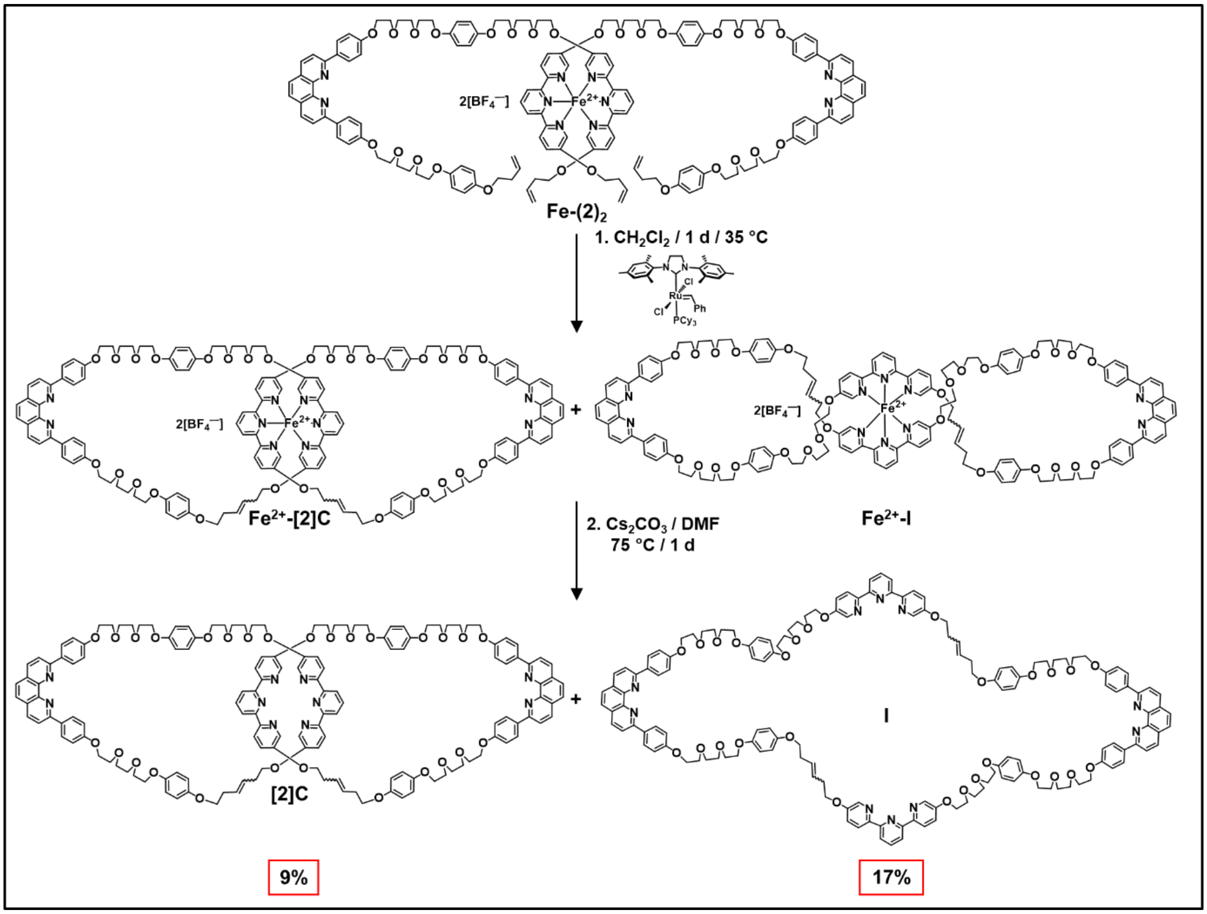

Scheme S13. Synthesis of compounds **[2]C** and **I**.

<u>Step 1:</u> A solution of **Fe-(2)₂** (0.209 g, 0.074 mmol, 1 eq.) and Grubbs' 2nd generation catalyst (0.012 g, 0.014 mmol, 0.2 eq.) was prepared in 200 mL CH_2Cl_2 in a 500 mL RB fitted with a Vigreux column. The dark red solution was then heated to 35 °C while stirring under N_2. The reaction progress was monitored by LR-ESI. After 1 d, the reaction was complete and was quenched with 1 mL ethyl vinyl ether (EVE) and 5 mL MeCN. The solvent was removed to afford the crude product as a dark red film. The crude material was purified via column chromatography with basic alumina ($CHCl_3$ to 5% MeOH / $CHCl_3$). All fractions, including mixed fractions, containing the desired product were collected to be demetalated prior to purification via recycling preparative GPC.

Step 2: The mixed fractions were redissolved in 25 mL DMF in a 50 mL RB flask with a stir bar. Solid Cs_2CO_3 (1.0 g, 3.07 mmol, 40 eq.) was added to the dark red solution and the suspension was heated at 75 °C while stirring open to air. After 1 d, the solvent was removed via rotary evaporator and the crude product was taken up in 200 mL CH_2Cl_2. The organics were washed with 3 x 50 mL brine. The aqueous layer was back-extracted with 2 x 100 mL CH_2Cl_2. The combined organics were dried over Na_2SO_4 and were filtered. The solvent was removed via rotary evaporator to afford the crude product as an orange film. It was redissolved in 5 mL GPC grade DMF and was filtered via syringe filter, followed by purification via recycling prep-GPC with DMF over one injection to separate the desired [2]catenane product **[2]C** (0.0163 g, 9%) from the large macrocycle **I** (0.0327 g, 17%). The identity of these species was determined by HR-ESI-MS/MS.

[2]C: ^{1}H NMR (500 MHz, $CDCl_3$): δ 8.47 (d, *J* = 8.7 Hz, 4H), 8.43 – 8.29 (m, 12H), 8.25 (d, *J* = 7.9 Hz, 4H), 8.19 (d, *J* = 8.4 Hz, 4H), 8.01 (d, *J* = 8.4 Hz, 4H), 7.83 (t, *J* = 7.8 Hz, 2H), 7.71 – 7.64 (m, 4H), 7.27 (dd, *J* = 8.2, 2.3 Hz, 2H), 7.23 (dd, *J* = 8.8, 2.9 Hz, 2H), 7.12 – 7.01 (m, 8H), 6.85 – 6.71 (m, 16H), 5.65 – 5.53 (m, 4H), 4.28 – 4.10 (m, 7H), 4.08 – 3.94 (m, 8H), 3.92 – 3.59 (m, 27H), 2.56 – 2.38 (m, 4H). ^{13}C NMR (125 MHz, $CDCl_3$): δ_C 160.2, 158.5, 156.3, 155.54, 155.48, 155.2, 155.1, 153.3, 153.2, 153.14, 153.08, 149.3, 149.2, 149.1, 146.1, 137.8, 137.6, 137.4, 136.9, 132.4, 129.2, 129.1, 127.8, 127.6, 125.7, 121.9, 121.8, 121.4, 119.5, 119.4, 115.7, 115.6, 115.5, 115.0, 114.9, 71.06, 71.00, 70.95, 70.92, 70.88, 70.03, 69.99, 69.97, 69.85, 69.74, 68.15, 68.10, 68.07, 67.99, 67.9, 67.7, 67.6, 32.7, 32.5. HRMS-ESI: calculated for $C_{150}H_{154}N_{10}O_{28}$: *m*/z = 1273.0557 $[M+2H]^{2+}$, 849.0395 $[M+H]^{3+}$, 637.0315 $[M+4H]^{4+}$; Found: 1273.0527 $[M+2H]^{2+}$, 849.0367 $[M+H]^{3+}$, 637.0292 $[M+4H]^{4+}$.

I: ^{1}H NMR (500 MHz, $CDCl_3$): δ 8.49 (dd, *J* = 8.7, 4.1 Hz, 4H), 8.41 (d, *J* = 8.6 Hz, 8H), 8.37 (d, *J* = 2.9 Hz, 2H), 8.33 (d, *J* = 2.8 Hz, 2H), 8.28 (d, *J* = 7.8 Hz, 4H), 8.21 (d, *J* = 8.4 Hz, 4H), 8.04 (d, *J* = 8.4 Hz, 4H), 7.86 (t, *J* = 7.8 Hz, 2H), 7.70 (s, 4H), 7.35 – 7.26 (m, 4H), 7.10 (dd, *J* = 8.7, 1.5 Hz, 8H), 6.88 – 6.73 (m, 16H), 5.62 (d, *J* = 5.4 Hz, 4H), 4.26 – 4.17 (m, 12H), 4.04 (ddd, *J* = 13.1,

9.6, 5.1 Hz, 16H), 3.94 – 3.66 (m, 50H), 2.63 – 2.39 (m, 8H). ^{13}C NMR (125 MHz, $CDCl_3$): δ_C 160.2, 156.4, 155.54, 155.49, 155.22, 155.15, 153.3, 153.22, 153.17, 153.1, 149.4, 149.3, 149.2, 146.1, 137.8, 137.3, 136.9, 132.4, 129.3, 129.1, 128.4, 127.9, 127.6, 127.1, 125.7, 121.9, 121.83, 121.81, 121.7, 119.6, 119.4, 71.1, 71.00, 70.99, 70.96, 70.06, 70.03, 69.9, 69.8, 68.24, 68.21, 68.18, 68.15, 68.05, 67.9, 67.7, 32.8, 32.6. HRMS-ESI: calculated for $C_{150}H_{154}N_{10}O_{28}$: m/z = 1273.0557 $[M+2H]^{2+}$, 849.0395 $[M+H]^{3+}$, 637.0315 $[M+4H]^{4+}$; Found: 1273.0548 $[M+2H]^{2+}$, 849.0383 $[M+H]^{3+}$, 637.0303 $[M+4H]^{4+}$.

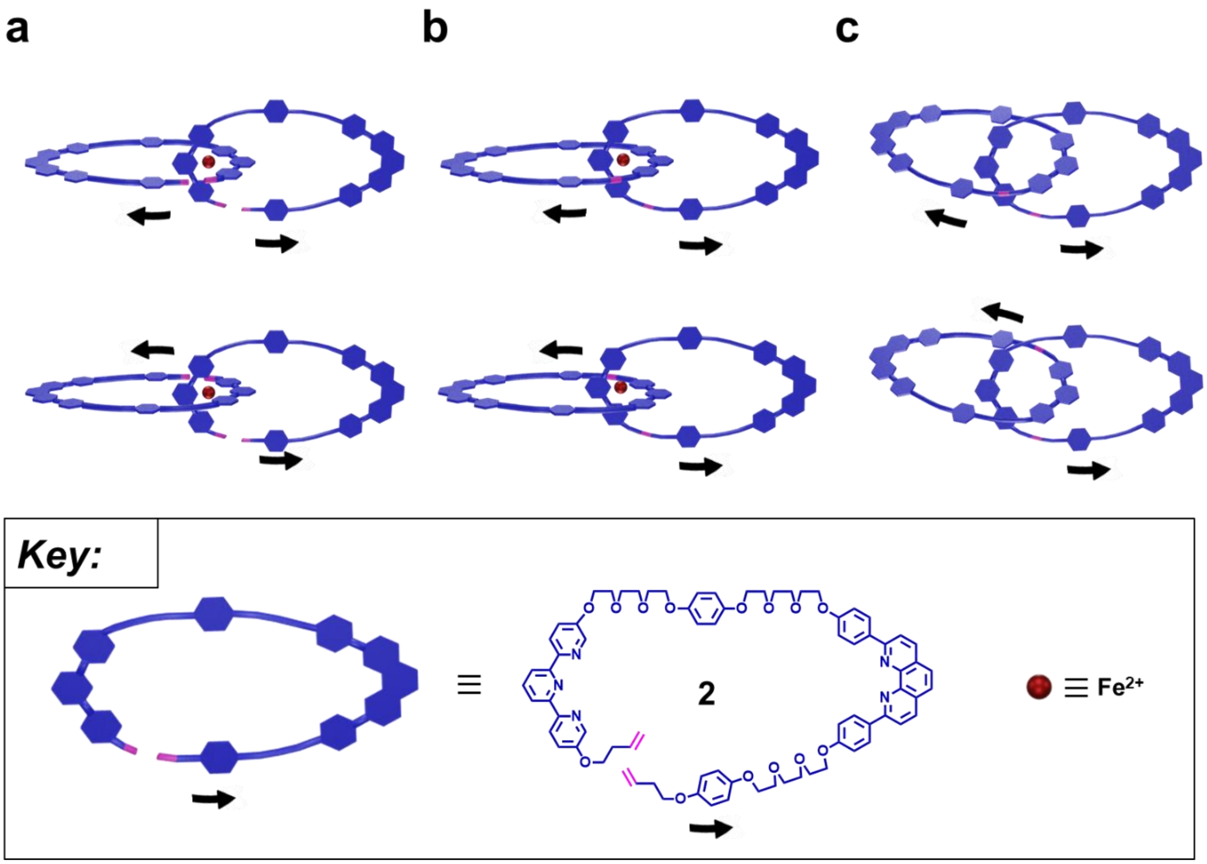

Figure S1. (**a**) Cartoon representation of the enantiomers of **Fe-(2)$_2$**. (**b**) Cartoon representation of the enantiomers of [2]catenate complex **Fe-[2]C**. (**c**) Cartoon representation of the enantiomers of **[2]C**. An arrow is used to easily show the orientation of the macrocycles in the enantiomeric species.

When **2** is metalated to form the bis-complex **Fe-(2)$_2$**, the directionality of the asymmetric open macrocycle is maintained, which results in a pair of enantiomeric coordination complexes. The

orientation of the macrocycles is preserved during ring-closing and after demetallation, resulting in enantiomers of **[2]C**.

*c) Fe^{2+}-Symmetric Terpy-Phen Macrocycle (**Fe-(3)₂**)*

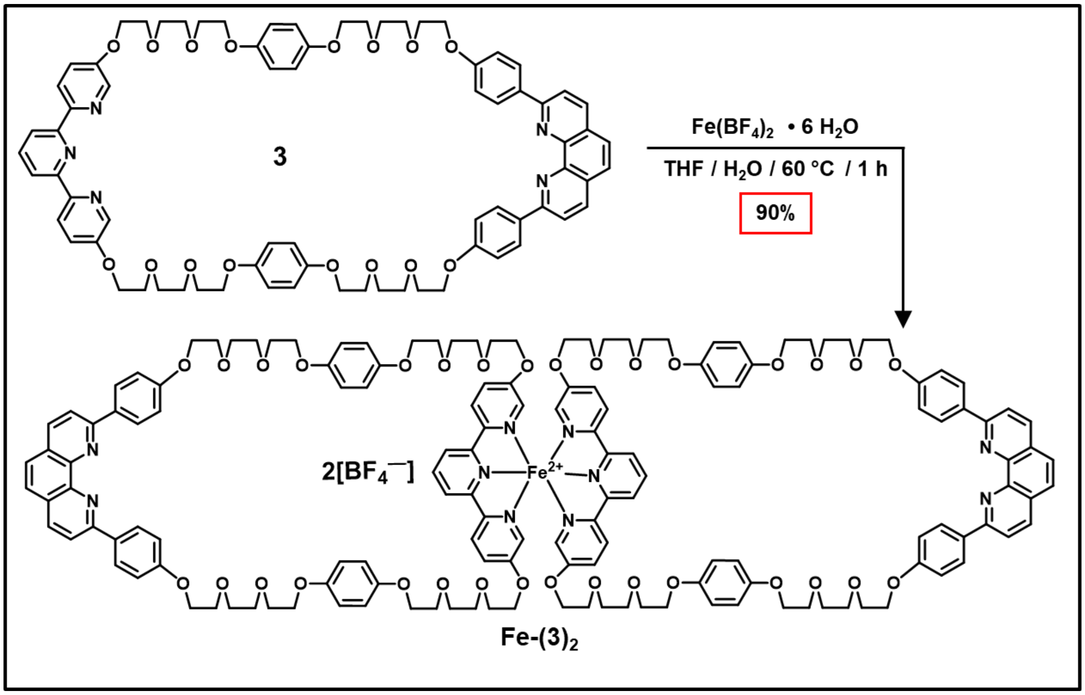

Scheme S14. Synthesis of compound **Fe-(3)$_2$**.

A solution of **3** (0.257 g, 0.197 mmol, 1 eq.) was prepared in 130 mL THF in a 250 mL RB flask. The flask was purged with N_2 and a solution of $Fe(BF_4)_2$ • 6 H_2O (0.099 g, 0.264 mmol, 1.5 eq.) in 20 mL DI H_2O was added via syringe. The dark red solution was heated at 60 °C for 1 h while stirring under N_2. The solution was then allowed to cool to room temperature. The solvent was then removed via rotary evaporator and the crude was taken up in 300 mL CH_2Cl_2. The dark red solution was washed with 3 x 100 mL DI H_2O . The organics were dried over Na_2SO_4 and were filtered. The solvent was then removed via rotary evaporator to afford the product as a dark red film. The film was then washed with 2 x 50 mL diethyl ether (Et_2O) and 2 x 50 mL hexanes. The crude product was isolated as a dark red solid (0.253 g, 90%) and was used in the next step without further purification. ^1H NMR (500 MHz, $CDCl_3$): δ 8.45 – 8.40 (m, 2H), 8.36 – 8.30 (m, 8H), 8.25 (d, *J* = 8.4 Hz, 4H), 8.20 (d, *J* = 7.9 Hz, 4H), 7.98 (d, *J* = 8.5 Hz, 4H), 7.93 (d, *J* = 8.8

Hz, 4H), 7.73 (s, 4H), 7.20 (dd, J = 9.0, 2.4 Hz, 4H), 7.09 (d, J = 8.8 Hz, 8H), 6.73 (dd, J = 62.4, 9.0 Hz, 16H), 6.35 (d, J = 2.5 Hz, 4H), 5.00 (s, 2H), 4.27 – 4.21 (m, 8H), 4.10 – 4.05 (m, 8H), 3.96 – 3.91 (m, 8H), 3.90 – 3.85 (m, 8H), 3.85 – 3.74 (m, 32H), 3.61 – 3.56 (m, 8H), 3.51 (s, 8H), 3.44 (dd, J = 11.3, 5.4 Hz, 14H). ^{13}C NMR (125 MHz, $CDCl_3$): δ_C 160.3, 159.2, 157.4, 156.0, 153.14, 153.05, 151.5, 149.4, 145.9, 142.1, 138.8, 137.1, 135.8, 132.1, 129.0, 128.3, 127.7, 125.8, 125.5, 124.7, 122.1, 120.9, 119.3, 115.7, 115.6, 115.5, 115.0, 70.93, 70.87, 70.46, 70.42, 70.0, 69.8, 69.6, 69.1, 68.8, 68.2, 68.1, 68.0, 67.8, 67.7, 34.3, 30.3. HRMS-ESI: calculated for $C_{150}H_{158}FeN_{10}O_{32}$: m/z = 1334.0209 $[M - 2BF_4]^{2+}$, 889.6830 $[M - 2BF_4 + H]^{3+}$, 667.5141 $[M - 2BF_4 + 2H]^{4+}$; Found: 1334.0167 $[M - 2BF_4]^{2+}$, 889.6805 $[M - 2BF_4 + H]^{3+}$, 667.5126 $[M - 2BF_4 + 2H]^{4+}$.

*d) [2]Catenane-Endcap Unsaturated (**[2]C-U**)*

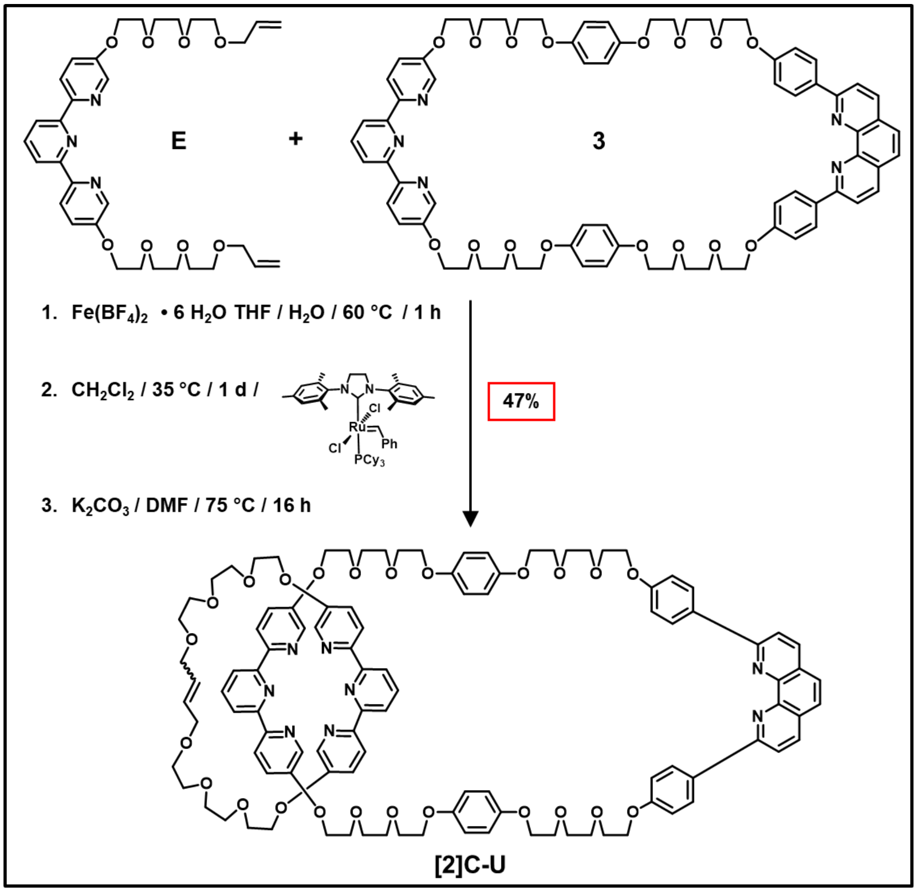

Scheme S15. Synthesis of compound **[2]C-U.**

Step 1: A solution of diallyl terpy **E** (0.271 g, 0.444 mmol, 2.0 eq.) and **3** (0.2817 g, 0.216 mmol, 1.0 eq.) was prepared in 300 mL THF in a 500 mL RB flask. While stirring under N_2, a solution of $Fe(BF_4)_2 \cdot 6\ H_2O$ (0.220 g, 0.65 mmol, 3.0 eq.) in 50 mL DI H_2O was added via syringe. The dark red solution was then heated to 60 °C for 1 h while stirring under N_2. The solution was then allowed to cool to room temperature while stirring for 2 d. The solvent was removed via rotary evaporator and crude material was taken up in 300 mL CH_2Cl_2. The dark red solution was washed with 3 x 100 mL DI H_2O. The organics were dried over Na_2SO_4 and were filtered. The solvent was removed via rotary evaporator and the crude product was isolated as a dark red film, which was taken forward to the next step without any further purification.

Step 2: The crude red film was redissolved in 500 mL CH_2Cl_2 in a 1 L RB flask. A solution of Grubbs' 2nd generation catalyst (0.018 g, 0.0216 mmol, 0.1 eq.) in 5 mL CH_2Cl_2 was added. The reaction was fitted with a Vigreux column and was heated to 35°C while stirring under N_2. The reaction progress was monitored by LR-ESI. After 8 h, an additional 0.05 eq. Grubbs' 2nd generation catalyst was added, and the reaction temperature was maintained at 35°C while stirring under N_2. After 1 d of total heating, the reaction was complete and was quenched with 5 mL EVE and 5 mL MeCN. The solvent was removed via rotary evaporator and the crude was transferred to a 100 mL RB flask.

Step 3: The crude red film was redissolved in 50 mL DMF, followed by the addition of solid K_2CO_3 (3.0 g, 21.7 mmol, 100 eq.). The suspension was then heated to 75°C for 16 h while stirring open to air. The solvent was then removed via rotary evaporator and the crude product was taken up in 300 mL CH_2Cl_2. The solution was washed with 2 x 100 mL DI H_2O and 2 x 100 mL brine. The organics were dried over Na_2SO_4 and were filtered. The solvent was removed via rotary evaporator to afford the crude demetalated mixture as a sticky brown solid. It was redissolved in 12 mL GPC grade DMF and was filtered via syringe filter, followed by purification via recycling

prep-GPC with DMF over three injections to afford to afford the asymmetric [2]catenane product as a sticky orange solid (0.189 g, 47%). ^{1}H NMR (500 MHz, $CDCl_3$): δ 8.57 – 8.48 (m, 4H), 8.40 (d, *J* = 8.8 Hz, 4H), 8.35 (d, *J* = 2.9 Hz, 2H), 8.29 – 8.25 (m, 4H), 8.23 (d, *J* = 8.4 Hz, 2H), 8.17 (d, *J* = 7.8 Hz, 2H), 8.05 (d, *J* = 8.4 Hz, 2H), 7.84 (t, *J* = 7.8 Hz, 1H), 7.80 (t, *J* = 7.8 Hz, 1H), 7.72 (s, 2H), 7.36 (dd, *J* = 8.9, 2.9 Hz, 2H), 7.30 (dd, *J* = 8.8, 2.9 Hz, 2H), 7.07 (d, *J* = 8.8 Hz, 4H), 6.78 (s, *J* = 2.3 Hz, 8H), 5.57 – 5.53 (m, 2H), 4.24 – 4.20 (m, 4H), 4.17 (dd, *J* = 9.8, 6.3 Hz, 8H), 4.04 – 3.99 (m, 8H), 3.85 (dd, *J* = 10.9, 7.1 Hz, 8H), 3.81 – 3.67 (m, 32H), 3.64 – 3.60 (m, 4H), 3.60 – 3.55 (m, 4H), 3.54 (dd, *J* = 5.9, 4.2 Hz, 4H), 3.47 – 3.41 (m, 4H). ^{13}C NMR (125 MHz, $CDCl_3$): δ_C 160.2, 156.3, 155.7, 155.5, 155.08, 155.06, 153.14, 153.11, 149.30, 149.26, 146.1, 138.6, 137.8, 137.7, 137.4, 136.9, 132.3, 129.6, 129.1, 127.6, 125.7, 122.5, 121.9, 121.83, 121.76, 119.5, 119.4, 119.1, 115.6, 114.9, 71.3, 71.03, 70.96, 70.7, 70.00, 69.96, 69.8, 69.7, 69.4, 68.3, 68.1, 68.0, 67.5. MALDI-TOF: calculated for $C_{106}H_{118}N_8O_{24}$: *m/z* = 1888.8 $[M + H]^+$; Found: 1889.0 $[M + H]^+$ (2,5-dihydroxybenzoic acid matrix).

*e) [2]Catenane-Endcap (**[2]C-E**)*

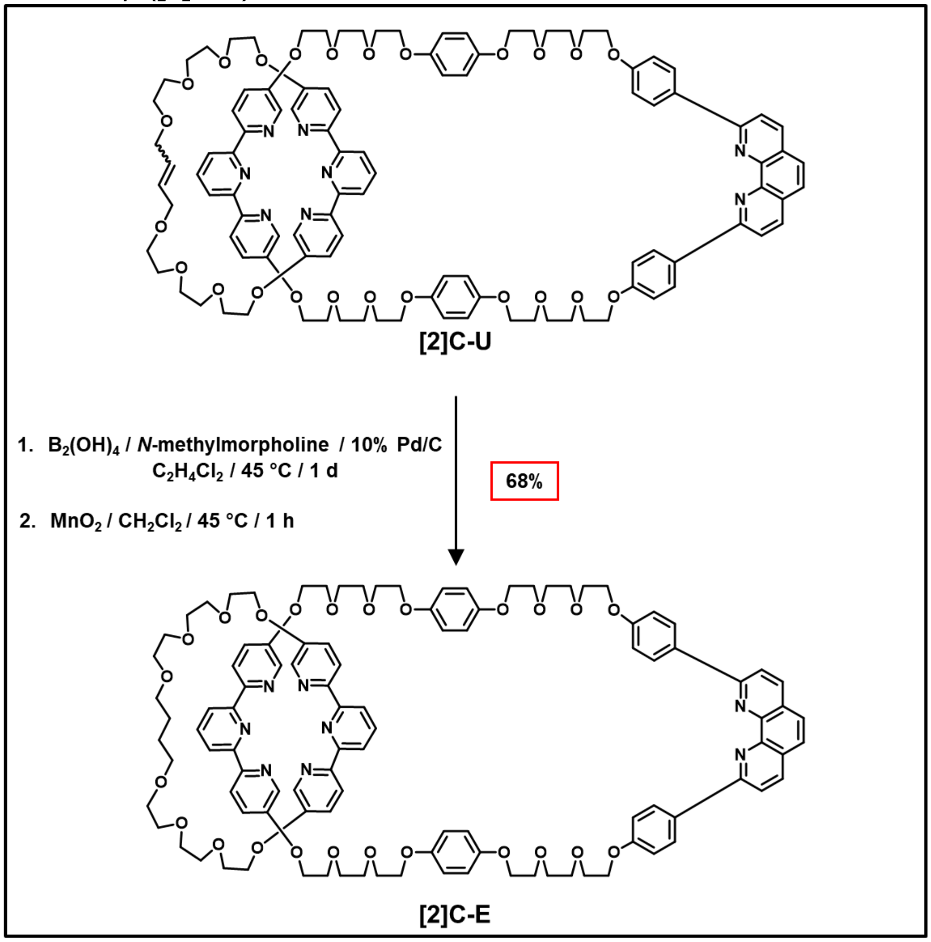

Scheme S16. Synthesis of compound **[2]C-E**.

<u>Step 1:</u> A solution of **[2]C-E** (0.0701 g, 0.0371 mmol, 1 eq.) was prepared in 5 mL $C_2H_4Cl_2$ in a 10 mL high-pressure vessel with a stir bar. The vessel was then charged with $B_2(OH)_4$ (0.341 g, 3.80 mmol, 100 eq.), *N*-methylmorpholine (0.75 g, 0.82 mL, 7.43 mmol, 200 eq.) and 10% Pd on carbon (0.035 g, 0.5 eq. wt/wt).[3] It was quickly sealed and was heated to 45°C while stirring for 1 d. The reaction was then allowed to cool to room temperature. The catalyst was removed via gravity filtration and the black solid was washed with 100 mL 10% MeOH / CH_2Cl_2. The filtrate was washed with 3 x 50 mL brine. The organics were dried over Na_2SO_4 and were filtered. The solvent was removed rotary evaporator and the crude yellow film was taken forward to the rearomatization step without further purification.

Step 2: The crude yellow film was redissolved in 25 mL CH_2Cl_2 in a 100 mL RB flask. Solid MnO_2 (1.4 g, 20 eq. wt/wt) was added, and the flask was fitted with a Vigreux column. The suspension was heated to 45°C while stirring under N_2 for 1 h. The crude material was then filtered over celite by vacuum filtration and the cake was washed with 200 mL 10% MeOH / CH_2Cl_2. The filtrate was concentrated and was then purified via a short basic alumina column (CH_2Cl_2 to 5% MeOH / CH_2Cl_2), which afforded the product as a as a yellow film (0.048 g, 68%). ^{1}H NMR (500 MHz, $CDCl_3$): δ 8.53 – 8.47 (m, 4H), 8.40 (d, *J* = 8.8 Hz, 4H), 8.35 (d, *J* = 2.9 Hz, 2H), 8.30 – 8.22 (m, 6H), 8.16 (d, *J* = 7.8 Hz, 2H), 8.06 (d, *J* = 8.5 Hz, 2H), 7.84 (t, *J* = 7.8 Hz, 1H), 7.79 (t, *J* = 7.8 Hz, 1H), 7.73 (s, 2H), 7.35 (dd, *J* = 8.9, 3.0 Hz, 2H), 7.31 (dd, *J* = 8.8, 2.9 Hz, 2H), 7.07 (d, *J* = 8.8 Hz, 4H), 6.79 (s, 8H), 4.27 – 4.14 (m, 12H), 4.05 – 3.99 (m, 8H), 3.89 – 3.83 (m, 8H), 3.82 – 3.67 (m, 28H), 3.66 – 3.57 (m, 8H), 3.53 (t, *J* = 5.2 Hz, 4H), 3.47 – 3.41 (m, 4H), 3.15 (d, *J* = 15.0 Hz, 4H). ^{13}C NMR (125 MHz, $CDCl_3$): δ_C 160.2, 156.4, 155.7, 155.5, 155.1, 155.0, 153.2, 153.1, 149.31, 149.27, 146.1, 138.4, 137.8, 137.7, 137.4, 136.9, 132.3, 129.1, 127.6, 125.7, 122.5, 122.0, 121.84, 121.79, 119.53, 119.47, 119.1, 115.64, 115.62, 114.9, 71.3, 71.1, 71.04, 70.97, 70.95, 70.90, 70.65, 70.63, 70.01, 69.98, 69.92, 69.86, 69.7, 68.2, 68.1, 68.0, 67.6, 26.2. MALDI-TOF: calculated for $C_{106}H_{120}N_8O_{24}$: *m/z* = 1889.9 [*M* + H]$^+$; Found: 1889.09 [*M* + H]$^+$ (α-cyano-4-hydroxycinnamic acid matrix).

*f) Fe^{2+}-[2]C-Endcap (**Fe-[2]C-E**)*

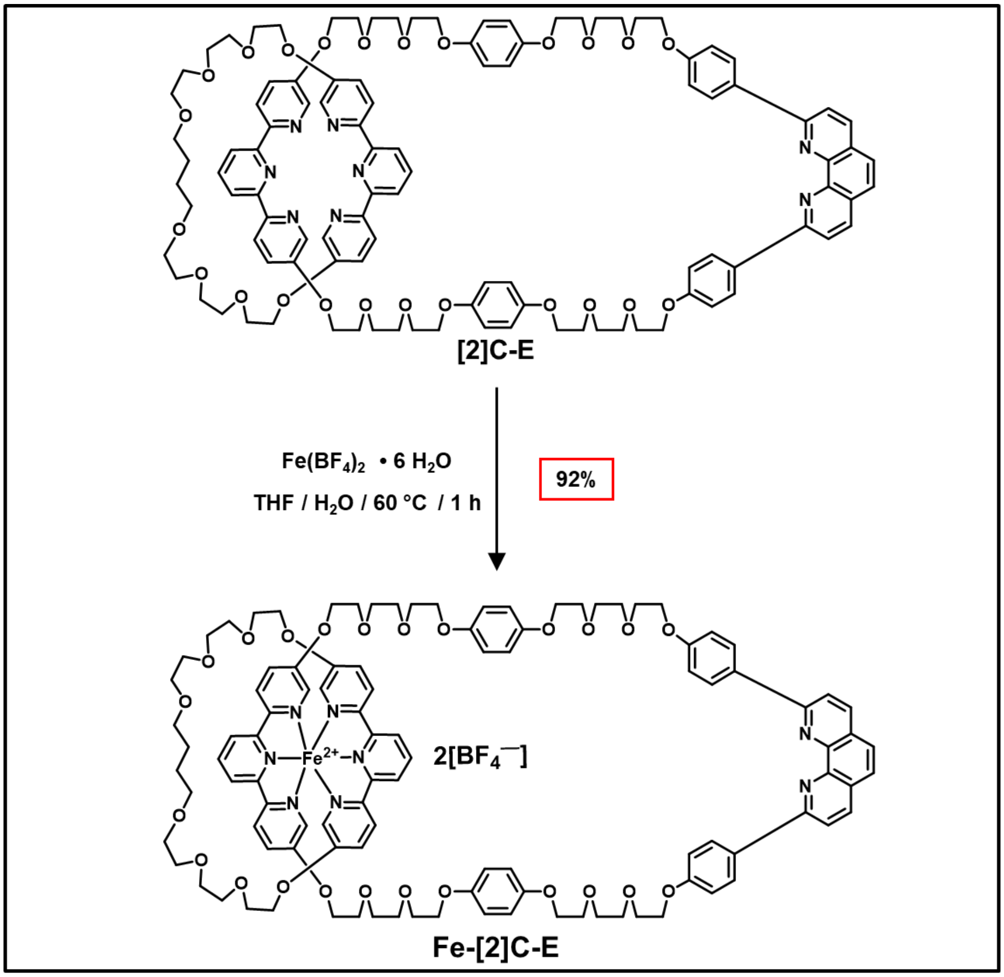

Scheme S17. Synthesis of compound **Fe-[2]C-E**.

A solution of **Fe-[2]C-E** (0.183 g, 0.0968 mmol, 1 eq.) was prepared in 100 mL 25% MeOH / THF in a 100 mL RB flask. The flask was purged with N_2 and a solution of $Fe(BF_4)_2$ • 6 H_2O (0.0507 g, 0.1501 mmol, 1.5 eq.) in 10 mL DI H_2O was added via syringe. The dark red solution was heated to 60°C for 1 h while stirring under N_2. The solution was then allowed to cool to room temperature and the solvent was removed via rotary evaporator. The crude product was taken up in 300 mL CH_2Cl_2 and was washed with 3 x 100 mL DI H_2O. The aqueous layer was back-extracted with 3 x 100 mL CH_2Cl_2. The combined organics were dried over Na_2SO_4 and were filtered. The solvent was removed via rotary evaporator to afford a dark red film. The crude product was redissolved

in a minimal amount of CH_2Cl_2 and was transferred to a 50 mL centrifuge tube. The product was precipitated out of solution by the addition of hexanes. The red suspension was centrifuged, and the supernatant was discarded. The dark red pellet was redissolved in CH_2Cl_2 and was again precipitated out of solution by the addition of hexanes. The red suspension was centrifuged, and the supernatant was discarded. The dark red pellet was redissolved in CH_2Cl_2 and was transferred to a vial. The solvent was removed via rotary evaporator to afford the product as a dark red solid (0.1881 g, 92%) and was used in the next step without further purification. ^1H NMR (500 MHz, $CDCl_3$): δ 8.81 (d, *J* = 8.1 Hz, 2H), 8.62 – 8.54 (m, 1H), 8.49 (d, *J* = 9.0 Hz, 2H), 8.44 – 8.36 (m, 6H), 8.30 – 8.22 (m, 3H), 8.18 (d, *J* = 8.9 Hz, 2H), 8.07 (d, *J* = 8.5 Hz, 2H), 7.76 (s, 2H), 7.36 (dd, *J* = 9.0, 2.6 Hz, 2H), 7.22 (dd, *J* = 8.9, 2.6 Hz, 2H), 7.09 (d, *J* = 8.8 Hz, 4H), 6.89 – 6.75 (m, 8H), 6.61 (d, *J* = 2.6 Hz, 2H), 6.50 (d, *J* = 2.6 Hz, 2H), 4.27 – 4.20 (m, 4H), 4.15 – 4.10 (m, 4H), 4.02 – 3.50 (m, 58H), 3.46 (dd, *J* = 5.7, 3.5 Hz, 4H), 3.36 (dd, *J* = 5.6, 3.6 Hz, 4H), 1.84 – 1.73 (m, 4H). ^{13}C NMR (125 MHz, $CDCl_3$): δ_C 160.3, 159.59, 159.56, 157.6, 157.5, 153.3, 153.2, 149.96, 149.94, 146.1, 141.8, 140.9, 138.9, 138.4, 137.1, 132.3, 129.0, 127.7, 125.9, 125.3, 124.9, 124.1, 122.3, 121.9, 121.2, 119.4, 115.86, 115.80, 115.1, 71.5, 71.1, 71.0, 70.9, 70.69, 70.64, 70.56, 70.47, 70.06, 69.93, 69.87, 69.80, 69.0, 68.9, 68.7, 68.3, 68.1, 67.7, 26.9. HRMS-ESI: calculated for $C_{106}H_{120}FeN_8O_{24}$: *m*/z = 972.8894 $[M – 2BF_4]^{2+}$, 648.9287 $[M – 2BF_4 + H]^{3+}$; Found: 972.8888 $[M – 2BF_4]^{2+}$, 648.9285 $[M – 2BF_4 + H]^{3+}$.

*g) [3]Catenane (**[3]C**)*

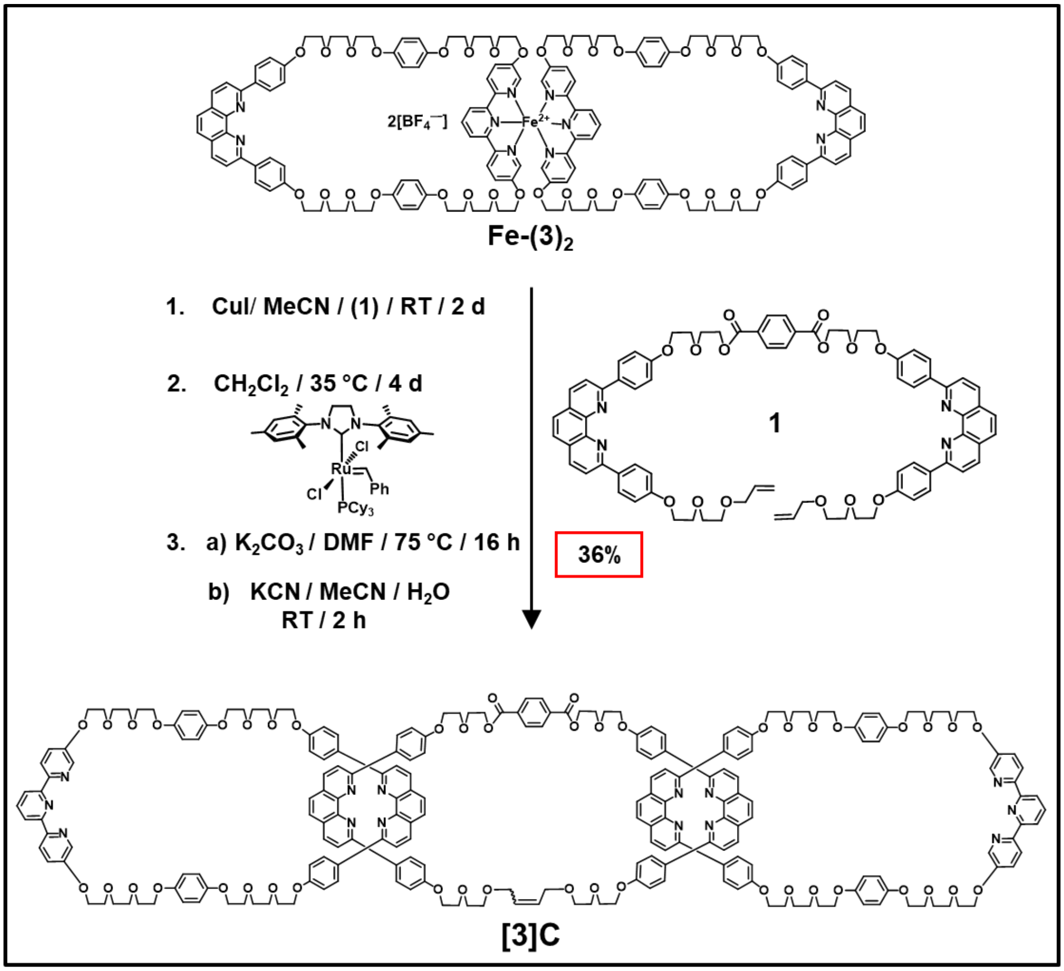

Scheme S18. Synthesis of **[3]C**.

Step 1: A solution of **Fe-(3)$_2$** (1.08 g, 0.38, mmol, 4.0 eq.) was prepared in 100 mL MeCN in a 500 mL RB flask with a stir bar. While stirring at 25 °C under N_2, a solution of CuI (0.135 g, 0.71 mmol, 7.5 eq.) in 50 mL MeCN was added. After stirring for 1 h, a solution of **1** (0.124 g, 0.096 mmol, 1.0 eq.) in 25 mL CH_2Cl_2 was added via syringe. The reaction was continued to stir at 25 °C for 16 h. The solvent was then removed via rotary evaporator and the crude dark red film was dried on high vacuum for several hours before ring-closing.

Step 2: The crude pre-[3]catenate complex redissolved in 250 mL CH_2Cl_2 in a 500 mL RB flask. A solution of Grubbs' 2nd generation catalyst (0.008 g, 0.0095 mmol, 0.1 eq.) was added and the

RB flask was fitted with a Vigreux column. The reaction was heated to 35 °C while stirring under N_2 for 3 d. An extra 0.1 eq. of Grubbs' 2nd generation catalyst was added, and the reaction was continued to heat for an additional 1 d. The reaction was then quenched with EVE and the solvent was removed via rotary evaporator.

Step 3: a) The Fe^{2+} ion was first removed from the crude mixture with the addition of a weak inorganic base and moderate heating. The crude red film was redissolved in 50 mL DMF in a 100 mL RB flask. Solid K_2CO_3 (2.6 g, 18.9 mmol, 200 eq.) was added and the suspension was heated to 75°C for 16 h while stirring open to air. The solvent was removed via rotary evaporator. b) The Cu^+ then removed by addition of an excess of strongly competing cyanide ligand, in the form of potassium cyanide (KCN). The crude solid was taken up in 50 mL MeCN and the mixture was sonicated to form a suspension. While stirring open to air, a solution of KCN (0.93 g, 14.3 mmol, 150 eq.) in 10 mL DI H_2O was added via syringe. The dark red solution was allowed to stir at 25°C for 2 h. The suspension was diluted with 500 mL CH_2Cl_2 and the organics were washed with 3 x 100 mL brine. The aqueous layer was back-extracted with 3 x 100 mL CH_2Cl_2. The combined organics were dried over Na_2SO_4 and were filtered. The solvent was removed via rotary evaporator to afford the crude as a dark red film. The crude was redissolved in 30 mL DMF and centrifuged in a 50 mL centrifuge tube to remove any remaining insoluble salts. The supernatant was filtered via syringe filter and the crude was purified via recycling prep-GPC with DMF over five injections to afford the product as a sticky orange solid (0.135 g, 36%). Note that the aliphatic proton resonances integrate a little higher relative to the aromatic proton resonances; thus, the observed integrations are reported. ^{1}H NMR (500 MHz, $CDCl_3$): δ_H 8.44 (d, J = 8.8 Hz, 4H), 8.39 – 8.29 (m, 20H), 8.23 (d, J = 7.8 Hz, 4H), 8.15 (dd, J = 8.4, 1.9 Hz, 8H), 8.00 (s, 4H), 7.96 (d, J = 8.4 Hz, 8H), 7.81 (t, J = 7.8 Hz, 2H), 7.67 – 7.59 (m, 8H), 7.24 (dd, J = 8.8, 2.9 Hz, 4H), 7.07 – 6.93 (m, 20H), 6.80 – 6.70 (m, 16H), 5.71 (d, J = 2.8 Hz, 2H), 4.43 – 4.36 (m, 4H), 4.22 (dd, J = 9.2, 4.2 Hz, 4H), 4.14 – 4.06 (m, 16H), 4.06 – 4.01 (m, 8H), 3.99 – 3.93 (m, 16H), 3.92 – 3.88 (m,

6H), 3.84 – 3.60 (m, 80H), 3.60 – 3.55 (m, 4H), 3.51 – 3.45 (m, 4H). ^{13}C NMR (125 MHz, $CDCl_3$): δ_C 165.7, 160.2, 160.1, 156.26, 156.23, 155.5, 155.0, 153.15, 153.11, 149.2, 146.0, 137.7, 137.4, 136.9, 133.9, 132.25, 132.21, 132.15, 129.8, 129.7, 129.6, 129.06, 129.03, 127.6, 125.7, 121.9, 121.7, 119.49, 119.43, 119.39, 115.6, 114.93, 114.89, 71.22, 71.17, 71.00, 70.95, 70.85, 70.82, 70.77, 70.1, 70.0, 69.9, 69.80, 69.78, 69.72, 69.69, 69.5, 69.3, 68.09, 68.05, 67.9, 67.63, 67.53, 67.49, 64.6, 26.4. MALDI-TOF: calculated for $C_{226}H_{228}N_{14}O_{46}$: *m/z* = 3874.6 [*M* + H]$^+$; Found: 3876.2 [*M* + H]$^+$ (2,5-dihydroxybenzoic acid matrix).

*h) [4]Catenane (**[4]C**)*

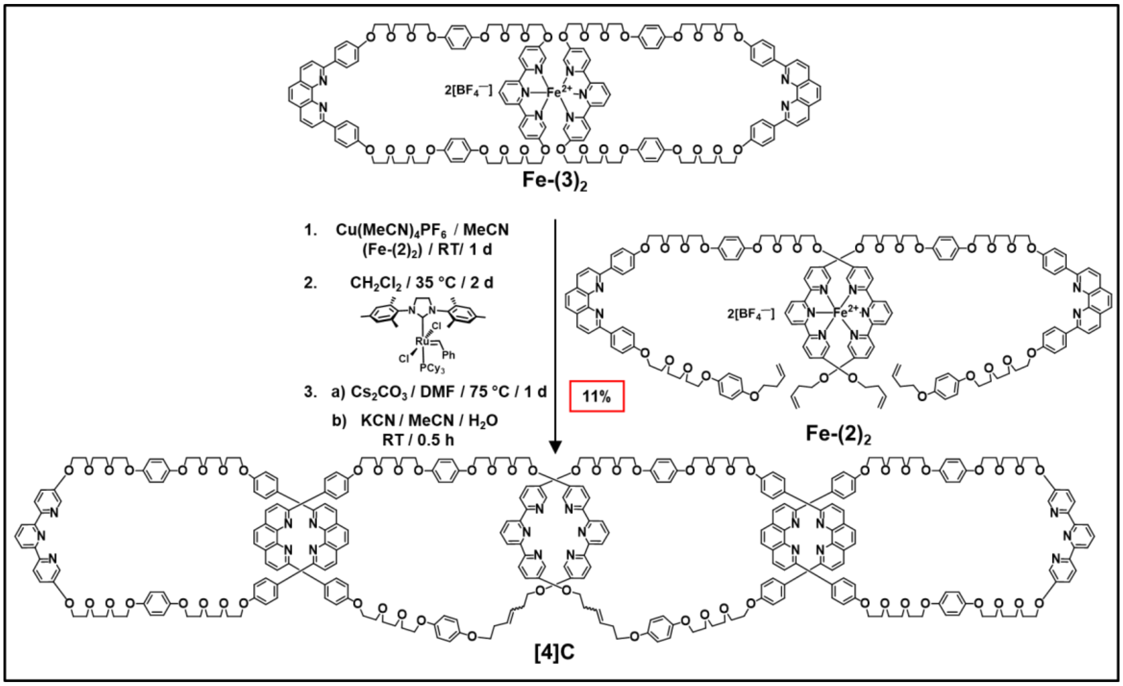

Scheme S19. Synthesis of **[4]C**.

<u>Step 1:</u> A solution of **Fe-(3)$_2$** (0.247 g, 0.0868 mmol, 4.0 eq.) in 25 mL N_2-purged anhydrous MeCN was added via syringe to solid $Cu(MeCN)_4PF_6$ (0.0616 g, 0.165 mmol, 7.5 eq.) in an oven-dried 100 mL RB flask. The dark red solution was stirred at room temperature under N_2. After 30 min, a solution of **Fe-(2)$_2$** (0.0643 g, 0.0227 mmol, 1.0 eq.) in 20 mL 50% MeCN / CH_2Cl_2 was added via syringe. The dark red solution was stirred at room temperature under N_2 for 1 d. The solvent

was then removed via rotary evaporator, and the crude product was taken up in 100 mL CH_2Cl_2. The dark red solution was washed with 3 x 25 mL DI H_2O. The organics were dried over Na_2SO_4 and were filtered. The solvent was removed via rotary evaporator to afford the pre-[4]catenate complex as a foamy red solid, which was taken forward to the next step without further purification.

Step 2: The crude foamy red solid was redissolved in 60 mL anhydrous CH_2Cl_2 in a 250 mL RB flask with a stir bar. A solution of Grubbs' 2nd generation catalyst (0.004 g, 0.0045 mmol, 0.2 eq.) in 1 mL CH_2Cl_2 was added. The flask was fitted with a Vigreux column, and the dark red solution was heated to 35°C while stirring under N_2. After 1 d, an additional 0.2 eq. Grubbs' 2nd generation catalyst was added. The reaction was heated at 35°C while stirring under N_2 for an additional 1 d. After 2 d of total heating, the reaction was quenched with 1 mL EVE and 5 mL MeCN. The solvent was removed via rotary evaporator to afford the crude [4]catenate mixture as a dark red film.

Step 3: a) The Fe^{2+} ion was first removed from the crude mixture with the addition of a weak inorganic base and moderate heating. The crude red film was redissolved in 50 mL DMF in a 250 mL RB flask. Solid Cs_2CO_3 (1.0 g, 3.07 mmol, 135 eq.) was added and the suspension was heated to 75°C for 1 d while stirring open to air. b) The Cu^+ then removed by addition of an excess of strongly competing ligand, namely KCN. The solvent was then removed via rotary evaporator and 50 mL MeCN was added to the crude suspension. A solution of KCN (0.2 g, 3.07 mmol, 135 eq.) in 10 mL H_2O was added via syringe. The suspension was stirred at room temperature for 30 min before dilution with 300 mL CH_2Cl_2. The organics were washed with 3 x 50 mL brine. The aqueous layer was back-extracted with 2 x 100 mL CH_2Cl_2. The combined organics were dried over Na_2SO_4 and were filtered. The filtrate was concentrated via rotary evaporator to afford the crude product as a yellow/orange film. The crude was redissolved in 5 mL GPC grade DMF and the solution was centrifuged to remove any remaining insoluble salts. The supernatant was filtered via syringe filter and was purified via recycling prep-GPC with DMF to afford only mixed [4]catenane product.

The mixed fractions were then purified a second time via recycling prep-GPC with DMF to afford **[4]C** as a sticky orange film (0.0133 g, 11%) as a racemic mixture; see Fig. S1 for further explanation. ^{1}H NMR (500 MHz, $CDCl_3$): δ 8.54 – 8.40 (m), 8.38 (d, *J* = 8.6 Hz), 8.33 (d, *J* = 2.8 Hz), 8.30 (d, *J* = 2.9 Hz), 8.25 (dd, *J* = 7.7, 3.5 Hz), 8.18 (dd, *J* = 8.4, 2.5 Hz), 8.16 – 8.03 (m), 8.00 (dd, *J* = 8.5, 2.0 Hz), 7.98 – 7.86 (m), 7.83 (td, *J* = 7.8, 1.6 Hz), 7.74 – 7.69 (m), 7.67 (d, *J* = 2.7 Hz,), 7.64 (d, *J* = 10.7 Hz), 7.37 – 7.21 (m), 7.11 (d, *J* = 7.5 Hz), 7.08 – 6.97 (m), 6.96 (s), 6.87 – 6.68 (m), 5.67 – 5.52 (m), 5.38 – 5.32 (m), 4.27 – 4.17 (m), 4.16 – 4.09 (m), 4.09 – 4.03 (m), 4.03 – 3.94 (m), 3.94 – 3.57 (m), 2.56 – 2.27 (m). ^{13}C NMR (125 MHz, $CDCl_3$): δ_C 160.3, 160.2, 156.3, 155.55, 155.51, 155.48, 155.14, 155.12, 153.3, 153.20, 153.16, 153.10, 149.34, 149.30, 146.1, 137.84, 137.78, 137.4, 137.3, 136.9, 132.4, 129.9, 129.2, 129.09, 129.06, 129.0, 127.9, 127.68, 127.64, 126.8, 126.5, 125.7, 121.89, 121.86, 121.8, 119.56, 119.54, 119.4, 115.72, 115.65, 115.60, 115.03, 114.98, 114.96, 71.08, 71.06, 71.00, 70.97, 70.93, 70.91, 70.89, 70.07, 70.03, 70.02, 69.97, 69.87, 69.86, 69.75, 69.73, 68.2, 68.1, 68.01, 67.98, 67.7, 67.59, 67.57, 32.1, 30.1. HRMS-ESI: calculated for $C_{300}H_{312}N_{20}O_{60}$: *m*/z = 2579.6110 $[M + 2H]^{2+}$, 1720.0764 $[M + 3H]^{3+}$; Found: *m*/z = 2579.5995 $[M + 2H]^{2+}$, 1720.0672 $[M + 3H]^{3+}$. MALDI-TOF: calculated for $C_{300}H_{312}N_{20}O_{60}$: *m/z* = 5158.2 $[M + H]^{+}$; Found: 5158.9 $[M + H]^{+}$ (α-cyano-4-hydroxycinnamic acid matrix).

*i) [5]Catenane **([5]C)***

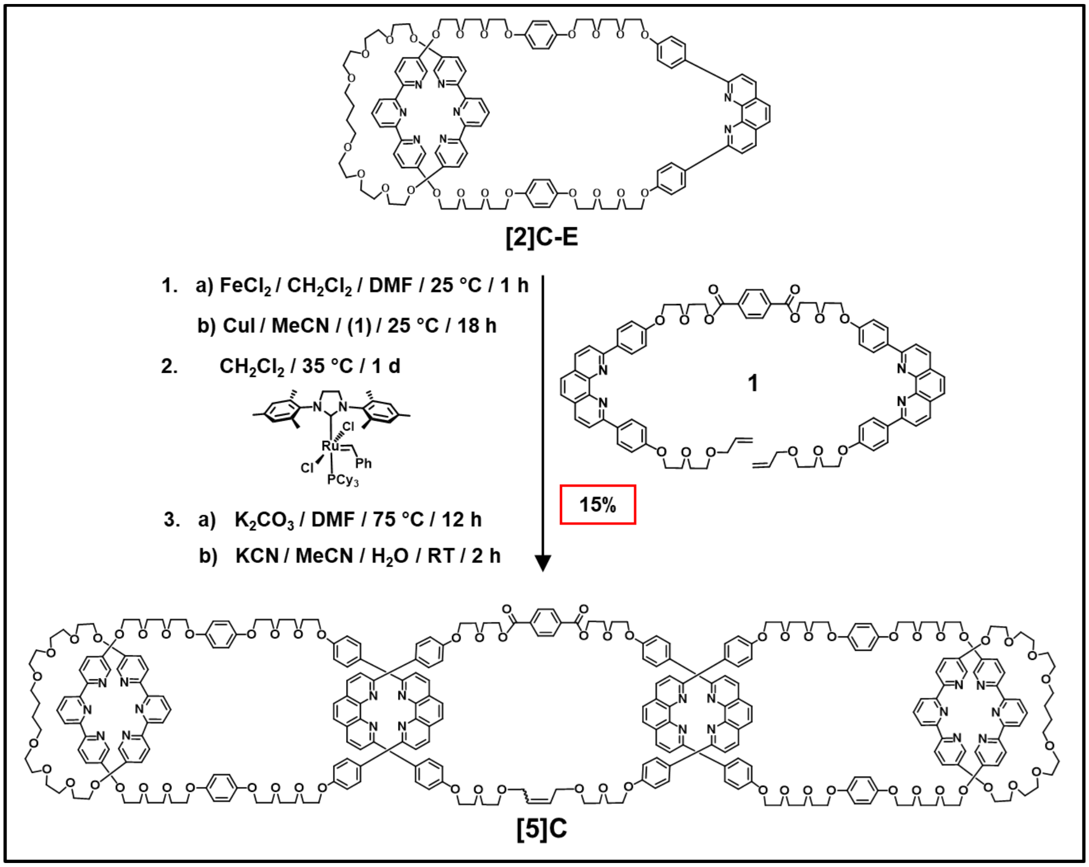

Scheme S20. Synthesis of **[5]C**.

<u>Step 1:</u> a) A solution of **[2]C-E** (0.100 g, 0.053 mmol, 5.0 eq.) was prepared in 5 mL anhydrous CH_2Cl_2 in a 25 mL RB flask. While stirring at 25°C under N_2, a solution of anhydrous $FeCl_2$ (0.007 g, 0.055 mmol, 5.0 eq.) in 1 mL anhydrous DMF was added via syringe. The solution immediately changed color from yellow to dark red. The solution was continued to stir under N_2 for 1 h. b) A solution of CuI (0.010 g, 0.053, 5.0 eq.) in 1 mL anhydrous MeCN was added via syringe and the reaction was continued to stir at 25°C. After 1 h, a solution of **1** (0.0138 g, 0.0106 mmol, 1.0 eq.) in 3 mL anhydrous CH_2Cl_2 was added via syringe. The dark red solution was then allowed to stir at 25°C under N_2 for 18 h. The solvent was removed via rotary evaporator and the crude was dried on high vacuum for several hours before ring-closing.

Step 2: The crude pre-[5]catenate complex was redissolved in 25 mL anhydrous CH_2Cl_2 in a 50 mL RB flask. A solution of Grubbs' 2nd generation catalyst (0.002 g, 0.0023 mmol, 0.2 eq.) was added and the RB flask was fitted with a Vigreux column. The reaction was heated to 35 °C while stirring under N_2. After 16 h, an additional 0.1 eq. Grubbs' 2nd generation catalyst was added and the reaction was continued to heat at 35 °C for 10. The reaction was quenched with EVE and the solvent was removed via rotary evaporator.

Step 3: a) The Fe^{2+} ion was first removed from the crude mixture with the addition of a weak inorganic base and moderate heating. The crude red film was redissolved in 15 mL DMF in a 100 mL RB flask. Solid K_2CO_3 (0.73 g, 5.28 mmol, 500 eq.) was added and the suspension was heated to 75°C for 12 h while stirring open to air. The solvent was removed via rotary evaporator. b) The Cu^+ then removed by addition of an excess of strongly competing cyanide ligand, in the form of KCN. The crude solid was taken up in 30 mL MeCN and the mixture was sonicated to form a suspension. While stirring open to air at room temperature, a solution of KCN (0.35 g, 5.28 mmol, 500 eq.) in 5 mL DI H_2O was added via syringe. The dark red solution was allowed to stir at 25°C for 2 h. The suspension was diluted with 300 mL CH_2Cl_2 and the organics were washed with 3 x 75 mL brine. The aqueous layer was back-extracted with 2 x 100 mL CH_2Cl_2. The combined organics were dried over Na_2SO_4 and were filtered. The solvent was removed via rotary evaporator to afford the crude as a dark reddish orange film. The crude was redissolved in 5 mL DMF and centrifuged in a 50 mL centrifuge tube to remove any remaining insoluble salts. The supernatant was filtered via syringe filter and the crude was purified via recycling prep-GPC with DMF over one injection to afford the product as a solid orange film (0.0073 g, 15%). ^{1}H NMR (500 MHz, $CDCl_3$): δ_H 8.46 (d, J = 8.7 Hz), 8.43 – 8.36 (m), 8.35 – 8.26 (m), 8.26 – 8.19 (m), 8.18 – 8.12 (m), 8.12 – 8.07 (m), 8.06 – 8.02 (m), 8.02 – 7.99 (m), 7.95 (t, J = 8.4 Hz), 7.79 (t, J = 7.8 Hz), 7.75 (t) , 7.71 (d, J = 6.5 Hz), 7.67 – 7.61 (m), 7.29 (dd, J = 8.8, 2.9 Hz), 7.24 (dd, J = 8.8, 2.9 Hz), 7.13 – 7.06 (m), 6.97 (d, J = 8.3 Hz), 6.74 (s), 5.83 (s), 5.75 – 5.68 (m), 4.52 – 4.47 (m,

J = 4.9 Hz), 4.42 – 4.36 (m), 4.26 – 4.20 (m), 4.19 – 4.15 (m), 4.12 – 4.07 (m), 4.07 – 3.88 (m), 3.80 – 3.67 (m), 3.67 – 3.52 (m), 3.52 – 3.45 (m), 3.40 (t, J = 5.1 Hz), 3.17 – 3.09 (m), 1.83 (s). ^{13}C NMR (125 MHz, $CDCl_3$): δ_C 165.9, 165.7, 163.9, 160.2, 160.1, 156.3, 156.2, 155.8, 155.5, 154.9, 153.1, 149.0, 146.0, 138.0, 137.8, 137.3, 137.2, 137.0, 136.9, 134.0, 133.9, 132.5, 132.4, 131.9, 129.8, 129.6, 129.1, 127.6, 125.8, 122.6, 122.0, 121.8, 119.5, 115.7, 115.6, 115.0, 114.9, 71.3, 71.1, 70.97, 70.93, 70.8, 70.6, 70.0, 69.9, 69.8, 69.7, 69.4, 68.3, 68.04, 67.99, 67.7, 67.5, 64.6, 29.8, 26.2. MALDI-TOF: calculated for $C_{288}H_{310}N_{20}O_{62}$: m/z = 5041.2 [M + H]$^+$; Found: 5042.0 [M + H]$^+$ (α-cyano-4-hydroxycinnamic acid matrix).

j) [6]Catenane ***([6]C)***

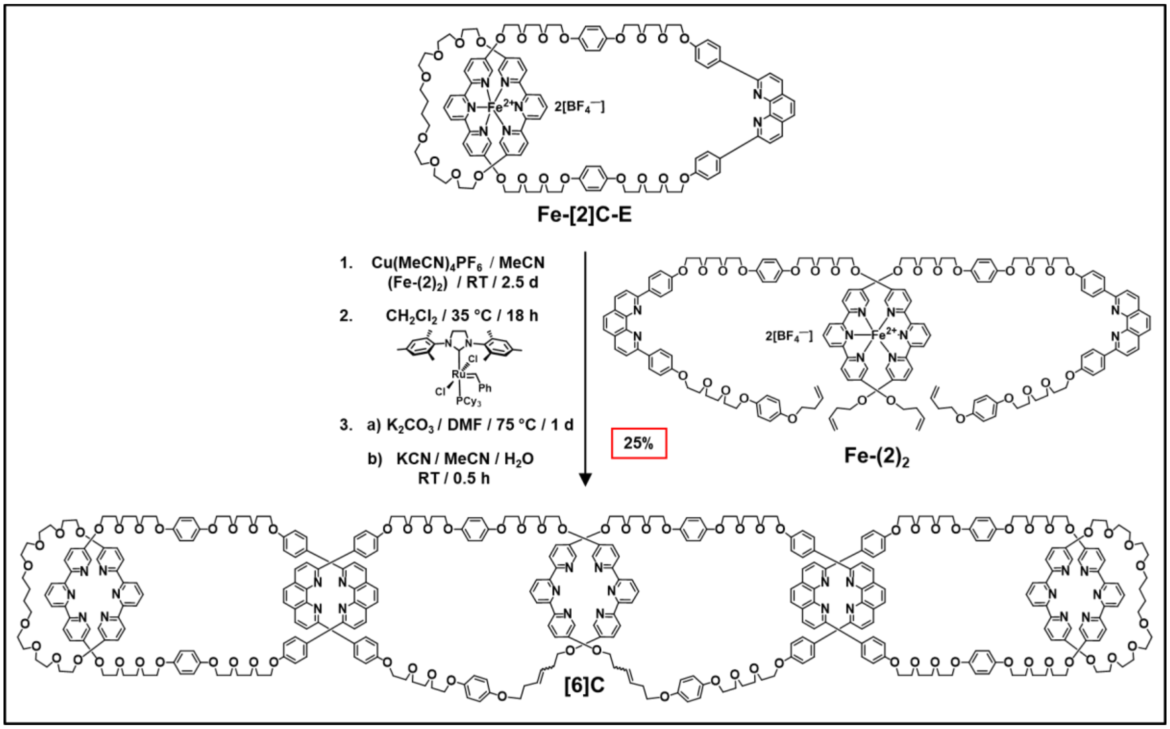

Scheme S21. Synthesis of **[6]C**.

<u>Step 1:</u> A solution of $Cu(MeCN)_4PF_6$ (0.0105 g, 0.028 mmol, 4.0 eq.) in 3 mL N_2-purged anhydrous MeCN was added via syringe to a solution of **Fe-[2]C-E** (0.0601 g, 0.0284 mmol, 4.0 eq.) in 10 mL N_2-purged anhydrous MeCN in an oven dried 50 mL RB flask. The dark red solution was stirred at room temperature under N_2. After 30 min, a solution of **Fe-(2)$_2$** (0.022 g, 0.0077 mmol,

1.0 eq.) in 7 mL N_2-purged anhydrous MeCN was added via syringe. The dark red solution continued to stir at room temperature under N_2 for 2.5 d. The solvent was then removed via rotary evaporator to afford the crude product as a foamy red solid, which was taken up in 100 mL CH_2Cl_2. The organic layer was washed with 3 x 50 mL DI H_2O and was dried over Na_2SO_4. The dark red solution was filtered, and the solvent was removed via rotary evaporator to afford the pre-[6]catenate complex as a dark red film, which was used in the next step without further purification.

Step 2: The crude foamy red solid was redissolved in 25 mL anhydrous CH_2Cl_2 in a 100 mL RB flask with a stir bar. A solution of Grubbs' 2nd generation catalyst (0.0013 g, 0.0015 mmol, 0.2 eq.) in 1 mL CH_2Cl_2 was added. The flask was fitted with a Vigreux column, and the dark red solution was heated to 35°C while stirring under N_2. After 18 h, an aliquot was quenched with EVE and the reaction was deemed complete by ^{1}H NMR. The remaining solution was quenched with 1 mL EVE and 5 mL MeCN. The solvent was removed via rotary evaporator to afford the crude [6]catenate mixture as a dark red film.

Step 3: a) The Fe^{2+} ion was first removed from the crude mixture with the addition of a weak inorganic base and moderate heating. The crude red film was redissolved in 25 mL DMF in a 100 mL RB flask. Solid K_2CO_3 (1.0 g, 7.23 mmol, 1000 eq.) was added and the suspension heated to 75°C for 1 d while stirring open to air. The solvent then removed via rotary evaporator and 50 mL MeCN was added to the crude suspension. b) The Cu^+ then removed by addition of an excess of strongly competing ligand, namely KCN. A solution of KCN (0.2 g, 3.07 mmol, 400 eq.) in 10 mL H_2O was added via syringe. The suspension was stirred at room temperature for 0.5 h before dilution with 300 mL CH_2Cl_2. The organics were washed with 3 x 100 mL DI H_2O. The organics were dried over Na_2SO_4 and were filtered. The solvent was removed via rotary evaporator and the crude was redissolved in 4 mL GPC grade DMF. The solution was filtered via syringe filter and was purified via recycling preparative GPC with DMF. The mixed fractions were repurified via

recycling preparative GPC with DMF. The linear [6]catenane **[6]C** was isolated as a yellow/orange film (0.0122 g, 25%) as a racemic mixture; see Fig. S1 for further explanation. ^{1}H NMR (500 MHz, $CDCl_3$): δ 8.50 – 8.44 (m), 8.36 (dd, *J* = 11.6, 8.7 Hz), 8.30 (ddd, *J* = 8.6, 6.8, 3.3 Hz), 8.27 – 8.22 (m), 8.18 (dd, *J* = 8.4, 2.8 Hz), 8.16 – 8.13 (m), 8.03 – 7.98 (m), 7.96 (d, *J* = 8.5 Hz), 7.85 – 7.73 (m), 7.67 (d, *J* = 2.8 Hz), 7.64 (d, *J* = 15.7 Hz), 7.33 – 7.27 (m), 7.26 – 7.17 (m), 7.11 (d, *J* = 8.2 Hz), 7.07 – 6.97 (m), 5.66 – 5.46 (m), 4.23 (s), 4.18 (t, *J* = 4.9 Hz), 4.16 – 4.03 (m), 4.03 – 3.88 (m), 3.87 – 3.53 (m), 3.50 (t, *J* = 5.2 Hz), 3.41 (dd, *J* = 5.6, 4.6 Hz), 3.14 (s), 2.55 – 2.34 (m). ^{13}C NMR (125 MHz, $CDCl_3$): δ_C 160.21, 160.19, 156.33, 156.31, 156.26, 155.7, 155.54, 155.49, 155.46, 155.19, 155.16, 155.12, 155.08, 155.04, 153.18, 153.15, 153.13, 153.12, 149.3, 149.20, 149.19, 146.14, 146.12, 138.5 137.8, 137.7, 137.4, 137.3, 136.9, 132.34, 132.30, 132.26, 129.2, 129.08, 129.05, 127.9, 127.6, 125.7, 122.5, 122.0, 121.86, 121.85, 121.7, 119.51, 119.46, 119.41, 119.1, 115.68, 115.63, 115.59, 115.58, 114.96, 114.91, 71.3, 71.1, 71.00, 70.96, 70.89, 70.83, 70.80, 70.66, 70.63, 70.01, 69.94, 69.91, 69.85, 69.80, 69.7, 68.21, 68.16, 68.07, 68.03, 67.99, 67.94, 67.91, 67.6, 67.5, 32.8, 32.6, 29.8, 26.3. HRMS-ESI: calculated for $C_{362}H_{394}N_{26}O_{76}$: *m*/z = 2109.2704 [*M* + 3H]$^{3+}$, 2130.2523 [*M* + 3Na]$^{3+}$; Found: *m*/z = 2109.2655 [*M* + 3H]$^{3+}$, 2130.2374 [*M* + 3Na]$^{3+}$. MALDI-TOF: calculated for $C_{362}H_{394}N_{26}O_{76}$: *m/z* = 6325.8 [*M* + H]$^{+}$; Found: 6325.3 [*M* + H]$^{+}$ (α-cyano-4-hydroxycinnamic acid matrix).

Section C. Spectroscopic Characterization

1) ^{1}H NMR Spectra

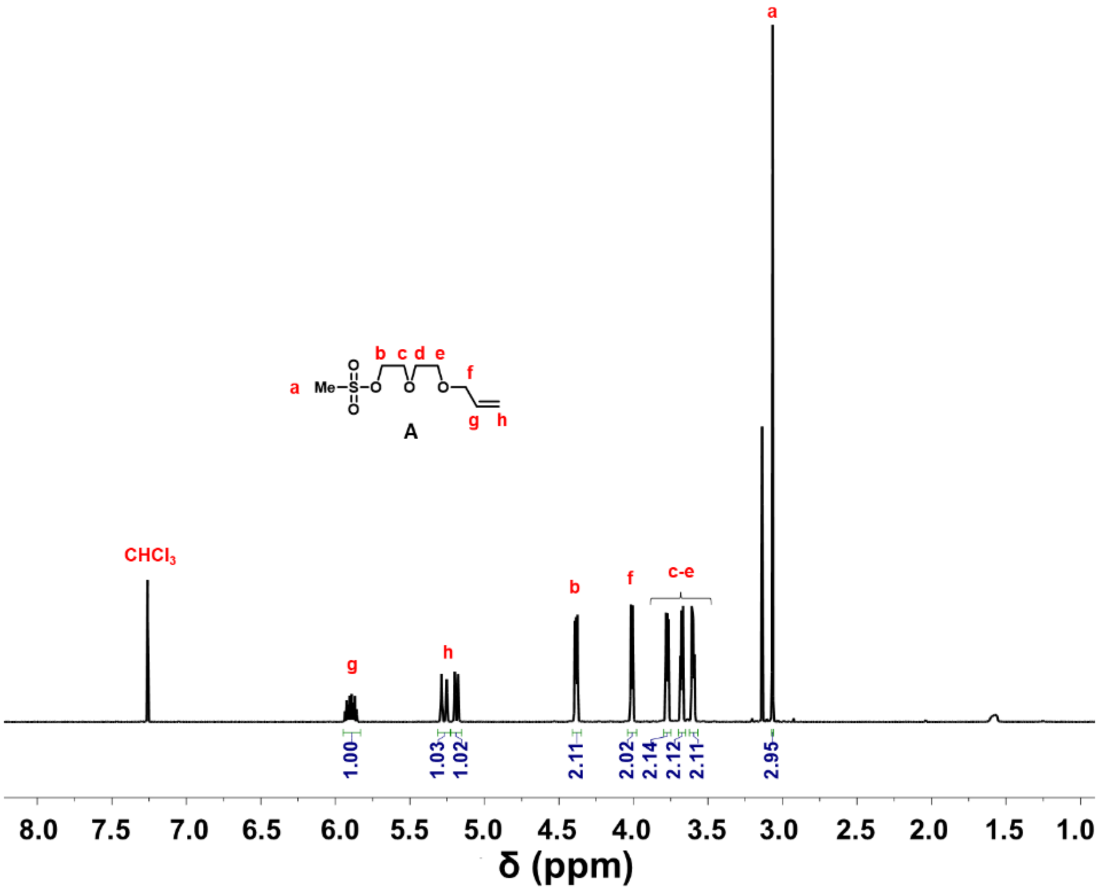

Figure S2. ^{1}H NMR spectrum (500 MHz, 25 °C, $CDCl_3$) of **A**.

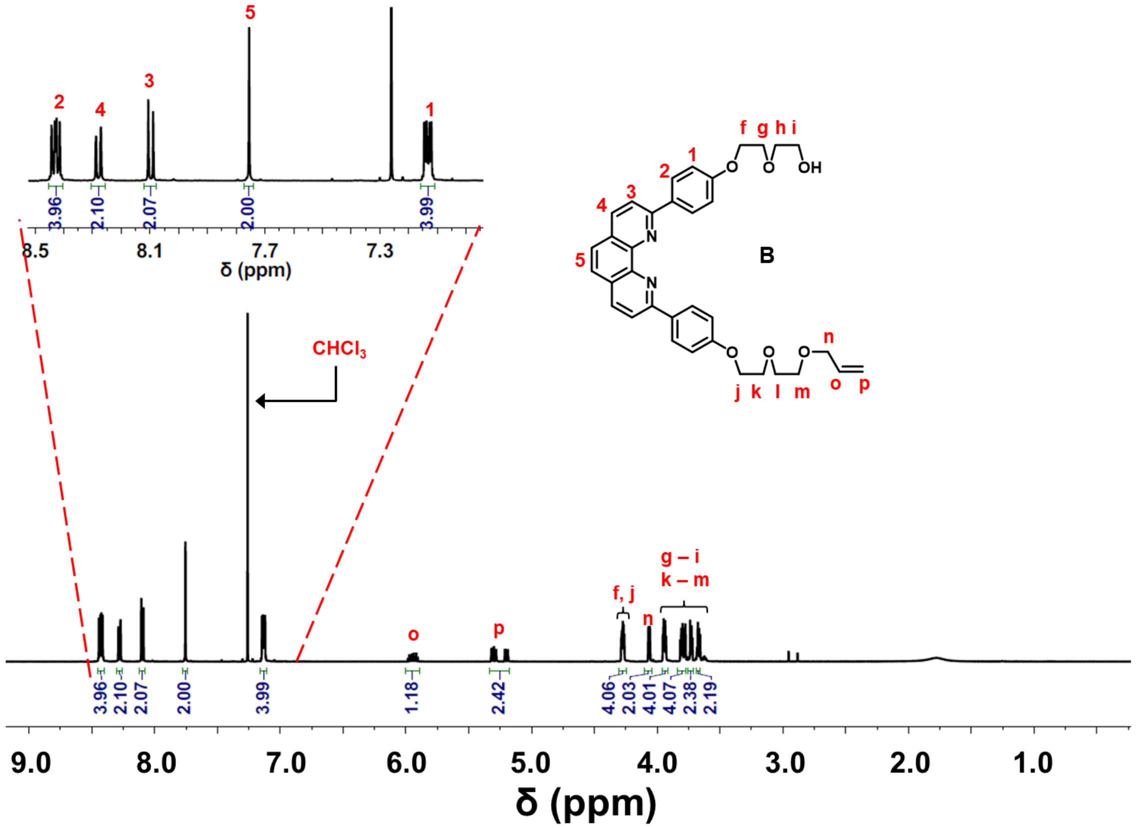

Figure S3. ^{1}H NMR spectrum (500 MHz, 25 °C, $CDCl_3$) of **B**.

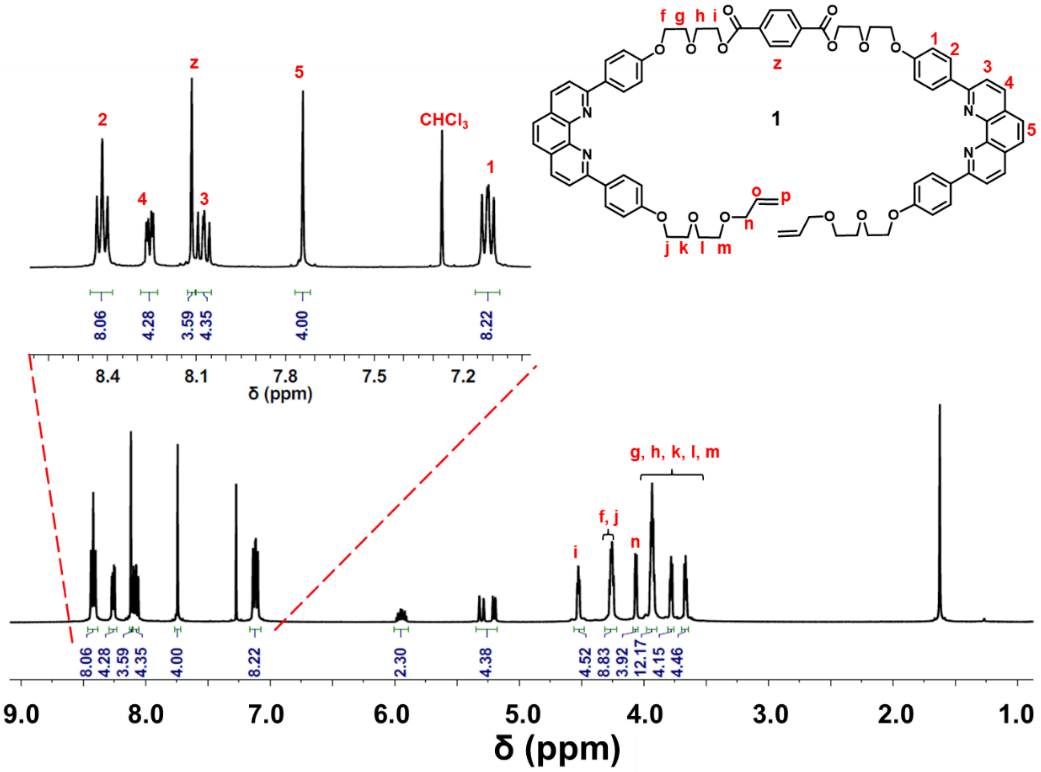

Figure S4. ^{1}H NMR spectrum (500 MHz, 25 °C, $CDCl_3$) of **1**.

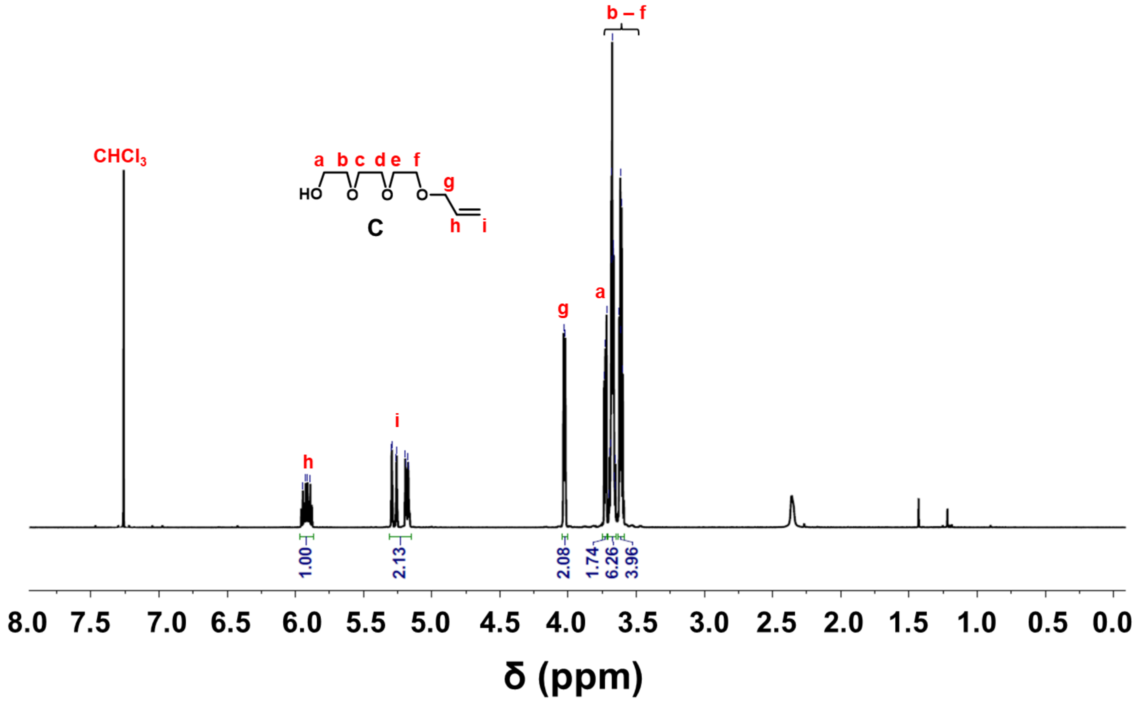

Figure S5. ^{1}H NMR spectrum (500 MHz, 25 °C, $CDCl_3$) of **C**.

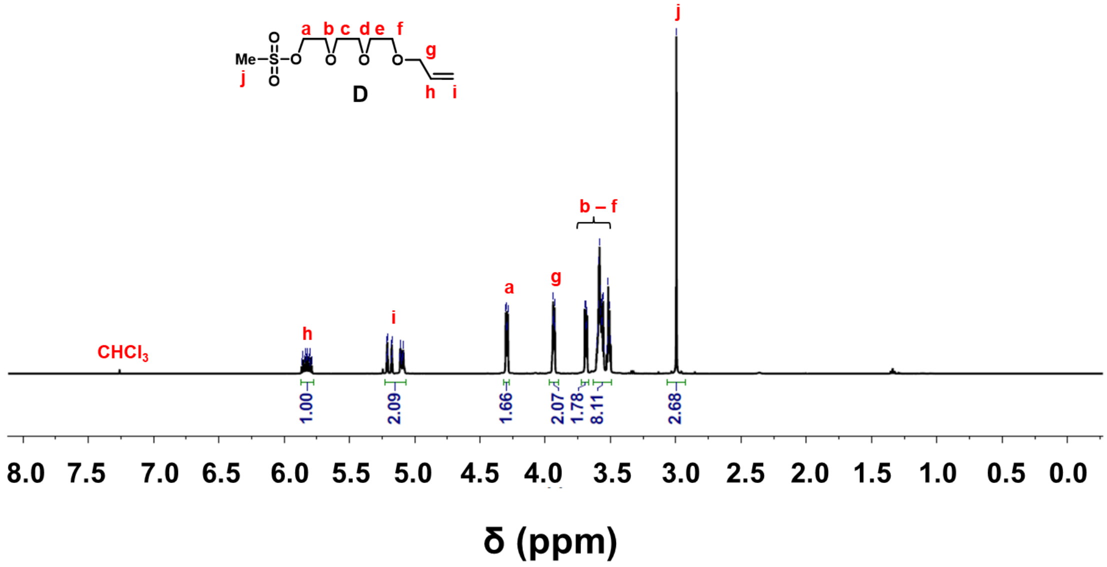

Figure S6. ^{1}H NMR spectrum (500 MHz, 25 °C, $CDCl_3$) of **D**.

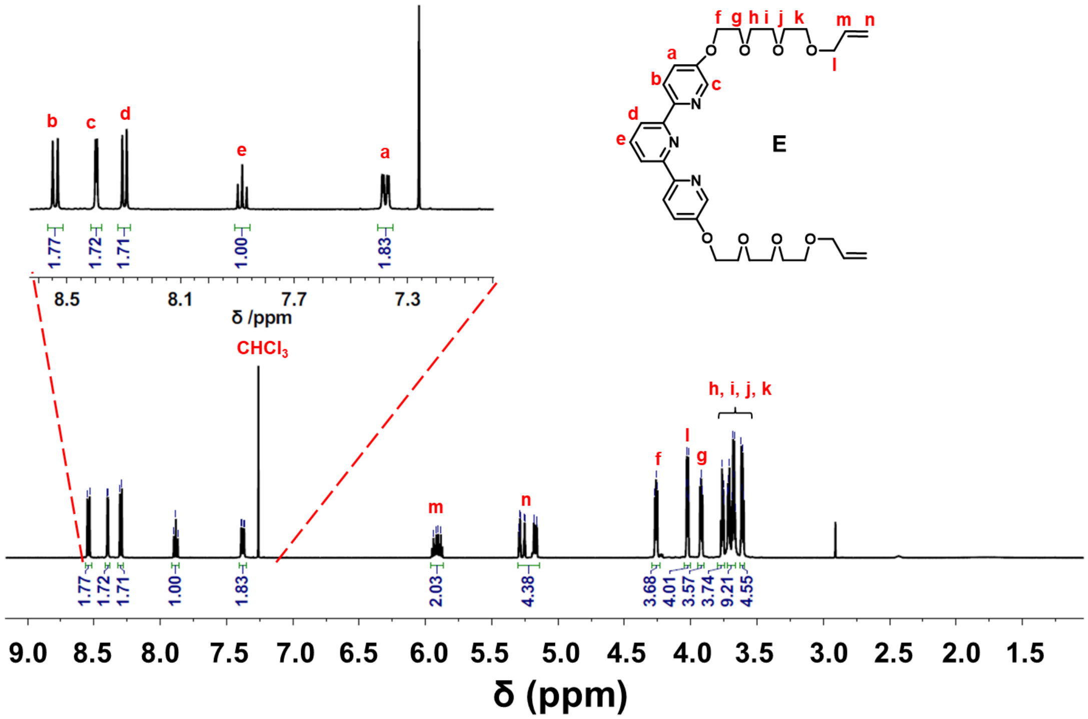

Figure S7. ^{1}H NMR spectrum (500 MHz, 25 °C, $CDCl_3$) of **E**.

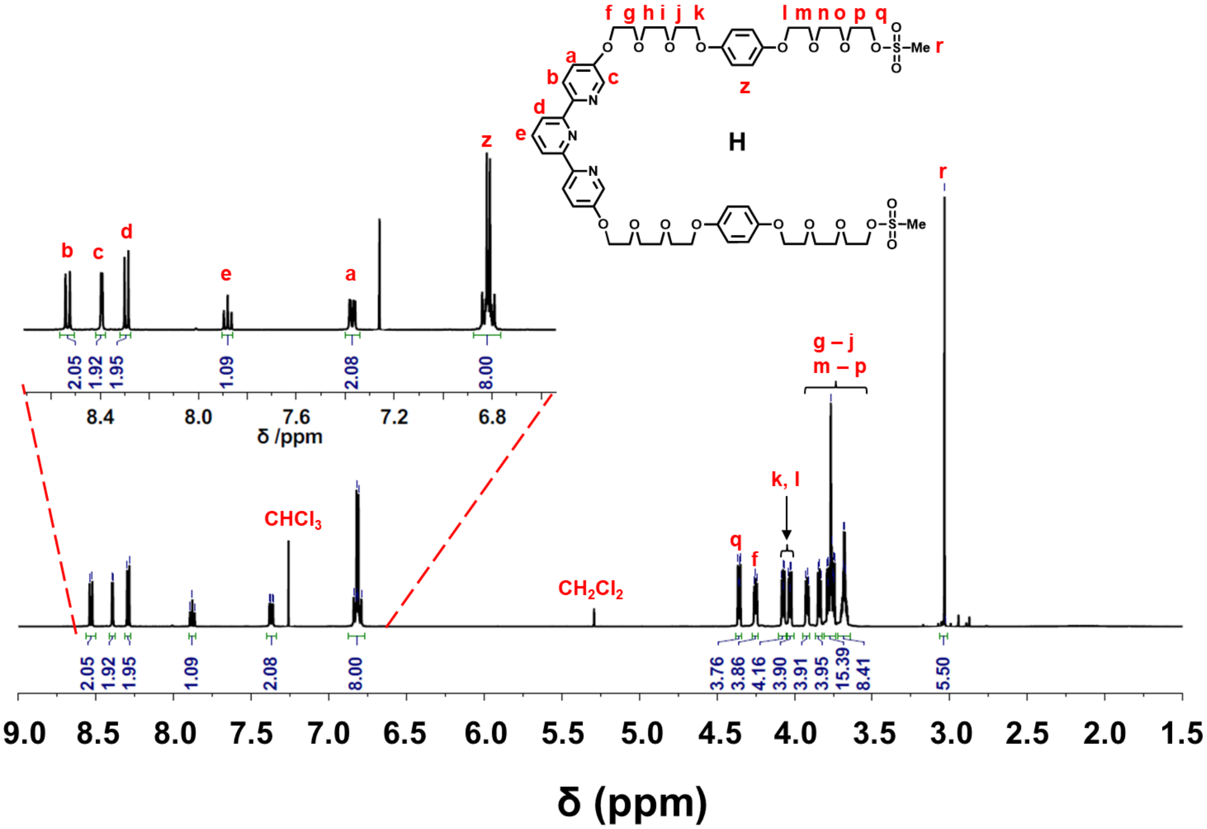

Figure S8. ^{1}H NMR spectrum (500 MHz, 25 °C, $CDCl_3$) of **H**.

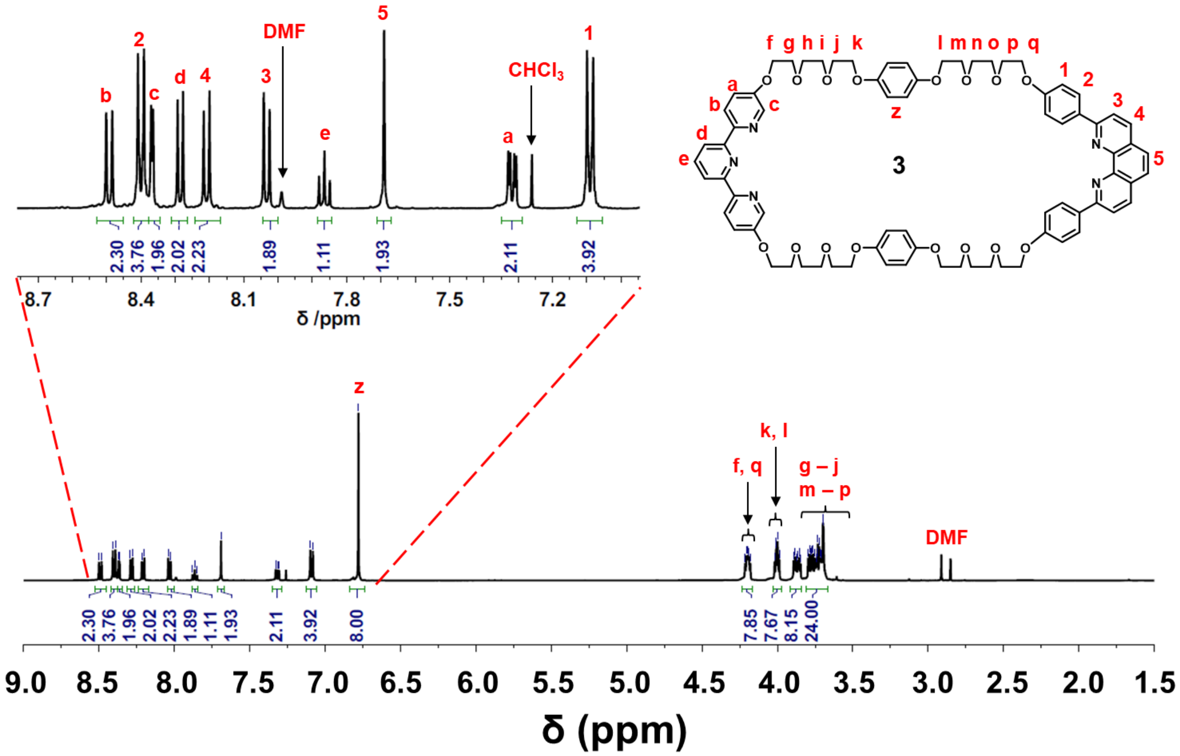

Figure S9. ^{1}H NMR spectrum (500 MHz, 25 °C, $CDCl_3$) of **3**.

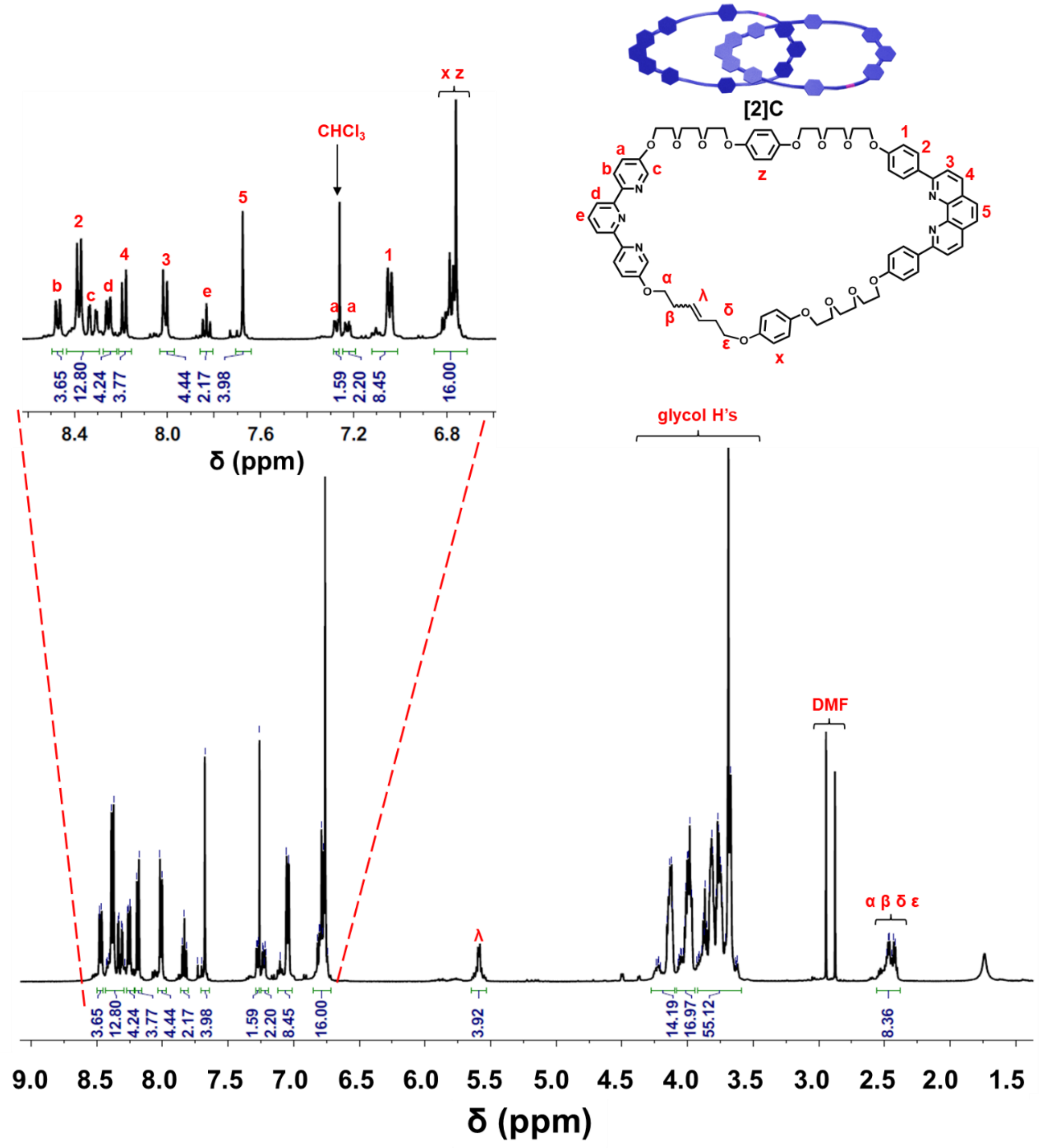

Figure S10. ^{1}H NMR spectrum (500 MHz, 25 °C, $CDCl_3$) of **[2]C**. Only one macrocycle is shown for clarity.

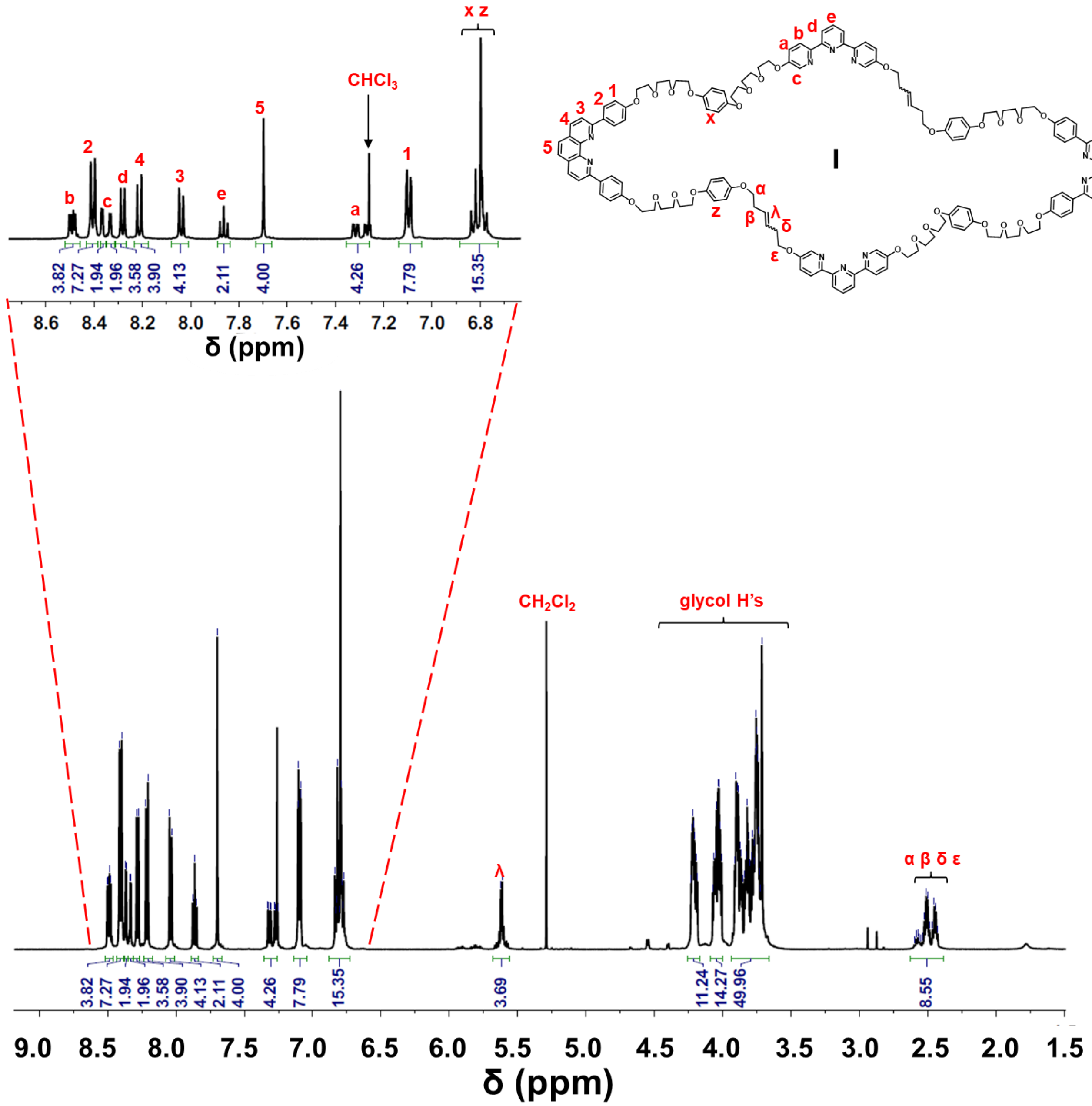

Figure S11. ^{1}H NMR spectrum (500 MHz, 25 °C, $CDCl_3$) of **I**.

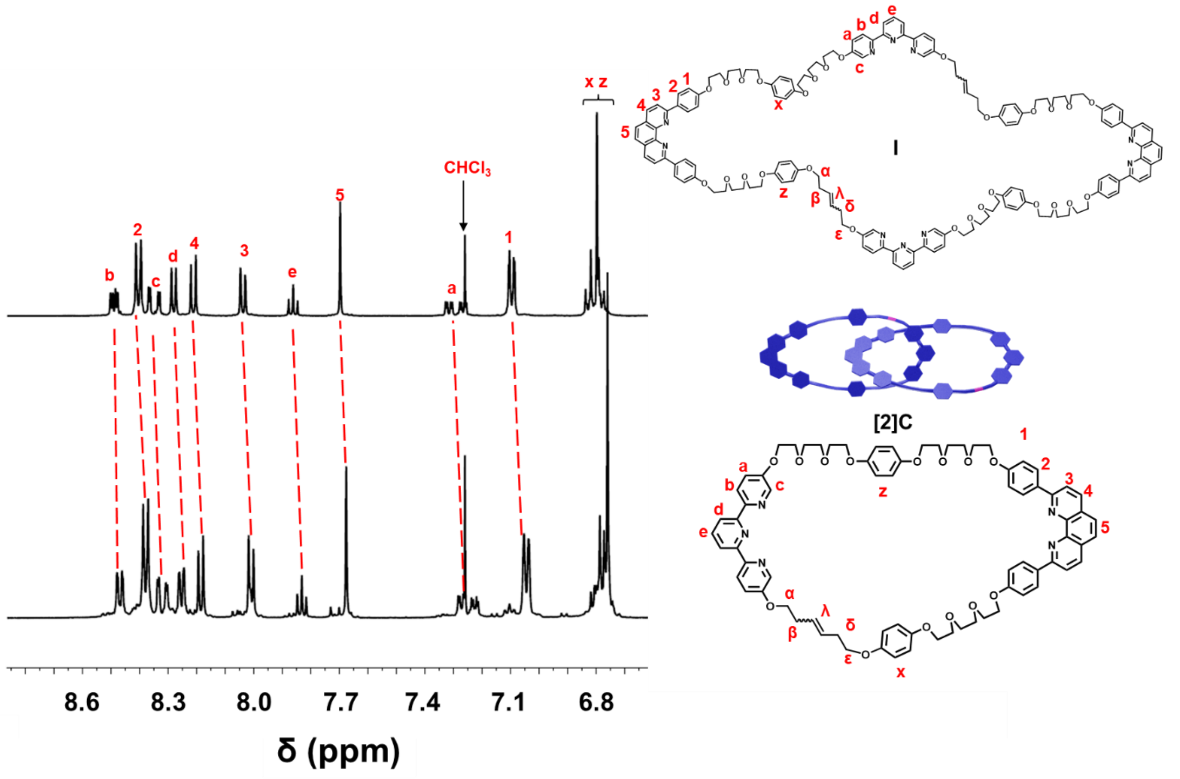

Figure S12. Stacked partial ^{1}H NMR spectra (500 MHz, 25 °C, $CDCl_3$) of the demetalated figure-eight TPM **I** and the [2]catenane TPM **[2]C**, where only a single macrocycle is shown for clarity.

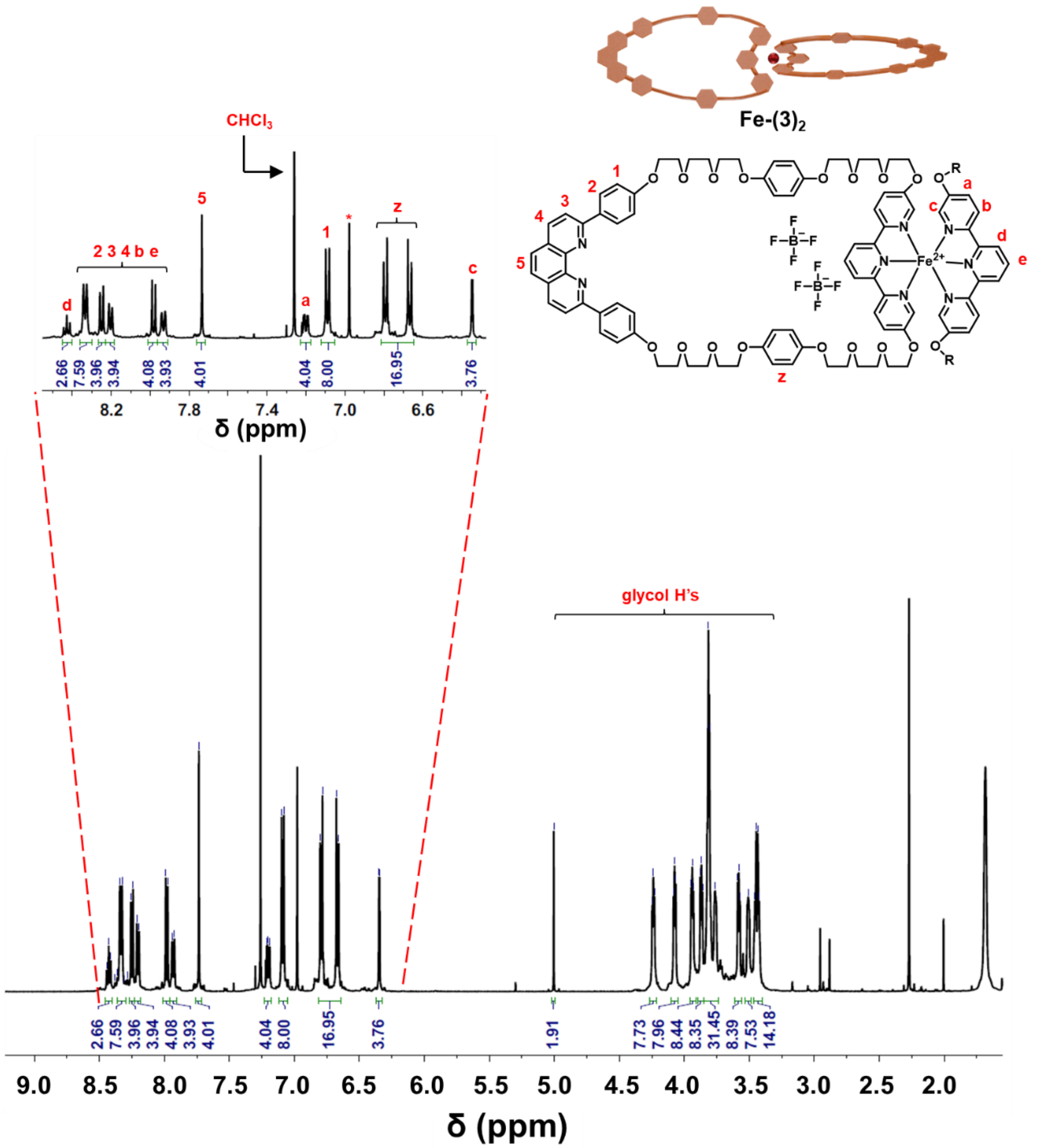

Figure S13. ^{1}H NMR spectrum (500 MHz, 25 °C, $CDCl_3$) of **Fe-(3)₂.** Single symmetric TPM macrocycle is shown for clarity (*unknown impurity).

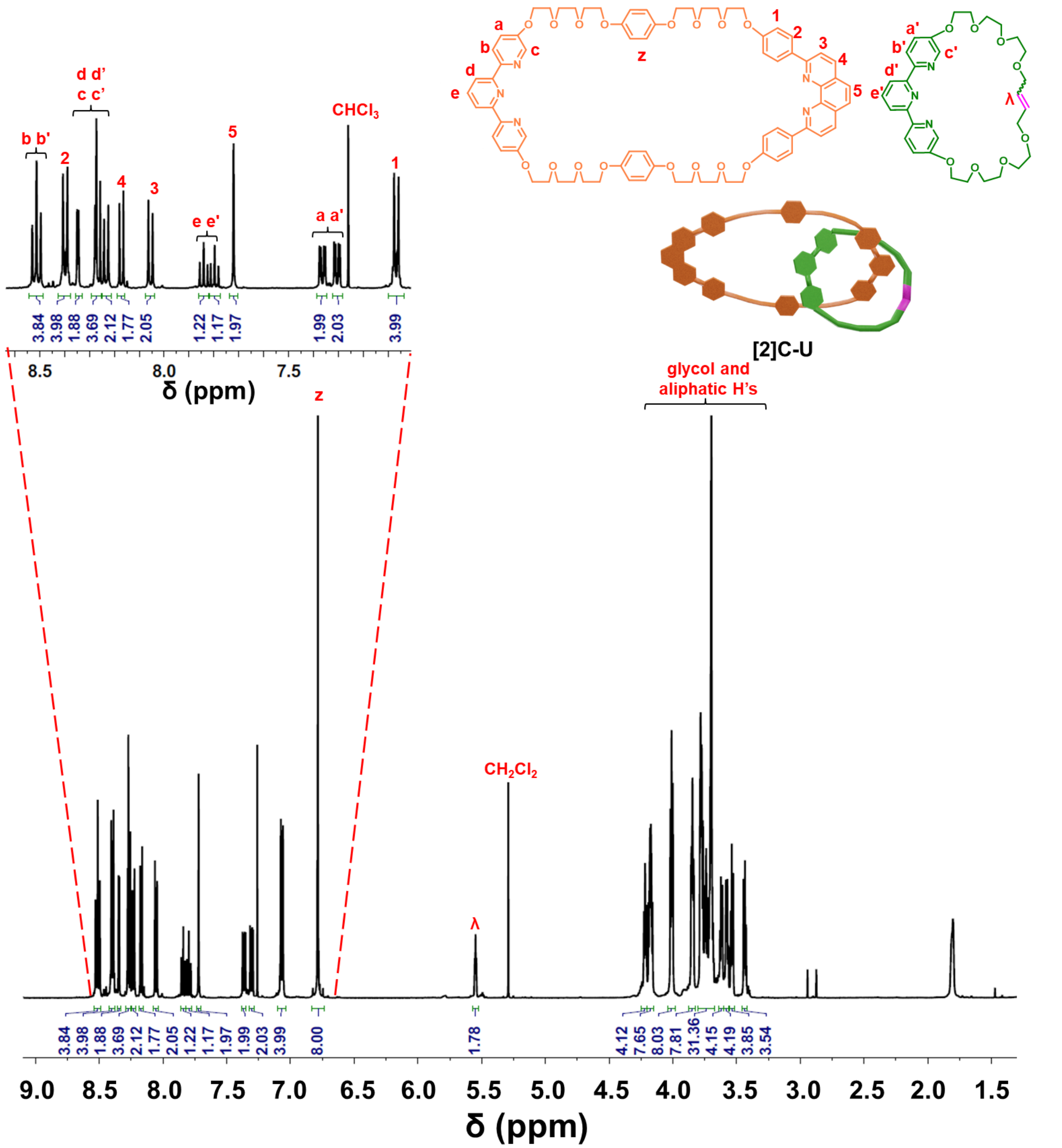

Figure S14. ^{1}H NMR spectrum (500 MHz, 25 °C, $CDCl_3$) of **[2]C-U**. Individual macrocycles shown for clarity.

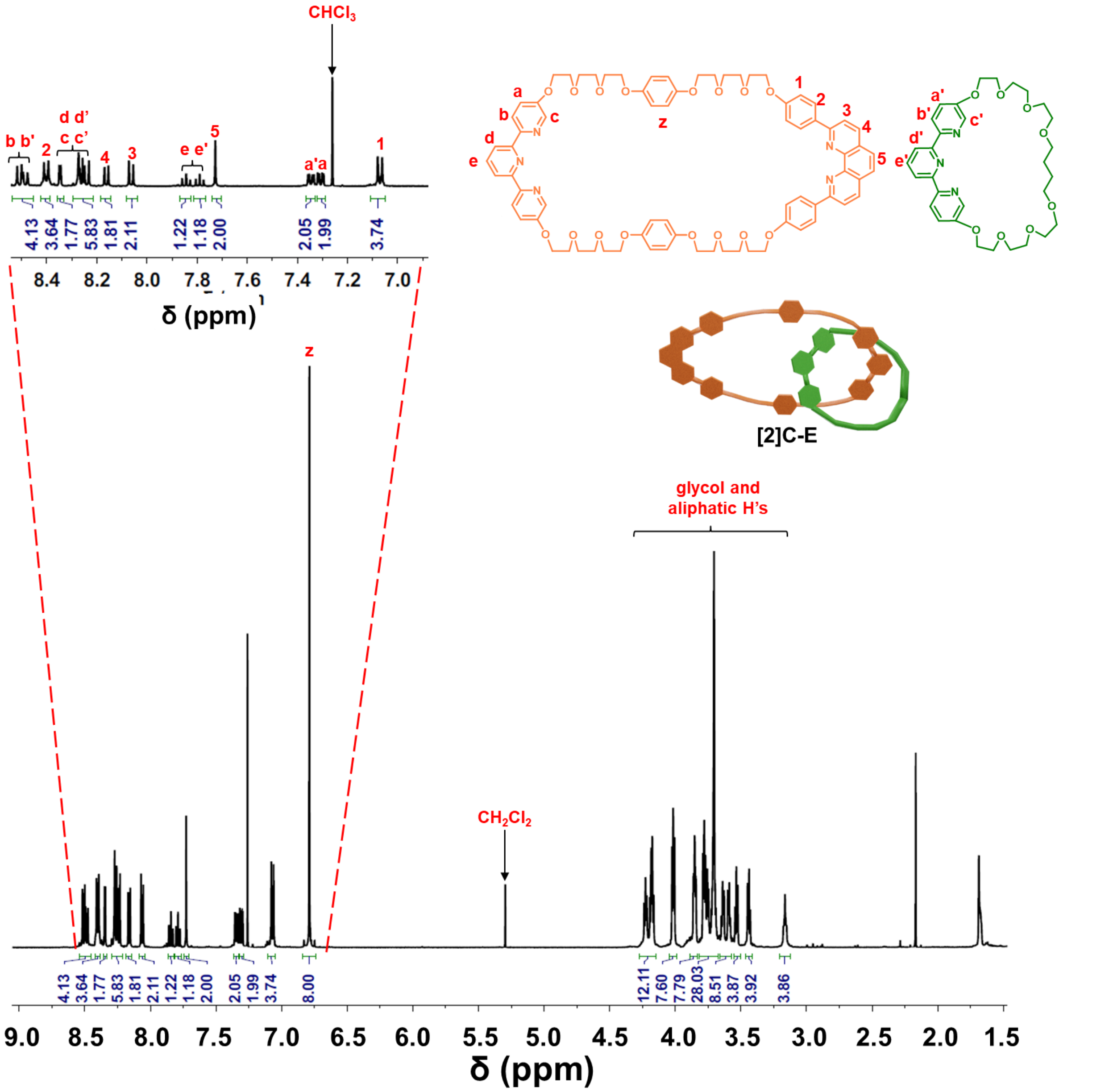

Figure S15. ^{1}H NMR spectrum (500 MHz, 25 °C, $CDCl_3$) of **[2]C-E**. Individual macrocycles shown for clarity.

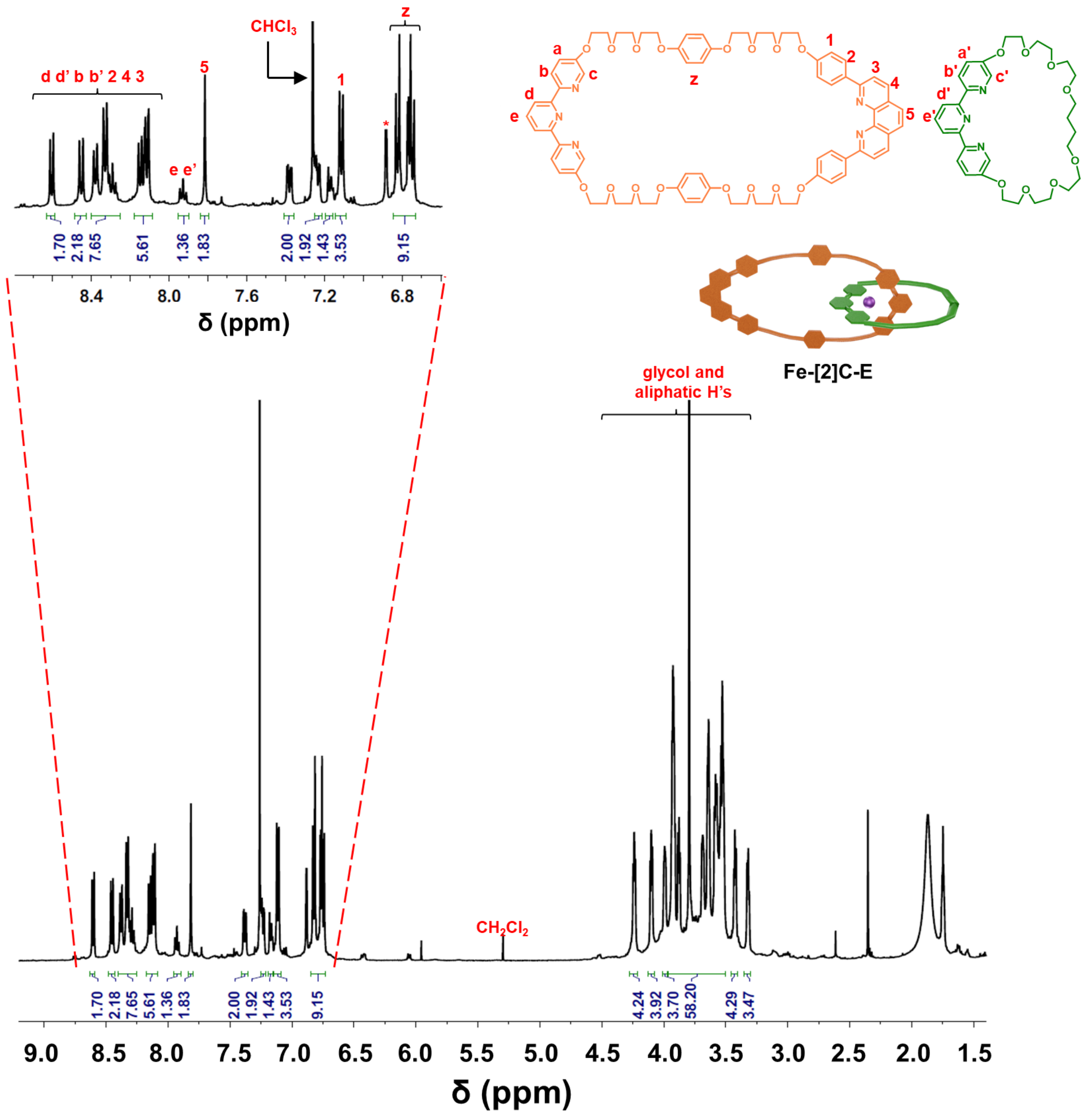

Figure S16. ^{1}H NMR (500 MHz, 25 °C, $CDCl_3$) spectrum of **Fe-[2]C-E**. Individual macrocycles shown for clarity.

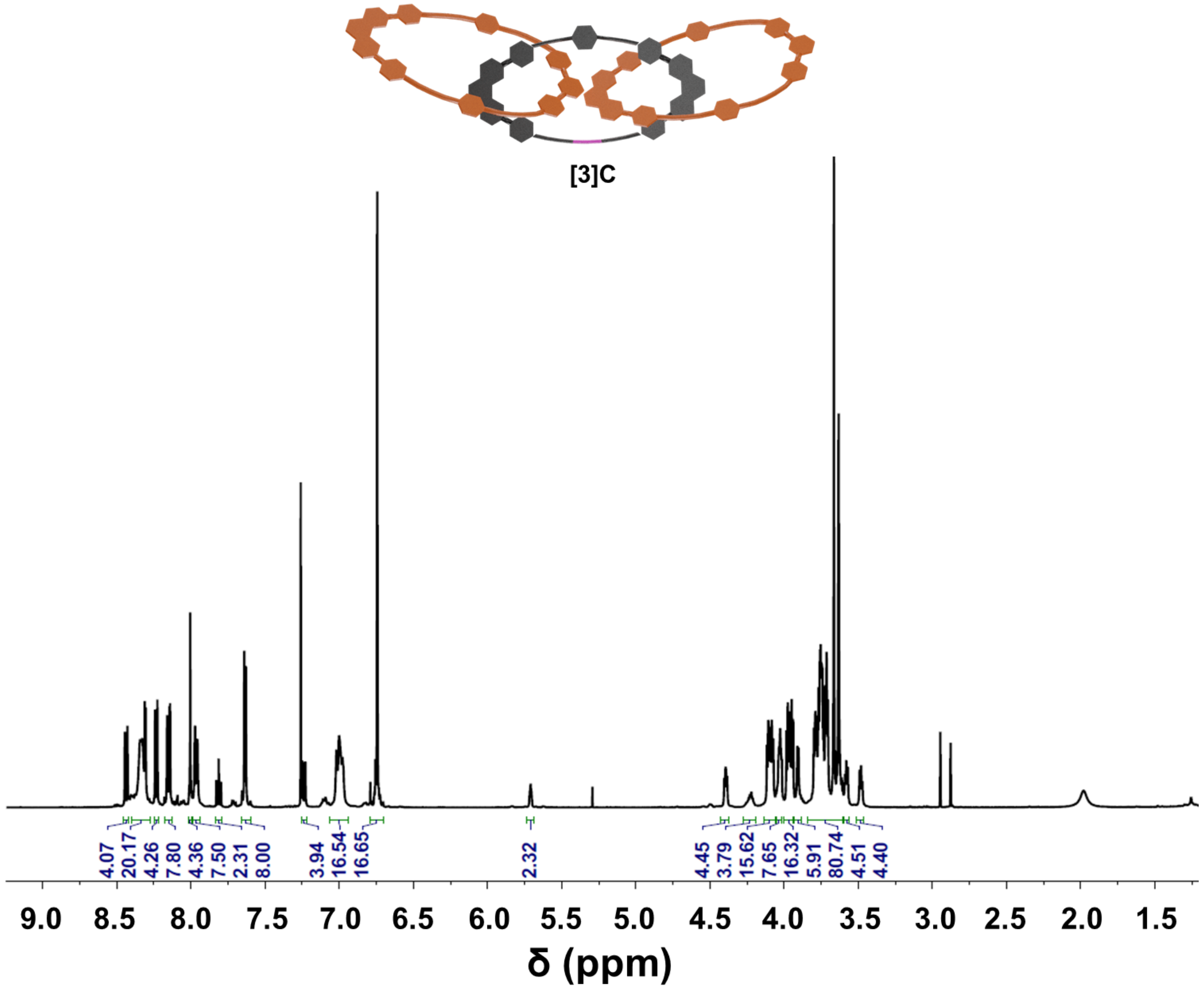

Figure S17. Full ^{1}H NMR (500 MHz, 25 °C, $CDCl_3$) full spectrum of **[3]C**. See Fig. S18 for zoomed-in spectrum of aromatic region.

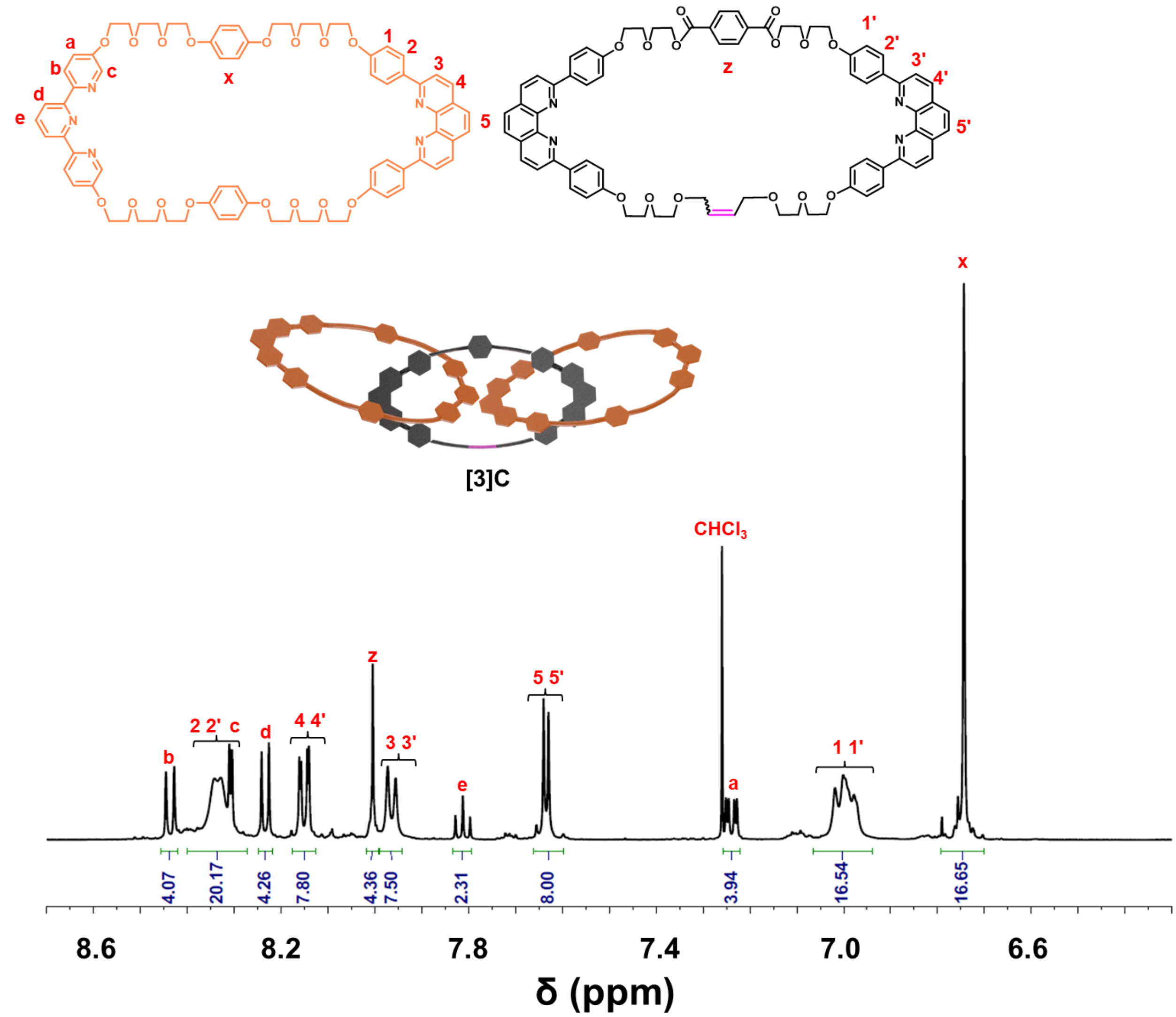

Figure S18. ^{1}H NMR (500 MHz, 25 °C, $CDCl_3$) spectrum showing the aromatic region for **[3]C.** Individual macrocycles shown for clarity.

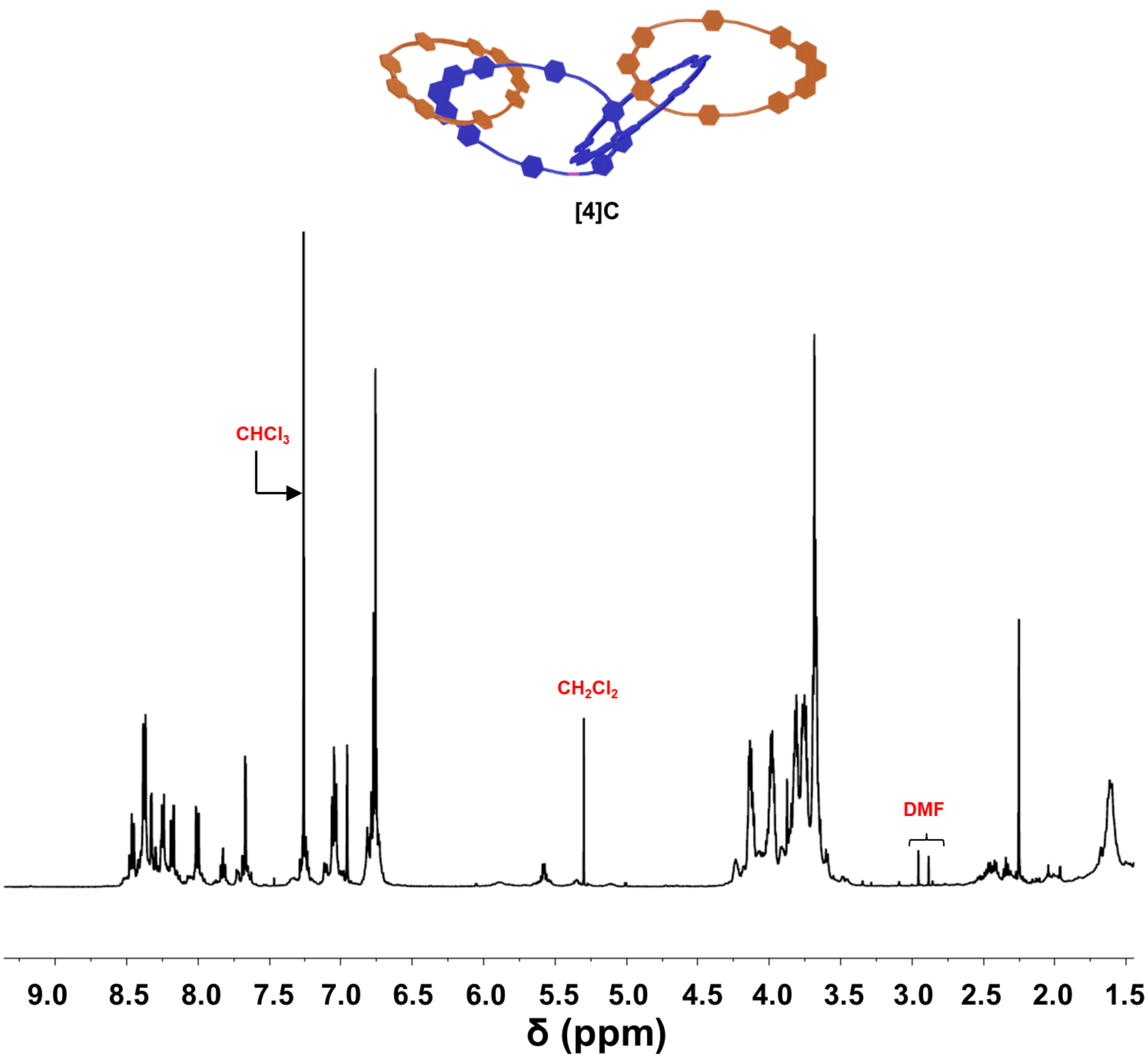

Figure S19. Full ^{1}H NMR (500 MHz, 25 °C, $CDCl_3$) full spectrum of **[4]C**. See Fig. S20 for zoomed-in spectrum of aromatic region.

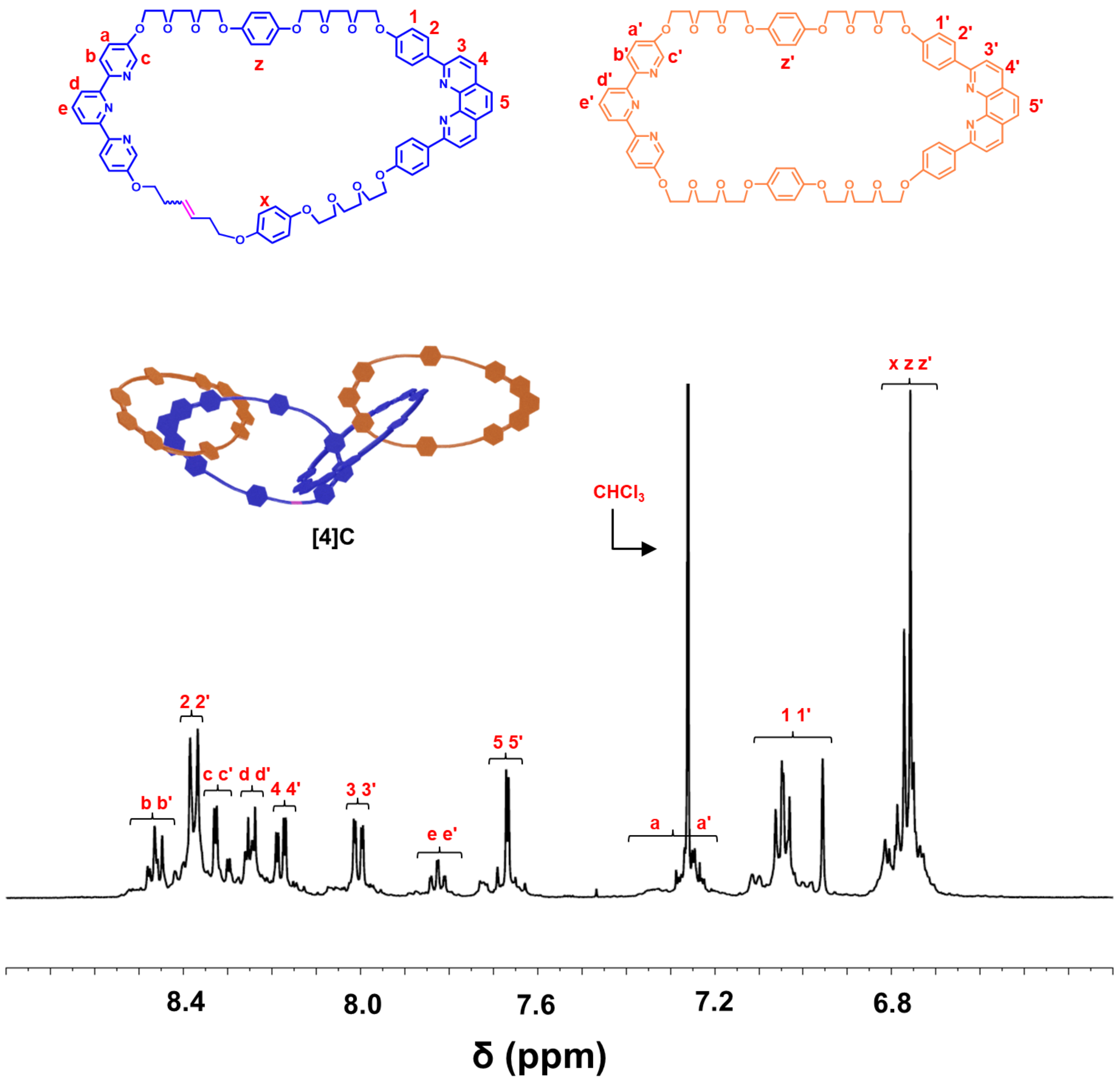

Figure S20. ^{1}H NMR (500 MHz, 25 °C, $CDCl_3$) spectrum showing the aromatic region for **[4]C.** Individual macrocycles shown for clarity.

The degrees of freedom afforded by the mechanical bonds lead to complex shielding and deshielding of protons, which cause apparent subpopulations of peaks and lead to difficulty in the integration of specific protons. However, these subpopulations are not due to lack of purity. The purity of **[4]C** was confirmed by recycling preparative GPC (Fig. S58), analytical GPC (Fig. S51), and analytical HPLC (Fig. S61), and the product identities confirmed by MALDI-TOF (Fig. S72).

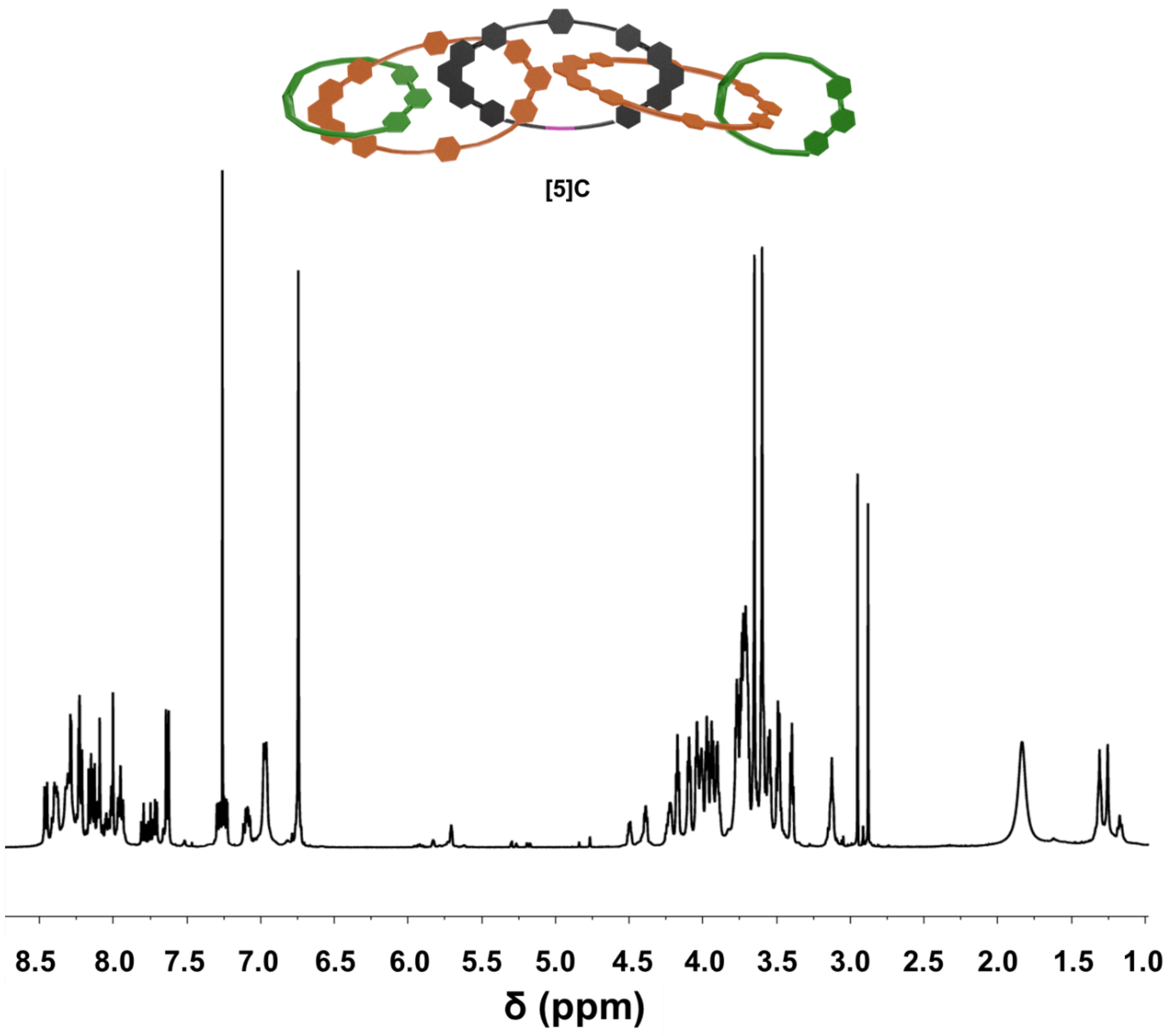

Figure S21. Full ^{1}H NMR (500 MHz, 25 °C, $CDCl_3$) full spectrum of **[5]C**. See Fig. S22 for zoomed-in spectrum of aromatic region.

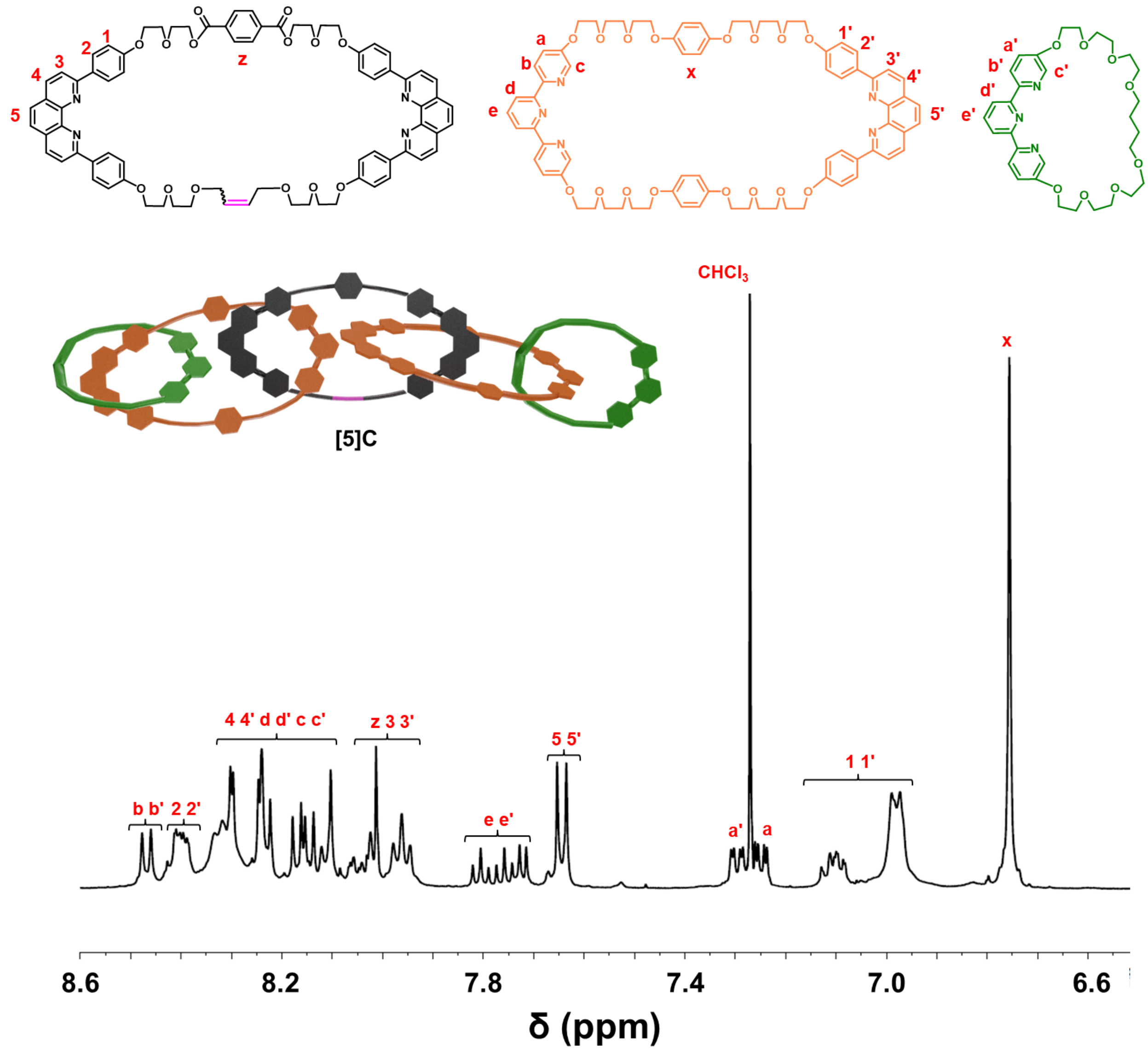

Figure S22. ^{1}H NMR (500 MHz, 25 °C, $CDCl_3$) spectrum showing the aromatic region for **[5]C.** Individual macrocycles shown for clarity.

Similarly observed for the ^{1}H NMR spectra of **[4]C** (Figs. S19-S20) and **[6]C** (Figs. S23-S24), The degrees of freedom afforded by the mechanical bonds lead to complex shielding and deshielding of protons, which cause apparent subpopulations of peaks and lead to difficulty in the integration of specific protons. These subpopulations are not due to lack of purity. The purity of **[5]C** was confirmed by recycling preparative GPC (Fig. S59), analytical GPC (Fig. S51), analytical HPLC (Fig. S61), and MALDI-TOF (Fig. S73).

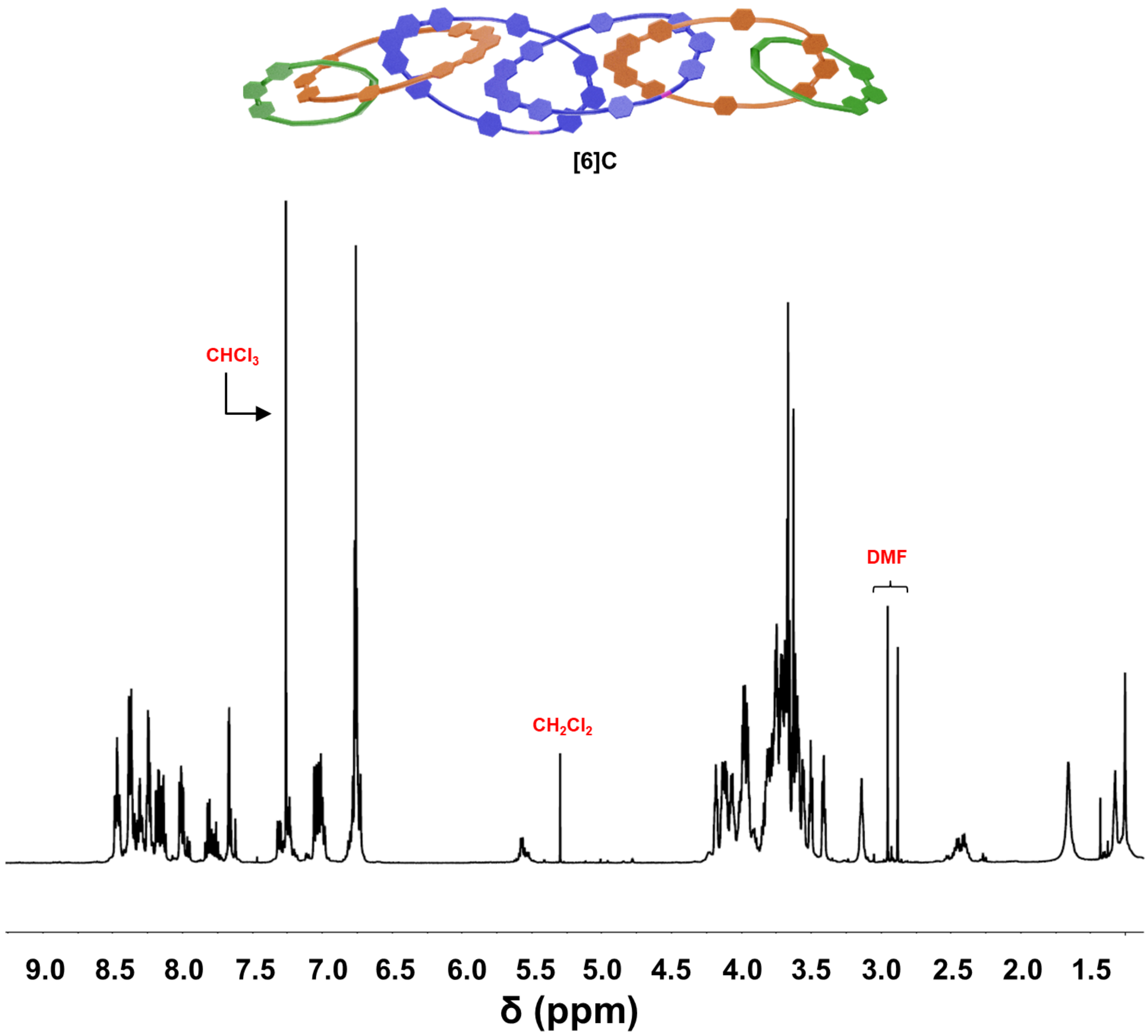

Figure S23. Full ^{1}H NMR (500 MHz, 25 °C, $CDCl_3$) full spectrum of **[6]C**. See Fig. S24 for zoomed-in spectrum of aromatic region.

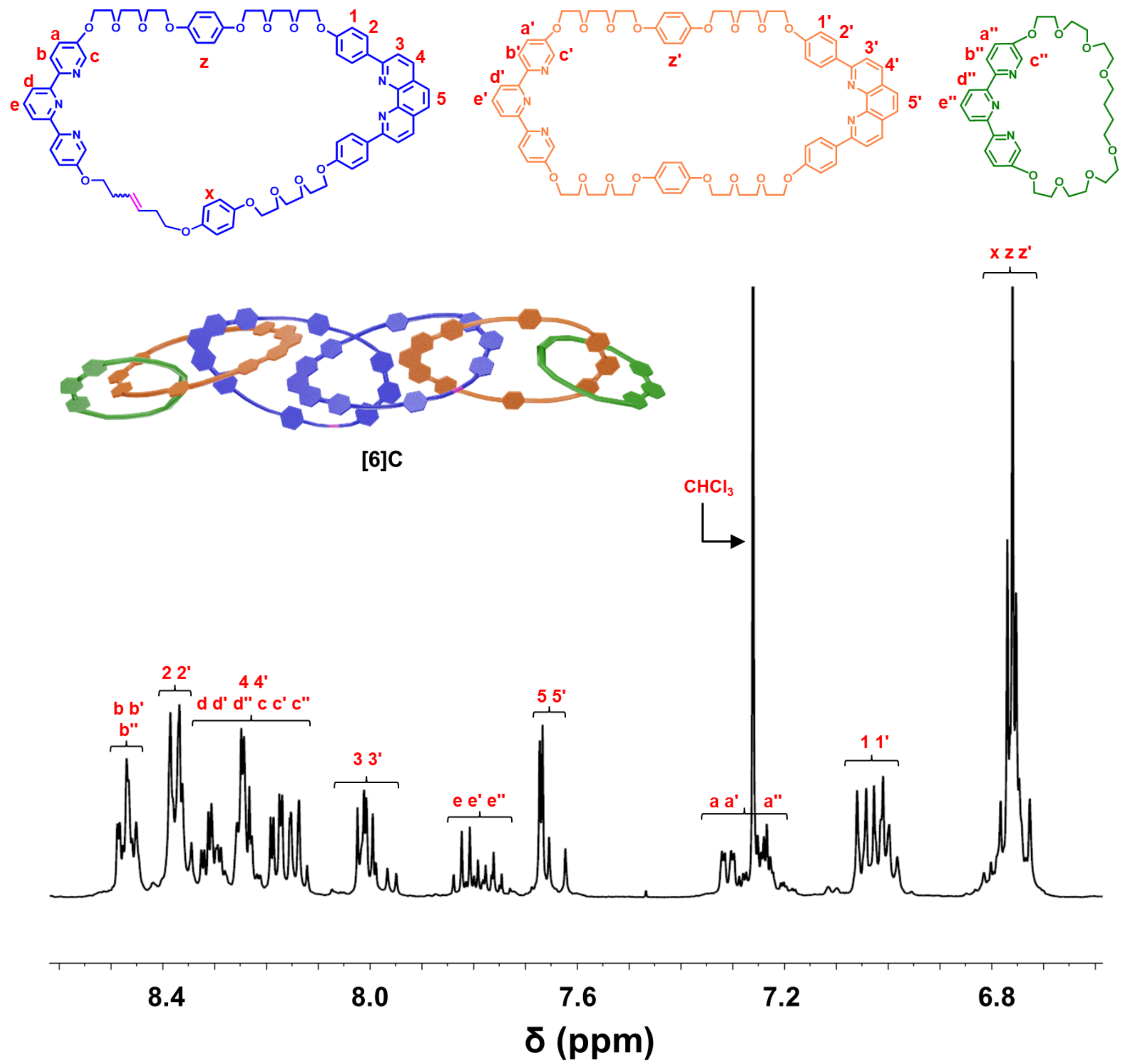

Figure S24. ^{1}H NMR (500 MHz, 25 °C, $CDCl_3$) spectrum showing the aromatic region for **[6]C.** Individual macrocycles shown for clarity.

Similarly observed for the ^{1}H NMR spectra of **[4]C** and **[5]C** (Figs. S19-S22), the degrees of freedom afforded by the mechanical bonds in **[6]C** lead to complex shielding and deshielding of protons, which cause apparent subpopulations of peaks and lead to difficulty in the integration of specific protons. These subpopulations are not due to lack of purity. The purity of **[6]C** was confirmed by recycling preparative GPC (Fig. S60), analytical GPC (Fig. S51), analytical HPLC (Fig. S61), and MALDI-TOF (Fig.S74).

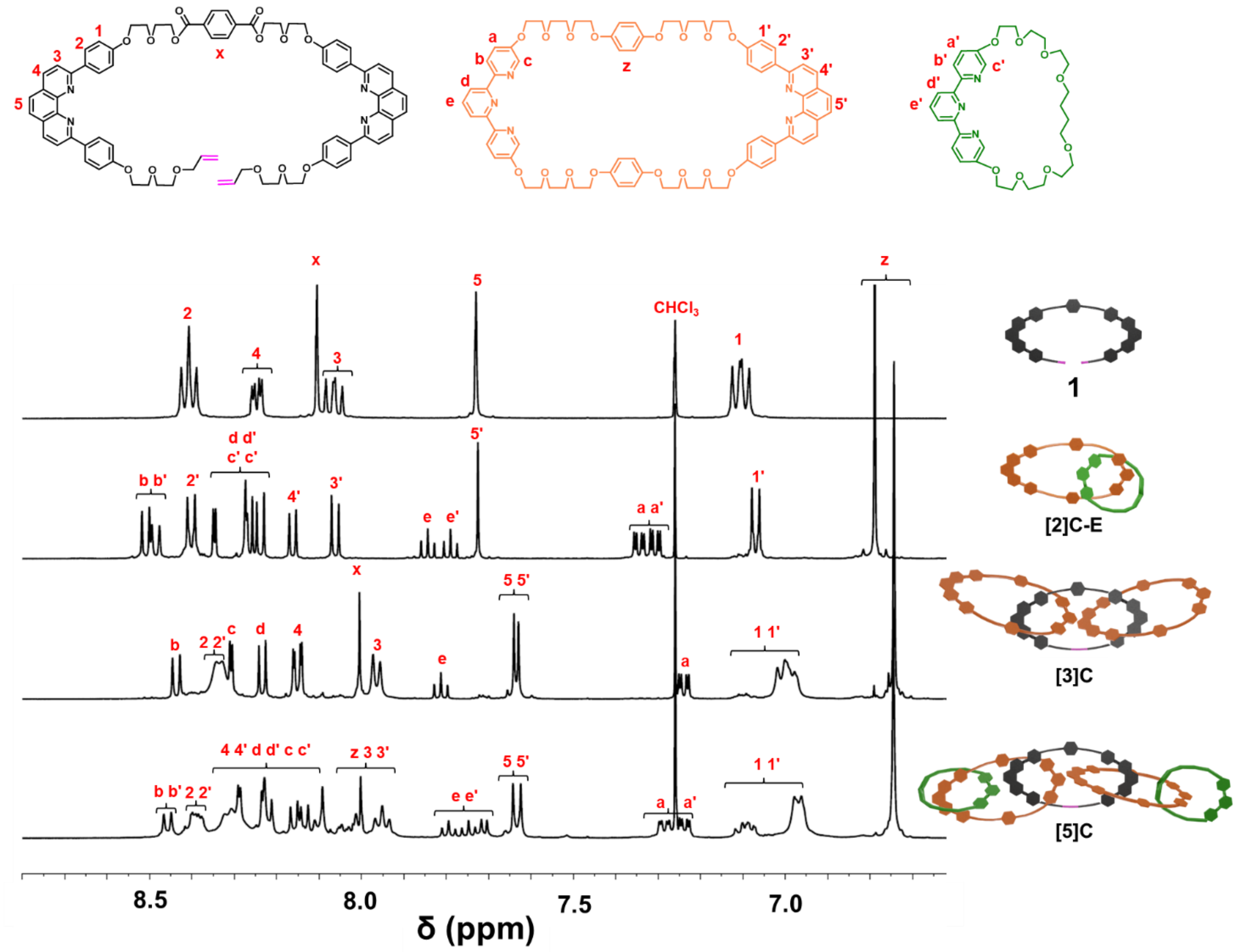

Figure S25. Stacked ^{1}H NMR (500 MHz, 25 °C, $CDCl_3$) spectra showing the aromatic region of for **1**, **[2]C-E**, **[3]C**, and **[5]C.** Individual macrocycles shown for clarity.

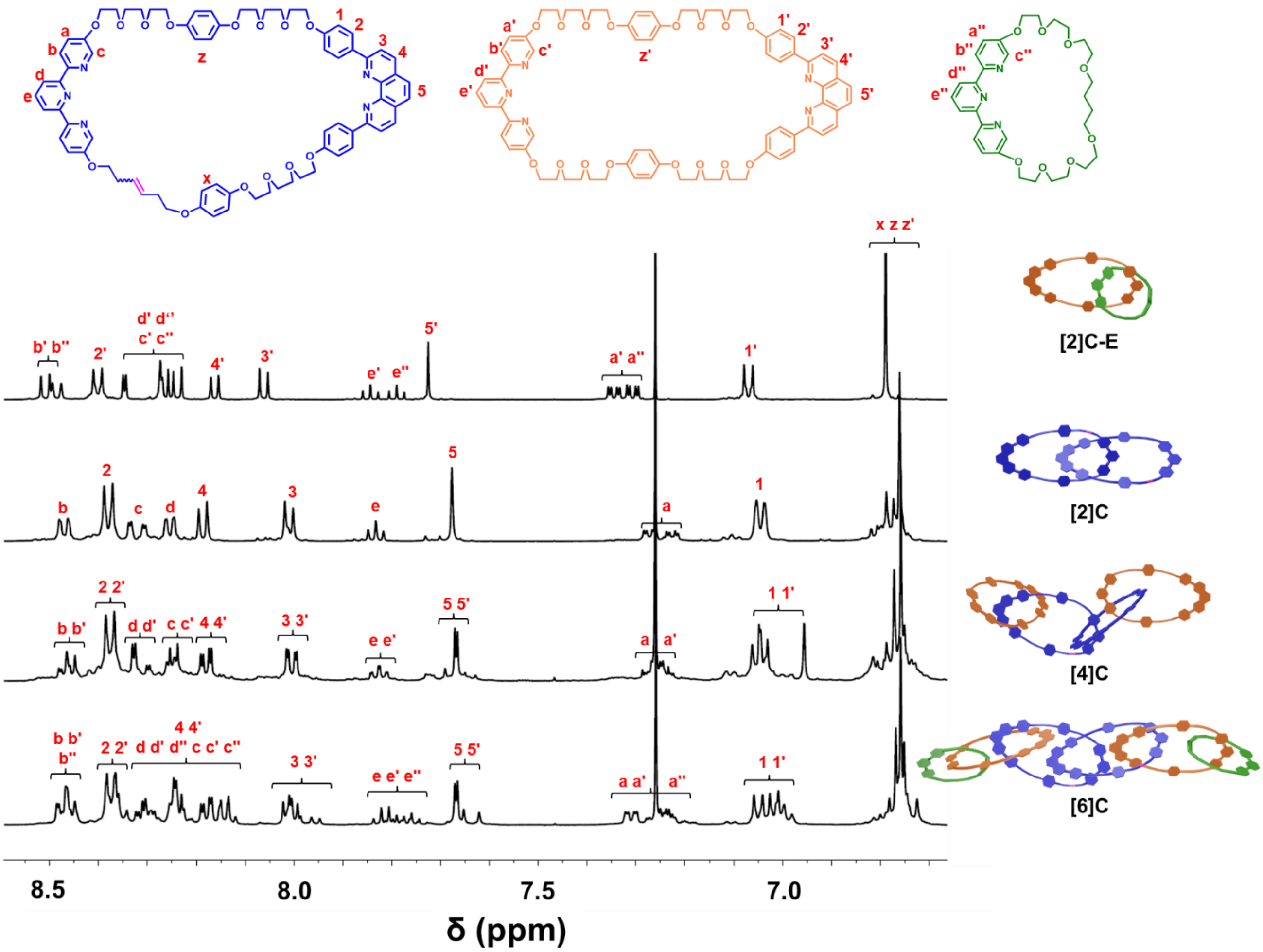

Figure S26. Stacked ¹H NMR (500 MHz, 25 °C, $CDCl_3$) spectra showing the aromatic region of for **[2]C-E**, **[2]C**, **[4]C**, and **[6]C.** Individual macrocycles shown for clarity.

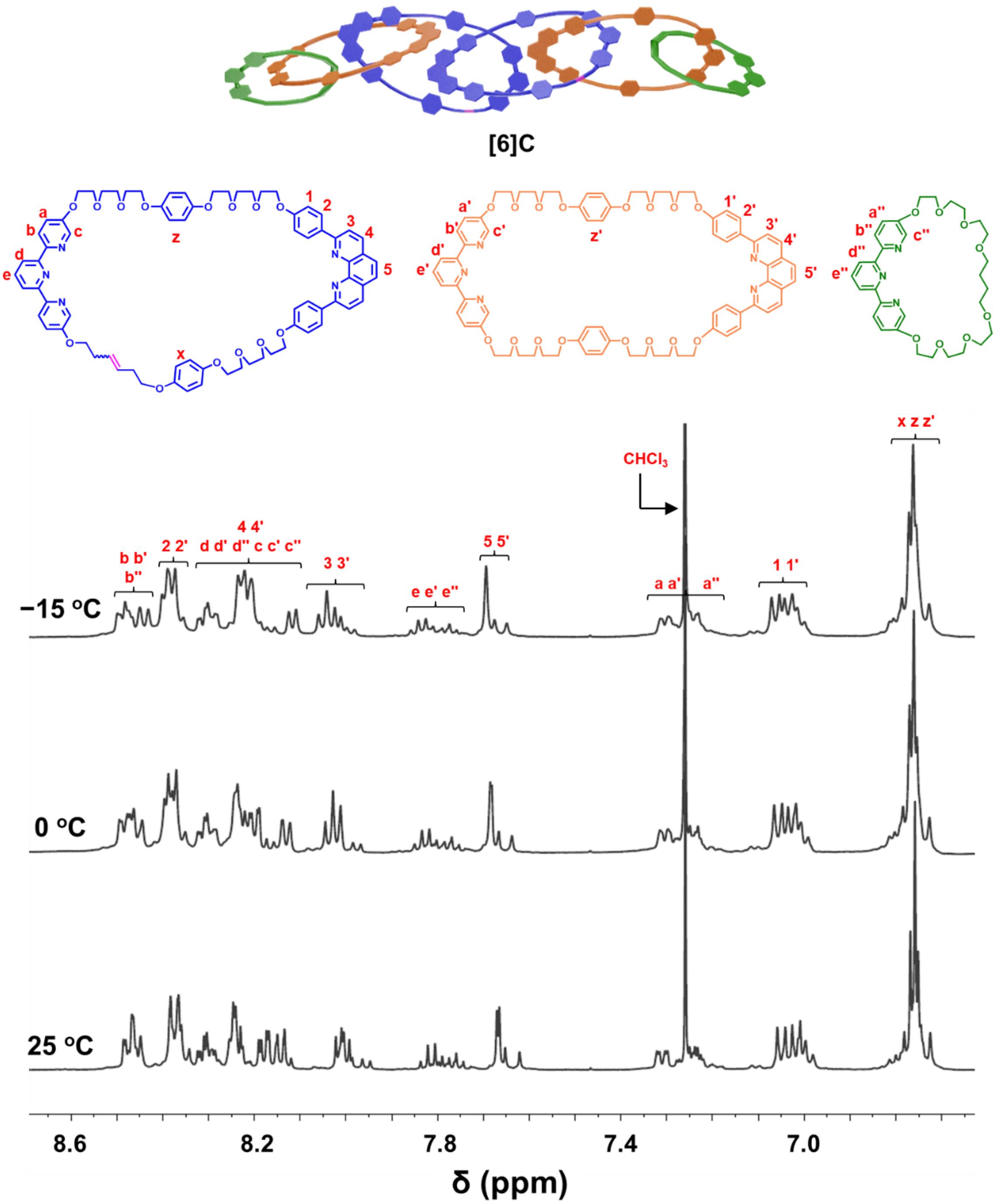

Figure S27. Stacked variable temperature ^{1}H NMR (500 MHz, $CDCl_3$) spectra of aromatic region of **[6]C**: top (−15 °C), middle (0 °C), and bottom (25 °C). Individual macrocycles shown for clarity.

As the temperature is lowered, many of the sharp peaks observed at 25 °C begin to broaden. This is likely due to the conformational motions becoming more restricted at lower temperatures.

2) 2D ^{1}H-^{1}H NMR Spectra

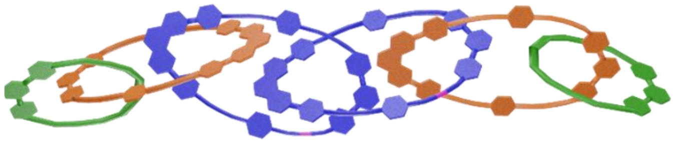

[6]C

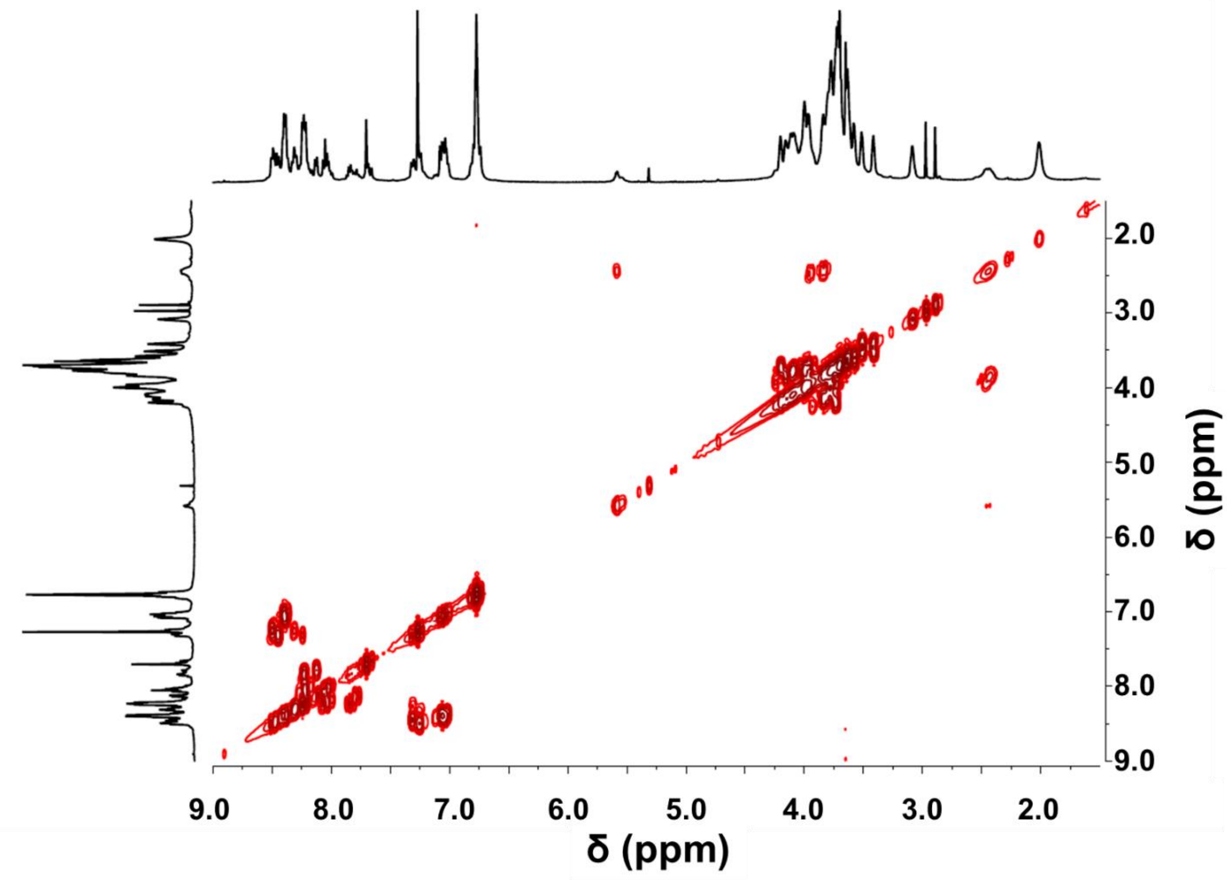

Figure S28. Full variable temperature ^{1}H-^{1}H NMR COSY spectrum (500 MHz, −15 °C, $CDCl_3$) of **[6]C**. See zoomed-in ^{1}H-^{1}H NMR COSY spectrum of **[6]C** in Fig. S29.

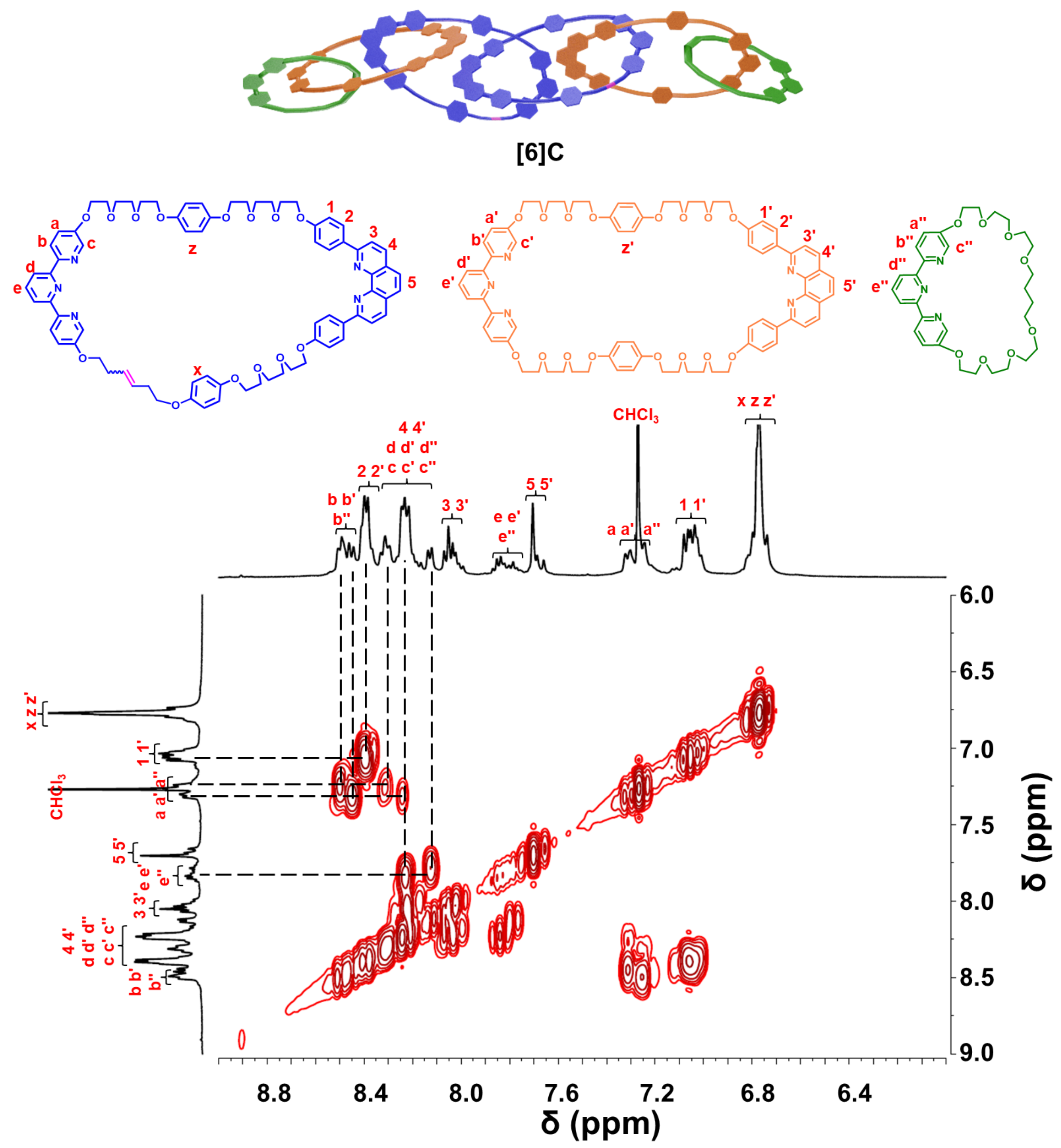

Figure S29. Variable temperature ^{1}H-^{1}H NMR COSY spectrum (500 MHz, −15 °C, $CDCl_3$) showing the aromatic region for **[6]C**.

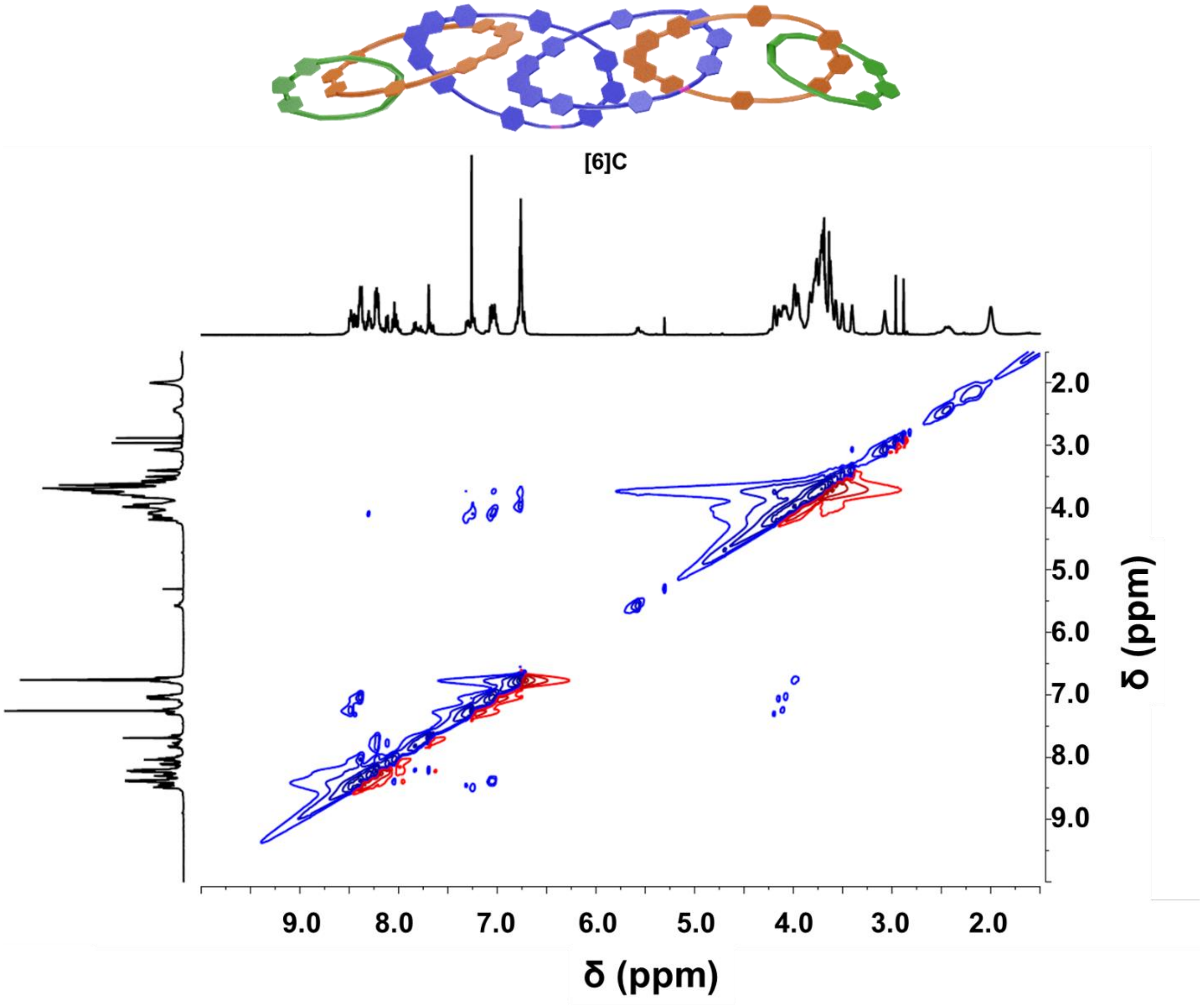

Figure S30. Full variable temperature ^{1}H-^{1}H NMR NOESY spectrum (500 MHz, −15 °C, $CDCl_3$) of **[6]C** with a mixing time of 500 ms. See Fig. S31 for zoomed-in spectrum of **[6]C**.

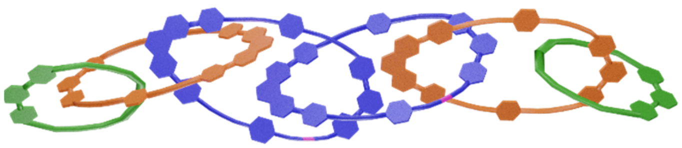

[6]C

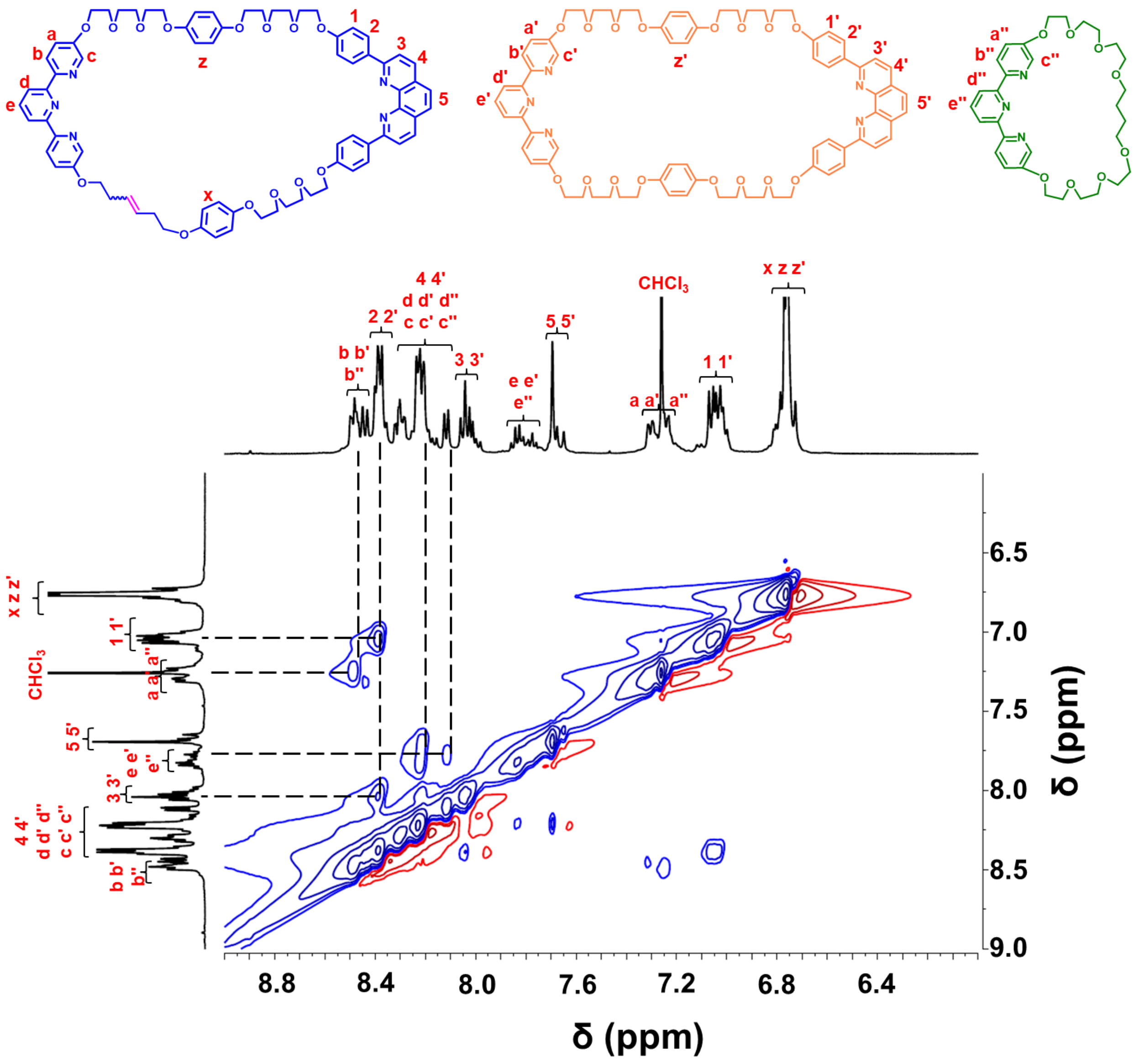

Figure S31. Partial variable temperature ^{1}H-^{1}H NMR NOESY spectrum (500 MHz, −15 °C, $CDCl_3$) showing the aromatic region for **[6]C** with a mixing time of 500 ms.

Although NOE correlations are observed, the chemical similarity of all of the macrocycles present makes it difficult to assign proton resonances on specific macrocycles.

3) ^{13}C NMR Spectra

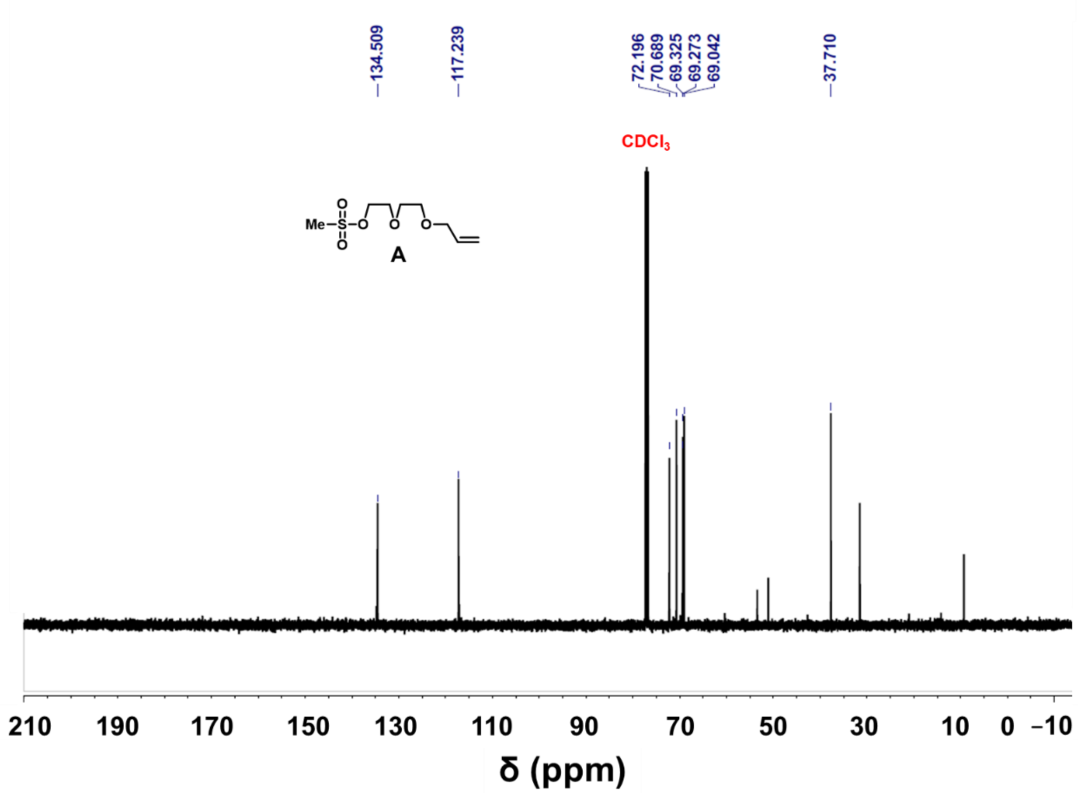

Figure S32. Full ^{13}C NMR spectrum (125 MHz, 25 °C, $CDCl_3$) of **A**.

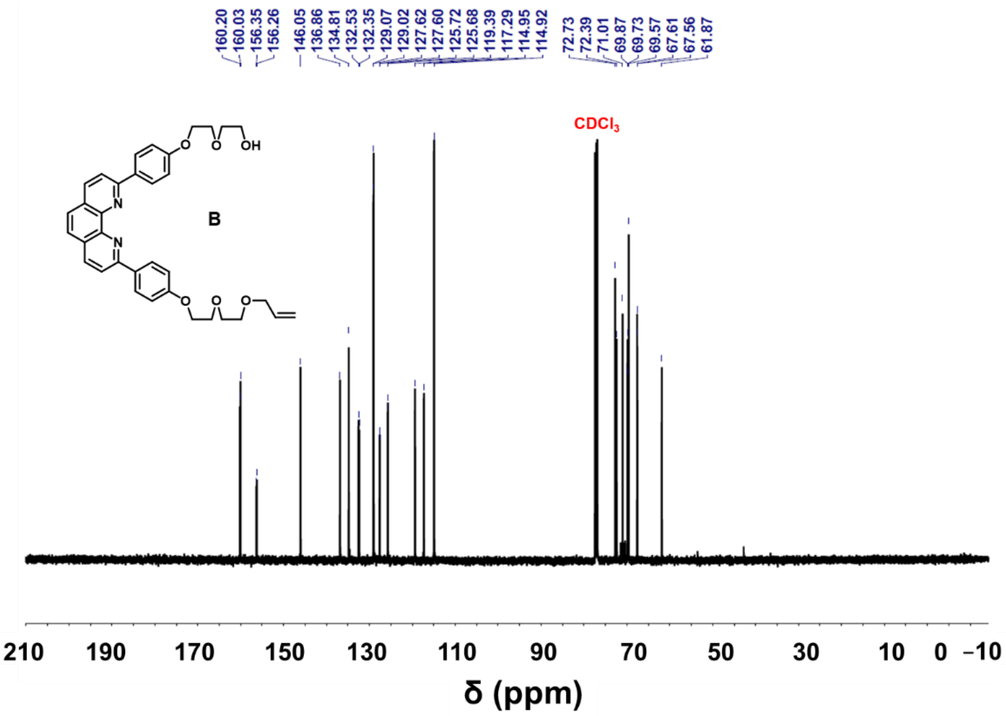

Figure S33. Full ^{13}C NMR spectrum (125 MHz, 25 °C, $CDCl_3$) of **B**.

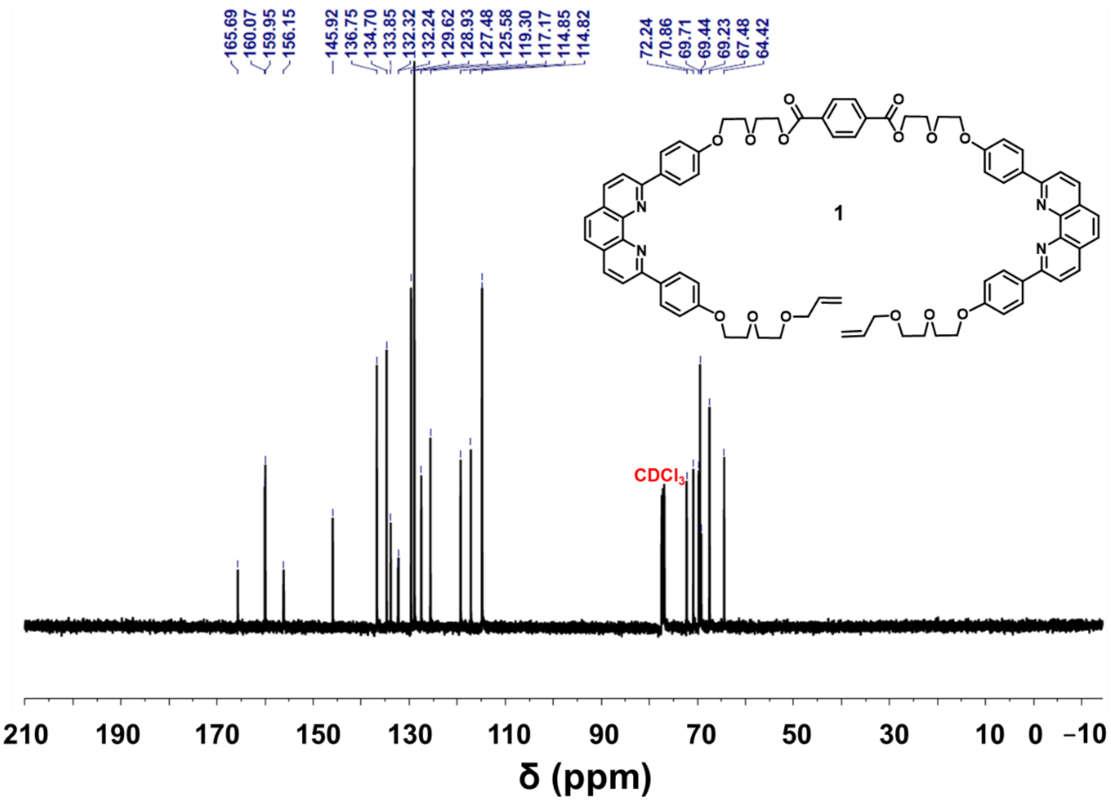

Figure S34. Full ^{13}C NMR spectrum (125 MHz, 25 °C, $CDCl_3$) of **1**.

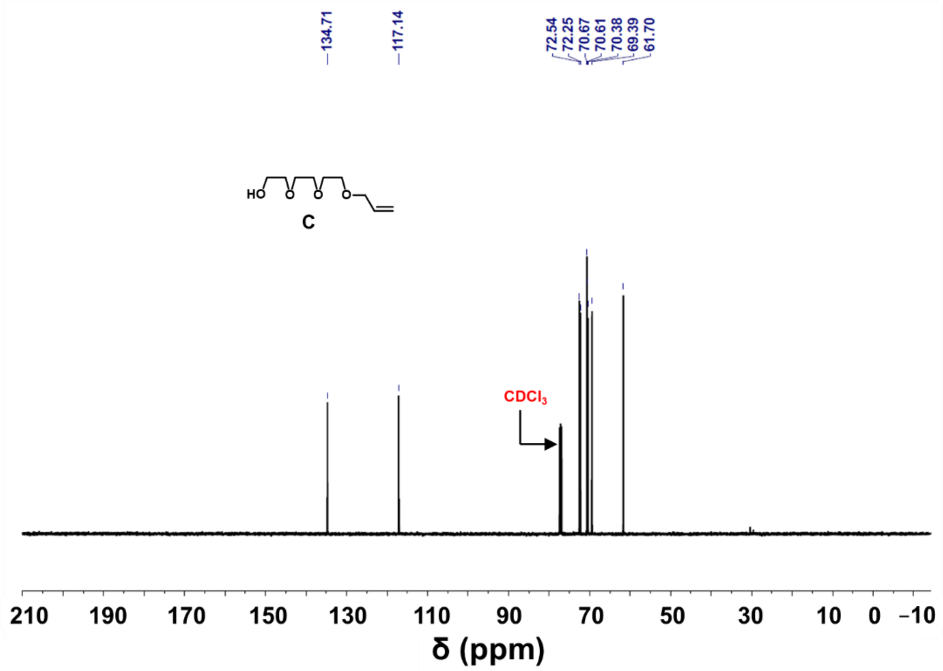

Figure S35. Full ^{13}C NMR spectrum (125 MHz, 25 °C, $CDCl_3$) of **C**.

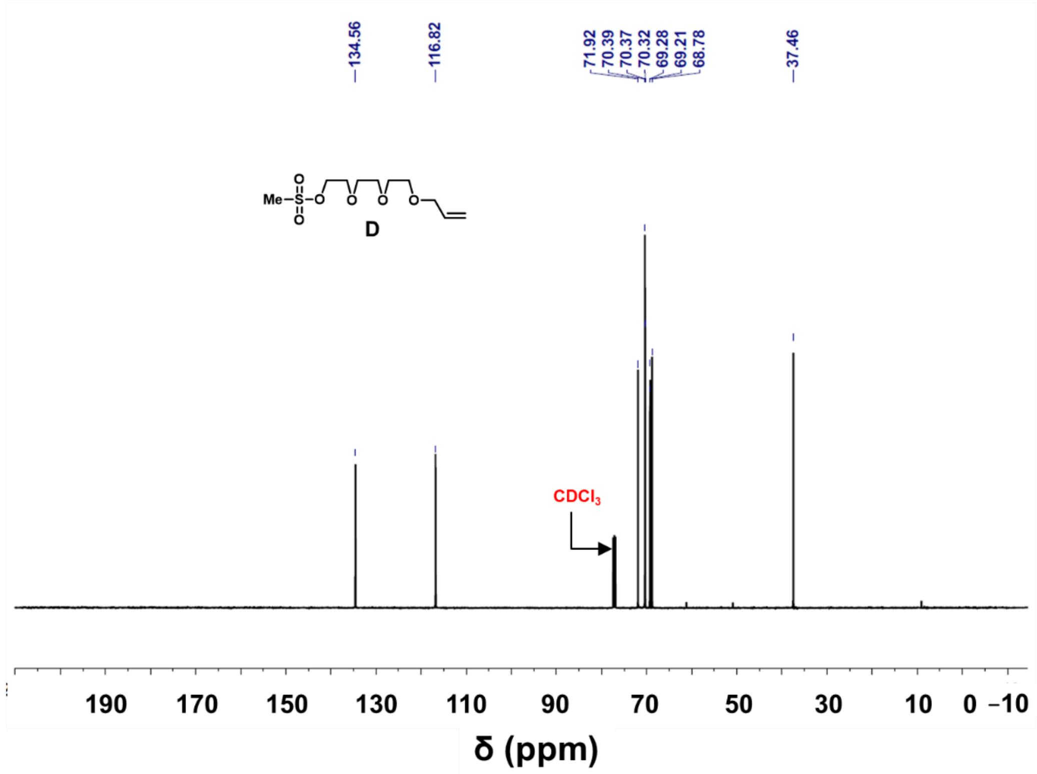

Figure S36. Full ^{13}C NMR spectrum (125 MHz, 25 °C, $CDCl_3$) of **D**.

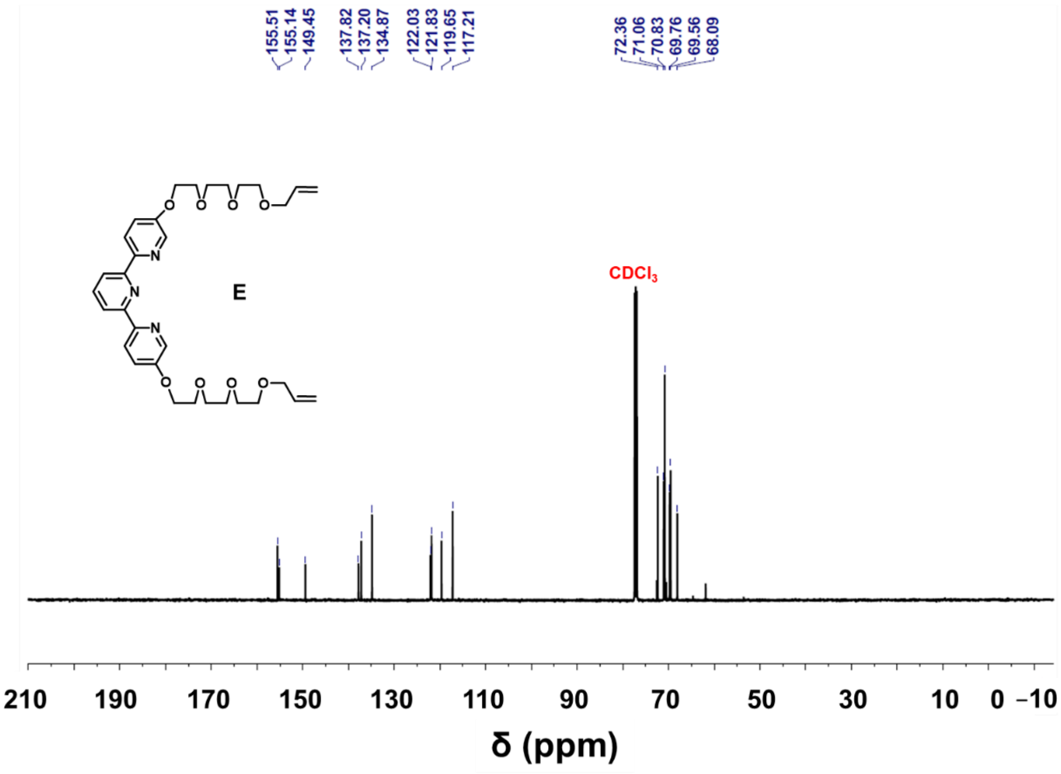

Figure S37. Full ^{13}C NMR spectrum (125 MHz, 25 °C, $CDCl_3$) of **E**.

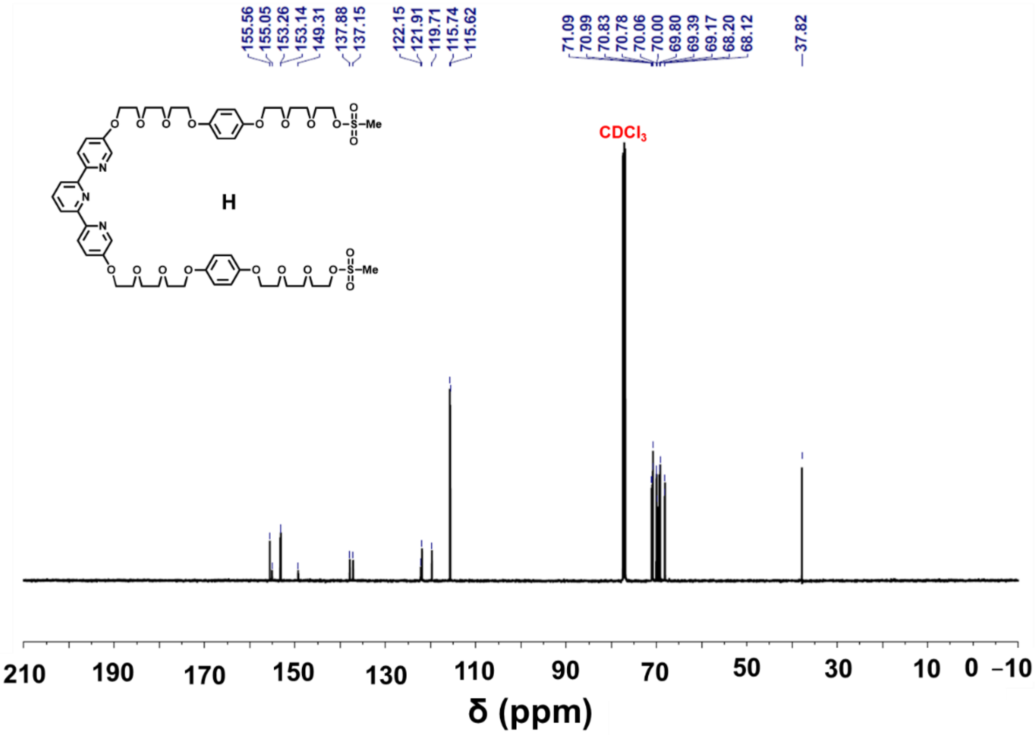

Figure S38. Full ^{13}C NMR spectrum (125 MHz, 25 °C, $CDCl_3$) of **H**.

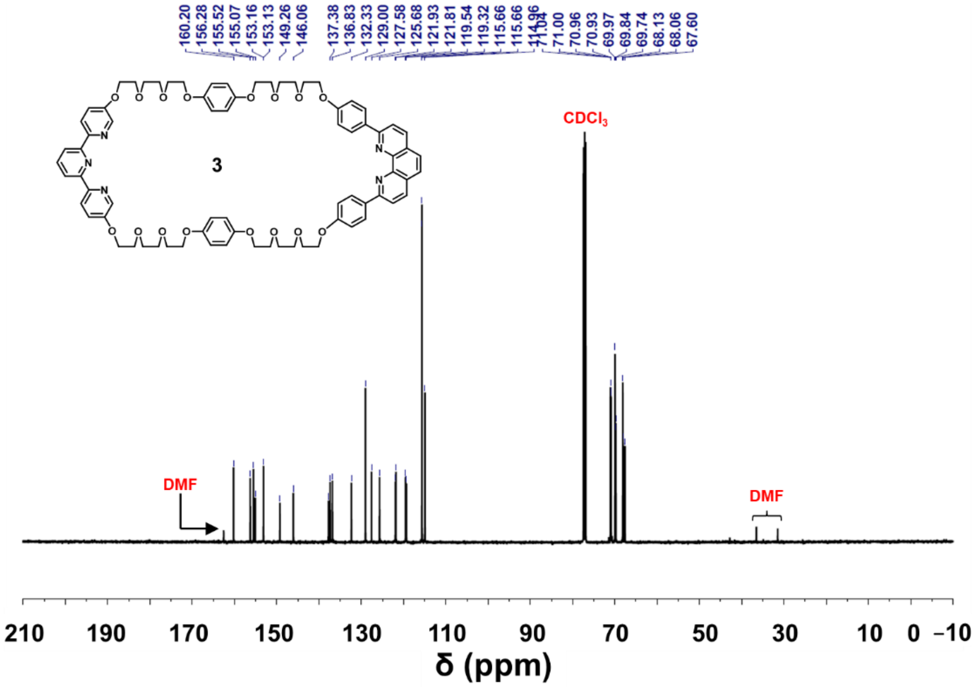

Figure S39. Full ^{13}C NMR spectrum (125 MHz, 25 °C, $CDCl_3$) of **3**.

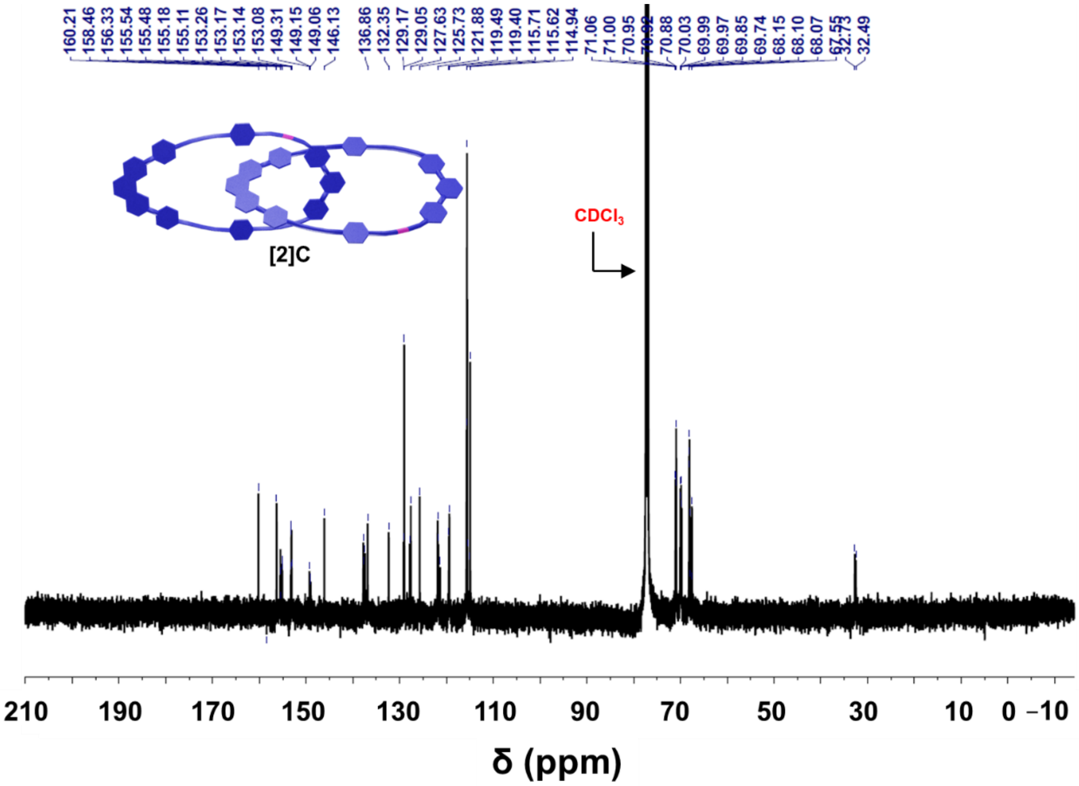

Figure S40. Full ^{13}C NMR spectrum (125 MHz, 25 °C, $CDCl_3$) of **[2]C**.

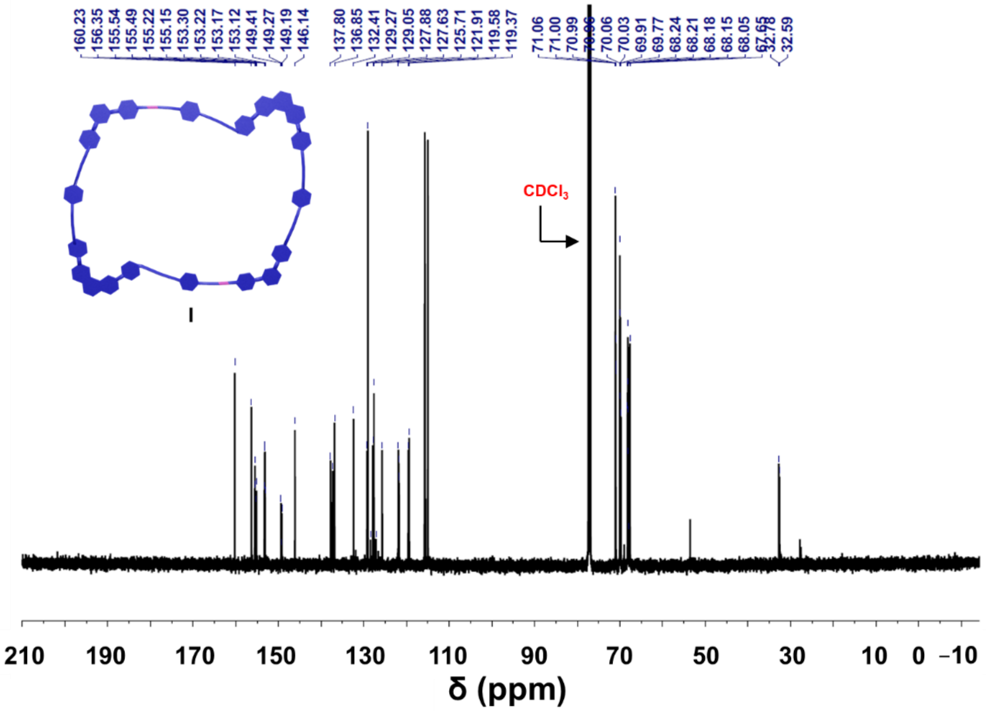

Figure S41. Full ^{13}C NMR spectrum (125 MHz, 25 °C, $CDCl_3$) of **I**.

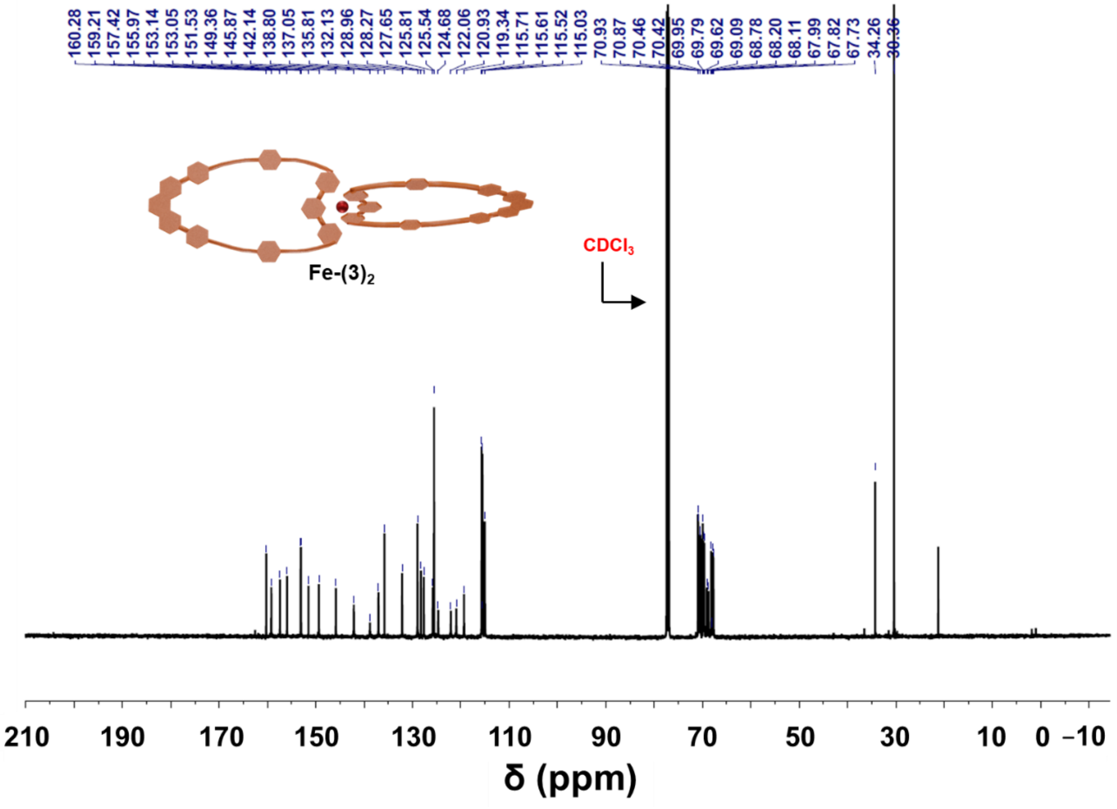

Figure S42. Full ^{13}C NMR spectrum (125 MHz, 25 °C, $CDCl_3$) of **Fe-(3)$_2$**.

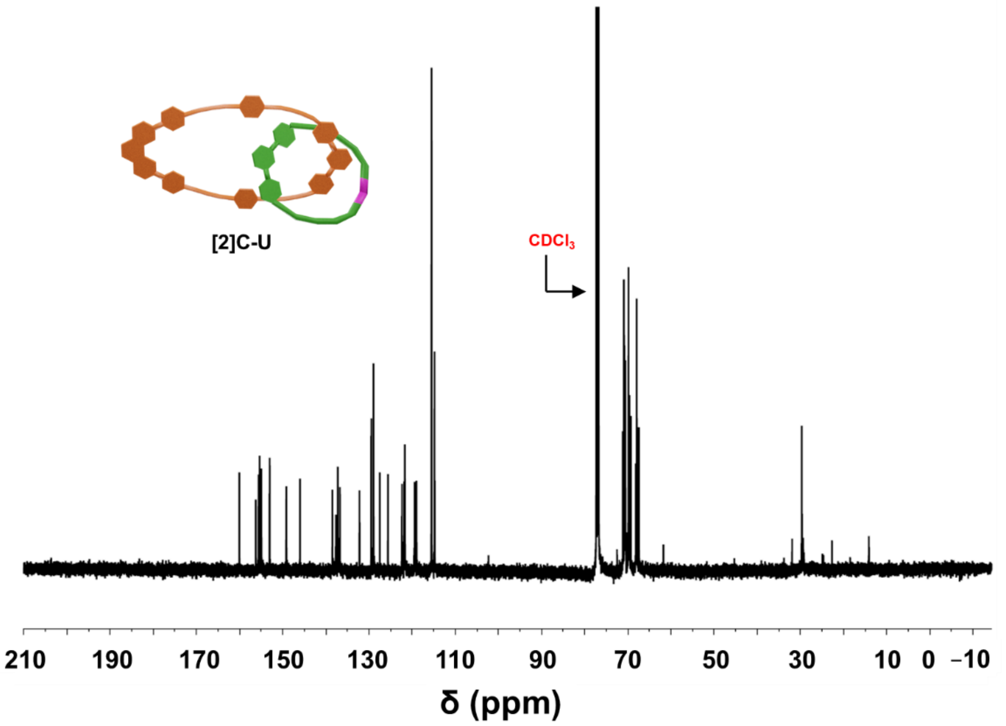

Figure S43. Full ^{13}C NMR spectrum (125 MHz, 25 °C, $CDCl_3$) of **[2]C-U**.

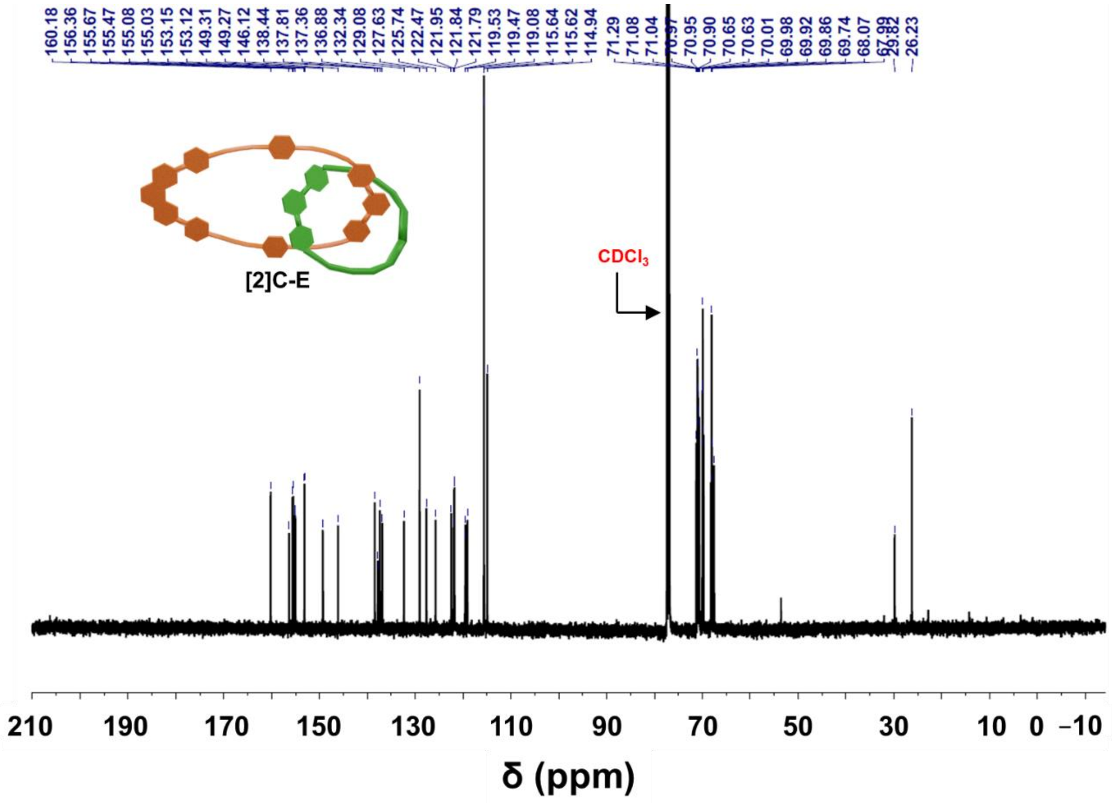

Figure S44. Full ^{13}C NMR spectrum (125 MHz, 25 °C, $CDCl_3$) of **[2]C-E**.

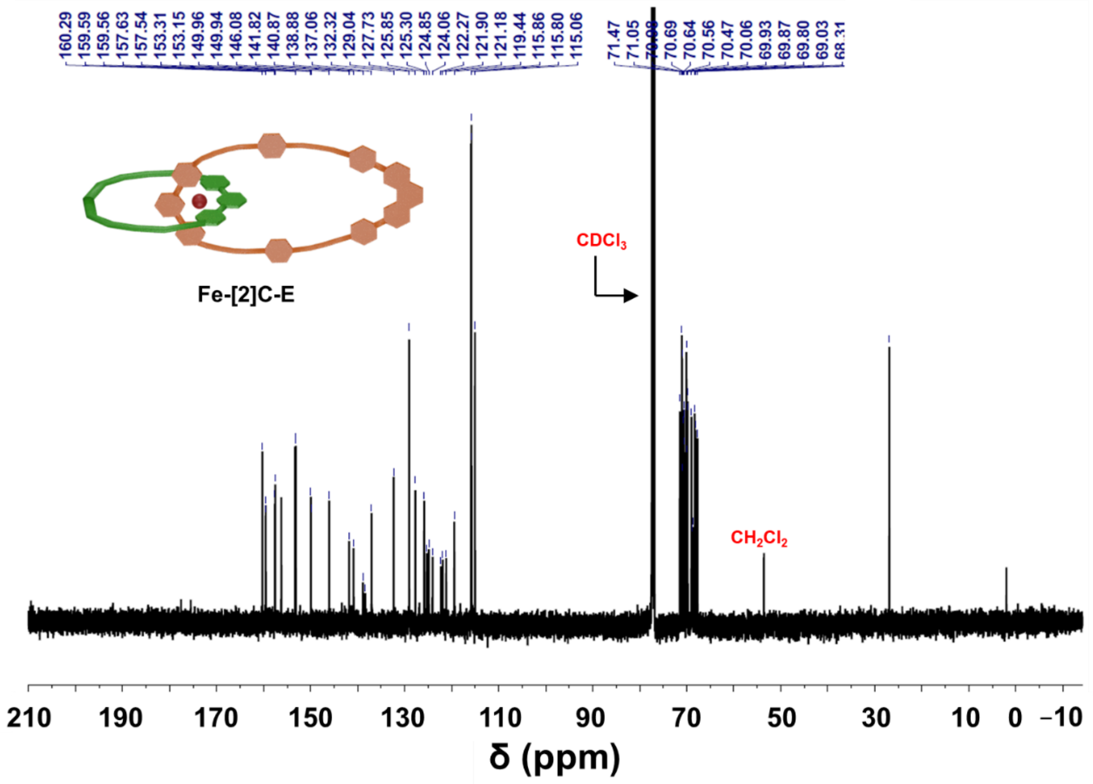

Figure S45. Full ^{13}C NMR spectrum (125 MHz, 25 °C, $CDCl_3$) of **Fe-[2]C-E**.

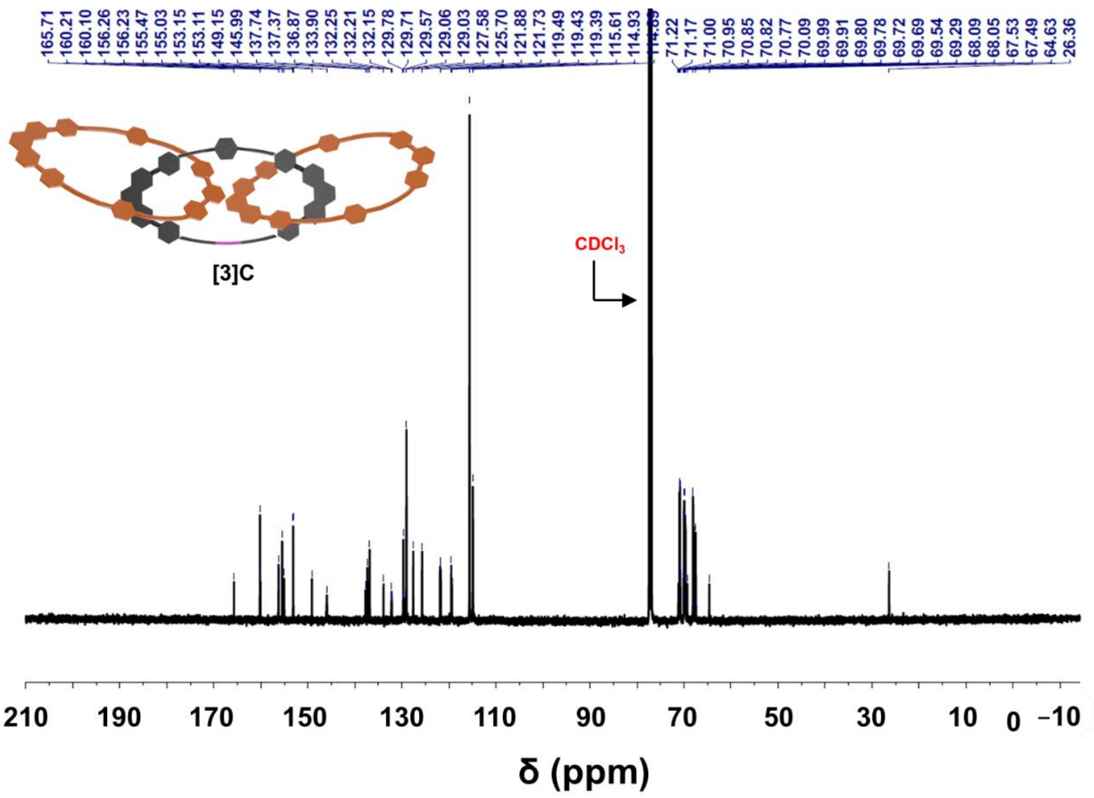

Figure S46. Full ^{13}C NMR spectrum (125 MHz, 25 °C, $CDCl_3$) of **[3]C**.

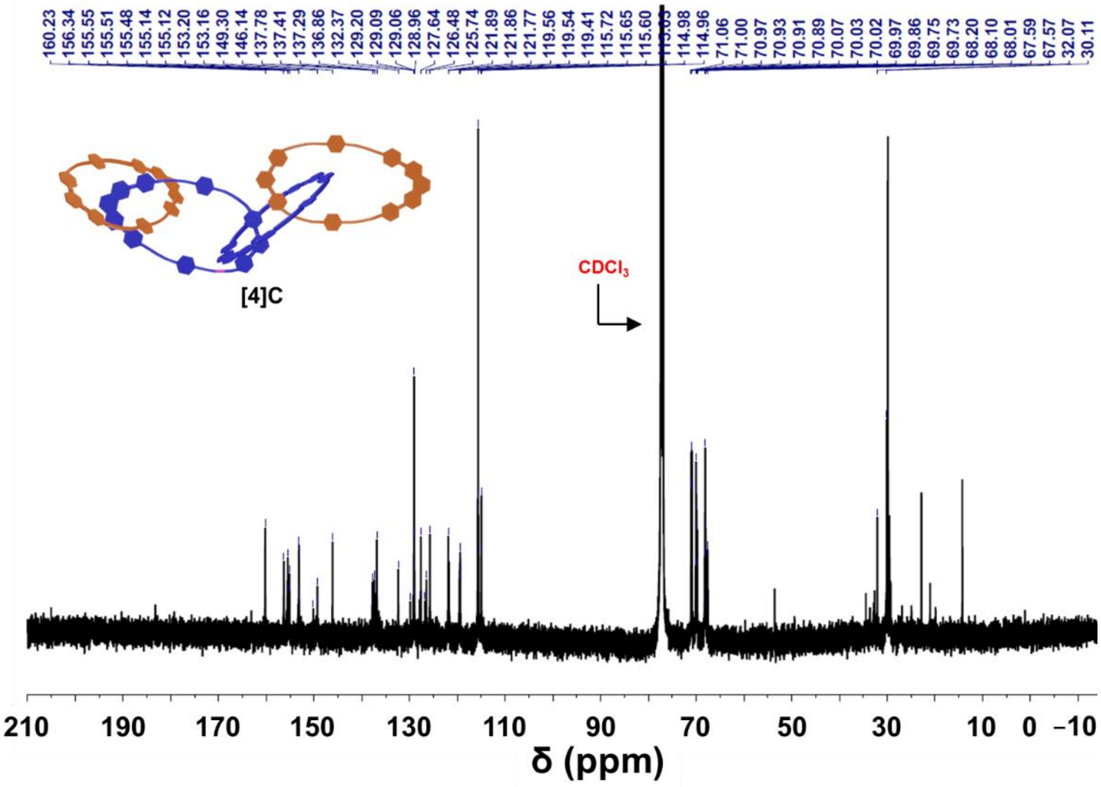

Figure S47. Full ^{13}C NMR spectrum (125 MHz, 25 °C, $CDCl_3$) of **[4]C**.

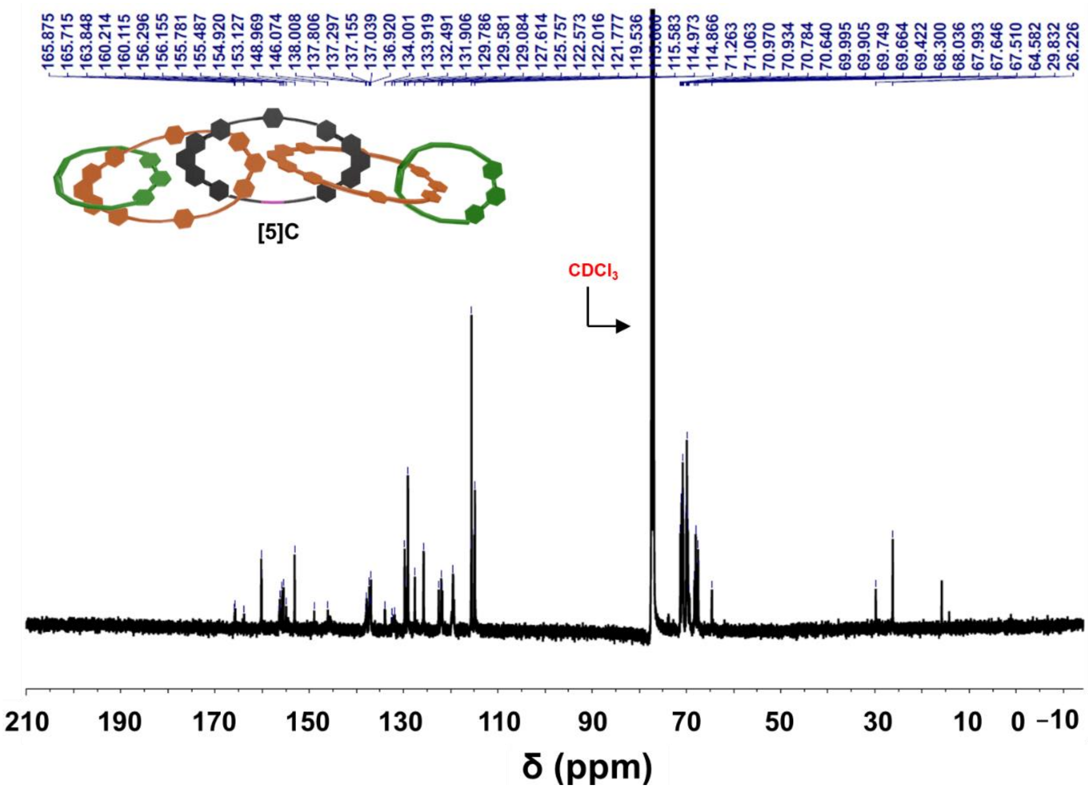

Figure S48. Full ^{13}C NMR spectrum (125 MHz, 25 °C, $CDCl_3$) of **[5]C**.

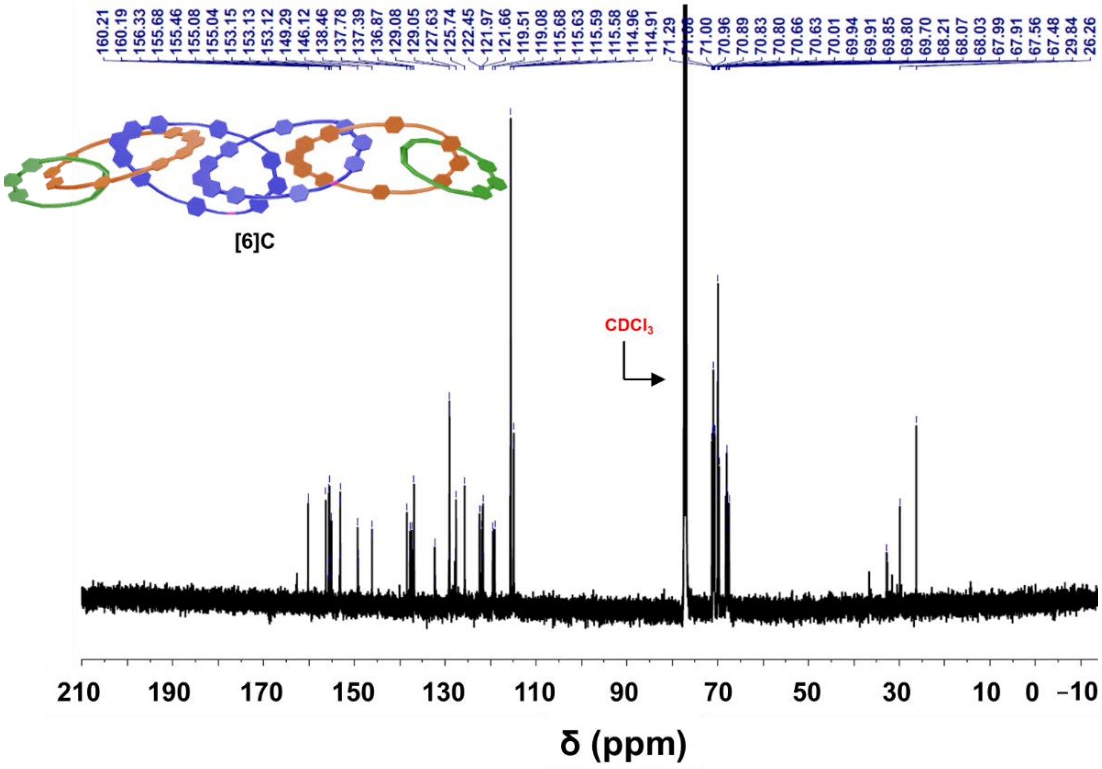

Figure S49. Full ^{13}C NMR spectrum (125 MHz, 25 °C, $CDCl_3$) of **[6]C**.

4) UV-Vis Spectra

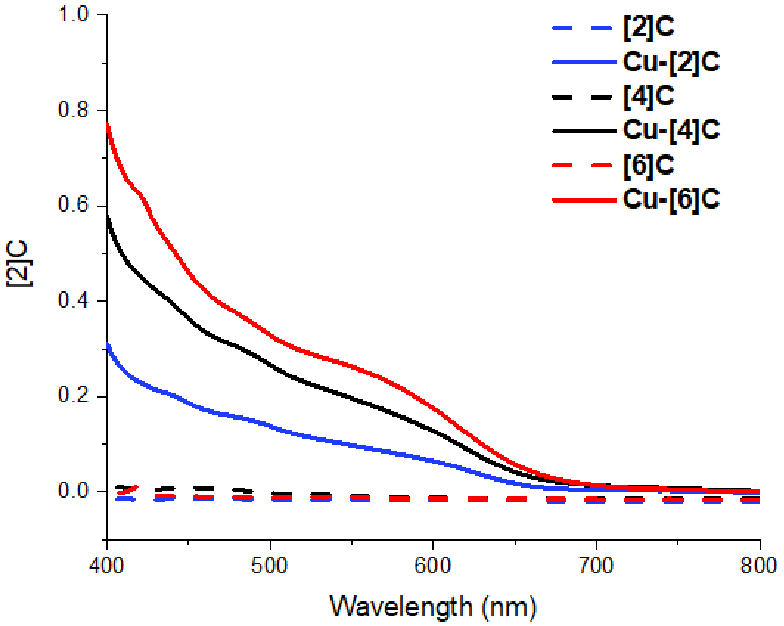

Figure S50. UV-vis spectra of **[2]C**, **[4]C**, and **[6]C** (0.1 mM, DMSO) before and after metalation with $Cu(MeCN)_4PF_6$ (25 mM, DMSO). 1 equivalent of the Cu^+ source was added per ligand in each catenane (i.e., 4 eq. Cu^+ for **[2]C**, 8 eq. Cu^+ for **[4]C**, and 10 eq. Cu^+ for **[6]C**) was added directly to the cuvette. The sample was inverted 3 times and the metalated spectrum was recorded.

This experiment was done in order to demonstrate that the isolated catenanes could be remetalated with Cu^+. However, it should be noted that this metalation will not exclusively form Cu^+-bis(phen) complexes because heteroleptic Cu^+-phen-terpy complexes are also favorable[4] because both species are 18 electron complexes. The selectively of orthogonal metalation during the synthesis of these catenanes was achieved by first blocking the terpy ligands with Fe^{2+}, followed by metalation with Cu^+.

5) Gel Permeation Chromatography (GPC)

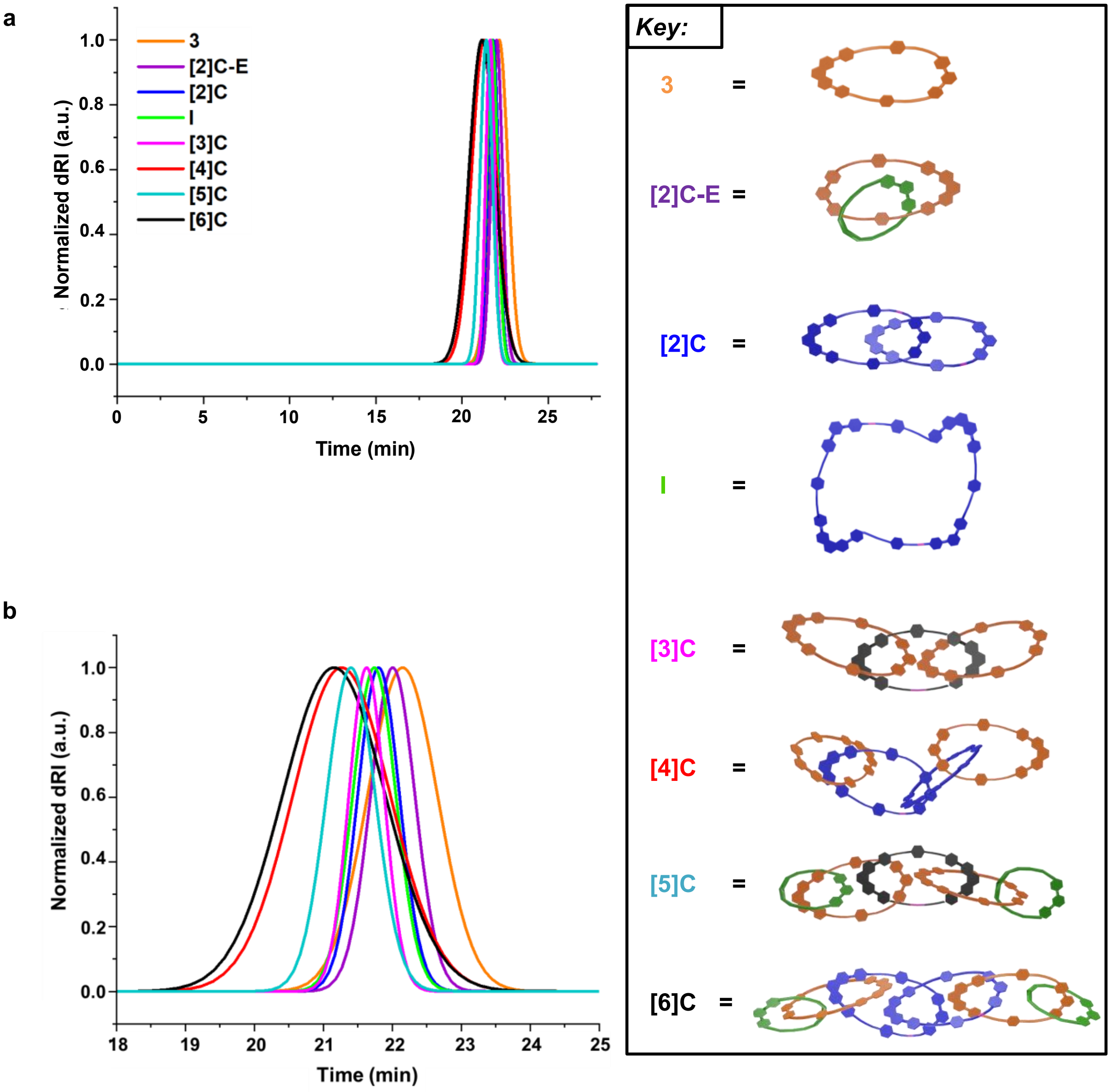

Figure S51. (**a**) Full analytical GPC (dRI) traces of **3**, **[2]C-E**, **[2]C**, **I**, **[3]C**, **[4]C**, **[5]C**, and **[6]C**. (**b**) Zoomed-in GPC (dRI) traces of **3**, **[2]C-E**, **[2]C**, **I**, **[3]C**, **[4]C**, **[5]C**, and **[6]C**.

These samples were run using two Shodex GPC KD-806M columns in sequence in a DMF mobile phase (0.025 M LiBr) running at 60 °C at 1.0 mL·min^{-1}. The raw data were normalized and baseline corrected.

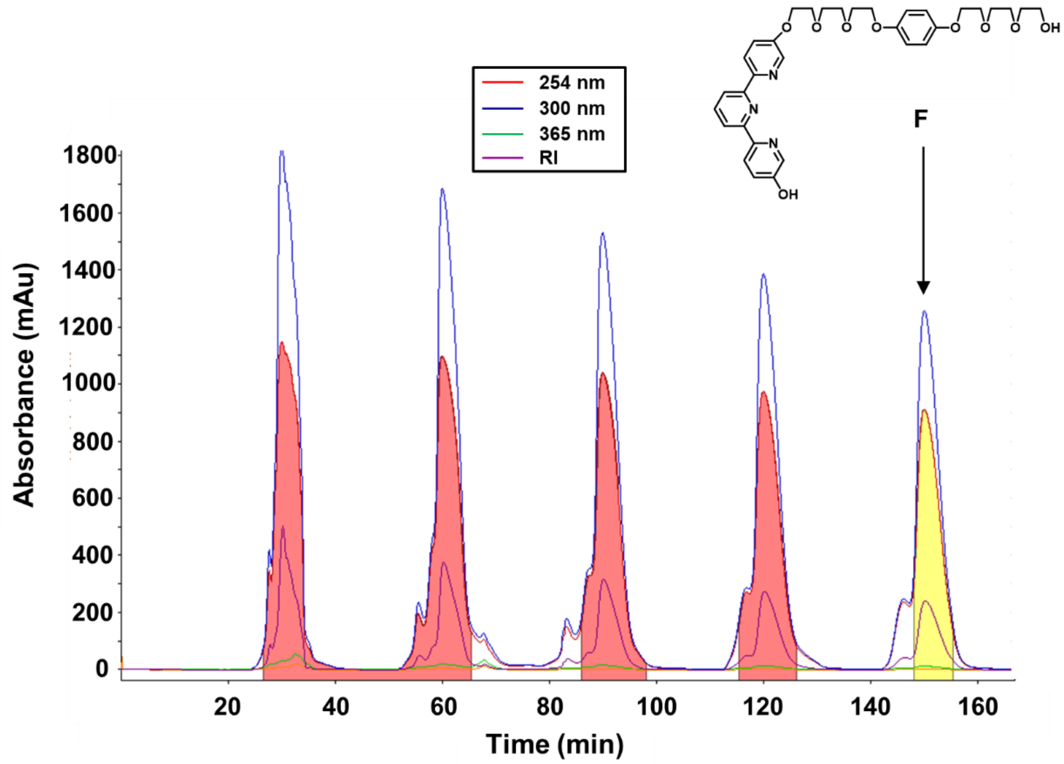

Figure S52. Recycling preparatory GPC trace of **F** using one JAIGEL-2HR column and one JAIGEL-2.5HR column in sequence with a DMF mobile phase at 8 mL·min^{-1}.

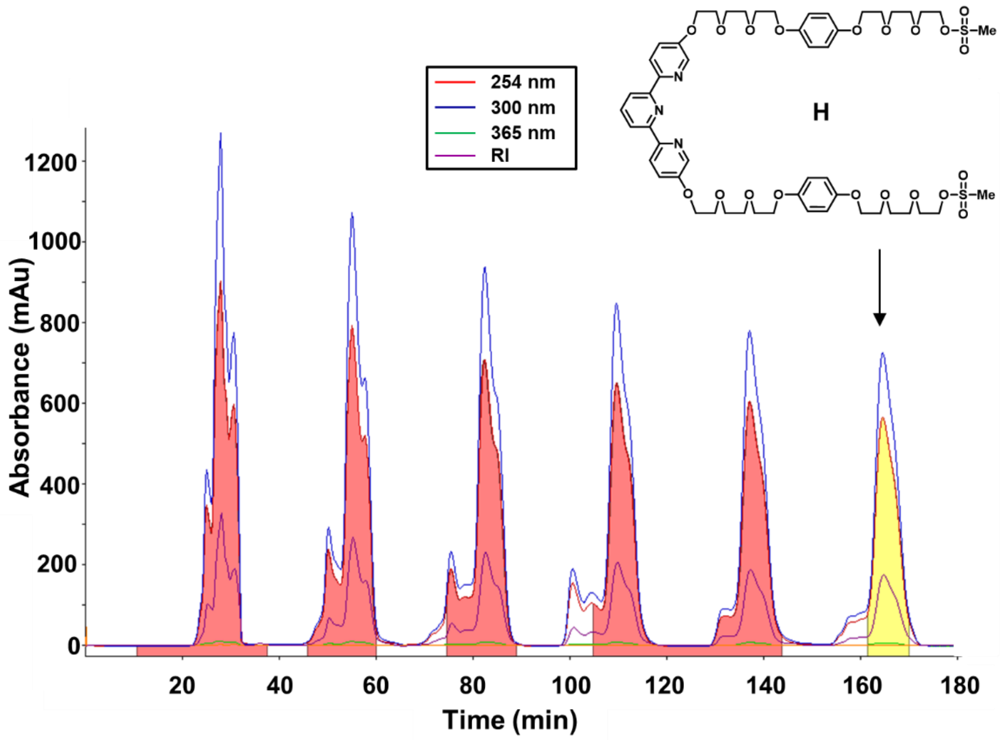

Figure S53. Recycling preparatory GPC trace of **H** using one JAIGEL-2HR column and one JAIGEL-2.5HR column in sequence with a DMF mobile phase at 8 mL·min^{-1}.

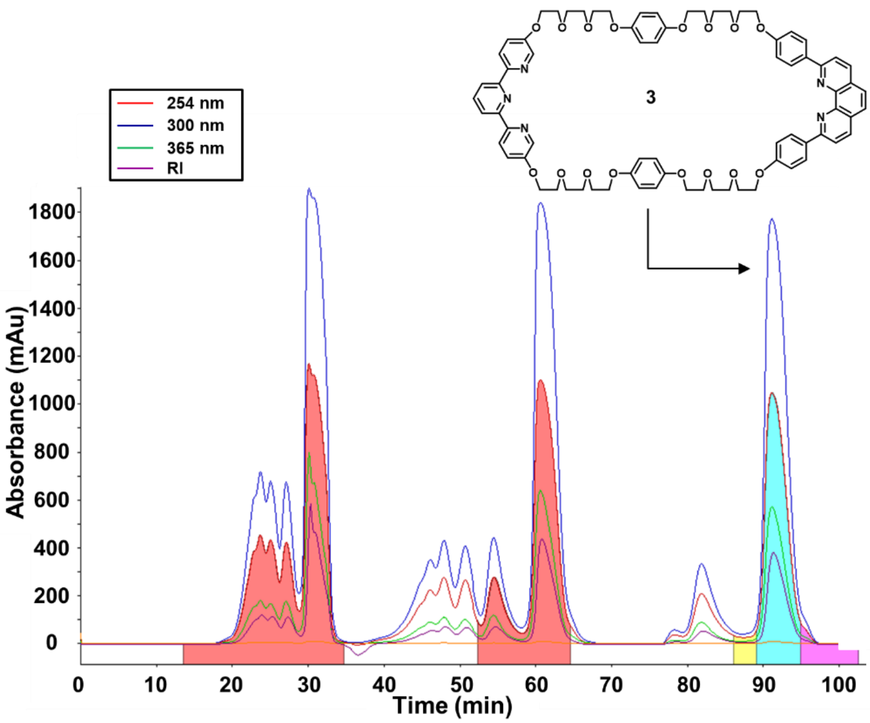

Figure S54. Recycling preparatory GPC trace of **3** using one JAIGEL-2HR column and one JAIGEL-2.5HR column in sequence with a DMF mobile phase at 8 mL·min^{-1}.

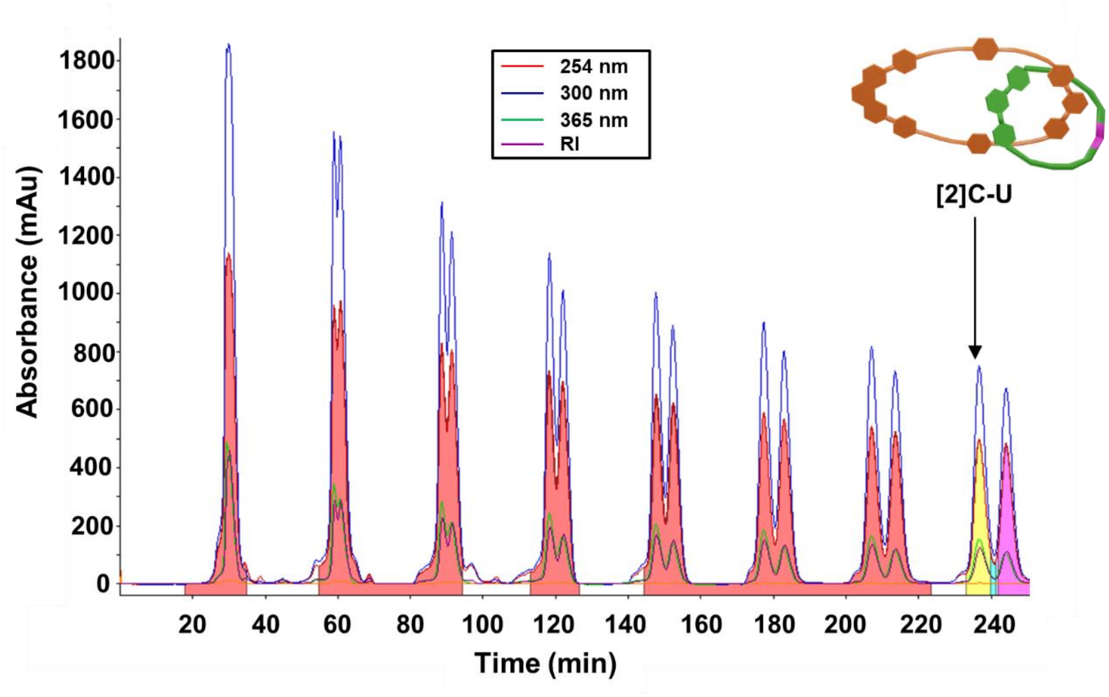

Figure S55. Recycling preparatory GPC trace of **[2]C-U** using one JAIGEL-2HR column and one JAIGEL-2.5HR column in sequence with a DMF mobile phase at 8 mL·min^{-1}.

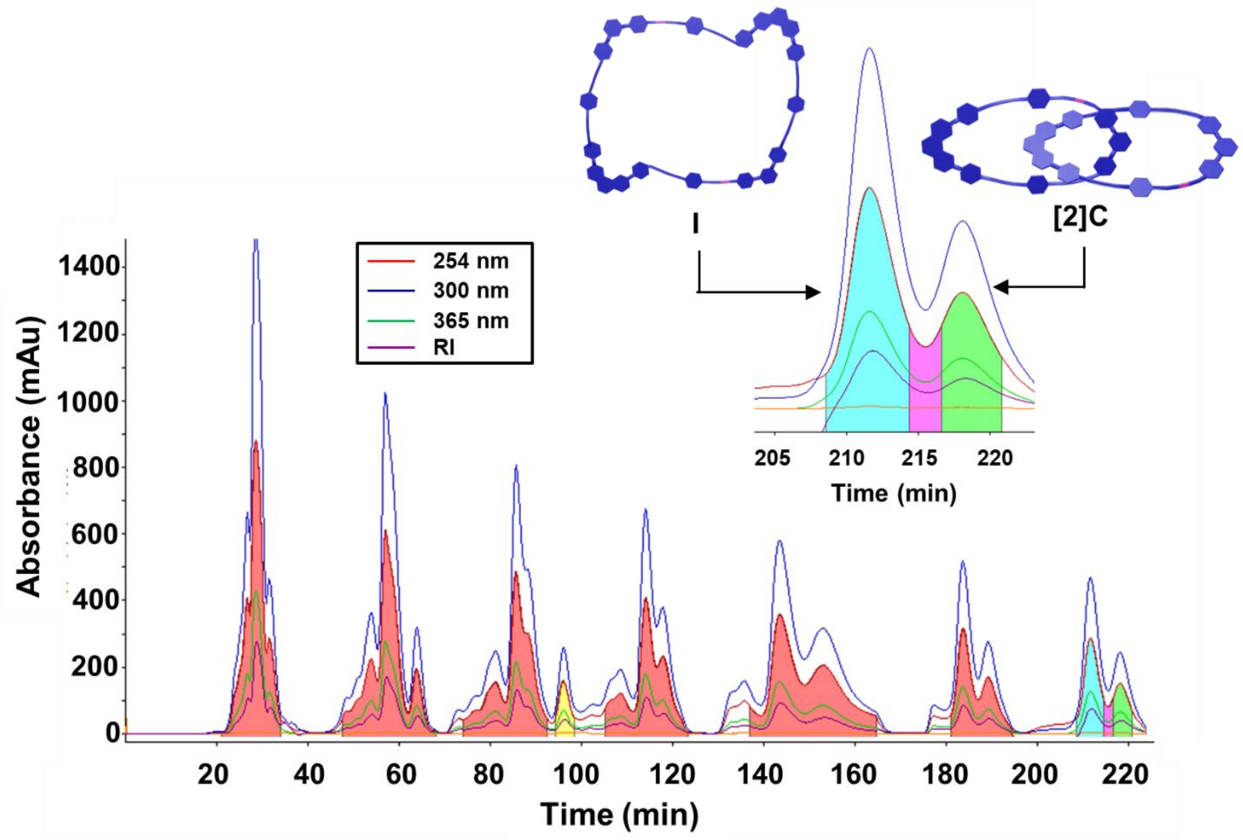

Figure S56. Recycling preparatory GPC trace of **[2]C** and **I** using one JAIGEL-2HR column and one JAIGEL-2.5HR column in sequence with a DMF mobile phase at 8 mL·min^{-1}.

Despite being topological products with the same chemical formula and molecular weight, the figure-eight TPM **I** and the [2]catenane TPM **[2]C** adopt different conformations in solution. Such differences in apparent size make it possible to separate these topological products by size exclusion chromatography. Leigh and coworkers have recently utilized recycling preparatory GPC for the separation of twisted [2]catenane and molecular granny knot topological products.[5]

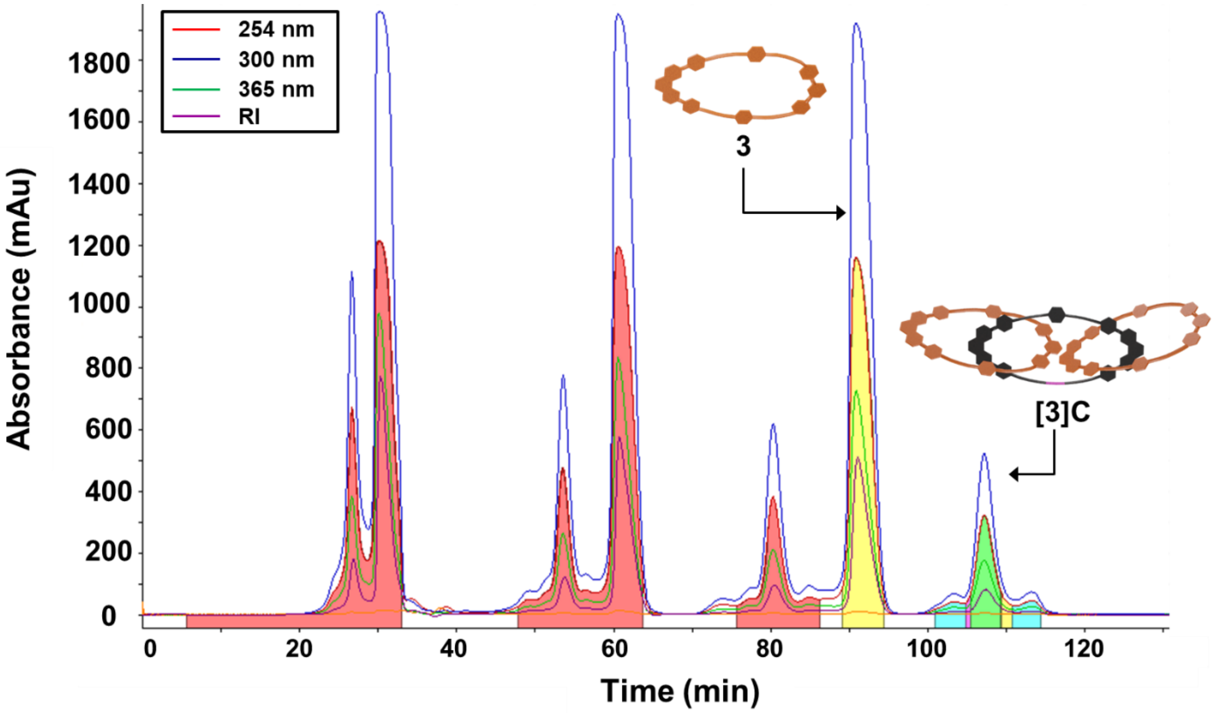

Figure S57. Recycling preparatory GPC trace of **[3]C** using one JAIGEL-2HR column and one JAIGEL-2.5HR column in sequence with a DMF mobile phase at 8 mL·min^{-1}. This trace was from the second GPC purification. Pure product was isolated in the green fraction at 108 min.

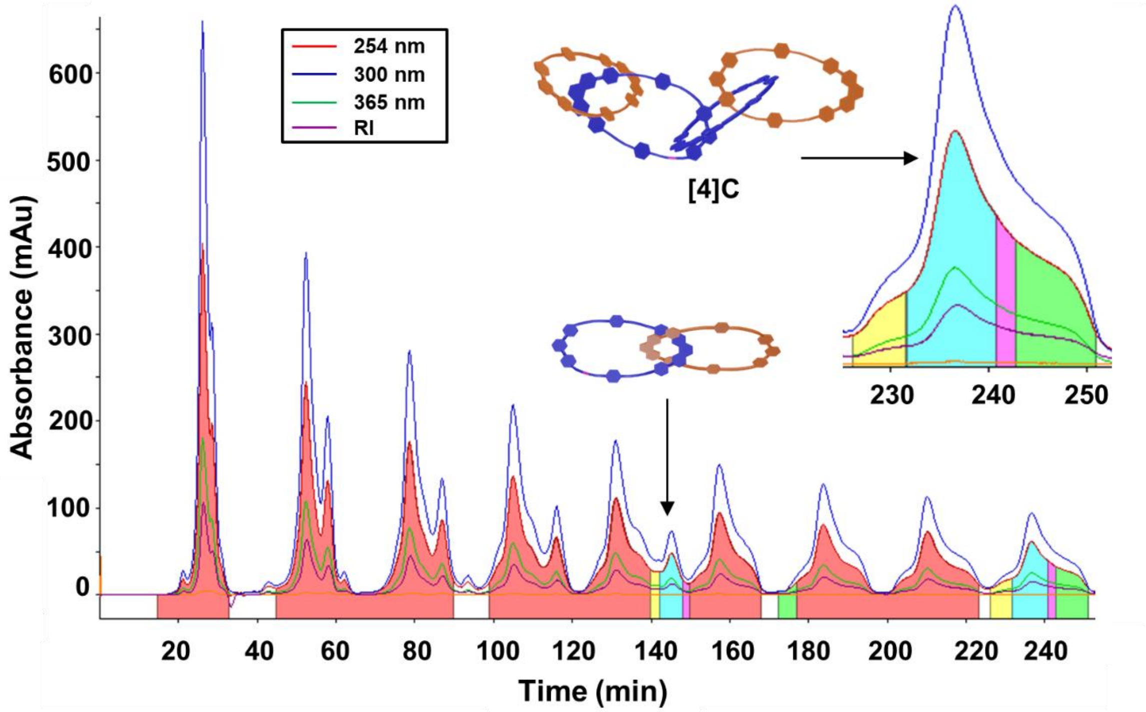

Figure S58. Recycling preparatory GPC trace of **[4]C** using one JAIGEL-2HR column and one JAIGEL-2.5HR column in sequence with a DMF mobile phase at 8 mL·min^{-1}. This trace was from the second GPC purification. Pure product was isolated in the light blue fraction at 235 min.

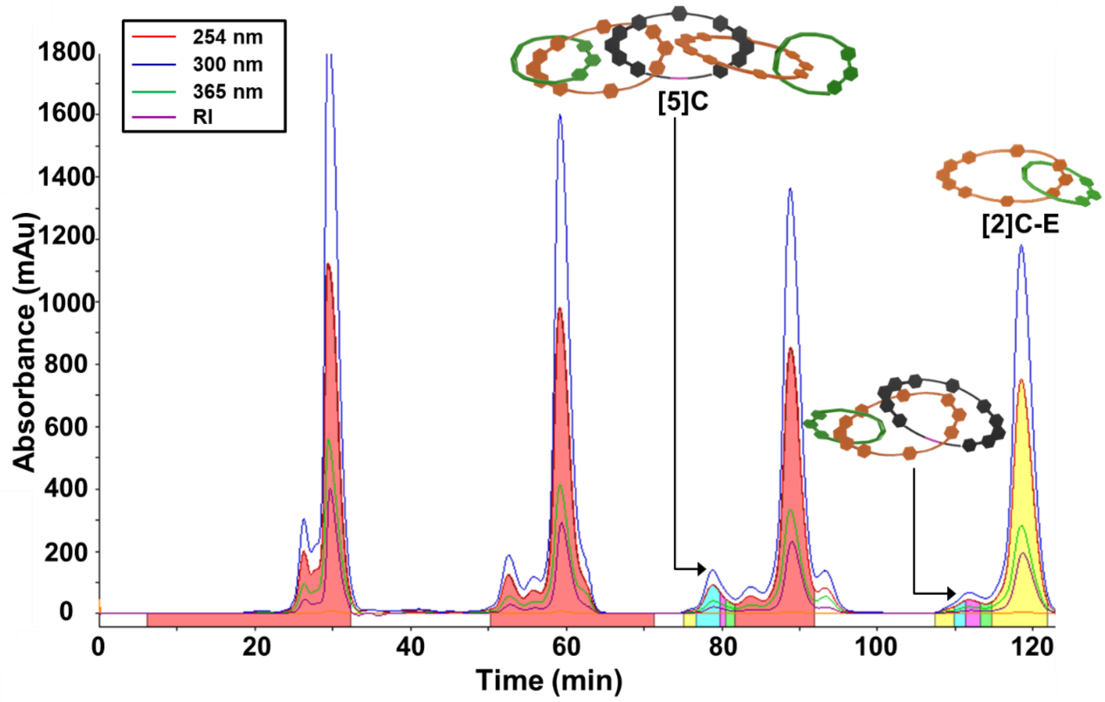

Figure S59. Recycling preparatory GPC trace of **[5]C** using one JAIGEL-2HR column and one JAIGEL-2.5HR column in sequence with a DMF mobile phase at 8 mL·min^{-1}. Pure product was isolated in the light blue fraction at 75 min.

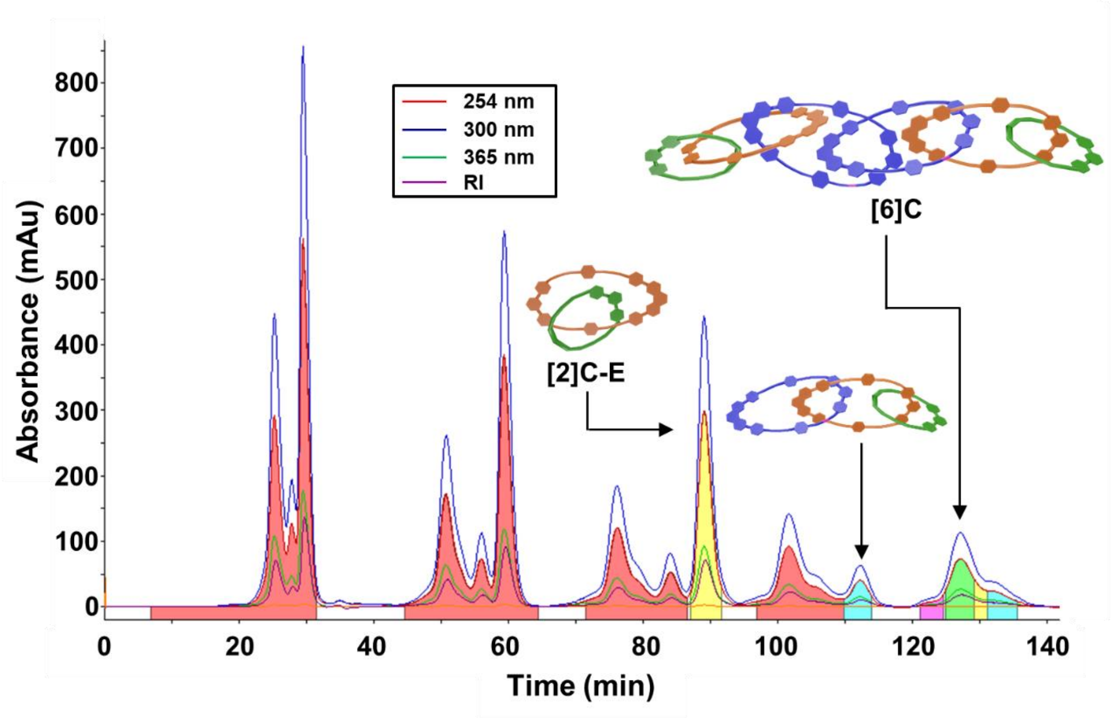

Figure S60. Recycling preparatory GPC trace of **[6]C** using one JAIGEL-2HR column and one JAIGEL-2.5HR column in sequence with a DMF mobile phase at 8 mL·min^{-1}. Pure product was isolated in the green fraction at 125 min. Mixed fractions were also re-purified for overall yield.

6) High-Pressure Liquid Chromatography (HPLC)

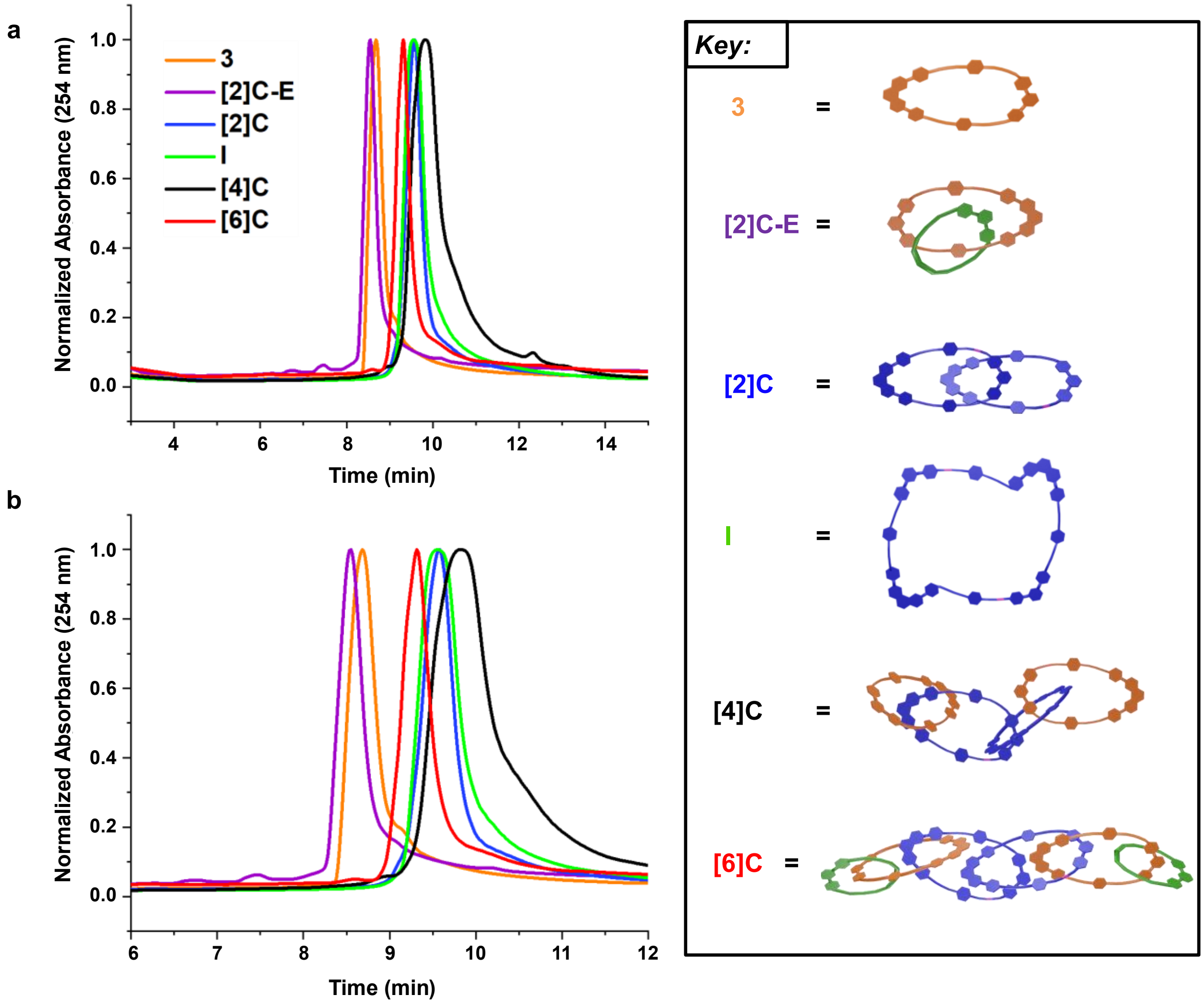

Figure S61. (**a**) Full analytical HPLC (UV, 254 nm) traces of **3**, **[2]C-E**, **[2]C**, **I**, **[3]C**, **[4]C**, **[5]C**, and **[6]C**. (**b**) Zoomed-in analytical HPLC (UV, 254 nm) traces of **3**, **[2]C-E**, **[2]C**, **I**, **[3]C**, **[4]C**, **[5]C**, and **[6]C**.

The samples were run on a Shodex Asahipak ODP-50-2D reverse-phase column with a gradient mobile phase of water (H_2O) with 0.1% trifluoracetic acid (TFA) and acetonitrile (MeCN) with 0.1% TFA running at 40 °C at 0.2 mL·min^{-1}. The gradient mobile phase was MeCN / H_2O (0.1% TFA): 5 to 100% in 10 min and 100% at 15 min. Although there are shoulders in some of the LC traces (i.e., **[5]C**, **[4]C**), these products are pure by preparative GPC (Figs. S54-S60), analytical GPC (Fig. 4c, Fig. S51), and ^{1}H NMR (Figs. S9-S12, and S15-S26). These shoulders could be caused

by these compounds streaking on the column due to limited solubility in H_2O and hydrophilic organic solvents (MeOH, MeCN) that are commonly used in the mobile phase on reverse-phase columns. The low solubility explanation also addresses why these seemingly polar molecules do not elute off the reverse-phase column until a gradient of nearly pure organic solvent is reached. Attempts at running the higher order catenanes on normal-phase HPLC columns also proved unsuccessful presumably due to their inherent polarity.

Section D. Spectrometric Characterization

1) High-res Mass Spectrometry Electrospray Ionization (HRMS-ESI) and Tandem High-res Mass Spectrometry Electrospray Ionization (THRMS-ESI)

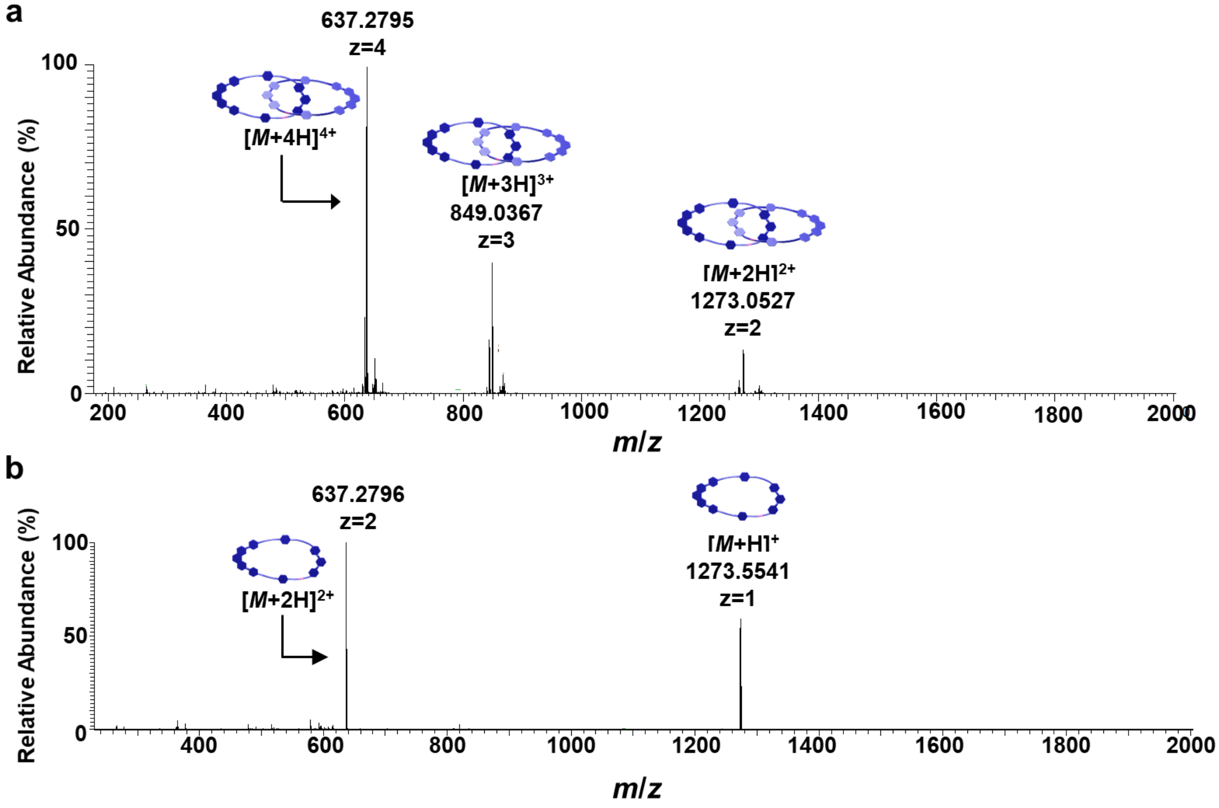

Figure S62. (**a**) Full HRMS-ESI of **[2]C** (**b**) Full THRMS-ESI, i.e., MS/MS of **[2]C**; isolation and fragmentation of **[2]C** peak at $[M+3H]^{3+}$ = 849.03.

The breaking of one macrocycle of **[2]C** via fragmentation yields an intact, unfragmented macrocycle as well. This result is only possible with the [2]catenane topology. Note that the

change in charge state (z value) allows for the differentiation between the [2]catenane **[2]C** and TPM macrocycle. Au-Yeung and coworkers observed similar fragmentation patterns with the tandem-MS of their [2]catenanes and [4]catenanes.[6]

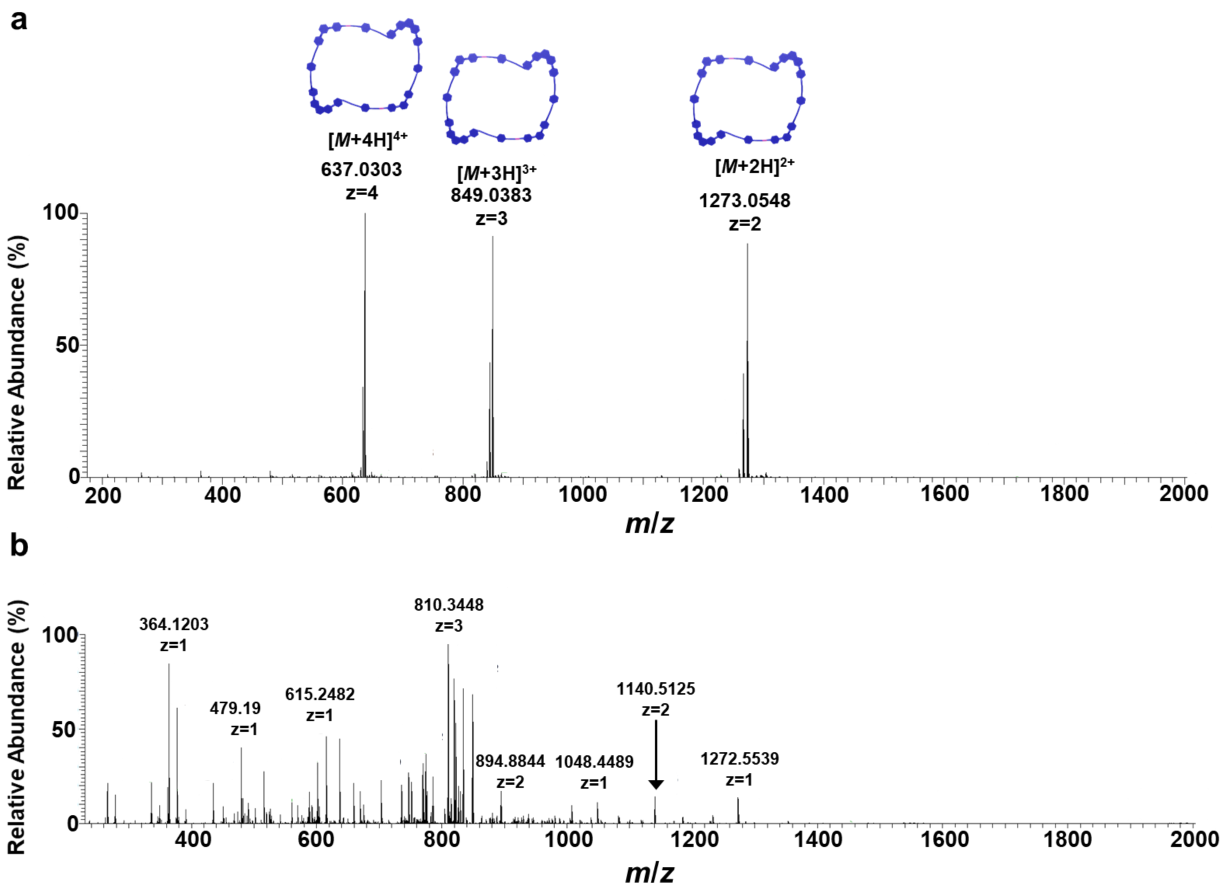

Figure S63. (**a**) Full HRMS-ESI of **I** (**b**) Full THRMS-ESI **I**; isolation and fragmentation of **I** peak at $[M + 3H]^{3+}$ = 849.03.

Unlike the THRMS-ESI of **[2]C**, the fragmentation of **I** results in a complex mass spectrum. Since there are no susceptible bonds in the large macrocycle, the fragmentation appears to be random and unpredictable, and therefore gives a wide range of fragments.

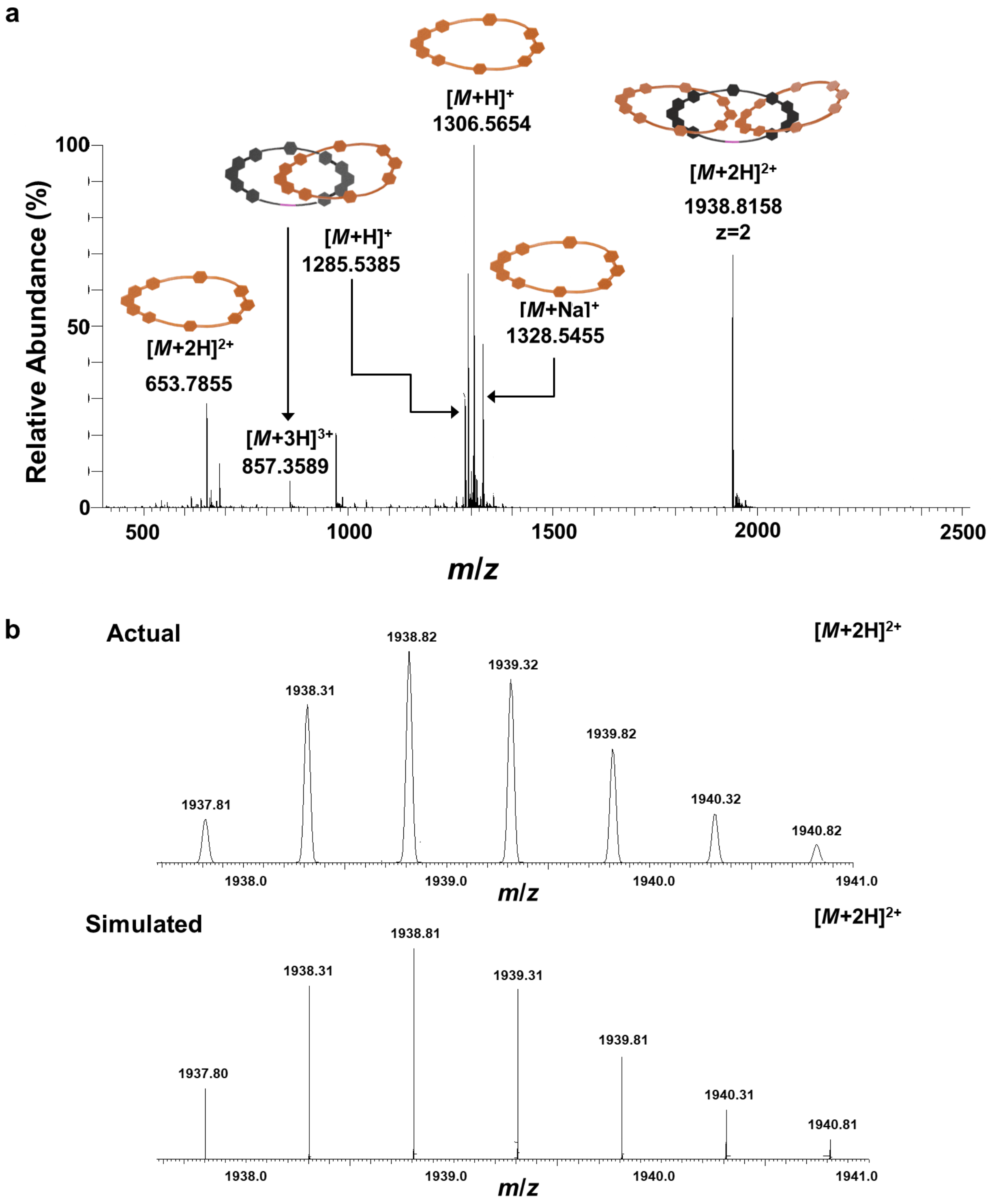

Figure S64. (**a**) Full HRMS-ESI of **[3]C.** (**b**) Zoom-in and simulation of [M + 2H]$^{2+}$ adduct of **[3]C.**

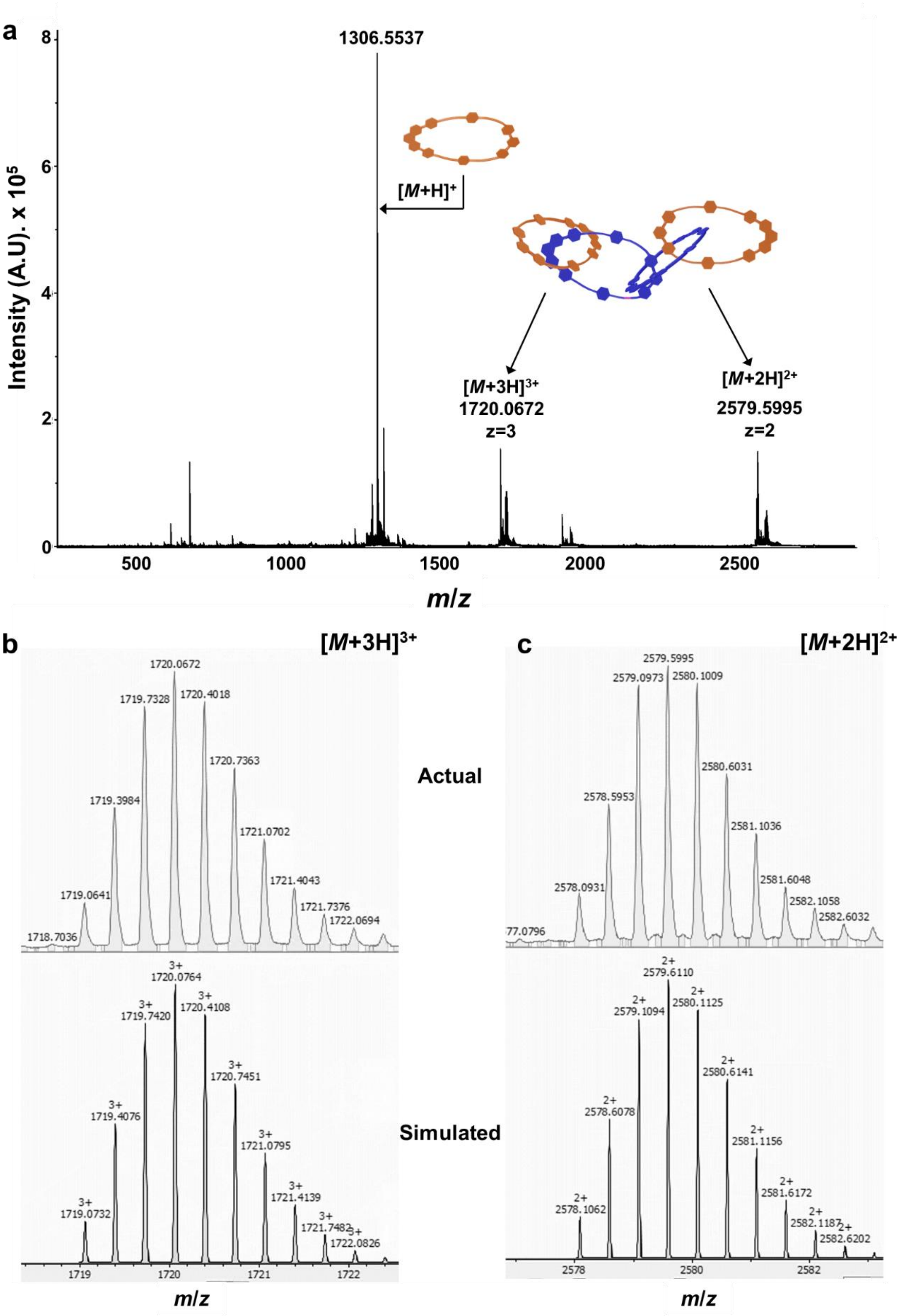

Figure S65. (**a**) Full HRMS-ESI of **[4]C.** (**b**) Zoom-in and simulation of [M + 3H]$^{3+}$ adduct of **[4]C**. (**c**) Zoom-in and simulation of [M + 2H]$^{2+}$ adduct of **[4]C.**

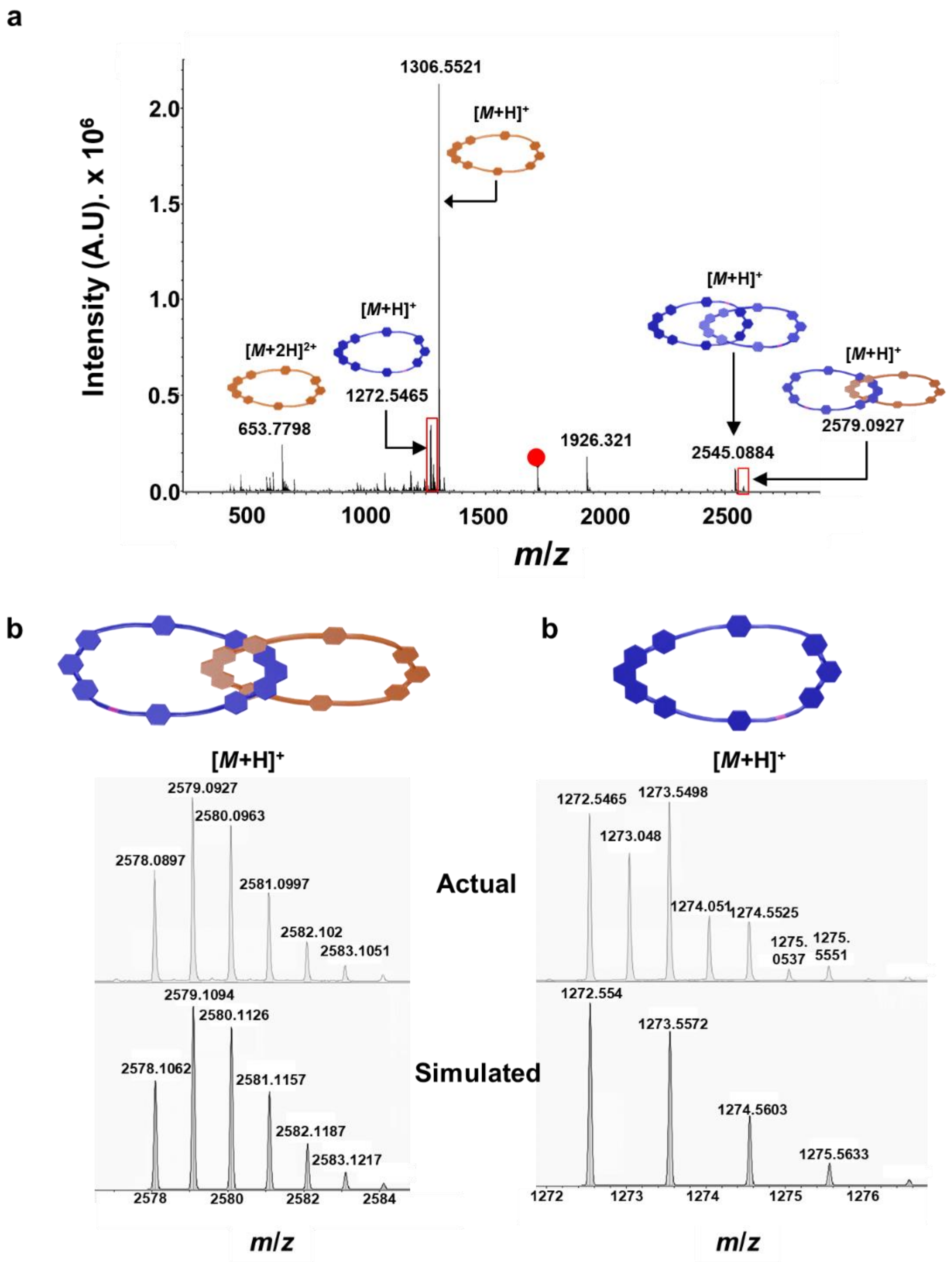

Figure S66. (**a**) Full THRMS-ESI of **[4]C**; isolation and fragmentation of peak at [*M* +3H]$^{3+}$ = 1720.06 at 130 eV collision energy. Red circle represents remaining unfragmented **[4]C**. (**b**) Zoomed-in THRMS-ESI mixed macrocycle [2]catenane fragment. (**c**) Zoomed-in THRMS-ESI of macrocycle fragment. The subpopulation of peaks corresponds to unfragmented **[2]C** flying as [*M* +2H]$^{2+}$, which is distinguishable from the macrocycle by its charge state.

The mixed macrocycle [2]catenane and macrocycle fragments could only have originated from the linear [4]catenane **[4]C**, as opposed to a hypothetical linear [3]catenane resulting from a figure-of-eight precursor.

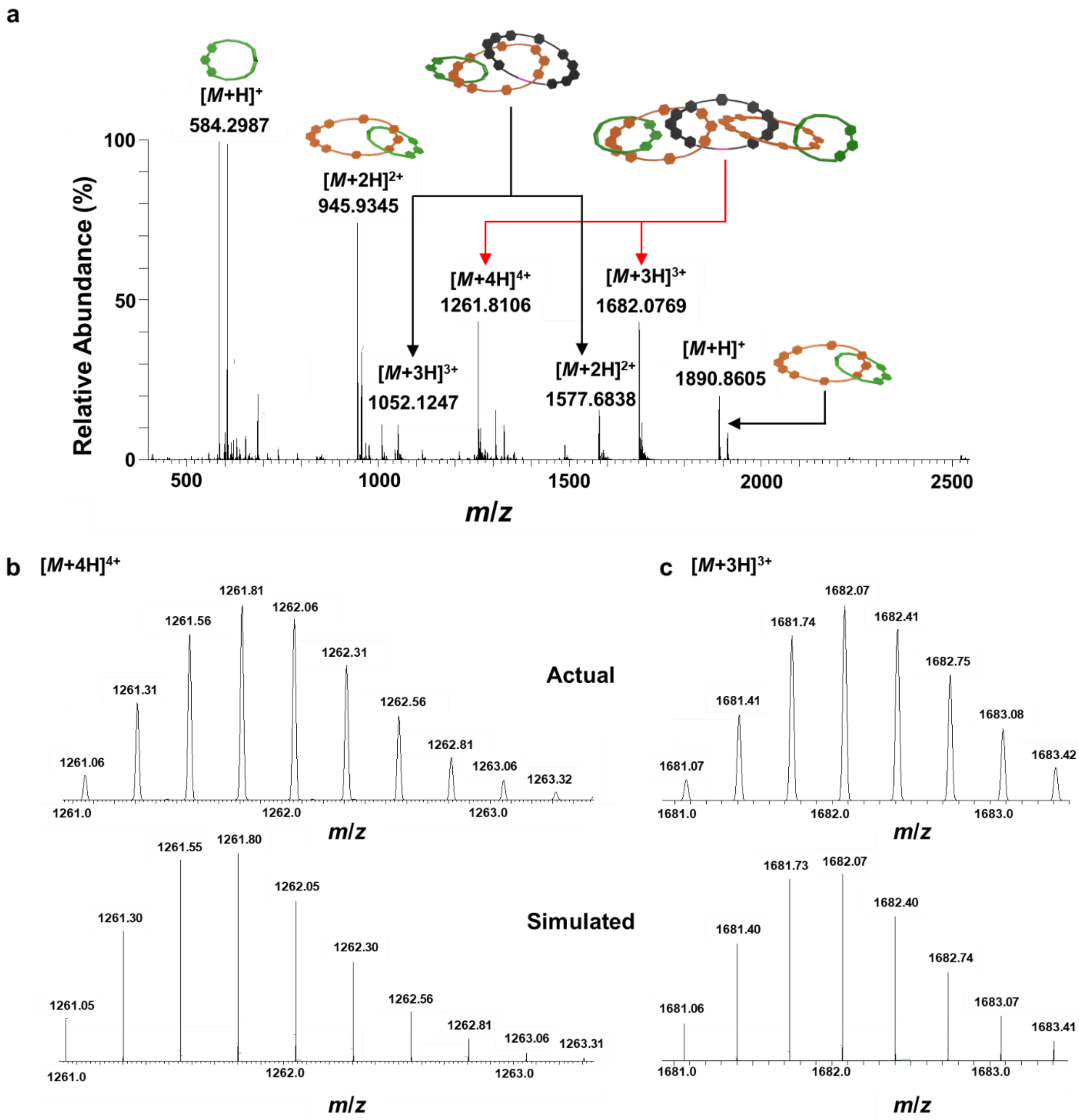

Figure S67. (**a**) Full HRMS-ESI of **[5]C.** (**b**) Zoom-in and simulation of $[M + 4H]^{4+}$ adduct of **[5]C**. (**c**) Zoom-in and simulation of $[M + 3H]^{3+}$ adduct of **[5]C.**

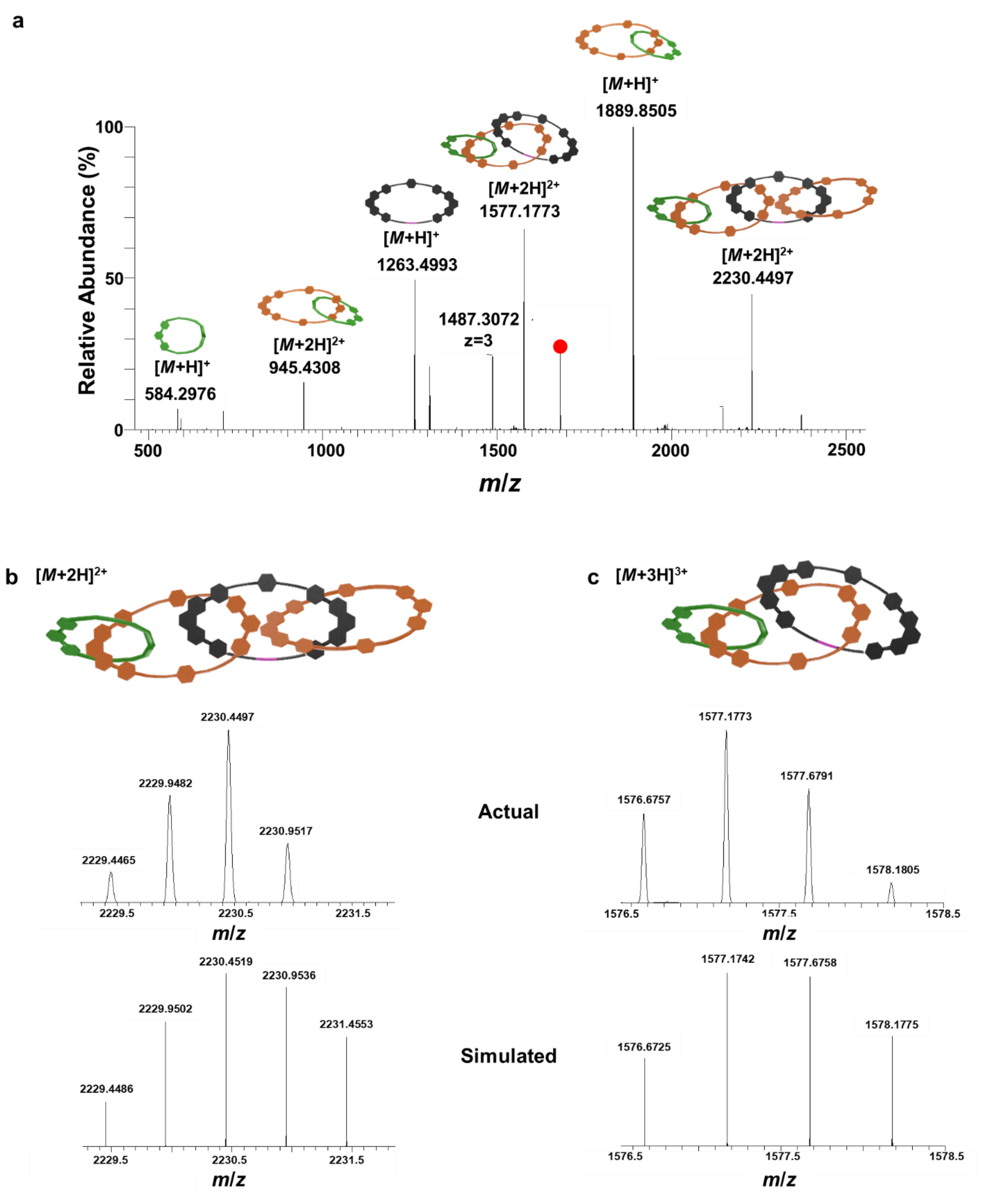

Figure S68. (**a**) Full THRMS-ESI of **[5]C**; isolation and fragmentation of peak at [M +3H]$^{3+}$ = 1682.06. Red circle represents remaining unfragmented **[5]C**. (**b**) Zoomed-in THRMS-ESI [4]catenane fragment. (**c**) Zoomed-in THRMS-ESI of [3]catenane fragment.

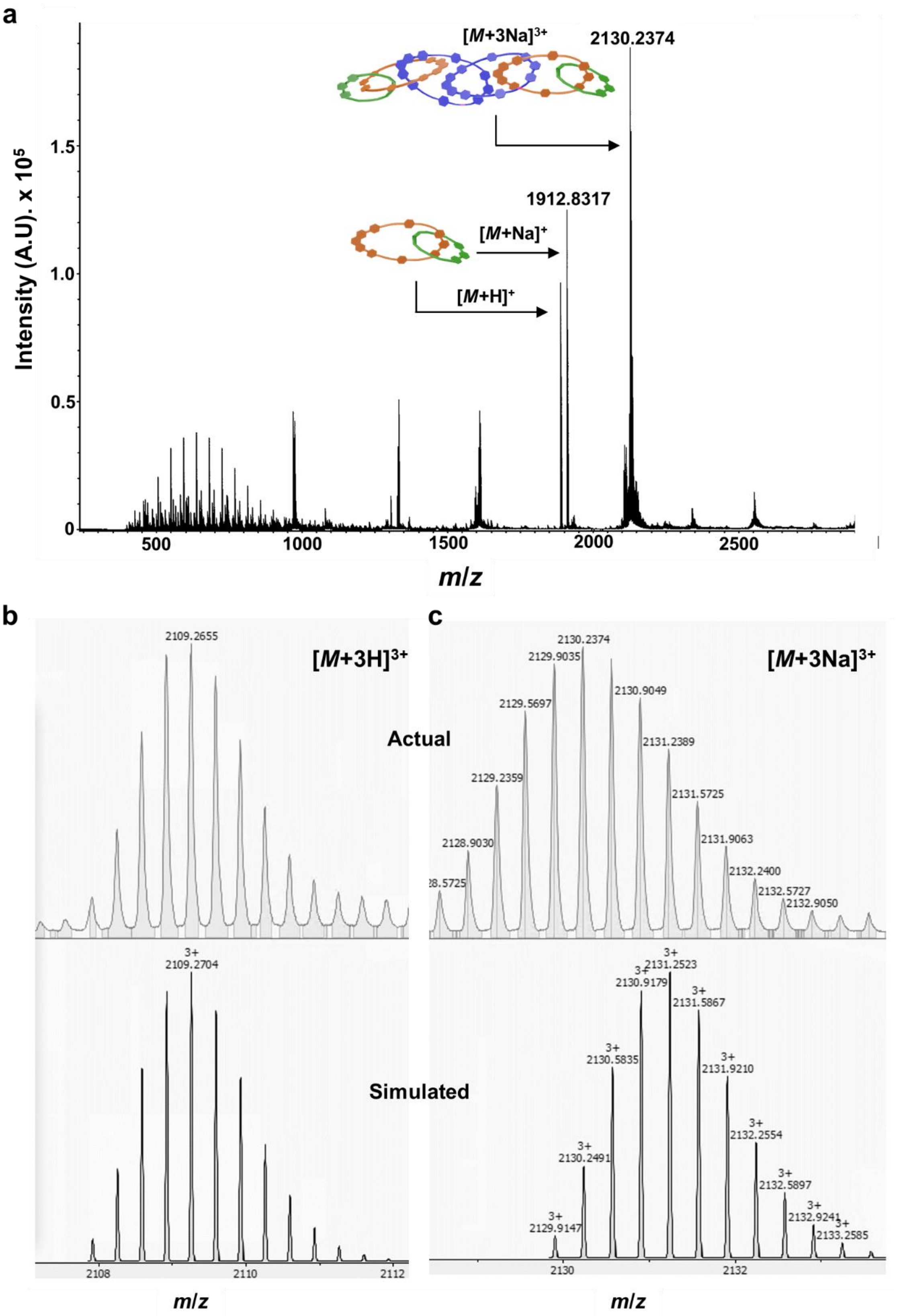

Figure S69. (**a**) Full HRMS-ESI of **[6]C.** (**b**) Zoom-in and simulation of [M + 3H]$^{3+}$ adduct of **[6]C**. (**c**) Zoom-in and simulation of [M + 3Na]$^{3+}$ adduct of **[6]C**.

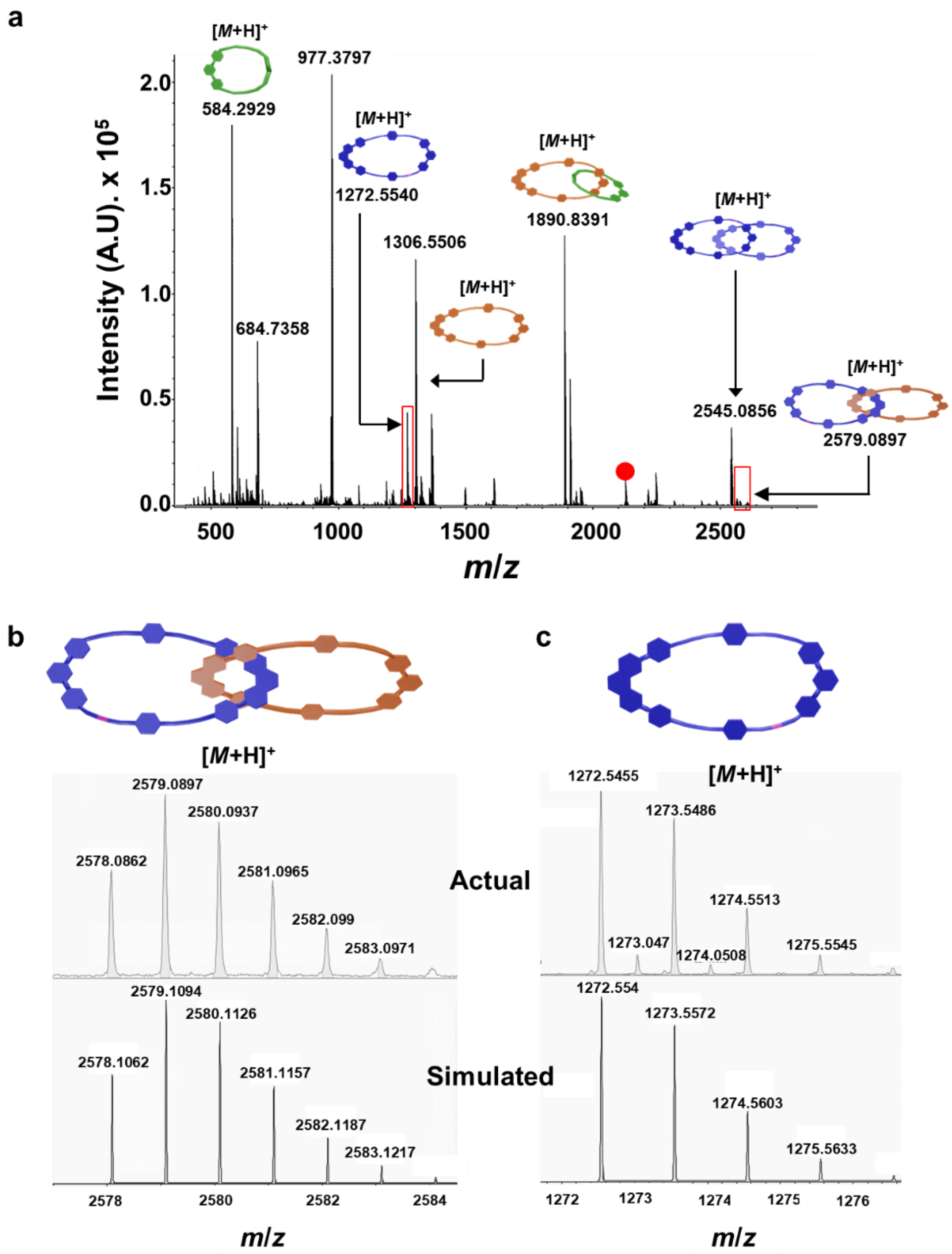

Figure S70. (**a**) Full THRMS-ESI of **[6]C**; isolation and fragmentation of peak at [M +3Na]$^{3+}$ = 2130.23 at 157 eV collision energy. Red circle represents remaining unfragmented **[6]C**. (**b**) Zoomed-in THRMS-ESI mixed macrocycle [2]catenane fragment. (**c**) Zoom-in THRMS-ESI of macrocycle fragment. The subpopulation of peaks corresponds to unfragmented **3** flying as [M +2H]$^{2+}$, which is distinguishable from the macrocycle by its charge state.

The mixed macrocycle [2]catenane and macrocycle fragments could only have originated from the linear [6]catenane **[6]C**, as opposed to a hypothetical linear [5]catenane resulting from figure-of-eight precursor.

2) Matrix-Assisted Laser Desorption/Ionization Time-of-Flight (MALDI-TOF)

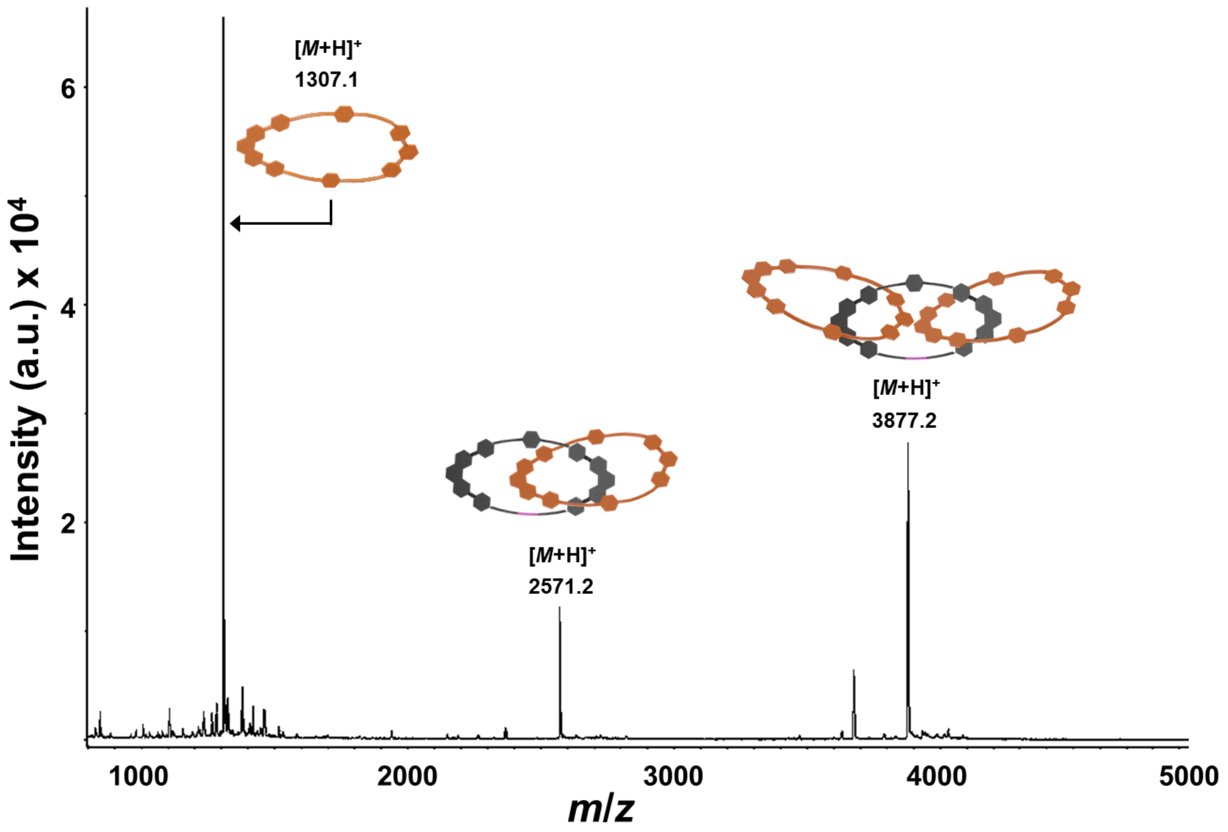

Figure S71. MALDI-TOF of **[3]C** with 2,5-dihydroxybenzoic acid matrix.

The observed asymmetric [2]catenane and macrocycle **3** are due to fragmentation of **[3]C** during MALDI characterization. These fragments are indicative of the linear catenane **[3]C**.

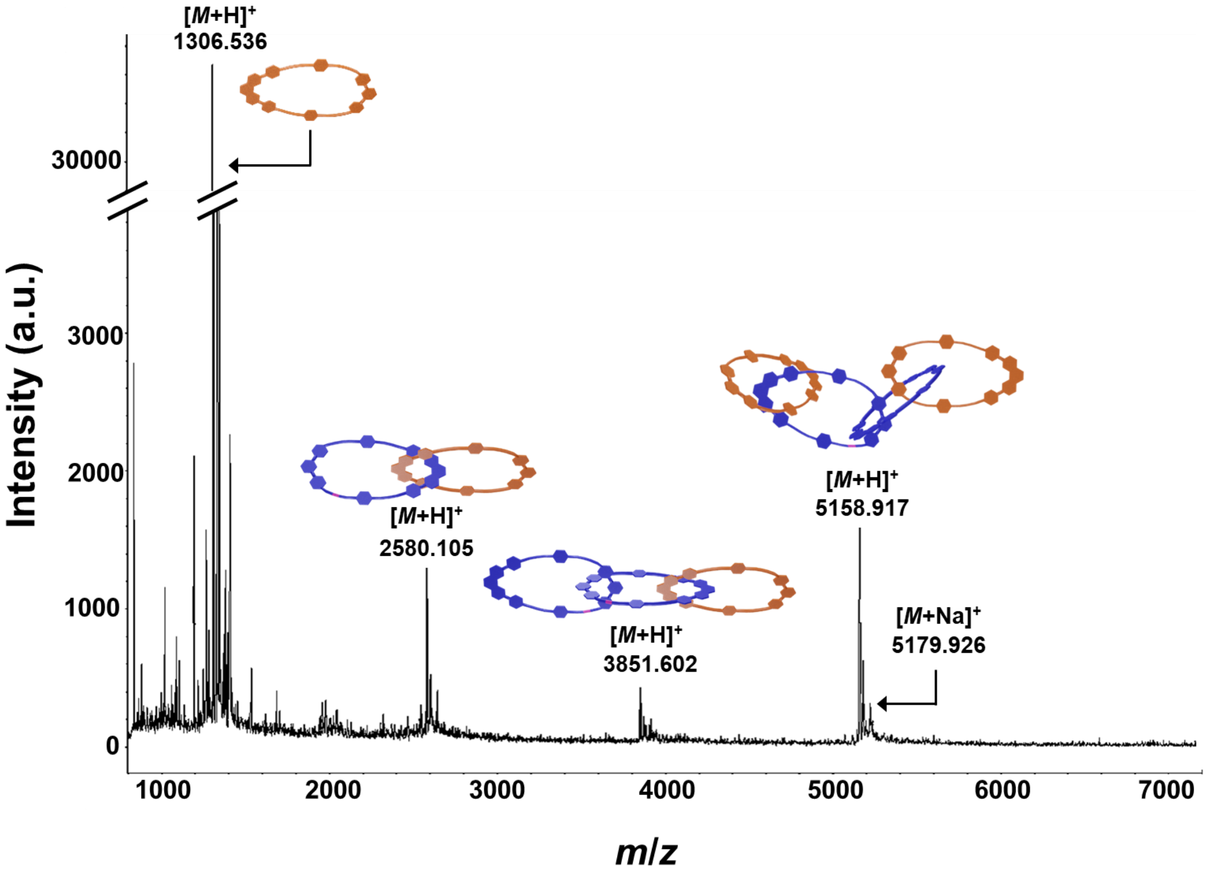

Figure S72. MALDI-TOF of **[4]C** with α-cyano-4-hydroxycinnamic acid matrix.

The MALDI-TOF experiment causes fragmentation of **[4]C**, resulting in [3]catenane, [2]catenane, and macrocycle fragments. The mixed macrocycle [2]catenane with *m*/z = 2580.1 could only be observed from the fragmentation of the linear [4]catenane **[4]C**, as opposed to a hypothetical linear [3]catenane from the figure-of-eight precursor. These results are in excellent agreement with the THRMS-ESI experiments of **[4]C** (Figs. S65-S66), as well as the unimodal GPC (Fig. S51), and HPLC (Fig. S61) traces.

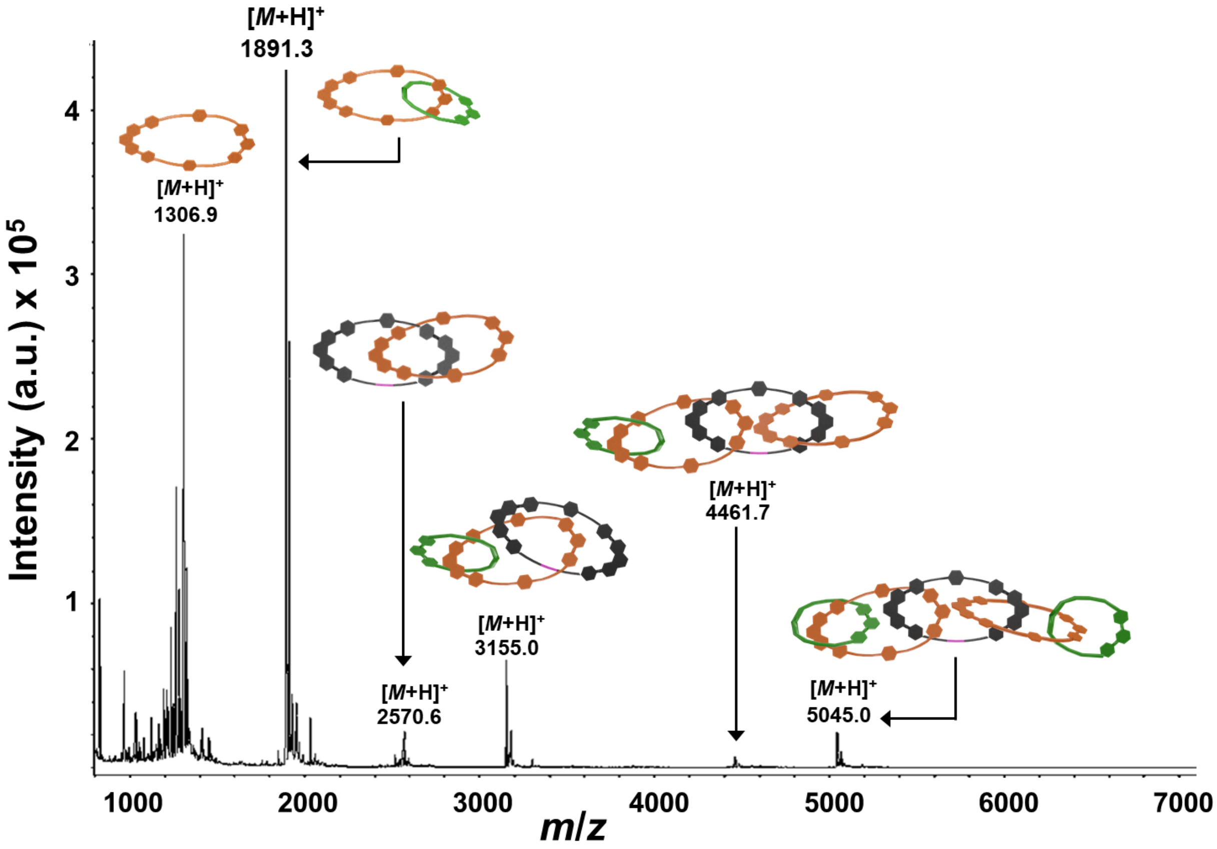

Figure S73. MALDI-TOF of **[5]C** with α-cyano-4-hydroxycinnamic acid matrix.

In addition to the observation of [*M* +H]$^+$ corresponding to an intact [5]catenane, a number of fragments are produced during the ionization process during MALDI characterization; this was also observed for **[3]C**, **[4]C**, and **[6]C**. Fragmentation of **[5]C** results in a linear [4]catenane, [3]catenane, two [2]catenanes, and macrocycle **3**. These fragments are indicative of the catenane topology of **[5]C**.

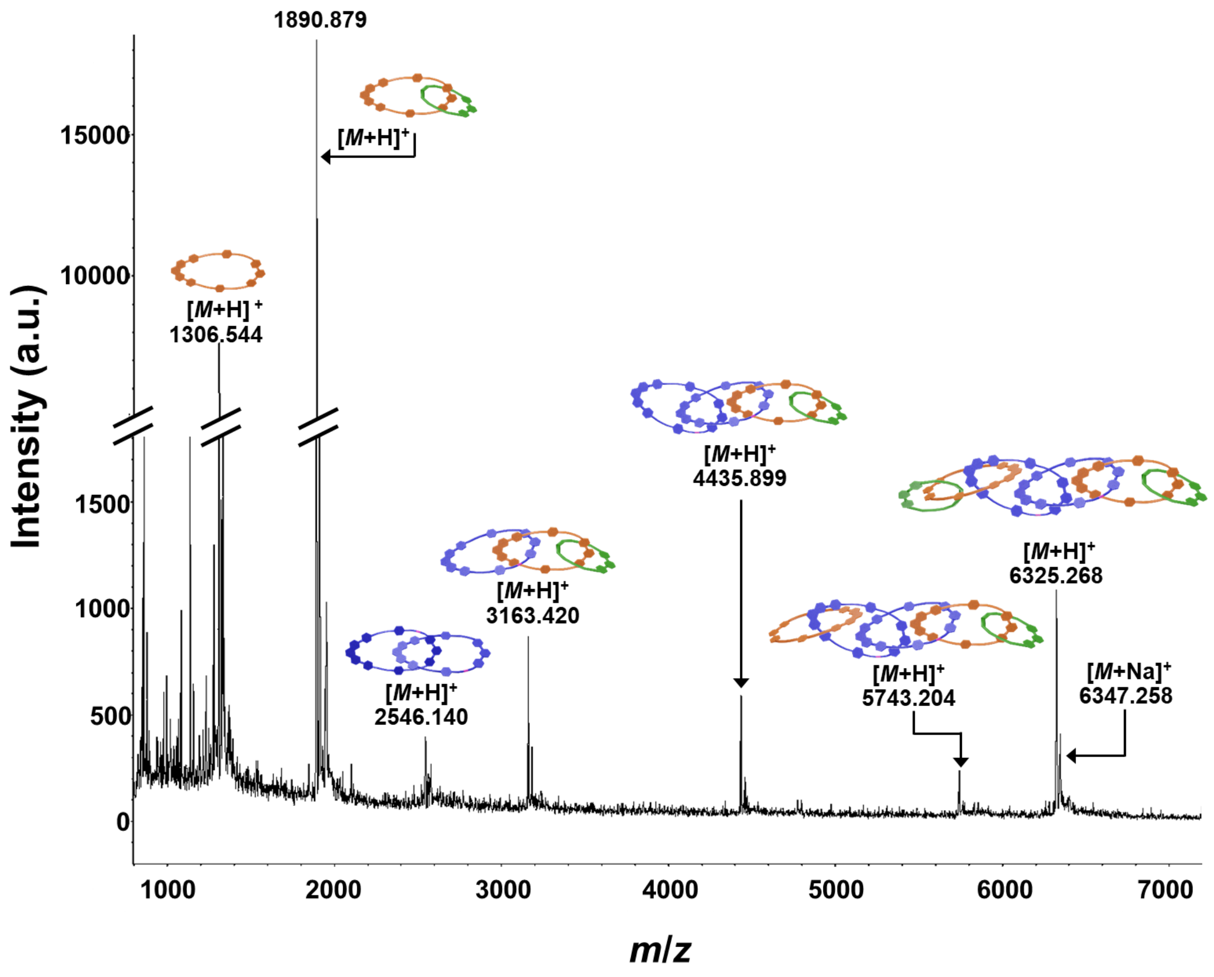

Figure S74. MALDI-TOF of **[6]C** with α-cyano-4-hydroxycinnamic acid matrix.

The MALDI-TOF experiment causes fragmentation of the **[6]C**, resulting in [5]catenane, [4]catenane, [3]catenane, [2]catenane, and macrocyclic fragments. The [3]catenane with *m*/z = 3163.4 could only be observed from the fragmentation of the linear [6]catenane **[6]C**, as opposed to a hypothetical linear [5]catenane from the figure-of-eight precursor. These results are in excellent agreement with the THRMS-ESI experiments of **[6]C** (Figs. S69-S70), as well as the unimodal GPC (Fig. S51), and HPLC (Fig. S61) traces.

3) *Low-res Mass Spectrometry Electrospray Ionization (LRMS-ESI)*

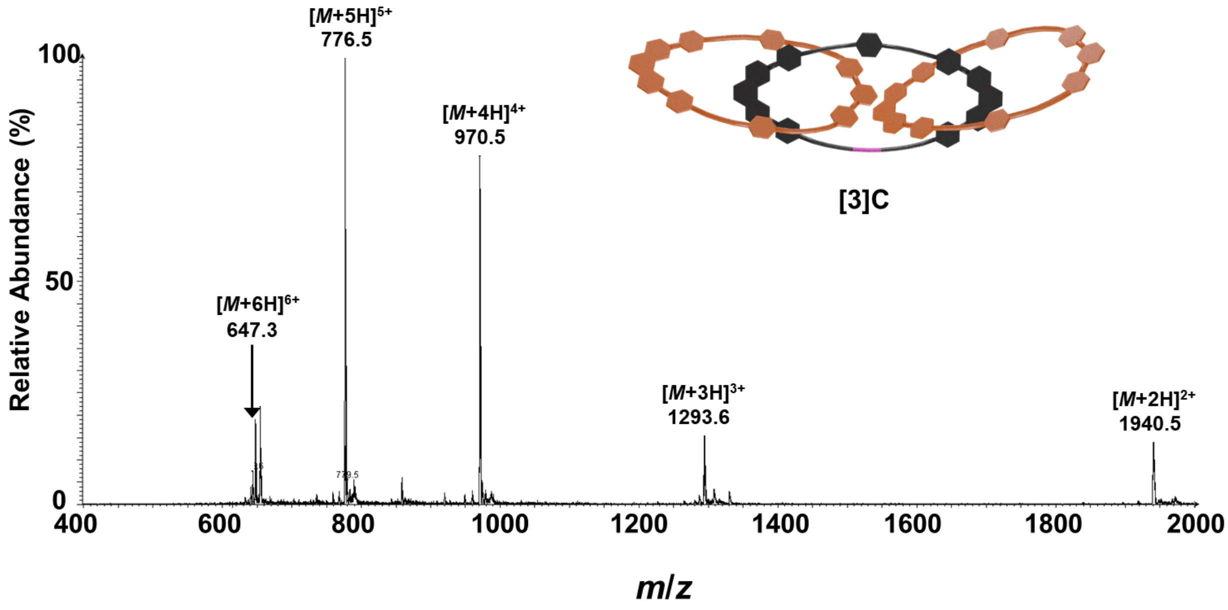

Figure S75. LRMS-ESI spectrum of major peak (8.5 min.) from analytical HPLC of **[3]C** shown in Fig. S61.

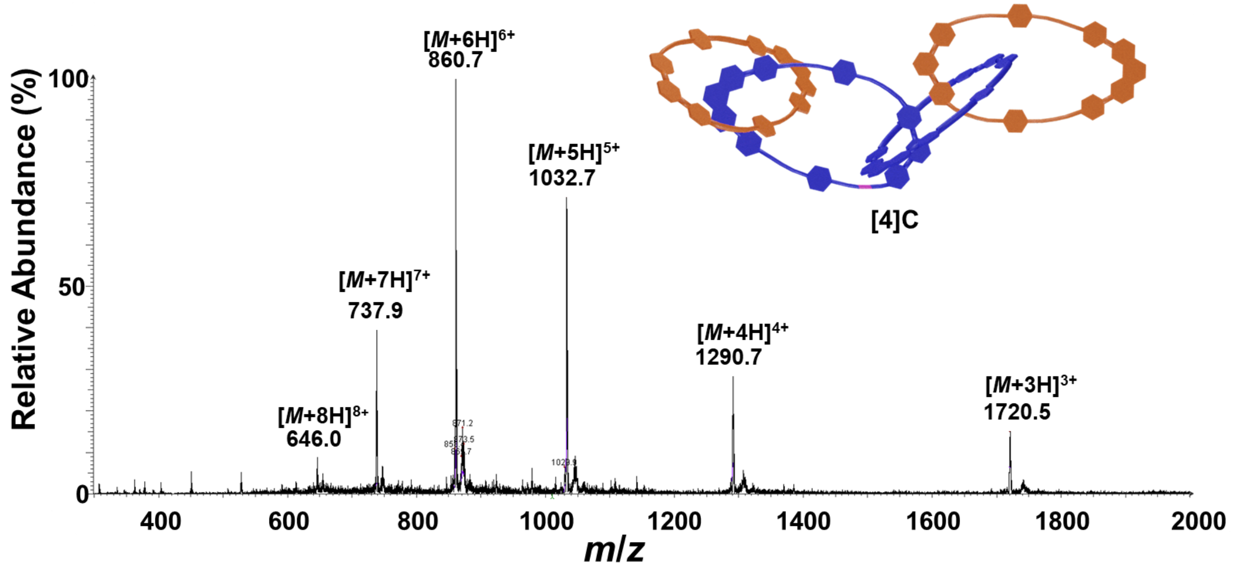

Figure S76. LRMS-ESI spectrum of major peak (9.0 min.) from analytical HPLC of **[4]C** shown in Fig. S61.

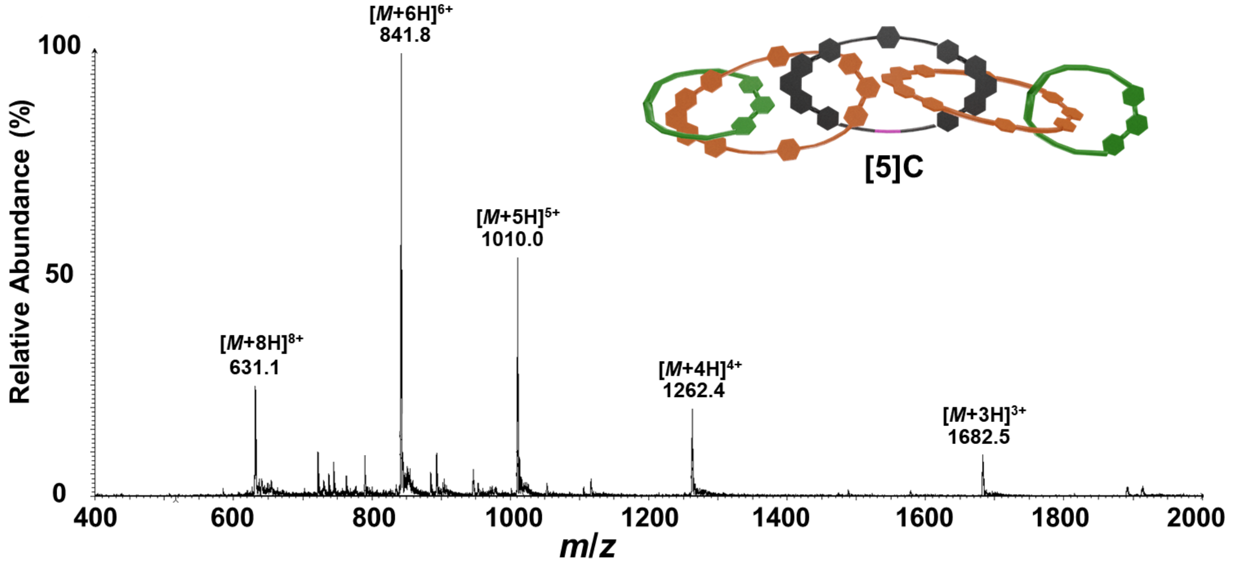

Figure S77. LRMS-ESI spectrum of major peak (8.3 min.) from analytical HPLC of **[5]C** shown in Fig. S61.

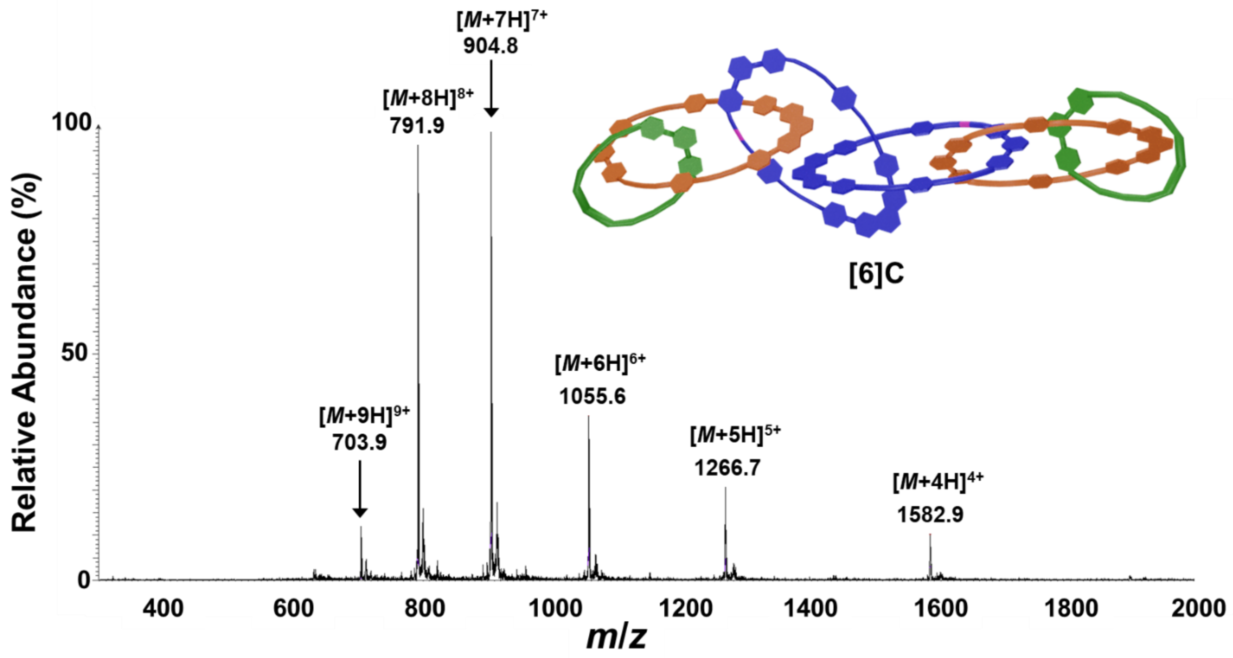

Figure S78. LRMS-ESI spectrum of major peak (8.7 min.) from analytical HPLC of **[6]C** shown in Fig. S61.

Section E. References

1. Colley, N. D.; Nosiglia, M. A.; Li, L.; Amir, F.; Chang, C.; Greene, A. F.; Fisher, J. M.; Li, R.; Li, X.; Barnes, J. C., One-Pot Synthesis of a Linear [4]Catenate Using Orthogonal Metal Templation and Ring-Closing Metathesis. *Inorg. Chem.* **2020,** *59* (15), 10450-10460.
2. Gupta, M.; Kang, S.; Mayer, M. F., A double ring-closing olefin metathesis approach to [3]catenanes. *Tetrahedron Lett.* **2008,** *49* (18), 2946-2950.
3. Korvinson, K. A.; Akula, H. K.; Malinchak, C. T.; Sebastian, D.; Wei, W.; Khandaker, T. A.; Andrzejewska, M. R.; Zajc, B.; Lakshman, M. K., Catalytic Reductions Without External Hydrogen Gas: Broad Scope Hydrogenations with Tetrahydroxydiboron and a Tertiary Amine. *Adv. Synth. Catal.* **2020,** *362* (1), 166-176.
4. De, S.; Pramanik, S.; Schmittel, M., A monomer–dimer nanoswitch that mimics the working principle of the SARS-CoV 3CLpro enzyme controls copper-catalysed cyclopropanation. *Dalton Trans.* **2014,** *43* (28), 10977-10982.
5. Danon, J. J.; Leigh, D. A.; Pisano, S.; Valero, A.; Vitorica-Yrezabal, I. J., A Six-Crossing Doubly Interlocked [2]Catenane with Twisted Rings, and a Molecular Granny Knot. *Angew. Chem. Int. Ed.* **2018,** *57* (42), 13833-13837.
6. Au-Yeung, H. Y.; Yee, C.-C.; Hung Ng, A. W.; Hu, K., Strategies To Assemble Catenanes with Multiple Interlocked Macrocycles. *Inorg. Chem.* **2018,** *57* (7), 3475-3485.
